# Adamantaniline Derivatives Target ATP5B to Inhibit Translation of Hypoxia Inducible Factor‐1*α*

**DOI:** 10.1002/advs.202301071

**Published:** 2023-07-03

**Authors:** Huiti Li, Yali Liu, Zian Xue, Li Zhang, Xiaoxue Ruan, Jintong Yang, Zhongjiao Fan, Hongfang Zhao, Yu Cao, Guoqiang Chen, Ying Xu, Lu Zhou

**Affiliations:** ^1^ Department of Medicinal Chemistry School of Pharmacy Fudan University 826 Zhangheng Road Shanghai 201203 P. R. China; ^2^ Institute of Aging & Tissue Regeneration National Key Laboratory of Cancer Systems Medicine and Chinese Academy of Medical Sciences Research Unit (NO.2019RU043) Renji Hospital Shanghai Jiao Tong University School of Medicine Shanghai 200025 China; ^3^ Institute of Precision Medicine the Ninth People's Hospital Shanghai Jiao Tong University School of Medicine 115 Jinzun Road Shanghai 200125 China; ^4^ Key Laboratory of Cell Differentiation and Apoptosis of Chinese Ministry of Education Shanghai Jiao Tong University School of Medicine Shanghai 200025 China

**Keywords:** adamantaniline derivatives, ATP5B, HIF‐1*α*, target identification

## Abstract

Hypoxia inducible factor‐1*α* (HIF‐1*α*) plays a critical role in cellular adaptation to hypoxia and it is a potential therapeutic target for anti‐cancer drugs. Applying high‐throughput screening, here it is found that HI‐101, a small molecule containing an adamantaniline moiety, effectively reduces HIF‐1*α* protein expression. With the compound as a hit, a probe (HI‐102) is developed for target identification by affinity‐based protein profiling. The catalytic *β* subunit of mitochondrial F_O_F_1_‐ATP synthase, ATP5B, is identified as the binding protein of HI‐derivatives. Mechanistically, HI‐101 promotes the binding of HIF‐1*α* mRNA to ATP5B, thus inhibiting HIF‐1*α* translation and the following transcriptional activity. Further modifications of HI‐101 lead to HI‐104, a compound with good pharmacokinetic properties, exhibiting antitumor activity in MHCC97‐L mice xenograft model, and HI‐105, the most potent compound with an IC_50_ of 26 nm. The findings provide a new strategy for further developing HIF‐1*α* inhibitors by translational inhibition through ATP5B.

## Graphical abstract

By means of high‐throughput screening, structure modification, and mechanism study, HI‐derivatives, containing an adamantaniline moiety are found, inhibiting hypoxia inducible factor‐1*α* (HIF‐1*α*) transcriptional activity by promoting the binding of HIF‐1*α* mRNA to ATP5B in a FoF_1_‐ATP synthase enzyme activity‐independent manner. The findings provide a new strategy for further developing HIF‐1*α* inhibitors by translational inhibition through ATP5B.

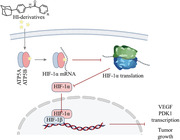

## 1 Introduction

Cancer is the second deadliest disease in the world, accounting for nearly 10 million deaths in 2020.^[^ ^1^ ^]^ A striking characteristic feature of tumor cells is that they can escape senescence and proliferate indefinitely, which results in the formation of a hypoxic microenvironment.^[^ ^2^, ^3^, ^4^ ^]^ Cells usually undergo a series of changes to adapt to the hypoxia microenvironment, such as the upregulation of vascular endothelial growth factor (VEGF), pyruvate dehydrogenase kinase isoenzyme 1 (PDK1) and glucose transporter 1 (GLUT1).^[^ ^5^, ^6^ ^]^ These detrimental events might promote tumor angiogenesis, growth, and metastatic, enhancing resistance to the anti‐tumor immune response at the same time in many solid malignancies.^[^ ^7^, ^8^, ^9^ ^]^ A growing number of studies show that targeting the hypoxic microenvironment of malignant tumors is a promising therapeutic strategy to inhibit tumor growth.^[^ ^10^, ^11^, ^12^ ^]^

Maintaining oxygen homeostasis is extremely important for tumor cell growth. Hypoxia inducible factor‐1 (HIF‐1), consisting of the hypoxic response factor—HIF‐1*α* and the constitutively expressed aryl hydrocarbon receptor nuclear translocator—HIF‐1*β*, is a transcription factor that is highly responsive to the hypoxic environment and is widely distributed throughout the human body.^[^ ^13^ ^]^ Hydroxylated HIF‐1*α* protein at P402 and P564 by prolyl‐4‐hydroxylases (PHDs) is recognized and ubiquitinated by the von Hippel−Lindau (VHL) E3‐ubiquitin ligase complex and then degraded by the 26S proteasome subsequently under normoxia condition. However, inactivated PHDs fail to hydroxylate HIF‐1*α* under hypoxia condition, which results in the stabilization and accumulation of HIF‐1*α*. Stabilized HIF‐1*α* can translocate to nuclear and dimerize with HIF‐1*β*, which induces the transcription of a series of downstream target genes, including VEGF and PDK1, to promote tumor growth and metastasis.^[^ ^14^, ^15^, ^16^, ^17^ ^]^ Recent studies suggest that HIF‐1*α* protein is highly overexpressed in various types of tumors, especially in solid tumors and the dramatic overexpression of HIF‐1*α* is closely associated with poor prognosis.^[^ ^18^ ^]^ A large number of clinical studies have shown correlations between HIF‐1*α* and the metastasis, recurrence, vascular proliferation, and prognosis in cancer patients.^[^ ^19^ ^]^ A previous report showed the expression level of HIF‐1*α* in liver cancer tissues is higher than in corresponding adjacent tissues and the overexpression of HIF‐1*α* is also associated with poor prognosis in liver cancer patients.^[^ ^20^ ^]^ Thus, HIF‐l*α* is considered to be a promising target for liver cancer treatment and multiple HIF‐1*α* inhibitors have been developed and are undergoing preclinical or clinical trials to date.^[^ ^21^, ^22^ ^]^

**Figure 1:**
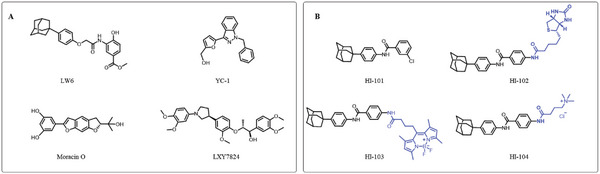
Structures of A) representative HIF‐1*α* inhibitors and B) adamantaniline derivatives in this work.

Mechanistically, HIF‐1*α* inhibitors can be mainly classified into two broad categories: inhibition of HIF‐1*α* transcriptional activity in either direct or indirect ways and induction of HIF‐1*α* degradation. In recent years, a number of novel small‐molecule inhibitors have been identified to inhibit HIF‐1*α* transcriptional activity or induce HIF‐1*α* degradation^[^ ^21^, ^23^ ^]^ (**Figure** **1**A), including (aryloxyacetylamino) benzoic acid derivatives (LW6), reducing HIF‐1*α* accumulation by inhibiting malate dehydrogenase 2 (MDH2) activity;^[^ ^24^, ^25^ ^]^ indazole/benzimidazole derivatives (YC‐1), downregulating HIF‐1*α* translational initiation by suppressing the PI3K/Akt/mTOR/4E‐BP pathway;^[^ ^26^, ^27^ ^]^ benzofuran derivatives (moracin O), inhibiting the initiation of HIF‐1*α* translation by binding to heterogeneous nuclear ribonucleoprotein A2B1(hnRNPA2B1);^[^ ^28^, ^29^ ^]^ Manassantin A derivatives (LXY7824), reducing HIF‐1*α* expression level via VHL‐ proteasome degradation system^[^ ^30^, ^31^ ^]^ and so forth.^[^ ^32^, ^33^, ^34^, ^35^ ^]^ Unfortunately, there is no HIF‐1*α* inhibitor approved by Food and Drug Administration (FDA) for the cancer treatment to date due to unsatisfied therapeutic effects or unexpected side effects. Thus, it is still an urgent need to discover novel HIF‐1*α* inhibitors with brand‐new mechanism, potential potency, excellent druggability as well as low toxicity.

Herein, we report HI‐101, a hit compound with an adamantaniline moiety screened by a cell‐based HIF‐1*α* transcriptional activity assay (Figure 1B). We discover that HI‐101 inhibits HIF‐1*α* transcriptional activity by decreasing HIF‐1*α* protein level as a translation inhibitor. With modified probes HI‐102 and HI‐103, we identify ATP synthase subunit beta (ATP5B) as a potential target which HI‐derivatives might interact with. Mechanism of action study reveals that HI‐derivatives interfere with HIF‐1*α* mRNA binding to ribosome by enhancing ATP5B binding to HIF‐1*α* mRNA. Further modification led to HI‐104, a compound with better pharmacokinetic properties, showing therapeutic effects on MHCC97‐L mice xenograft model. This study provides new insight for better understanding HIF‐1*α* translation related signaling pathways and demonstrates that ATP5B might be a promising target for the development of HIF‐1*α* inhibitors as anticancer agents.

## 2 Experiments and Results

### 2.1 Identification of Hit Compounds by High‐Throughput Screening

To screen HIF‐1*α* inhibitors in a robust cell‐based assay, we optimized the dual‐luciferase assay with pLenti‐HIF‐1*α*P2A (P402A and P564A) instead of pLenti‐HIF‐1*α* in order to ensure the expression of HIF‐1*α* under normoxia condition. In the cell‐based assay, HEK293T cells were co‐transfected with pLenti‐HIF‐1*α*P2A, renilla, and hypoxia‐responsive element (HRE)‐Firefly Luciferase (Figure S1, Supporting Information). Inhibition of HIF‐1*α* transcriptional activity by compounds decreased the expression of firefly luciferase resulting in attenuating firefly luminescence, and renilla luminescence was an internal reference for cell numbers. The inhibition rates (IRs) of screened compounds were initially measured at a compound concentration of 10 µm after incubation for 24 h. Three compounds were selected as the positive controls including two inhibitors (PX‐478 2HCl and BAY 87‐2243) and an activator (BAY 85‐3934) in the screening assay.

A collection of 101 254 compounds in the National Compound Library of the Shanghai Institute of Materia Medica, Chinese Academy of Sciences, were screened based on dual‐luciferase assay. Fifteen inhibitors were yielded to decrease significantly the firefly/renilla luciferase signal in HEK293T at a concentration of 10 µm (Figure S2, Supporting Information).

### 2.2 Hit Compound Confirmation

**Figure 2:**
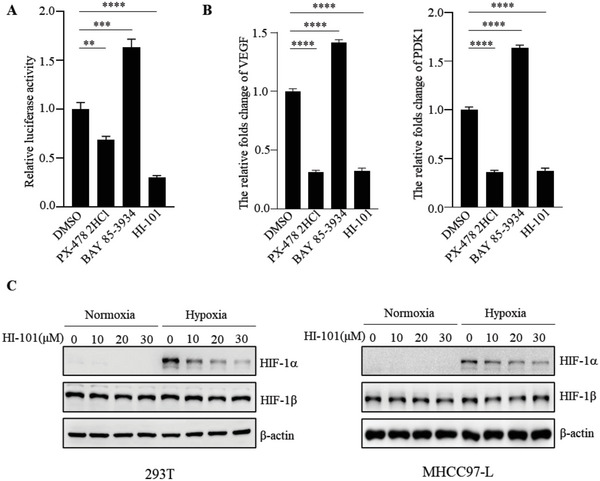
Effect of HI‐101 on HIF‐1*α* transcriptional activity and protein level. A) Activity measurement of HI‐101 and positive controls on dual‐luciferase assay. B) Effects of HI‐101 and positive controls on HIF‐1*α* target genes expression, including VEGF and PDK1. C) Expressions of HIF‐1*α* were examined in HEK293T and MHCC97‐L cells treated with HI‐101 at indicated concentrations. *p < 0.05, **p < 0.01, ***p < 0.001, ****p < 0.0001(two‐tailed Student's *t*‐test for unpair wise comparisons). Data are mean ± SD.

To validate the results of the primary screening and exclude false positives for further studies, we reperformed luciferase assays and confirmed that these fifteen hit compounds decreased the luciferase activity in a concentration‐dependent manner (Figure S3, Supporting Information). Next, we examined the effects of these compounds on the expressions of HIF‐1*α* targeted genes by quantitative RT‐PCR. As shown in Figure S4, Supporting Information, all these fifteen hit compounds significantly reduced HIF‐1*α* target genes (VEGF and PDK1) mRNA expression. Besides, we examined their effects on HIF‐1*α* protein level and the results showed that compounds 3, 5, 8, 9, 11, and 14 all significantly reduced HIF‐1*α* protein level while the other compounds had almost no effect on HIF‐1*α* protein level (Figure S5, Supporting Information). The same effects of these fifteen hit compounds on HIF‐1*α* transcriptional activity and target genes expressions were observed in HEK293T cells under hypoxia condition (Figure S6, Supporting Information). We selected compounds with the same core structure (8 and 9) for further study after excluding pan‐assay interference compounds.^[^ ^36^ ^]^ Results showed that the hit compound 8 (named as HI‐101) significantly inhibited HIF‐1*α* transcriptional activity and the expressions of HIF‐1*α* target genes (**Figure** **2**A,B). In addition, HI‐101 specifically reduced the protein level of HIF‐1*α* in a dose‐dependent manner under hypoxic condition in both HEK293T and MHCC97‐L cells (Figure 2C).

### 2.3 Hit Compound Optimization and Probe Design

In order to obtain a potent bio‐active probe for target identification, we performed the structure activity relationship (SAR) study. HI‐101 was further studied in two regions, the phenyl ring (region A) and the adamantane phenyl group (region B). First, the phenyl ring in region A was modified with small hydrophobic and hydrophilic groups as well as long chain substituents. As shown in **Tables** **1** and **2**, compounds containing hydrophilic groups, such as amino (1‐3s–1‐3u) and hydroxyl (1‐3m–1‐3o) were much more potent than compounds with lipophilic groups, such as hexyloxyl (1‐3ai and 1‐3aj) and octyl (1‐3ak), suggesting that the substituents at this position might lie in a hydrophilic pocket. In addition, substitutions at the ortho position led to greatly reduced or loss of activity while para‐ and meta‐substitutions were tolerable and the activity of compounds with long hydrophilic chains was sustained. Besides, the activity was mainly reserved when the benzene ring was replaced by various aromatic heterocyclic rings (1‐4a–1‐4r) (**Table** **3**). Second, we explored if the adamantane phenyl group (region B) was necessary for inhibiting HIF‐1*α* transcriptional activity. Compounds either with substitutions (methyl or hydroxyl) at the adamantane group or linkers between adamantane and phenyl group were designed and synthesized. As shown in **Tables** **4** and **5**, both substitutions at the adamantane group and introduction of linkers remarkably decreased activities which suggested the adamantane group was very important. Finally, a biotin group was introduced at the para‐position to give probe HI‐102 for target identification and a bodipy group was introduced at the same position to obtain probe HI‐103 for mechanism study.

**Table 1:** HIF‐1*α* inhibitory activity of compounds 1‐3a–1‐3z.

Compound^a)^	*R* ^1^	Inhibition rate [%] @ 10 µm*	IC_50_ [µm]*
1‐3a(HI‐101)	3‐Cl	81.4 ± 0.9	2.0 ± 0.3
1‐3b	2‐Cl	58.4 ± 1.5	–
1‐3c	4‐Cl	63.4 ± 5.4	–
1‐3d	2‐Br	52.3 ± 3.1	–
1‐3e	3‐Br	86.3 ± 0.2	1.4 ± 0.3
1‐3f	4‐Br	67.1 ± 5.1	–
1‐3g	2‐OMe	40.2 ± 2.1	–
1‐3h	3‐OMe	92.1 ± 0.6	0.82 ± 0.13
1‐3i	4‐OMe	86.0 ± 1.4	1.6 ± 0.3
1‐3j	2‐CN	41.8 ± 3.4	–
1‐3k	3‐CN	67.4 ± 3.8	–
1‐3l	4‐CN	61.2 ± 5.5	–
1‐3m	2‐OH	88.4 ± 0.2	0.51 ± 0.02
1‐3n(HI‐105)	3‐OH	99.7 ± 0.1	0.026 ± 0.005
1‐3o	4‐OH	96.4 ± 0.2	0.053 ± 0.008
1‐3p	2‐*tert*‐butyl carbamate	40.3 ± 3.2	–
1‐3q	3‐*tert*‐butyl carbamate	75.5 ± 0.3	3.5 ± 0.2
1‐3r	4‐*tert*‐butyl carbamate	54.6 ± 1.6	–
1‐3s	2‐NH_2_	90.4 ± 1.7	0.54 ± 0.02
1‐3t	3‐NH_2_	97.3 ± 0.4	0.23 ± 0.03
1‐3u	4‐NH_2_	98.4 ± 0.1	0.11 ± 0.01
1‐3v	2‐acetamido	18.4 ± 2.2	–
1‐3w	3‐acetamido	77.4 ± 2.3	1.9 ± 0.2
1‐3x	4‐acetamido	84.4 ± 1.9	0.95 ± 0.11
1‐3y(HI‐102)	4‐NH‐biotin	90.4 ± 0.8	0.28 ± 0.10
1‐3z(HI‐103)	4‐NH‐bodipy	80.3 ± 1.2	2.1 ± 0.5
LW6		70.9 ± 3.7	2.6 ± 0.5

^a)^ The data are presented as mean ± s.d.(*n* = 3); N.D.: not determined; ‐: not detected; LW6 as a positive control.

**Table 2:** HIF‐1*α* inhibitory activity of compounds 1‐3aa–1‐3au

Compound^a)^	R^1^	Inhibition rate [%] @10 µm	IC_50_ [µm]*
1‐3aa	2‐dimethylamino	38.9 ± 3.4	–
1‐3ab	3‐dimethylamino	60.5 ± 3.1	–
1‐3ac	4‐dimethylamnio	40.9 ± 5.7	–
1‐3ad	2‐CF_3_	31.3 ± 3.3	–
1‐3ae	3‐CF_3_	20.2 ± 1.5	–
1‐3af	4‐CF_3_	37.9 ± 0.7	–
1‐3ag	4‐morpholinyl	41.6 ± 8.9	–
1‐3ah	4‐pyrrolidinyl	66.5 ± 0.9	–
1‐3ai	2‐hexyloxyl	28.2 ± 5.5	–
1‐3aj	3‐hexyloxyl	28.4 ± 6.9	–
1‐3ak	4‐octyl	32.5 ± 7.2	–
1‐3al	3‐NHCO(CH_2_)_4_Br	89.5 ± 1.1	1.0 ± 0.1
1‐3am	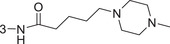	86.4 ± 0.9	1.3 ± 0.2
1‐3an	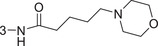	93.4 ± 0.5	0.37 ± 0.04
1‐3ao	4‐NHCO(CH_2_)_4_Br	82.9 ± 2.4	2.3 ± 0.5
1‐3ap	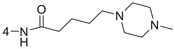	88.8 ± 0.2	1.4 ± 0.5
1‐3aq	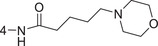	69.5 ± 0.5	–
1‐3ar	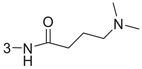	79.4 ± 1.8	2.1 ± 0.4
1‐3as	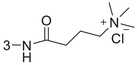	76.4 ± 1.3	2.5 ± 0.7
1‐3at	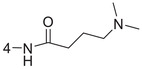	90.0 ± 1.7	0.94 ± 0.02
1‐3au (HI‐104)	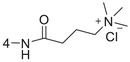	89.9 ± 1.6	1.3 ± 0.3

^a)^ The data are presented as mean ± s.d.(*n* = 3); N.D.: not determined; ‐: not detected; LW6 as a positive control.

**Table 3:** HIF‐1*α* inhibitory activity of compounds 1‐4a–1‐4r.

Compound^a)^	*R* ^2^	Inhibition rate [%] @10 µm	IC_50_ [µm]
1‐4a	2‐pyridyl	99.7 ± 0.2	0.065 ± 0.022
1‐4b	3‐pyridyl	75.2 ± 1.9	–
1‐4c	4‐pyridyl	41.6 ± 2.0	–
1‐4d	4‐pyrimidine	97.1 ± 0.4	0.28 ± 0.06
1‐4e	5‐methylpyrazine	36.3 ± 4.2	–
1‐4f	2‐5‐methoxypyridyl	36.1 ± 5.6	–
1‐4g	2‐4‐methoxypyridyl	68.5 ± 0.4	–
1‐4h	2‐4‐chloropyridyl	91.5 ± 1.3	0.72 ± 0.02
1‐4i	5‐thiazole	97.1 ± 0.4	0.52 ± 0.02
1‐4j	2‐thiophene	98.7 ± 0.6	0.39 ± 0.01
1‐4k	4‐1*H*‐imidazole	99.7 ± 0.2	0.086 ± 0.003
1‐4l	5‐1‐methyl‐1*H*‐imidazole	97.8 ± 0.6	0.083 ± 0.003
1‐4m	3‐1*H‐*pyrazole	99.8 ± 0.1	0.079 ± 0.002
1‐4n	2‐1‐methyl‐1*H*‐pyrrole	57.5 ± 2.0	–
1‐4o	3‐thiophene	48.5 ± 8.7	–
1‐4p	2‐4‐aminopyridyl	99.2 ± 0.1	0.073 ± 0.002
1‐4q	2‐6‐aminopyridyl	98.6 ± 0.1	0.11 ± 0.02
1‐4r	2‐3‐aminopyridyl	97.5 ± 1.0	0.17 ± 0.02

^a)^ The data are presented as mean ± s.d.(*n* = 3); N.D.: not determined; ‐: not detected; LW6 as a positive control.

**Table 4:** HIF‐1*α* inhibitory activity of compounds 2‐3a–2‐3e

Compound^a)^	Structure	Inhibition rate [%] @ 10 µm*	IC_50_ [µm]*
2‐3a	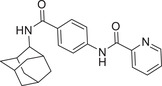	78.5 ± 4.5	1.5 ± 0.2
2‐3b	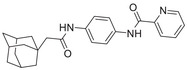	68.0 ± 4.7	3.5 ± 1.0
2‐3c	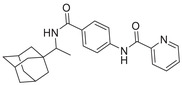	79.9 ± 2.0	2.1 ± 0.4
2‐3d	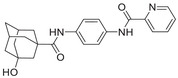	N.D.	–
2‐3e	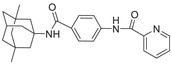	N.D.	–

^a)^ The data are presented as mean ± s.d.(*n* = 3); N.D.: not determined; ‐: not detected; LW6 as a positive control.

**Table 5:** HIF‐1*α* inhibitory activity of compounds 3‐2a–3‐2i^a)^

Compound	Structure	Inhibition rate [%] @ 10 µm*	IC_50_ [µm]*
3‐2a	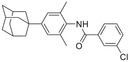	34.8 ± 3.8	–
3‐2b	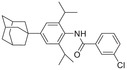	27.0 ± 4.9	–
3‐2c	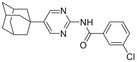	65.3 ± 4.3	–
3‐2d	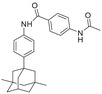	31.4 ± 4.5	–
3‐2e	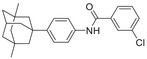	27.1 ± 1.0	–
3‐2f	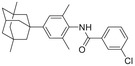	65.7 ± 6.7	–
3‐2g	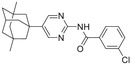	27.8 ± 8.8	–
3‐2h	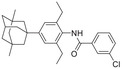	30.0 ± 8.4	–
3‐2i	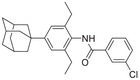	35.1 ± 5.7	–

^a)^ The data are presented as mean ± s.d.(*n* = 3); N.D.: not determined; ‐: not detected; LW6 as a positive control.

### 2.4 Target Identification and Validation of HI‐Derivatives

**Figure 3:**
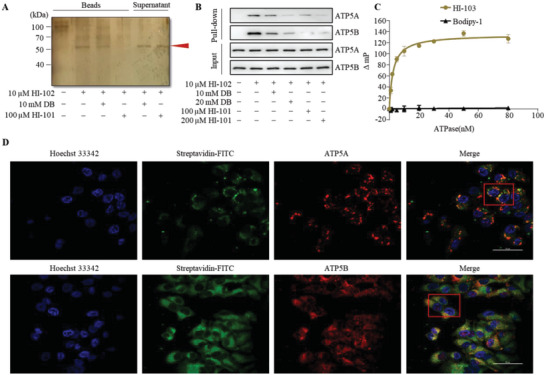
Target identification and validation of HI derivatives. A) Silver staining of HI‐102 binding protein from HEK293T cell lysate using HI‐101 and desulfurization biotin (DB) as competitive compounds. B) Western blot analysis of HI‐102 pull‐down protein using ATP5A and ATP5B antibodies. C) Saturation binding curve of fluorescence polarization assay. F_1_‐ATP synthase at indicated concentrations was incubated with HI‐103 or bodipy‐1 (4 nm), respectively. D) Immunofluorescent imaging of HI‐102 (green) and ATP5A/ATP5B (red) showing co‐localization (merge) in HEK293T cells. Scale bars, 50 µm.

Affinity‐based protein profiling has been widely used as a powerful chemical proteomic strategy to discover the direct target of compounds.^[^ ^37^, ^38^, ^39^ ^]^ To identify the target of HI‐102, pull‐down assays were carried out as described in the experimental procedures. The on‐beads samples were performed with silver staining after SDS‐PAGE and a clear protein band with molecular weights between 50 and 70 kDa was detected in HI‐102 treatment group (**Figure** **3**A). The band was cut and sent for protein analysis by mass spectrometry in 2 replicates. ATP5B was found in both samples and ATP synthase alpha (ATP5A) was found for once from the mass spectrometry analysis (Figure S7, Supporting Information). Competitive ABPP experiments in *vitro* using desulfurization biotin and HI‐101 at indicated concentrations were performed and western blot analysis supported that ATP5A and ATP5B were the possible targets of HI‐102 (Figure 3B). Confocal immunofluorescence experiment was also performed and revealed that HI‐102 co‐localized with ATP5A and ATP5B respectively (Figure 3D).

ATP5A and ATP5B are important components of F_O_F_1_–ATP synthase, also known as ATP synthase, which is a protein complex that catalyzes ATP synthesis by utilizing energy from the translocation of protons across biological membranes.^[^ ^40^ ^]^ F_O_F_1_‐ATP synthase consists of two domains: the membrane‐spanning domain, F_O_, which acts as a proton channel, and the soluble catalytic domain, F_1_, which synthesizes ATP and is composed of nine subunits(*α*_3_ *β*_3_ *γδ*ɛ).^[^ ^41^, ^42^, ^43^, ^44^ ^]^ Many studies have indicated that F_O_F_1_‐ATP synthase is closely associated with the proliferation, invasion, and metastasis of tumor cells.^[^ ^45^, ^46^, ^47^ ^]^ To verify whether HI‐101 targeted ATP5A and ATP5B, we knocked down the subunits of F_1_ domains separately by shRNA. HI‐101 failed to decrease HIF‐1*α* protein expression only when ATP5A or ATP5B was knocked down, even at a relatively high concentration (30 µm) (Figures S8 and S9, Supporting Information). Given the high sequence identity of ATP5A or ATP5B between humans and pigs with 98.7% and 97.4% respectively, we extracted and purified F_1_‐ATP synthase complex from pig hearts as described by J. E. Walker^[^ ^48^ ^]^ and fluorescence polarization binding assay showed that HI‐103 had a high affinity for F_1_‐ATP synthase complex with a *K*_d_ of 2.6 nm (Figure 3C). Competitive fluorescence polarization binding assay suggested that HI‐101 bound to F_1_‐ATP synthase complex in a similar way as HI‐103 but different from reported F_1_‐ATP synthase inhibitors Oligomycin and Aurovertin B (Figure S10, Supporting Information). Taken together, all the above experiments demonstrated that HI compounds bound ATP5A/ATP5B to inhibit HIF‐1*α* transcriptional activity.

### 2.5 HI‐101 Inhibits HIF‐1*α* Translation

**Figure 4:**
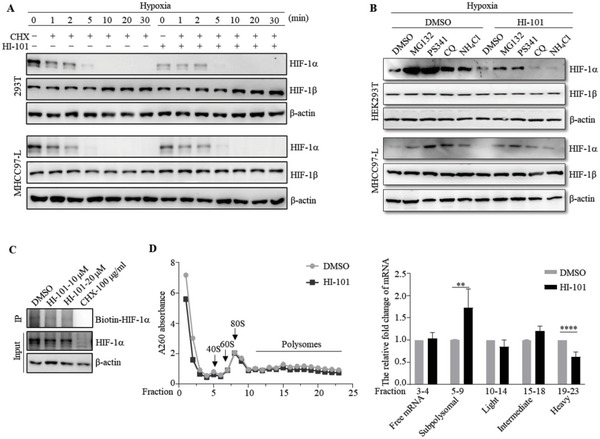
HI‐101 decreased HIF‐1*α* protein level by inhibiting its translational process. A) Protein quantification of HIF‐1*α* and HIF‐1*β* with the treatment of cycloheximide (CHX, 100 µg mL^−1^) and HI‐101 (10 µm) or CHX only under hypoxic conditions over a period of 30 min. B) Protein quantification of HIF‐1*α* and HIF‐1*β* with the treatment of HI‐101 (10 µm) or DMSO. Accumulations of HIF‐1*α* were induced by protein degradation inhibitors (10 µm MG132, 10 µm PS342, 50 µm CQ and 10 mm NH_4_Cl) under hypoxia condition. C) Quantification of the nascent HIF‐1*α* protein labeled with AHA in HEK293T cells after treatment with HI‐101 or CHX. D) Quantitative analysis of ribosome‐mRNA complexes at different translation stages.

HI‐101 treatment exhibited a significant reduction in HIF‐1*α* protein level in a dose‐dependent manner which indicated HI‐101 either enhanced the degradation of mature HIF‐1*α* protein or impaired the synthesis of nascent HIF‐1*α* protein. We first examined whether the degradation rate of HIF‐1*α* protein was enhanced by HI‐101. We used cycloheximide (CHX) to block protein synthesis and determined the HIF‐1*α* protein decay curve by collecting and analyzing protein lysates at different time points after CHX treatment. Cells were pre‐incubated under hypoxia for 6–8 h to achieve a steady HIF‐1*α* protein level. Then, the cells were co‐incubated with 100 µg mL^−1^ CHX as well as 10 µm HI‐101 or DMSO at the indicated time. The half‐life of HIF‐1*α* was examined by western blot analysis. As shown in **Figure** **4**A, HI‐101 decreased HIF‐1*α* protein level while did not have a significant effect on the rate of HIF‐1*α* protein degradation. Therefore, we speculated that HI‐101 might reduce the synthesis of nascent HIF‐1*α* protein.

Lysosome and ubiquitin/proteasome systems are two main pathways for cellular protein degradation.^[^ ^49^, ^50^ ^]^ MG‐132 and PS‐341 are potent, selective, and reversible proteasome inhibitors, chloroquine (CQ) and NH_4_Cl are autophagy inhibitors that inhibit lysosomal degradation by increasing lysosomal pH. MG‐132, PS‐341, CQ, NH_4_Cl, and DMSO at indicated concentrations were added to HEK293T cells for 6 h followed by treatment with DMSO or 10 µm HI‐101 for another 12 h under hypoxia condition. Cells were then collected, lysed, and immunoblotted to detect HIF‐1*α* protein level. The results showed that HIF‐1*α* protein level was decreased with HI‐101 treatment regardless of whether protein degradation inhibitors were present or not, while the control proteins level (HIF‐1*β* and *β*‐actin) were unaffected (Figure 4B), which suggested us that HI‐101 decreased HIF‐1*α* protein level might by inhibiting the production of nascent HIF‐1*α* protein. In order to verify whether HI‐101 inhibited the synthesis of HIF‐1*α* protein, l‐azidohomoalanine (AHA) labeling assay^[^ ^51^ ^]^ was carried out. The nascent protein was labeled by AHA incorporation, enriched by click reaction, and detected by anti‐biotin antibody. Experimental results showed that HIF‐1*α* protein synthesis was significantly inhibited by HI‐101 (Figure 4C).

The whole protein synthesis process can be divided into four major steps: transcription, translation, posttranslational modifications and folding to a mature protein, and protein secretion.^[^ ^52^ ^]^ We then investigated the potential effects of HI‐101 on HIF‐1*α* transcription and translation. Real time‐qPCR (RT‐qPCR) experiments were performed and demonstrated HI‐101 did not affect HIF‐1*α* and HIF‐1*β* mRNA expression (Figure S11, Supporting Information), while it significantly decreased the mRNA expression of HIF‐1*α* target gene VEGF. On the basis of the existing experimental results, we draw a conclusion that HI‐101 inhibited the synthesis of nascent HIF‐1*α* protein without affecting its transcription.

Given that HI‐101 affected neither the transcription of HIF‐1*α* nor its degradation, we next tested whether HI‐101 regulated HIF‐1*α* translation. Polysome profile analysis is a commonly used method for studying translation process by separating polysomes and ribosomal subunits using a sucrose density gradient (SDG).^[^ ^53^ ^]^ After treating with HI‐101 or DMSO for 12 h co‐incubated with MG‐132 under hypoxia condition, HEK293T cells were collected and polysome‐bound and polysome‐free mRNAs were isolated by using 10–50% sucrose gradient fractionation. As evidenced in Figure 4D, ribosome‐free subunits were upregulated and heavy polysomes were downregulated with HI‐101 treatment, which indicated HI‐101 efficiently inhibited HIF‐1*α* translation. Overall, these results suggested that HI‐101 decreased HIF‐1*α* expression by inhibiting its translation.

### 2.6 HI‐101 Inhibits HIF‐1*α* Translation by Enhancing ATP5B and HIF‐1*α* mRNA Interaction

**Figure 5:**
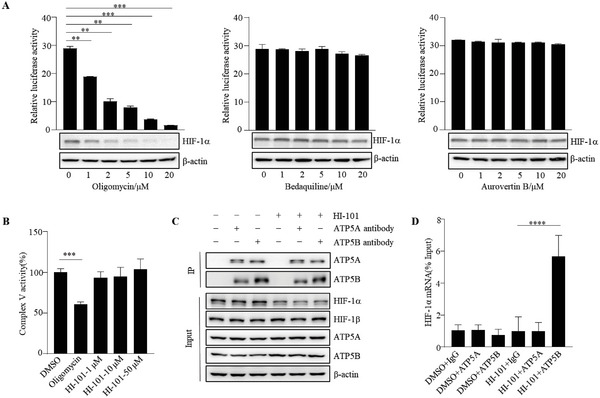
Mechanism study of HI‐101 on inhibiting HIF‐1*α* translation. A) Relative‐Luciferase activity and expression of HIF‐1*α* were examined in HEK293T cells treated with selected ATP synthase inhibitors, including Oligomycin, Bedaquiline, and Aurovertin B at indicated concentrations under hypoxia. B) Detection of the effects of HI‐101 and Oligomycin on the activity of ATP synthase with the MitoTox Complex V OXPHOS activity assay kit. C) Cells treated with DMSO and HI‐101 were lysed and immunoprecipitated with IgG and ATP5A/ATP5B antibodies. Protein quantification of the samples was analyzed by western blot. D) HIF‐1*α* mRNA levels of the same samples in (C) were analyzed by quantitative RT‐PCR analysis.

Although we revealed that HI‐101 bound to ATP5A/ATP5B and decreased HIF‐1*α* protein expression by inhibiting HIF‐1*α* translation, the exact mechanism still remained unclear. It has been reported recently that KUSC‐5037 inhibited HIF‐1*α* transcription by suppressing FoF_1_‐ATP synthase activity.^[^ ^35^ ^]^ Thus, we wondered whether HI‐101 inhibited HIF‐1*α* translation in a similar way. Several ATP synthase inhibitors with various mechanisms of action have been reported.^[^ ^54^ ^]^ Among them, Oligomycin inhibits the ATP synthase activity by binding the F_O_ domain; Bedaquiline, an FDA‐approved drug, inhibits mitochondrial ATP production by targeting the gamma subunit of the ATP synthase;^[^ ^55^ ^]^ Aurovertin B decreases ATP synthase activity by targeting the subunits of F_1_ domain. We first measured HIF‐1*α* transcriptional activity and protein level with the presence of reported ATP synthase inhibitors. Only Oligomycin decreased HIF‐1*α* transcriptional activity and protein level in a dose‐dependent manner (**Figure** **5**A), which indicated that there was a relationship between HIF‐1*α* transcriptional activity and FoF_1_‐ATP synthase activity. However, HI‐101 showed no effect on ATP synthase activity even at a relatively high concentration (50 µm) (Figure 5B), which indicated that HI‐101 inhibited HIF‐1*α* translation in a FoF_1_‐ATP synthase enzyme activity‐independent manner. A recent study reported that ATP5B shut down mPTP by binding circRNA SCAR in an enzymatic‐independent way,^[^ ^56^ ^]^ which reminded us whether ATP5B played a similar role in blocking HIF‐1*α* translation. RNA‐binding protein immunoprecipitation was then performed using the Magna RIP RNA‐Binding Protein Immunoprecipitation Kit (Millipore, Burlington, MA) to detect the interaction between ATP5B and HIF‐1*α* mRNA. As shown in Figure 5C, HI‐101 affected neither the protein expression of ATP5A/ATP5B nor their interaction. However, HI‐101 improved HIF‐1*α* mRNA binding to ATP5B only in the immunoprecipitation sample using anti‐ATP5B antibody, indicating that this interaction was independent of ATP5A/ATP5B complex. To explain why ATP5A knock‐down showed a similar phenotype under HI‐101 treatment, we then checked the protein level of ATP5B in ATP5A knock‐down cell line because both ATP5A and ATP5B are subunits of mitochondrial ATP synthase. The western blot result (Figure S12, Supporting Information) showed that ATP5A knock‐down downregulated the protein level of ATP5B, which attenuated the HIF‐1*α*‐reducing effect of HI‐101 in ATP5A knock‐down cell lines.

In order to better understand the mode of the action of HI compounds with ATP5B, we tried to co‐crystallize HI compounds and F_1_‐ATP synthase. However, we only solved the crystal structure of pig F_1_‐ATP synthase at a resolution of 3.1 Å and failed to obtain the co‐crystal structure, probably due to poor aqueous solubility of HI compounds. Thus, we utilized molecular docking to reveal how HI compounds bind to ATP5B. The results (Figure S16, Supporting Information) showed that HI‐105 bound to ATP5B by forming two key hydrogen bonds with Leu342 and Arg412 in the hinge region. The adamantyl group fully occupied the hydrophobic pocket formed by Pro350, Leu351, and Leu378, demonstrating that modifications on the adamantyl group would lead to clashes and impaired affinity. In addition, the benzene ring extended into the solvent‐exposed region and substituents on the benzene ring were tolerated, which correlated well with structure‐activity relationship. Among them, HI‐105 exhibits excellent inhibitory activity for an extra hydrogen bond with Leu342 via the hydroxyl group. Although the binding mode of HI compounds and ATP5B was proposed, the structure mechanism of how HI compound promotes ATP5B binding to HIF‐1*α* mRNA still remains unclear.

To sum up, the above experimental results showed that HI‐101 inhibited HIF‐1*α* translation by enhancing the interaction of HIF‐1*α* mRNA and ATP5B in an FoF_1_‐ATP synthase activity‐independent manner.

### 2.7 Pharmacokinetic Properties of Compound HI‐104 In Vivo

First, we roughly screened the concentrations in the plasma of seven selected compounds (1‐3n, 1‐3au, 1‐4a, 1‐4k, 1‐4l, 1‐4m, and 1‐4p) at indicated times after intraperitoneal administration in the Institute of Cancer Research (ICR) mice at a dose of 50 mg kg^−1^ with two mice per compound, the blood at five different times was collected and the plasma concentration was analyzed finally. The results (Figure S13, Supporting Information) showed that 1‐3n, 1‐4a, 1‐4k, 1‐4l, 1‐4m, and 1‐4p were barely detectable in the plasma and 1‐3au (HI‐104) had a high concentration in the plasma. Therefore, we further investigated the pharmacokinetic properties of HI‐104 in detail. Compound HI‐104 had a half‐life of 5.66 h, a high maximum concentration (*C*_max_) of 91.68 µg mL^−1^, and an area under the curve (AUC_0‐24 h_) of 860.56 µg h mL^−1^, which showed a very good drug exposure in the blood. Besides, we examined the toxicity of HI‐104 in ICR mice by administering for 1 week at a dose of 100 mg kg^−1^ by intraperitoneal injection. HI‐104 was not overtly toxic, as there was no significant change in body weight and mental status of mice (Figure S14, Supporting Information). The above experiments proved that HI‐104 can be used for further efficiency studies in vivo.

### 2.8 HI‐104 Attenuates Tumor Growth in MHCC97‐L Xenograft Model

**Figure 6:**
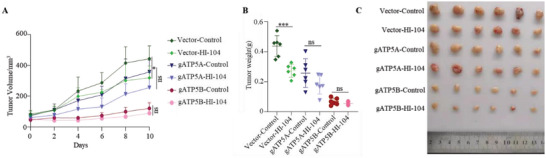
Effects of HI‐104 on multiple MHCC97‐L xenografts tumor growth. Nude mice bearing tumors formed by MHCC97‐L or gATP5A/gATP5B cells were administered with HI‐104. Figures represent the A) average tumor volumes, B) isolated tumor weights, C) tumor images at the end of observation.

HIF‐1*α* overexpression is implicated in human hepatocellular carcinoma (HCC).^[^ ^57^ ^]^ To investigate the biological significance of HI‐104 in the progression of HCC by targeting ATP5A or ATP5B, we performed xenograft models in nude mice with ATP5A or ATP5B stable knock‐out MHCC97‐L cell lines (**Figure** **6**A). The cells were inoculated to 6–8 weeks old nude mice under the armpits, and daily intraperitoneal injection of HI‐104 was started after the tumor volume reached 100 mm^3^. Tumor volume and body weight were monitored during the treatment. Knockout of ATP5B significantly reduced tumor growth in MHCC97‐L xenograft model, which is similar to those previously reported in the literature.^[^ ^58^ ^]^ Besides, we observed that HI‐104 reduced the tumor volume and weight in wildtype MHCC97‐L xenograft tumor model with a tolerant weight loss, while almost had no effect in ATP5B KO models, which indicated that HI‐104 possibly inhibited tumor growth through ATP5B (Figure 6A–C, Figure S15, Supporting Information).

## 3 Conclusion 

In summary, we conducted a high throughput screening based on a dual luciferase‐reporter assay in order to search for novel HIF‐1*α* transcriptional inhibitors. Hit compound HI‐101 with an adamantaniline group was identified, and then probe HI‐102 was developed for target identification. Through a series of experiments, ATP5B was identified as the potential target. Further mechanism study suggested HI‐101 inhibited HIF‐1*α* translational process and decreased protein expression by promoting the binding of ATP5B with HIF‐1*α* mRNA. Besides, extensive structural optimization was performed to improve potency and compound HI‐105 exhibited the most potency in HIF‐1*α* transcriptional activity with an IC_50_ of 26 nm, HI‐104 showed a mild degree of antitumor effect in xenograft mouse model. Taken together, we revealed that adamantaniline derivatives inhibited HIF‐1*α* transcriptional activity by blocking its translational process via ATP5B, which provided a new strategy for antitumor agent development.

## 4 Chemistry

**Scheme 1:**
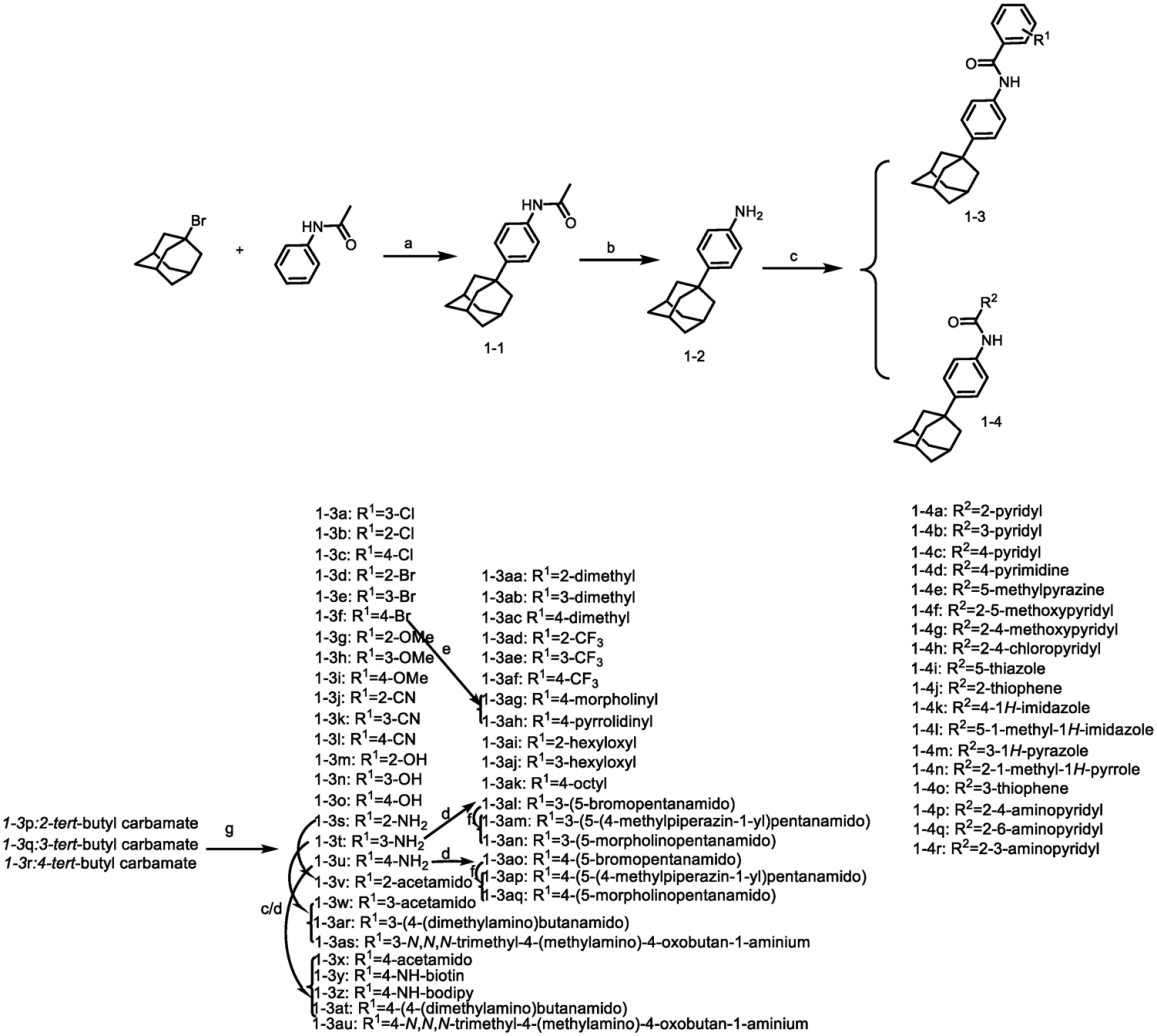
Synthesis routes to class A compounds. Reagents and conditions: a) AlCl_3_, 140 °C, 40–48 h; b) Con HCl, MeOH, reflux,20–30 h; c) HATU, DIPEA, DMF, RT, 5–12 h; d) DCM, triethylamine, RT, overnight; e) Pd_2_(dba)_3_, BINAP, NaOBu‐t, dioxane, 90 °C;(f) K_2_CO_3_, KI, DMF, 80 °C; g) DCM, TFA, RT, 5–12 h.

As shown in **Scheme** **1**, we started from commercially available 1‐bromoadamantane and acetanilide. After Friedel‐Crafts Alkylation, followed by hydrolysis with MeOH and HCl to obtain the key intermediate 1‐2. Derivatives (1‐3a–1‐3r/1‐3aa–1‐3ak/1‐4a–1‐4r) were prepared by acylation of 1‐2 with corresponding acids. Deprotection of Boc group (1‐3p–1‐3r) with TFA gave compounds (1‐3s–1‐3u). Acylation of 1‐3s–1‐3u with corresponding acids or acyl chloride to give final products (1‐3v–1‐3z and 1‐3al–1‐3au).

**Scheme 2:**
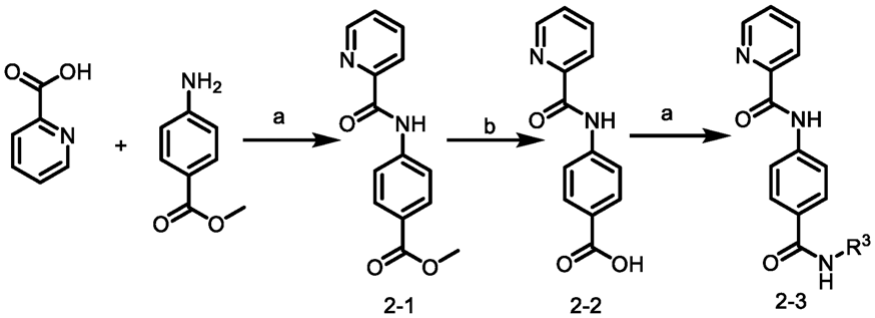
Synthesis routes to class B compounds. Reagents and conditions: a) HATU, DIPEA, DMF, RT, corresponding amino; b) 2 m NaOH, MeOH, 50 °C.

**Scheme 3:**
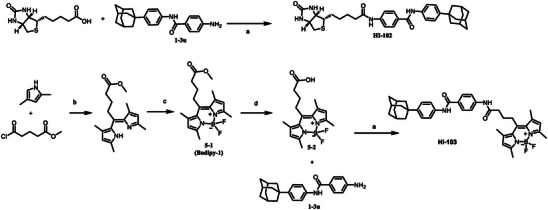
Synthesis routes to probes HI‐102 and HI‐103. Reagents and conditions: a) HATU, DIPEA, DMF, RT, 5–12 h; b) N_2_, 0 °C, 30 min; RT, 3 h; RT—0 °C; c) Et_3_N, 30 min, 0 °C; BF_3_·OEt_2_, 0 °C‐RT; d) LiOH, MeOH, 75 °C.

Class B compounds with a linker between adamantane and benzene ring were synthesized as **Scheme** **2**. As shown in Table 3, since 1‐4a demonstrated particularly good activity against HIF‐1*α* transcriptional activity, we designed and synthesized compounds with the 2‐pyridylderivatives or bioisosteres. 2‐1 was synthesized through amide condensation using 2‐picolinic acid and methyl 4‐aminobenzoate as starting materials, followed by hydrolysis to give intermediates 2‐2. 2‐3a–2‐3e were obtained by a similar procedure with corresponding amines (Scheme 2). Besides, substituents in adamantane phenyl group were introduced into the hit compound and these derivatives (3‐2a–3‐2i) were synthesized following a similar synthetic route as shown in Scheme 1. Besides, Probes HI‐102 and HI‐103 were synthesized as shown in **Scheme** **3**.

## 5 Experimental Section

### Chemistry

All reagents and solvents were purchased commercially and used without further purification. Reaction processes were monitored by thin‐layer chromatography (TLC) and visualized under UV light at 254 and 365 nm, or color reagents. Column chromatography was conducted on silica gel (300–400 mesh) using automatic purification apparatus. ^1^H NMR and ^13^C NMR spectra were recorded on Bruker AC400 and Bruker AC600NMR spectrometer respectively in Chloroform‐*d* or DMSO‐*d_6_* with tetramethylsilane (TMS) as an internal reference. High‐resolution mass spectra were recorded on triple TOF 5600+ MS/MS system (AB Sciex, Concord, Ontario, Canada) in negative or positive ESI mode. The purity of target compounds was determined by high‐performance liquid chromatography with Promosil C18 column from Agela Technologies (4.6 mm × 150 mm, 5 µm particle size). Mobile phase A was double distilled water containing 0.1% trifluoroacetic acid and mobile phase B was methanol containing 0.1% trifluoroacetic acid. Flow rate was 1 mL min^−1^ using linear gradients as follows: 0–1 min was 40%B, 1–5 min was from 40%B to 95%B, 5–7 min was 95%B, 7–8 min was from 95%B to 40%B, 8–10 min was 40%B. All the biologically tested compounds confirmed at least 95% purity.

### General Procedure for the Synthesis of Compounds 1‐3a–1‐3r, 1‐3aa–1‐3af, 1‐3ai–1‐3ak, 1‐3ar–1‐3au,1‐4a–1‐4r

Step1. To a mixture of 1‐bromoadamantane (10.7 g, 0.05 mol) and acetanilide (108 g, 0.80 mol) in 500 mL three‐necked flask equipped with a mechanical stirrer, a nitrogen inlet with a thermometer and a condenser. Anhydrous aluminum chloride (1.33 g, 0.01 mol) as the catalyst was added to the mixture until the reactants were melted at 120 °C. The reaction was stirred vigorously for 36 h at 145 °C. The mixture was poured into ice water containing 4 m hydrochloric acid then purified by precipitation in refluxed water for 5–8 times and recrystallization in toluene ultimately. The precipitants were dried in a vacuum oven.

Step2. The crude product was dissolved in a mixture of methanol and 4 m hydrochloric acid and refluxed for 24 h, anhydrous potassium hydroxide was added slowly into the solution with ice bath, and the suspension was extracted with ethyl acetate. The organic phase was washed with brine, dried over anhydrous Na_2_SO_4_, and filtered. The residue was concentrated in vacuo and purified by silica chromatography to get intermediate 1‐2.

Step3. Corresponding acids (1.2 mmol) and HATU (570 mg, 1.5 mmol) were added to a solution of 5 mL dry DMF and stirred for 30 min, then the 1‐2 (227 mg, 1 mmol) and DIPEA (870 µL, 5 mmol) were added slowly into the reaction mixture respectively and stirred vigorously overnight. The reaction was stopped by adding water and extracted with ethyl acetate, the organic phase was washed with brine twice, dried over anhydrous Na_2_SO_4_, and filtered. The residue was concentrated in vacuo and purified by silica chromatography to get target compounds 1‐3a–1‐3r, 1‐3aa–1‐3af, 1‐3ai–1‐3ak, 1‐4a–1‐4r.

Step4. Trifluoroacetate (382 µL, 5 mmol) was slowly pipetted drop by drop to corresponding aminos (compounds 1‐3p–1‐3r, 0.5 mmol) dissolved in dry dichloromethane under argon atmosphere in an ice bath and the mixture was stirred at rt overnight. Then saturated sodium bicarbonate solution (10–20 mL) was added to the reaction after it was completed followed by extracting with ethyl acetate and then the organic phase was washed with brine twice, dried with saturated anhydrous Na_2_SO_4_, and filtered, then the residue was purified by silica chromatography after removal of ethyl acetate to afford compounds 1‐3s–1‐3u.

### *N*‐(4‐((3r,5r,7r)‐Adamantan‐1‐yl)Phenyl)−3‐Chlorobenzamide(HI‐101, 1‐3a)

It was obtained as a light pink solid in 86% yield. ^1^H NMR (400 MHz, DMSO‐*d*_6_) *δ* 10.30 (s, 1H), 7.99 (s, 1H), 7.91 (d, *J* = 8.0 Hz, 1H), 7.67 (t, *J* = 7.6 Hz, 3H), 7.57 (t, *J* = 8.0 Hz, 1H), 7.34 (d, *J* = 8.4 Hz, 2H), 2.06 (s, 3H), 1.86 (s, 6H), 1.73 (s, 6H). ^13^C NMR (151 MHz, DMSO‐*d*_6_) *δ* 163.71, 146.48, 136.77, 136.11, 133.03, 131.16, 130.26, 127.17, 126.26, 124.66, 120.13, 42.49, 36.02, 35.26, 28.15. MS (ESI) (*m*/*z*): 366.3 (M+H)^+^.HRMS (ESI) calcd for C_23_H_24_ClNO [M+Na]^+^: 388.1439; found: 388.1441. Purity: 100%

### *N*‐(4‐((3r,5r,7r)‐Adamantan‐1‐yl)Phenyl)−2‐Chlorobenzamide(1‐3b)

It was obtained as a white solid in 85% yield.^1^H NMR (400 MHz, DMSO‐*d*_6_) *δ* 10.43 (s, 1H), 7.67–7.59 (m, 2H), 7.58–7.39 (m, 4H), 7.37–7.28 (m, 2H), 2.05 (s, 3H), 1.85 (s, 6H), 1.73 (s, 6H). ^13^C NMR (151 MHz, DMSO‐*d*_6_) *δ* 165.19, 146.96, 137.58, 136.89, 131.46, 130.39, 130.09, 129.36, 127.70, 125.38, 119.88, 43.14, 36.67, 35.88, 28.79. MS (ESI) (*m*/*z*): 366.2 (M+H)^+^. HRMS (ESI) calcd for C_23_H_24_ClNO [M+H]^+^: 366.1619; found: 366.1619. Purity: 99.66%

### *N*‐(4‐((3r,5r,7r)‐Adamantan‐1‐yl)Phenyl)−4‐Chlorobenzamide(1‐3c)

It was obtained as a white solid in 89% yield. ^1^H NMR (400 MHz, DMSO‐*d*_6_) *δ* 10.26 (s, 1H), 7.98 (d, *J* = 8.8 Hz, 2H), 7.68 (d, *J* = 8.8 Hz, 2H), 7.61 (d, *J* = 8.0 Hz, 2H), 7.33 (d, *J* = 8.8 Hz, 2H), 2.06 (s, 3H), 1.86 (s, 6H), 1.74 (s, 6H). ^13^C NMR (151 MHz, DMSO‐*d*_6_) *δ* 164.67, 146.96, 136.89, 134.17, 130.04, 128.90, 125.26, 120.71, 43.14, 36.67, 35.89, 28.80. MS (ESI) (*m*/*z*): 366.23(M+H)^+^. HRMS (ESI) calcd for C_23_H_24_ClNO [M+H]^+^: 366.1619; found: 366.1616. Purity: 98.21%

### *N*‐(4‐((3r,5r,7r)‐Adamantan‐1‐yl)Phenyl)−2‐Bromobenzamide(1‐3d)

It was obtained as a white solid in 79% yield. ^1^H NMR (600 MHz, DMSO‐*d*_6_) *δ* 10.38 (s, 1H), 7.72 (d, *J* = 8.4 Hz, 1H), 7.64 (d, *J* = 8.4 Hz, 2H), 7.54–7.48 (m, 2H), 7.42 (td, *J* = 7.8, 2.4 Hz, 1H), 7.36–7.30 (m, 2H), 2.06 (s, 3H), 1.86 (s, 6H), 1.75 (s, 6H). ^13^C NMR (151 MHz, DMSO‐*d*_6_) *δ* 166.08, 146.94, 139.74, 136.91, 133.16, 131.55, 129.29, 128.17, 125.37, 119.90, 119.48, 43.14, 36.67, 35.88, 28.79. MS (ESI) (*m/z*): 408.3 (M‐H)^−^.HRMS (ESI) calcd for C_23_H_24_BrNO [M‐H]^−^: 408.0968; found: 408.0970. Purity: 98.72%

### *N*‐(4‐((3r,5r,7r)‐Adamantan‐1‐yl)Phenyl)−3‐Bromobenzamide(1‐3e)

It was obtained as a white solid in 87% yield. ^1^H NMR (400 MHz, Chloroform‐*d*) *δ* 7.99 (s, 1H), 7.77 (s, 2H), 7.66 (d, *J* = 7.6 Hz, 1H), 7.59–7.51 (m, 2H), 7.41–7.30 (m, 3H), 2.10 (s, 3H), 1.90 (s, 6H), 1.77 (s, 6H).^13^C NMR (151 MHz, Chloroform‐*d*) *δ* 163.56, 147.62, 136.47, 134.31, 134.08, 129.71, 129.62, 124.97, 122.33, 119.54, 42.57, 36.14, 35.35, 28.31.MS (ESI) (*m/z*): 408.3 (M‐H)^−^.HRMS (ESI) calcd for C_23_H_24_BrNO [M‐H]^−^: 408.0968; found: 408.0971. Purity: 99.03%

### *N*‐(4‐((3r,5r,7r)‐Adamantan‐1‐yl)Phenyl)−4‐Bromobenzamide(1‐3f)

It was obtained as a white solid in 85% yield. ^1^H NMR (400 MHz, DMSO‐*d_6_*) *δ* 10.26 (s, 1H), 7.96–7.85 (m, 2H), 7.78–7.64 (m, 4H), 7.37–7.29 (m, 2H), 2.05 (s, 3H), 1.85 (s, 6H), 1.73 (s, 6H). ^13^C NMR (151 MHz, DMSO‐*d*_6_) *δ* 164.14, 146.32, 136.25, 133.89, 131.21, 129.60, 125.06, 124.63, 120.05, 42.49, 36.03, 35.25, 28.16.MS (ESI) (*m*/*z*): 408.3 (M‐H)^−^.HRMS (ESI) calcd for C_23_H_24_BrNO [M‐H]^−^: 408.0968; found: 408.0964. Purity: 98.95%

### *N*‐(4‐((3r,5r,7r)‐Adamantan‐1‐yl)Phenyl)−2‐Methoxybenzamide(1‐3g)

It was obtained as a white solid in 83% yield. ^1^H NMR (400 MHz, DMSO‐*d*_6_) *δ* 10.04 (s, 1H), 7.65 (d, *J* = 9.2 Hz, 3H), 7.49 (t, *J* = 8.0 Hz, 1H), 7.30 (d, *J* = 8.4 Hz, 2H), 7.17 (d, *J* = 8.8 Hz, 1H), 7.06 (t, *J* = 7.6 Hz, 1H), 3.89 (s, 3H), 2.05 (s, 3H), 1.85 (s, 6H), 1.73 (s, 6H). ^13^C NMR (151 MHz, DMSO‐*d*_6_) *δ* 164.65, 156.97, 146.64, 137.01, 132.44, 130.18, 125.29, 120.96, 120.01, 112.48, 56.37, 43.16, 36.68, 35.85, 28.81. MS (ESI) (*m/z*): 362.2(M+H)^+^. HRMS (ESI) calcd for C_24_H_27_NO_2_ [M+H]^+^: 362.2115; found: 362.2116. Purity: 99.27%

### *N*‐(4‐((3r,5r,7r)‐Adamantan‐1‐yl)Phenyl)−3‐Methoxybenzamide(1‐3h)

It was obtained as a white solid in 86% yield. ^1^H NMR (400 MHz, DMSO‐*d*_6_) *δ* 10.14 (d, *J* = 9.2 Hz, 1H), 7.74–7.62 (m, 2H), 7.57–7.37 (m, 3H), 7.35–7.26 (m, 2H), 7.20–7.09 (m, 1H), 3.88–3.75 (m, 3H), 2.05 (s, 3H), 1.84 (d, *J* = 8.4 Hz, 6H), 1.72 (d, *J* = 10.4 Hz, 6H). ^13^C NMR (151 MHz, DMSO‐*d*_6_) *δ* 165.49, 159.65, 146.82, 137.03, 129.98, 125.20, 120.72, 120.29, 117.67, 113.34, 55.80, 43.15, 36.68, 35.88, 28.81. MS (ESI) (*m/z*): 362.2 (M+H)^+^. HRMS (ESI) calcd for C_24_H_27_NO_2_ [M+H]^+^: 362.2115; found: 362.2116. Purity: 99.16%

### *N*‐(4‐((3r,5r,7r)‐Adamantan‐1‐yl)Phenyl)−4‐Methoxybenzamide(1‐3i)

It was obtained as a white solid in 86% yield. ^1^H NMR (400 MHz, DMSO‐*d*_6_) *δ* 10.04 (s, 1H), 7.96 (d, *J* = 8.4 Hz, 2H), 7.69 (d, *J* = 8.0 Hz, 2H), 7.32 (d, *J* = 8.0 Hz, 2H), 7.06 (d, *J* = 8.4 Hz, 2H), 3.84 (s, 3H), 2.06 (s, 3H), 1.86 (s, 6H), 1.74 (s, 6H). ^13^C NMR (151 MHz, DMSO‐*d*_6_) *δ* 165.15, 162.28, 146.51, 137.28, 129.99, 127.53, 125.14, 120.63, 114.02, 55.88, 43.17, 36.68, 35.85, 28.82. MS (ESI) (*m/z*): 362.27 (M+H)^+^. HRMS (ESI) calcd for C_24_H_27_NO_2_ [M+H]^+^: 362.2115; found: 362.2112. Purity: 99.07%

### *N*‐(4‐((3r,5r,7r)‐Adamantan‐1‐yl)Phenyl)−2‐Cyanobenzamide(1‐3j)

It was obtained as a yellow solid in 83% yield. ^1^H NMR (600 MHz, DMSO‐*d*_6_) *δ* 8.00–7.95 (m, 2H), 7.94–7.89 (m, 2H), 7.51 (d, *J* = 8.4 Hz, 2H), 7.38 (d, *J* = 8.4 Hz, 2H), 2.09 (s, 3H), 1.92 (s, 6H), 1.77 (s, 6H).^13^C NMR (151 MHz, DMSO‐*d*_6_) *δ* 167.63, 151.22, 135.16, 132.04, 129.77, 127.48, 125.70, 123.87, 42.99, 36.60, 36.29, 28.77. MS (ESI) (*m/z*): 357.20 (M+H)^+^. HRMS (ESI) calcd for C_24_H_24_N_2_O [M+H]^+^: 357.1961; found: 357.1958. Purity: 99.42%

### *N*‐(4‐((3r,5r,7r)‐Adamantan‐1‐yl)Phenyl)−3‐Cyanobenzamide(1‐3k)

It was obtained as a white solid in 85% yield. ^1^H NMR (400 MHz, DMSO‐*d*_6_) *δ* 10.42–10.31 (m, 1H), 8.46–8.36 (m, 1H), 8.24 (t, *J* = 7.6 Hz, 1H), 8.06 (t, *J* = 7.2 Hz, 1H), 7.80–7.63 (m, 3H), 7.42–7.29 (m, 2H), 2.04 (s, 3H), 1.85 (s, 6H), 1.73 (s, 6H). ^13^C NMR (151 MHz, DMSO‐*d*_6_) *δ* 163.85, 147.19, 136.70, 136.45, 135.36, 132.95, 131.68, 130.29, 125.33, 120.67, 118.82, 111.98, 43.13, 36.66, 35.92, 28.80. MS (ESI) (*m/z*): 357.26 (M+H)^+^ 355.40 (M‐H)^−^. HRMS (ESI) calcd for C_24_H_24_N_2_O [M+H]^+^: 357.1961; found: 357.1959. Purity: 99.00%

### *N*‐(4‐((3r,5r,7r)‐Adamantan‐1‐yl)Phenyl)−4‐Cyanobenzamide(1‐3l)

It was obtained as a white solid in 88% yield. ^1^H NMR (400 MHz, DMSO‐*d*_6_) *δ* 10.42 (s, 1H), 8.10 (d, *J* = 8.0 Hz, 2H), 8.02 (d, *J* = 7.6 Hz, 2H), 7.69 (d, *J* = 8.4 Hz, 2H), 7.34 (d, *J* = 8.4 Hz, 2H), 2.04 (s, 3H), 1.85 (s, 6H), 1.73 (s, 6H). ^13^C NMR (151 MHz, DMSO‐*d*_6_) *δ* 164.36, 147.24, 139.50, 136.68, 132.92, 128.96, 125.32, 120.73, 118.81, 114.23, 43.13, 36.66, 35.92, 28.79. MS (ESI) (*m/z*): 357.26 (M+H)^+^355.41 (M‐H)^−^. HRMS (ESI) calcd for C_24_H_24_N_2_O [M+H]^+^: 357.1961; found: 357.1959. Purity: 96.53%

### *N*‐(4‐((3r,5r,7r)‐Adamantan‐1‐yl)Phenyl)−2‐Hydroxybenzamide(1‐3m)

It was obtained as a white solid in 28% yield. ^1^H NMR (400 MHz, DMSO‐*d*_6_) *δ* 11.96 (s, 1H), 10.36 (s, 1H), 7.99 (d, *J* = 8.0 Hz, 1H), 7.63 (d, *J* = 8.8 Hz, 2H), 7.50–7.31 (m, 3H), 6.98 (d, *J* = 8.8 Hz, 2H), 2.06 (s, 3H), 1.88 (d, *J* = 10.4 Hz, 6H), 1.75 (d, *J* = 10.4 Hz, 6H). ^13^C NMR (151 MHz, DMSO‐*d*_6_) *δ* 167.08, 159.24, 147.40, 136.00, 134.15, 129.36, 125.37, 121.34, 119.44, 117.73, 43.12, 36.66, 35.92, 28.80. MS (ESI) (*m/z*): 348.31(M+H)^+^. HRMS (ESI) calcd for C_23_H_25_NO_2_ [M+H]^+^: 348.1958; found: 348.1957. Purity: 97.05%

### *N*‐(4‐((3r,5r,7r)‐Adamantan‐1‐yl)Phenyl)−3‐Hydroxybenzamide(1‐3n, HI‐105)

It was obtained as a white solid in 45% yield. ^1^H NMR (400 MHz, DMSO‐*d*_6_) *δ* 10.07 (s, 1H), 9.73 (s, 1H), 7.67 (d, *J* = 8.0 Hz, 2H), 7.32 (d, *J* = 15.2 Hz, 5H), 6.96 (s, 1H), 2.05 (s, 3H), 1.85 (s, 7H), 1.73 (s, 5H). ^13^C NMR (151 MHz, DMSO‐*d*_6_) *δ* 165.83, 157.80, 146.68, 137.15, 129.84, 125.18, 120.61, 118.83, 118.59, 114.96, 43.16, 36.68, 35.86, 28.81.MS (ESI) (*m/z*): 348.3 (M+H)^+^346.3 (M‐H)^−^ HRMS (ESI) calcd for C_23_H_25_NO_2_ [M+H]^+^: 348.1958; found: 348.1961. Purity: 99.47%

### *N*‐(4‐((3r,5r,7r)‐Adamantan‐1‐yl)Phenyl)−4‐Hydroxybenzamide(1‐3o)

It was obtained as a yellow solid in 51% yield. ^1^H NMR (400 MHz, DMSO‐*d*_6_) *δ* 10.09 (s, 1H), 9.91 (s, 1H), 7.90–7.79 (m, 2H), 7.72–7.61 (m, 2H), 7.34–7.26 (m, 2H), 6.90–6.81 (m, 2H), 2.05 (s, 3H), 1.85 (s, 6H), 1.73 (s, 6H). ^13^C NMR (151 MHz, DMSO‐*d*_6_) *δ* 164.87, 160.24, 145.91, 136.57, 129.45, 125.25, 124.52, 120.06, 114.74, 42.51, 38.70, 36.01, 35.18, 28.14.MS (ESI) (*m/z*): 348.3 (M+H)^+^346.3 (M‐H)^−^HRMS (ESI) calcd for C_23_H_25_NO_2_ [M+H]^+^: 348.1958; found: 348.1969. Purity: 99.83%

### *Tert*‐Butyl 2‐(2‐((4‐((3r,5r,7r)‐Adamantan‐1‐yl)Phenyl)Carbamoyl)Phenyl)Carbamate(1‐3p)

It was obtained as a white solid in 62% yield. ^1^H NMR (400 MHz, DMSO‐*d*_6_) *δ* 10.23–10.07 (m, 1H), 9.61–9.46 (m, 1H), 8.12–7.97 (m, 1H), 7.76–7.48 (m, 4H), 7.45–7.22 (m, 3H), 2.05 (s, 3H), 1.94–1.83 (m, 6H), 1.80–1.65 (m, 6H), 1.57–1.37 (m, 9H). ^13^C NMR (151 MHz, DMSO‐*d*_6_) *δ* 166.03, 153.28, 146.73, 140.17, 137.12, 136.38, 129.03, 125.21, 121.44, 120.59, 118.08, 79.75, 43.15, 36.68, 35.87, 28.80, 28.59.MS (ESI) (*m*/*z*): 447.2 (M+H)^+^445.4(M‐H)^−^.HRMS (ESI) calcd for C_28_H_35_N_3_O_3_S [M+H]^+^: 447.2642; found: 447.2643. Purity: 100%

### *Tert*‐Butyl (3‐((4‐((3r,5r,7r)‐Adamantan‐1‐yl)Phenyl)Carbamoyl)Phenyl)Carbamate(1‐3q)

It was obtained as a white solid in 68% yield.^1^H NMR (400 MHz, DMSO‐*d_6_*) *δ* 10.00 (s, 1H), 9.40 (s, 1H), 7.87 (s, 1H), 7.52 (d, *J* = 8.4 Hz, 2H), 7.45 (d, *J* = 8.4 Hz, 1H), 7.37 (d, *J* = 7.8 Hz, 1H), 7.24 (t, *J* = 8.0 Hz, 1H), 7.17 (d, *J* = 8.4 Hz, 2H), 1.90 (s, 3H), 1.71 (s, 6H), 1.58 (s, 6H), 1.34 (s, 9H). ^13^C NMR (151 MHz, DMSO‐*d*_6_) *δ* 166.03, 153.28, 146.72, 140.18, 137.13, 136.39, 129.02, 125.21, 121.47, 120.59, 118.09, 79.75, 43.15, 36.68, 35.87, 28.81, 28.59.MS (ESI) (*m/z*): 447.2 (M+H)^+^445.4 (M‐H)^−^.HRMS (ESI) calcd for C_28_H_34_N_2_O_3_ [M+Na]^+^: 469.2462; found: 469.2461. Purity: 100%

### *Tert*‐Butyl (4‐((4‐((3r,5r,7r)‐Adamantan‐1‐yl)Phenyl)Carbamoyl)Phenyl)Carbamate(1‐3r)

It was obtained as a white solid in 64% yield.^1^H NMR (400 MHz, DMSO‐*d*_6_) *δ* 10.02 (s, 1H), 9.72 (s, 1H), 7.89 (d, *J* = 8.4 Hz, 2H), 7.68 (d, *J* = 8.0 Hz, 2H), 7.58 (d, *J* = 8.4 Hz, 2H), 7.31 (d, *J* = 8.0 Hz, 2H), 2.06 (s, 3H), 1.86 (s, 6H), 1.74 (s, 6H), 1.50 (s, 9H). ^13^C NMR (151 MHz, DMSO‐*d*_6_) *δ* 165.18, 153.07, 151.55, 146.54, 143.05, 140.08, 137.23, 128.98, 125.16, 120.64, 117.58, 80.02, 43.16, 36.68, 35.86, 28.81, 28.55.MS (ESI) (*m/z*): 447.4 (M+H)^+^445.4 (M‐H)^−^.HRMS (ESI) calcd for C_28_H_34_N_2_O_3_ [M+H]^+^: 447.2642; found: 447.2641. Purity: 100%

### *N*‐(4‐((3r,5r,7r)‐Adamantan‐1‐yl)Phenyl)−2‐Aminobenzamide(1‐3s)

It was obtained as a white solid in 57% yield with two steps after removing the Boc protecting group of 1‐3p with trifluoroacetic acid.^1^H NMR (400 MHz, Chloroform‐*d*) *δ* 7.73 (s, 1H), 7.53–7.41 (m, 3H), 7.34 (s, 1H), 7.24 (t, *J* = 7.4 Hz, 1H), 6.71 (d, *J* = 8.0 Hz, 2H), 5.48 (s, 2H), 2.10 (s, 3H), 1.90 (s, 6H), 1.82–1.74 (m, 6H). ^13^C NMR (151 MHz, DMSO‐*d*_6_) *δ* 168.15, 150.15, 146.51, 137.16, 132.45, 129.09, 125.10, 120.84, 116.80, 115.85, 115.14, 43.17, 36.69, 35.85, 28.81.MS (ESI) (*m/z*): 347.3 (M+H)^+^.HRMS (ESI) calcd for C_23_H_26_N_2_O [M+H]^+^: 347.2118; found: 347.2117. Purity: 98.38%

### *N*‐(4‐((3r,5r,7r)‐Adamantan‐1‐yl)Phenyl)−3‐Aminobenzamide(1‐3t)

It was obtained as a white solid in 49% yield with two steps with the same procedure as 1‐3s.^1^H NMR (400 MHz, DMSO‐*d_6_*) *δ* 10.01 (d, *J* = 12.8 Hz, 1H), 7.67 (d, *J* = 7.2 Hz, 2H), 7.30 (d, *J* = 7.8 Hz, 2H), 7.19–7.02 (m, 3H), 6.73 (d, *J* = 8.8 Hz, 1H), 5.33 (d, *J* = 12.4 Hz, 2H), 2.05 (s, 3H), 1.87 (d, *J* = 12.8 Hz, 6H), 1.74 (d, *J* = 12.0 Hz, 6H). ^13^C NMR (151 MHz, DMSO‐*d*_6_) *δ* 166.63, 149.22, 146.49, 137.31, 136.51, 129.18, 125.14, 120.52, 117.13, 115.15, 113.43, 43.17, 36.68, 35.85, 28.81.MS (ESI) (*m/z*): 347.3 (M+H)^+^345.4 (M‐H)^−^.HRMS (ESI) calcd for C_23_H_26_N_2_O [M+H]^+^: 347.2118; found: 347.2118. Purity: 99.60%

### *N*‐(4‐((3r,5r,7r)‐Adamantan‐1‐yl)Phenyl)−4‐Aminobenzamide(1‐3u)

It was obtained as a yellow solid in 58% yield with two steps with the same procedure as 1‐3s.^1^H NMR (400 MHz, DMSO‐*d*_6_) *δ* 9.70 (s, 1H), 7.70 (d, *J* = 8.0 Hz, 2H), 7.65 (d, *J* = 8.4 Hz, 2H), 7.27 (d, *J* = 8.0 Hz, 2H), 6.58 (d, *J* = 8.0 Hz, 2H), 5.75 (s, 2H), 2.05 (s, 3H), 1.84 (s, 6H), 1.73 (s, 6H). ^13^C NMR (151 MHz, DMSO‐*d*_6_) *δ* 164.95, 151.86, 145.34, 137.06, 129.10, 124.40, 121.07, 119.80, 112.37, 42.57, 36.07, 35.17, 28.19.MS (ESI) (*m/z*): 347.3 (M+H)^+^.HRMS (ESI) calcd for C_23_H_26_N_2_O [M+H]^+^: 347.2118; found: 347.2120. Purity: 99.53%

### 2‐Acetamido‐*N*‐(4‐((3r,5r,7r)‐Adamantan‐1‐yl)Phenyl)Benzamide(1‐3v)

To a mixture of 1‐3s (70 mg, 0.2 mmol) and Et_3_N (83.2 µL, 0.6 mmol) dissolved in dry DCM (10 mL) in a round‐bottomed flask, acetyl chloride (28.5 µL, 0.4 mmol) was added dropwise to the reaction while swirling slowly. After reagents disappeared, the reaction mixture was extracted with dichloromethane and water, then the organic layer was dried with anhydrous sodium sulfate and concentrated in vacuo, and purified by silica gel column chromatography finally. 1‐3v (64 mg) was obtained as a white solid in 83% yield.^1^H NMR (400 MHz, DMSO‐*d*_6_) *δ* 10.51 (s, 1H), 10.41 (s, 1H), 8.20 (d, *J* = 8.0 Hz, 1H), 7.79 (d, *J* = 7.8 Hz, 1H), 7.69 (d, *J* = 8.0 Hz, 2H), 7.56 (t, *J* = 8.0 Hz, 1H), 7.38 (d, *J* = 8.0 Hz, 2H), 7.27 (t, *J* = 8.0 Hz, 1H), 2.11 (s, 6H), 1.91 (s, 7H), 1.79 (s, 6H). ^13^C NMR (151 MHz, DMSO‐*d*_6_) *δ* 168.70, 167.22, 147.17, 138.31, 136.70, 132.00, 129.09, 125.23, 123.51, 122.10, 121.02, 43.15, 36.67, 35.91, 28.80, 24.90.MS (ESI) (*m/z*): 387.4 (M‐H)^−^.HRMS (ESI) calcd for C_25_H_28_N_2_O_2_ [M‐H]^−^: 387.2078; found: 387.2081. Purity: 98.72%

### 3‐Acetamido‐*N*‐(4‐((3r,5r,7r)‐Adamantan‐1‐yl)Phenyl)Benzamide(1‐3w)

It was obtained as a white solid in 80% yield with the same procedure as 1‐3v.^1^H NMR (400 MHz, DMSO‐*d*_6_) *δ* 10.16 (d, *J* = 13.6 Hz, 2H), 8.06 (s, 1H), 7.82 (d, *J* = 8.0 Hz, 1H), 7.71–7.54 (m, 3H), 7.43 (t, *J* = 8.0 Hz, 1H), 7.32 (d, *J* = 6.8 Hz, 2H), 2.07 (s, 6H), 1.86 (s, 6H), 1.73 (s, 6H). ^13^C NMR (151 MHz, DMSO‐*d*_6_) *δ* 168.99, 165.85, 146.77, 139.91, 137.08, 136.26, 129.13, 125.22, 122.33, 122.30, 120.64, 118.95, 43.15, 36.68, 35.88, 28.80, 24.46.MS (ESI) (*m/z*): 389.3 (M+H)^+^387.4 (M‐H)^−^.HRMS (ESI) calcd for C_25_H_28_N_2_O_2_ [M+Na]^+^: 411.2043; found: 411.2040. Purity: 99.70%

### 4‐Acetamido‐*N*‐(4‐((3r,5r,7r)‐Adamantan‐1‐yl)Phenyl)Benzamide(1‐3x)

It was obtained as a white solid in 86% yield with the same procedure as 1‐3v.^1^H NMR (400 MHz, DMSO‐*d*_6_) *δ* 10.23 (s, 1H), 10.05 (s, 1H), 7.91 (d, *J* = 8.4 Hz, 2H), 7.69 (t, *J* = 10.0 Hz, 4H), 7.32 (d, *J* = 8.4 Hz, 2H), 2.13–2.02 (m, 6H), 1.86 (s, 6H), 1.73 (s, 6H). ^13^C NMR (151 MHz, DMSO‐*d*_6_) *δ* 168.71, 168.60, 166.78, 164.56, 162.16, 150.98, 145.98, 143.18, 142.03, 139.47, 136.59, 134.49, 130.21, 128.36, 124.55, 120.00, 118.02, 117.93, 42.54, 36.05, 35.62, 30.61, 28.18, 23.97.MS (ESI) (*m/z*): 389.3 (M+H)^+^387.4(M‐H)^−^.HRMS (ESI) calcd for C_25_H_28_N_2_O_2_ [M+Na]^+^: 411.2043; found: 411.2040. Purity: 98.41%

### *N*‐(4‐((3r,5r,7r)‐Adamantan‐1‐yl)Phenyl)−4‐(5‐((3aS,4S,6aR)−2‐Oxohexahydro‐1*H*‐Thieno[3,4‐d]Imidazol‐4‐yl)Pentanamido)Benzamide (1‐3y, HI‐102)

It was obtained as a yellow solid in 36% yield. ^1^H NMR (400 MHz, DMSO‐*d_6_*) *δ* 10.56 (s, 1H), 10.18 (s, 1H), 7.97 (d, *J* = 5.6 Hz, 2H), 7.79 (d, *J* = 5.6 Hz, 2H), 7.72 (d, *J* = 5.6 Hz, 2H), 7.32 (d, *J* = 5.6 Hz, 2H), 6.50 (s, 1H), 6.44 (s, 1H), 4.32 (s, 1H), 4.25–4.13 (m, 1H), 3.17 (t, *J* = 4.0 Hz, 2H), 2.90 (d, *J* = 3.2 Hz, 1H), 2.84 (d, *J* = 12.0 Hz, 1H), 2.74 (d, *J* = 3.1 Hz, 1H), 2.60 (d, *J* = 12.6 Hz, 1H), 2.40 (s, 2H), 2.06 (s, 3H), 1.87 (s, 6H), 1.75 (s, 5H), 1.64 (s, 2H), 1.38 (d, *J* = 9.3 Hz, 1H), 1.24 (d, *J* = 2.9 Hz, 1H). ^13^C NMR (151 MHz, DMSO‐*d*_6_) *δ* 171.59, 164.53, 162.56, 162.16, 145.87, 142.20, 136.64, 128.68, 128.33, 124.46, 120.09, 117.97, 60.90, 59.06, 55.22, 48.39, 42.53, 36.06, 35.63, 35.22, 28.17, 28.06, 27.95, 24.87.MS (ESI) (*m*/*z*): 571.5 (M‐H)^−^. HRMS(ESI) calcd for C_33_H_40_N_4_O_3_S [M‐H]^−^: 571.2748; found: 571.2743. Purity: 99.65%

### *N*‐(4‐((3r,5r,7r)‐Adamantan‐1‐yl)Phenyl)−4‐(4‐(5,5‐Difluoro‐1,3,7,9‐Tetramethyl‐5*H*‐4l4,5l4‐Dipyrrolo[1,2‐c:2′,1′‐f][1,3,2]Diazaborinin‐10‐yl)Butanamido)Benzamide(1‐3z, HI‐103)

The bodipy‐COOH was prepared as previously described.^[^ ^59^ ^]^ It was obtained as a fuchsia solid in 35% yield. ^1^H NMR (400 MHz, DMSO‐*d_6_*) *δ* 10.32 (s, 1H), 10.06 (s, 1H), 7.92 (d, *J* = 8.4 Hz, 2H), 7.73 (d, *J* = 8.4 Hz, 2H), 7.68 (d, *J* = 8.4 Hz, 2H), 7.32 (d, *J* = 8.4 Hz, 2H), 6.25 (s, 2H), 5.32 (t, *J* = 4.8 Hz, 1H), 3.03 (t, *J* = 8.8 Hz, 2H), 2.58 (t, *J* = 6.8 Hz, 2H), 2.44 (s, 5H), 2.40 (s, 5H), 2.08–1.82 (m, 14H), 1.73 (s, 5H), 1.45 (s, 1H). ^13^C NMR (151 MHz, DMSO‐*d*_6_) *δ* 171.29, 165.16, 153.69, 146.60, 142.44, 141.44, 137.21, 131.25, 129.75, 129.03, 125.18, 122.24, 120.61, 118.76, 43.16, 36.93, 36.68, 35.86, 28.81, 27.68, 27.58, 16.33, 14.57.MS (ESI) (*m*/*z*): 661.6 (M‐H)^−^. HRMS (ESI) calcd for C_40_H_45_BF_2_N_4_O_2_ [M‐H]^−^: 661.3531; found: 661.3537. Purity: 96.96%

### *N*‐(4‐((3r,5r,7r)‐Adamantan‐1‐yl)Phenyl)−2‐(Dimethylamino)Benzamide(1‐3aa)

It was obtained as a white solid in 73% yield.^1^H NMR (600 MHz, DMSO‐*d*_6_) *δ* 11.18 (s, 1H), 7.71–7.62 (m, 3H), 7.45 (t, *J* = 7.8 Hz, 1H), 7.32 (d, *J* = 9.0 Hz, 2H), 7.23 (d, *J* = 7.8 Hz, 1H), 7.09 (t, *J* = 7.8 Hz, 1H), 2.78 (s, 6H), 2.06 (s, 3H), 1.86 (s, 6H), 1.74 (s, 6H). ^13^C NMR (151 MHz, DMSO‐*d*_6_) *δ* 166.01, 151.64, 146.55, 137.13, 131.87, 130.37, 128.15, 125.39, 122.16, 119.89, 119.27, 44.33, 43.16, 36.68, 35.85, 28.81. MS (ESI) (*m/z*): 375.4 (M+H)^+^.HRMS (ESI) calcd for C_25_H_30_N_2_O [M+H]^+^: 375.2431; found: 375.2428. Purity: 100%

### *N*‐(4‐((3r,5r,7r)‐Adamantan‐1‐yl)Phenyl)−3‐(Dimethylamino)Benzamide(1‐3ab)

It was obtained as a white solid in 73% yield.^1^H NMR (400 MHz, DMSO‐*d*_6_) *δ* 10.06 (s, 1H), 7.68 (d, *J* = 5.2 Hz, 2H), 7.31 (t, *J* = 8.4, 3.2 Hz, 3H), 7.21 (s, 2H), 6.91 (d, *J* = 7.8 Hz, 1H), 2.96 (d, *J* = 3.2 Hz, 6H), 2.05 (s, 3H), 1.85 (s, 6H), 1.79–1.66 (m, 6H). ^13^C NMR (151 MHz, DMSO‐*d*_6_) *δ* 166.47, 150.77, 146.63, 137.21, 136.27, 129.32, 125.15, 120.70, 115.63, 111.67, 43.17, 36.68, 35.87, 28.81.MS (ESI) (*m/z*): 375.4 (M+H)^+^.HRMS (ESI) calcd for C_25_H_30_N_2_O [M+H]^+^: 375.2431; found: 375.2424. Purity: 99.20%

### *N*‐(4‐((3r,5r,7r)‐Adamantan‐1‐yl)Phenyl)−4‐(Dimethylamino)Benzamide(1‐3ac)

It was obtained as a white solid in 86% yield. ^1^H NMR (400 MHz, DMSO‐*d*_6_) *δ* 9.79 (s, 1H), 7.86 (d, *J* = 8.4 Hz, 2H), 7.67 (d, *J* = 8.0 Hz, 2H), 7.29 (d, *J* = 8.4 Hz, 2H), 6.75 (d, *J* = 8.4 Hz, 2H), 2.99 (s, 6H), 2.05 (s, 3H), 1.85 (s, 6H), 1.73 (s, 6H). ^13^C NMR (151 MHz, DMSO‐*d*_6_) *δ* 165.04, 152.19, 145.63, 136.80, 128.89, 124.46, 120.84, 119.99, 110.60, 42.53, 36.02, 35.16, 28.15.MS (ESI) (*m/z*): 375.3 (M+H)^+^.HRMS (ESI) calcd for C_25_H_30_N_2_O [M+H]^+^: 375.2431; found: 375.2427. Purity: 99.92%

### *N*‐(4‐((3r,5r,7r)‐Adamantan‐1‐yl)Phenyl)−2‐(Trifluoromethyl)Benzamide(1‐3ad)

It was obtained as a white solid in 75% yield.^1^H NMR (600 MHz, DMSO‐*d*_6_) *δ* 10.46 (s, 1H), 7.85 (d, *J* = 7.8 Hz, 1H), 7.79 (t, *J* = 7.8 Hz, 1H), 7.71 (t, *J* = 7.8 Hz, 1H), 7.67 (d, *J* = 7.2 Hz, 1H), 7.61 (d, *J* = 9.0 Hz, 2H), 7.33 (d, *J* = 9.0 Hz, 2H), 2.06 (s, 3H), 1.86 (s, 6H), 1.75 (s, 6H). ^13^C NMR (151 MHz, DMSO‐*d_6_*) *δ* 164.23, 147.14, 136.74, 136.31, 132.24, 130.15, 129.65 (q, *J* = 32.0 Hz), 128.49 (q, *J* = 3.7 Hz), 125.27, 124.66 (q, *J* = 3.9 Hz), 120.82, 43.13, 36.66, 35.90, 28.81. MS (ESI) (*m/z*): 400.26 (M+H)^+^398.42 (M‐H)^−^. HRMS (ESI) calcd for C_24_H_24_F_3_NO [M+H]^+^: 400.1883; found: 400.1880.Purity: 99.25%

### *N*‐(4‐((3r,5r,7r)‐Adamantan‐1‐yl)Phenyl)−3‐(Trifluoromethyl)Nenzamide(1‐3ae)

It was obtained as a white solid in 86% yield.^1^H NMR (600 MHz, DMSO‐*d*_6_) *δ* 10.40 (s, 1H), 8.31–8.25 (m, 2H), 7.96 (d, *J* = 7.8 Hz, 1H), 7.78 (t, *J* = 7.8 Hz, 1H), 7.71 (d, *J* = 6.6 Hz, 2H), 7.35 (d, *J* = 8.4 Hz, 2H), 2.05 (s, 3H), 1.86 (s, 6H), 1.74 (d, *J* = 3.7 Hz, 6H). ^13^C NMR (151 MHz, DMSO‐*d*_6_) *δ* 165.85, 147.02, 133.05, 130.40, 128.97, 126.76 (q, *J* = 4.8 Hz), 126.28 (q, *J* = 31.3 Hz), 125.39, 119.96, 43.14, 36.67, 35.88, 28.79. MS (ESI) (*m/z*): 400.25 (M+H)^+^398.42 (M‐H)^−^. HRMS (ESI) calcd for C_24_H_24_F_3_NO [M+H]^+^: 400.1883; found: 400.1879. Purity: 98.30%

### *N*‐(4‐((3r,5r,7r)‐Adamantan‐1‐yl)Phenyl)−4‐(Trifluoromethyl)Benzamide(1‐3af)

It was obtained as a white solid in 83% yield.^1^H NMR (600 MHz, DMSO‐*d*_6_) *δ* 10.40 (s, 1H), 8.14 (s, 2H), 7.91 (s, 2H), 7.71 (s, 2H), 7.35 (s, 2H), 2.06 (s, 3H), 1.87 (s, 6H), 1.74 (s, 6H). ^13^C NMR (151 MHz, DMSO‐*d*_6_) *δ* 164.61, 147.16, 139.30, 136.77, 131.75 (d, *J* = 31.8 Hz), 129.01, 125.83 (q, *J* = 3.9 Hz), 125.30, 120.73, 43.14, 36.66, 35.91, 28.80. MS (ESI) (*m/z*): 400.25 (M+H)^+^398.42 (M‐H)^−^. HRMS (ESI) calcd for C_24_H_24_F_3_NO [M+H]^+^: 400.1883; found: 400.1880. Purity: 100%

### *N*‐(4‐((3r,5r,7r)‐Adamantan‐1‐yl)Phenyl)−4‐Morpholinobenzamide(1‐3ag)

Compound 1‐3f (81 mg, 0.2 mmol), *tris*(dibenzylideneacetone)dipalladium (Pd_2_ (dba)_3_, 20 mg, 0.02 mmol), 2,20‐bis(diphenylphosphino)−1,10‐binaphthyl (BINAP, 20 mg, 0.03 mmol), sodium *tert*‐butoxide (NaOBu‐t, 96 mg, 1 mmol) were added to corresponding aminos (1 mmol) under argon atmosphere and the mixture was stirred at 85 °C overnight. Then it was cooled to room temperature and added to 10% aqueous HCl (20 mL). The suspension was extracted with ethyl acetate and the organic phase was washed with brine, dried over anhydrous Na_2_SO_4_, and filtered. The residue was purified by silica chromatography after removal of ethyl acetate to afford compounds 1‐3ag and 1‐3ah. It was obtained as a yellow solid (43 mg) in 53% yield.^1^H NMR (400 MHz, DMSO‐*d*_6_) *δ* 9.91 (s, 1H), 7.90 (s, 2H), 7.70 (s, 2H), 7.32 (s, 2H), 7.04 (s, 2H), 3.77 (s, 4H), 3.27 (s, 4H), 2.07 (s, 3H), 1.92 (s, 6H), 1.76 (s, 6H). ^13^C NMR (151 MHz, DMSO‐*d*_6_) *δ* 152.96, 136.82, 128.81, 124.46, 124.09, 119.91, 113.19, 65.76, 47.16, 42.55, 36.06, 35.20, 28.18. MS (ESI) (*m/z*): 417.4 (M+H)^+^.HRMS (ESI) ca*l*cd for C_27_H_32_N_2_O_2_ [M+H]^+^: 417.2537; found: 417.2534. Purity: 96.44%

### *N*‐(4‐((3r,5r,7r)‐Adamantan‐1‐yl)Phenyl)−4‐(Pyrrolidin‐1‐yl)Benzamide(1‐3ah)

It was obtained as an orange solid in 58% yield with the same procedure as 1‐3ag. ^1^H NMR (400 MHz, DMSO‐*d*_6_) *δ* 9.77 (s, 1H), 7.85 (d, *J* = 5.6 Hz, 2H), 7.67 (d, *J* = 5.6 Hz, 2H), 7.28 (d, *J* = 5.2 Hz, 2H), 6.58 (d, *J* = 5.6 Hz, 2H), 3.36 (d, *J* = 3.2 Hz, 6H), 2.05 (s, 3H), 1.98 (s, 2H), 1.85 (s, 6H), 1.73 (s, 6H). ^13^C NMR (151 MHz, DMSO‐*d*_6_) *δ* 164.90, 149.55, 145.36, 137.05, 129.02, 124.40, 120.35, 119.83, 110.42, 47.09, 42.57, 36.07, 35.18, 28.19, 24.80. MS (ESI) (*m/z*): 401.3 (M+H)^+^399.3 (M‐H)^−^.HRMS (ESI) calcd for C_27_H_32_N_2_O [M+H]^+^: 401.2587; found: 401.2590. Purity: 98.65%

### *N*‐(4‐((3r,5r,7r)‐Adamantan‐1‐yl)Phenyl)−2‐(Hexyloxy)Benzamide(1‐3ai)

It was obtained as a white solid in 61% yield.^1^H NMR (400 MHz, Chloroform‐*d*) *δ* 10.04 (s, 1H), 8.32 (d, *J* = 6.4 Hz, 1H), 7.61 (d, *J* = 7.6 Hz, 2H), 7.46 (d, *J* = 8.0 Hz, 1H), 7.36 (d, *J* = 8.8 Hz, 2H), 7.16–7.07 (m, 1H), 7.01 (d, *J* = 8.0 Hz, 1H), 4.24–4.16 (m, 2H), 2.11 (s, 3H), 2.03–1.90 (m, 8H), 1.78 (s, 6H), 1.63–1.52 (m, 2H), 1.47–1.31 (m, 4H), 0.92 (d, *J* = 6.4 Hz, 3H). ^13^C NMR (151 MHz, Chloroform‐*d*) *δ* 163.46, 157.17, 147.61, 136.37, 133.37, 132.85, 125.73, 122.14, 121.78, 120.26, 112.68, 69.75, 43.57, 37.14, 36.23, 31.88, 29.70, 29.31, 26.24, 22.91, 14.35. MS (ESI) (*m/z*): 432.4 (M+H)^+^. HRMS (ESI) calcd for C_29_H_37_NO_2_ [M+H]^+^: 432.2897; found: 432.2902. Purity: 98.74%

### *N*‐(4‐((3r,5r,7r)‐Adamantan‐1‐yl)Phenyl)−3‐(Hexyloxy)Benzamide(1‐3aj)

It was obtained as a white solid in 54% yield.^1^H NMR (400 MHz, DMSO‐*d*_6_) *δ* 10.13 (s, 1H), 7.68 (d, *J* = 8.4 Hz, 2H), 7.51 (d, *J* = 8.0 Hz, 1H), 7.47 (s, 1H), 7.42 (s, 1H), 7.32 (d, *J* = 8.4 Hz, 2H), 7.13 (d, *J* = 8.0 Hz, 1H), 4.03 (t, *J* = 6.4 Hz, 2H), 2.05 (s, 3H), 1.86 (s, 4H), 1.73 (s, 8H), 1.44 (s, 2H), 1.38–1.06 (m, 6H), 0.93–0.85 (m, 3H). ^13^C NMR (151 MHz, DMSO‐*d*_6_) *δ* 164.87, 158.42, 146.22, 136.34, 129.38, 124.57, 120.11, 119.56, 117.38, 113.24, 67.50, 42.51, 36.03, 35.24, 30.83, 28.45, 28.16, 25.03, 21.91, 13.75. MS (ESI) (*m/z*): 432.4 (M+H)^+^.HRMS (ESI) calcd for C_29_H_37_NO_2_ [M+H]^+^: 432.2897; found: 432.2881. Purity: 99.14%

### *N*‐(4‐((1s,3s)‐Adamantan‐1‐yl)Phenyl)−4‐Octylbenzamide(1‐3ak)

It was obtained as a yellow solid in 46% yield.^1^H NMR (400 MHz, DMSO‐*d*_6_) *δ* 10.10 (s, 1H), 7.86 (d, *J* = 8.0 Hz, 2H), 7.68 (d, *J* = 8.4 Hz, 2H), 7.36–7.30 (m, 4H), 2.64 (t, *J* = 7.6 Hz, 2H), 2.05 (s, 3H), 1.86 (s, 3H), 1.73 (s, 7H), 1.59 (s, 2H), 1.26 (d, *J* = 16.0 Hz, 10H), 0.87–0.85 (m, 2H). ^13^C NMR (151 MHz, DMSO‐*d*_6_) *δ* 165.11, 146.08, 136.48, 132.22, 128.08, 127.47, 124.56, 120.02, 42.52, 36.03, 35.22, 34.78, 31.08, 30.56, 28.62, 28.48, 28.42, 28.16, 21.91, 13.78. MS (ESI) (*m/z*): 444.5 (M+H)^+^.HRMS (ESI) calcd for C_31_H_41_NO [M+H]^+^: 444.3261; found: 444.3259. Purity: 99.75%

### *N*‐(4‐((1s,3s)‐Adamantan‐1‐yl)Phenyl)−3‐(5‐Bromopentanamido)Benzamide(1‐3al)

It was obtained as a white solid in 65% yield with the same procedure as 1‐3v. ^1^H NMR (400 MHz, DMSO‐*d*_6_) *δ* 10.18 (s, 1H), 10.12 (s, 1H), 8.07 (s, 1H), 7.83 (d, *J* = 8.0 Hz, 1H), 7.67 (d, *J* = 8.4 Hz, 2H), 7.60 (d, *J* = 8.0 Hz, 1H), 7.44 (t, *J* = 8.0 Hz, 1H), 7.32 (d, *J* = 8.0 Hz, 2H), 3.57 (t, *J* = 6.4 Hz, 2H), 2.37 (t, *J* = 7.2 Hz, 2H), 2.05 (s, 3H), 1.85 (s, 8H), 1.73 (s, 8H). ^13^C NMR (151 MHz, DMSO‐*d*_6_) *δ* 171.56, 165.85, 146.77, 139.85, 137.09, 136.28, 129.15, 125.23, 122.38, 120.65, 119.02, 43.16, 36.68, 35.88, 35.79, 35.25, 32.22, 28.80, 24.17.MS (ESI) (*m/z*): 509.3 (M+H)^+^507.5 (M‐H)^−^.HRMS (ESI) calcd for C_28_H_33_BrN_2_O_2_ [M+Na]^+^: 531.1618; found: 531.1617. Purity: 98.81%

### *N*‐(4‐((1s,3s)‐Adamantan‐1‐yl)Phenyl)−3‐(5‐(4‐Methylpiperazin‐1‐yl)Pentanamido)Benzamide(1‐3am)

Compound 1‐3ak (51 mg, 0.1 mmol), K_2_CO_3_ (69 mg, 0.5 mmol), KI (1.5 mg, 0.01 mmol) and corresponding aminos (0.3 mmol) were added to dry DMF (≈1–2 mL) and the mixture was stirred at 85 °C overnight. Then it was cooled to room temperature and added to saturated sodium chloride (20 mL). The suspension was extracted with ethyl acetate and the organic phase was washed with brine, dried over anhydrous Na_2_SO_4_, and filtered. The residue was purified by silica chromatography after removal of ethyl acetate to afford target compounds.

It was obtained as a white solid in 82% yield.^1^H NMR (400 MHz, DMSO‐*d*_6_) *δ* 10.16 (d, *J* = 13.6 Hz, 2H), 8.02 (d, *J* = 8.0 Hz, 1H), 7.76 (d, *J* = 8.5 Hz, 1H), 7.60 (d, *J* = 8.4 Hz, 2H), 7.52 (d, *J* = 8.0 Hz, 1H), 7.34 (t, *J* = 8.0 Hz, 1H), 7.24 (d, *J* = 8.4 Hz, 2H), 2.42 (s, 8H), 2.27 (d, *J* = 8.0 Hz, 5H), 2.15 (d, *J* = 7.8 Hz, 3H), 1.97 (s, 3H), 1.77 (s, 6H), 1.65 (s, 6H), 1.51 (q, *J* = 7.8 Hz, 1H), 1.44–1.33 (m, 1H), 1.15 (s, 1H). ^13^C NMR (151 MHz, DMSO‐*d*_6_) *δ* 171.57, 165.36, 146.34, 139.10, 136.23, 135.47, 128.58, 124.61, 121.93, 121.84, 120.18, 118.40, 56.86, 53.61, 51.52, 44.64, 42.49, 35.99, 35.92, 35.21, 28.12, 25.21, 22.75.MS (ESI) (*m/z*): 529.5 (M+H)^+^527.6(M‐H)^−^.HRMS (ESI) calcd for C_33_H_44_N_4_O_2_ [M+H]^+^: 529.3537; found: 529.3539. Purity: 97.75%

### *N*‐(4‐((1s,3s)‐Adamantan‐1‐yl)Phenyl)−3‐(5‐Morpholinopentanamido)Benzamide(1‐3an)

It was obtained as a white solid in 79% yield with the same procedure as 1‐3am.^1^H NMR (400 MHz, DMSO‐*d*_6_) *δ* 10.22 (d, *J* = 13.2 Hz, 1H), 10.13 (d, *J* = 12.8 Hz, 1H), 8.11 (d, *J* = 13.2 Hz, 1H), 7.86 (t, *J* = 9.6 Hz, 1H), 7.75–7.57 (m, 3H), 7.50–7.40 (m, 1H), 7.40–7.29 (m, 2H), 3.59 (d, *J* = 12.0 Hz, 4H), 2.55 (s, 2H), 2.43–2.28 (m, 6H), 2.09 (d, *J* = 14.4 Hz, 3H), 1.89 (d, *J* = 13.6 Hz, 5H), 1.77 (d, *J* = 14.0 Hz, 5H), 1.68–1.59 (m, 2H), 1.49 (s, 2H), 1.33–1.22 (m, 4H). ^13^C NMR (151 MHz, DMSO‐*d*_6_) *δ* 171.56, 165.35, 146.35, 139.09, 136.22, 135.50, 128.61, 124.63, 121.82, 120.15, 118.38, 65.85, 57.68, 53.04, 42.49, 35.99, 35.22, 28.12, 25.12, 22.79.MS (ESI) (*m/z*): 516.4 (M+H)^+^514.6 (M‐H)^−^.HRMS (ESI) calcd for C_32_H_41_N_3_O_3_ [M+H]^+^: 516.3221; found: 516.3223. Purity: 99.85%

### *N*‐(4‐((3r,5r,7r)‐Adamantan‐1‐yl)Phenyl)−4‐(5‐Bromopentanamido)Benzamide(1‐3ao)

It was obtained as a white solid in 70% yield with the same procedure as 1‐3v.^1^H NMR (400 MHz, DMSO‐*d*_6_) *δ* 10.21 (s, 1H), 10.05 (s, 1H), 7.92 (d, *J* = 8.0 Hz, 2H), 7.70 (dd, *J* = 16.8, 8.4 Hz, 4H), 7.31 (d, *J* = 8.0 Hz, 2H), 3.57 (t, *J* = 6.4 Hz, 2H), 2.39 (t, *J* = 7.3 Hz, 2H), 2.05 (s, 3H), 1.91–1.81 (m, 8H), 1.78–1.67 (m, 8H). ^13^C NMR (151 MHz, DMSO‐*d*_6_) *δ* 171.14, 164.49, 145.94, 141.96, 136.58, 128.91, 128.37, 124.55, 119.98, 117.99, 42.52, 36.04, 35.21, 34.63, 31.57, 28.17, 23.43. MS (ESI) (*m/z*): 509.2 (M+H)^+^.HRMS (ESI) calcd for C_28_H_33_BrN_2_O_2_ [M+H]^+^: 509.1798; found: 509.1796. Purity: 98.31%

### *N*‐(4‐((3r,5r,7r)‐Adamantan‐1‐yl)Phenyl)−4‐(5‐(4‐Methylpiperazin‐1‐yl)Pentanamido)Benzamide(1‐3ap)

It was obtained as a white solid in 83% yield with the same procedure as 1‐3am.^1^H NMR (400 MHz, DMSO‐*d*_6_) *δ* 10.42 (s, 1H), 10.11 (s, 1H), 7.94 (d, *J* = 8.4 Hz, 2H), 7.80–7.67 (m, 4H), 7.31 (d, *J* = 8.4 Hz, 2H), 2.43–2.34 (m, 7H), 2.31 (s, 4H), 2.19 (s, 3H), 2.05 (s, 3H), 1.86 (s, 6H), 1.73 (s, 6H), 1.64–1.54 (m, 2H), 1.50–1.43 (m, 2H), 1.23 (s, 1H). ^13^C NMR (151 MHz, DMSO‐*d*_6_) *δ* 171.75, 164.66, 146.12, 142.00, 136.39, 128.76, 128.33, 124.55, 120.17, 118.09, 57.07, 54.00, 51.93, 45.04, 42.50, 36.04, 36.00, 35.20, 28.13, 25.40, 22.73.MS (ESI) (*m/z*): 529.5 (M+H)^+^527.7 (M‐H)^−^.HRMS (ESI) calcd for C_33_H_44_N_4_O_2_ [M+H]^+^: 529.3537; found: 529.3532. Purity: 99.84%

### *N*‐(4‐((3r,5r,7r)‐Adamantan‐1‐yl)Phenyl)−4‐(5‐Morpholinopentanamido)Benzamide(1‐3aq)

It was obtained as a white solid in 78% yield with the same procedure as 1‐3am.^1^H NMR (400 MHz, DMSO‐*d*_6_) *δ* 10.18 (s, 1H), 10.05 (s, 1H), 7.91 (d, *J* = 8.4 Hz, 2H), 7.70 (dd, *J* = 16.8, 8.4 Hz, 4H), 7.31 (d, *J* = 8.4 Hz, 2H), 3.60–3.50 (m, 4H), 2.42–2.23 (m, 8H), 2.05 (s, 3H), 1.85 (d, *J* = 2.9 Hz, 6H), 1.73 (s, 6H), 1.66–1.54 (m, 2H), 1.54–1.41 (m, 2H). ^13^C NMR (151 MHz, DMSO‐*d*_6_) *δ* 172.26, 165.16, 146.88, 142.73, 137.17, 129.51, 128.99, 125.18, 120.62, 66.68, 58.41, 53.83, 43.16, 36.74, 36.68, 35.86, 28.81, 25.98, 23.34.MS (ESI) (*m/z*): 516.5 (M+H)^+^514.6 (M‐H)^−^.HRMS (ESI) calcd for C_32_H_41_N_3_O_3_ [M+H]^+^: 516.3221; found: 516.3221. Purity: 99.64%

### *N*‐(4‐((3r,5r,7r)‐Adamantan‐1‐yl)Phenyl)−3‐(4‐(Dimethylamino)Butanamido)Benzamide(1‐3ar)

It was obtained as a white solid in 75% yield.^1^H NMR (400 MHz, DMSO‐*d*_6_) *δ* 10.23 (t, *J* = 9.2 Hz, 2H), 8.14 (d, *J* = 7.2 Hz, 1H), 7.85 (t, *J* = 8.0 Hz, 1H), 7.75–7.64 (m, 3H), 7.54–7.45 (m, 1H), 7.39–7.33 (m, 2H), 3.17–3.08 (m, 2H), 2.82 (d, *J* = 8.5 Hz, 6H), 2.48 (d, *J* = 7.8 Hz, 2H), 2.10 (s, 3H), 1.98 (t, *J* = 8.0 Hz, 2H), 1.90 (s, 6H), 1.78 (s, 6H). ^13^C NMR (151 MHz, DMSO‐*d*_6_) *δ* 170.66, 165.74, 146.83, 139.69, 137.05, 136.26, 129.18, 125.24, 122.47, 120.66, 119.18, 56.80, 43.16, 42.81, 36.67, 35.88, 33.20, 28.80, 20.20.MS (ESI) (*m/z*): 460.5 (M+H)^+^458.5 (M‐H)^−^.HRMS (ESI) calcd for C_29_H_37_N_3_O_2_ [M+H]^+^: 460.2959; found: 460.2956. Purity: 99.68%

### 4‐((3‐((4‐((3r,5r,7r)‐Adamantan‐1‐yl)Phenyl)Carbamoyl)Phenyl)Amino)‐*N*,*N*,*N*‐Trimethyl‐4‐Oxobutan‐1‐Aminium(1‐3as)

It was obtained as a white solid in 70% yield.^1^H NMR (400 MHz, DMSO‐*d*_6_) *δ* 10.25 (s, 1H), 10.18 (s, 1H), 8.11 (s, 1H), 7.87–7.78 (m, 1H), 7.73–7.60 (m, 3H), 7.46 (d, *J* = 8.0 Hz, 1H), 7.33 (d, *J* = 8.0 Hz, 2H), 3.07 (s, 11H), 2.11–1.95 (m, 6H), 1.86 (s, 7H), 1.74 (s, 6H). ^13^C NMR (151 MHz, DMSO‐*d*_6_) *δ* 169.84, 165.18, 146.33, 138.95, 136.27, 135.54, 128.60, 124.63, 121.88, 120.12, 118.54, 64.61, 52.10, 42.50, 36.00, 35.23, 32.17, 28.14, 17.92.MS (ESI) (*m/z*): 474.4 (M+H)^+^.HRMS (ESI) calcd for C_30_H_40_N_3_O_2_ [M+H]^+^: 474.3121; found: 474.3123. Purity: 99.90%

### *N*‐(4‐((3r,5r,7r)‐Adamantan‐1‐yl)Phenyl)−4‐(4‐(Dimethylamino)Butanamido)Benzamide(1‐3at)

It was obtained as a white solid in 73% yield.^1^H NMR (400 MHz, DMSO‐*d*_6_) *δ* 10.33 (s, 1H), 10.09 (s, 1H), 7.97 (d, *J* = 8.4 Hz, 2H), 7.73 (dd, *J* = 18.8, 8.4 Hz, 4H), 7.35 (d, *J* = 8.4 Hz, 2H), 3.20–3.07 (m, 2H), 2.83 (s, 7H), 2.49 (d, *J* = 7.8 Hz, 1H), 2.09 (s, 3H), 2.03–1.94 (m, 2H), 1.89 (s, 6H), 1.77 (s, 6H). ^13^C NMR (151 MHz, DMSO‐*d*_6_) *δ* 170.22, 164.45, 146.01, 141.76, 136.54, 129.11, 128.41, 124.55, 120.01, 118.09, 56.12, 42.54, 42.14, 36.05, 35.23, 32.62, 28.18, 19.43.MS (ESI) (*m/z*): 460.4 (M+H)^+^458.5 (M‐H)^−^.HRMS (ESI) calcd for C_29_H_37_N_3_O_2_ [M+H]^+^: 460.2959; found: 460.2961. Purity: 99.86%

### 4‐((4‐((4‐((3r,5r,7r)‐Adamantan‐1‐yl)Phenyl)Carbamoyl)Phenyl)Amino)‐*N*,*N*,*N*‐Trimethyl‐4‐Oxobutan‐1‐Aminium(1‐3au, HI‐104)

It was obtained as a yellow solid in 65% yield.^1^H NMR (400 MHz, DMSO‐*d*_6_) *δ* 10.32 (d, *J* = 8.4 Hz, 1H), 10.06 (d, *J* = 8.4 Hz, 1H), 7.93 (d, *J* = 8.0 Hz, 2H), 7.79–7.62 (m, 4H), 7.31 (d, *J* = 8.0 Hz, 2H), 3.12–3.04 (m, 11H), 2.06 (s, 5H), 1.87 (d, *J* = 8.4 Hz, 7H), 1.74 (d, *J* = 8.0 Hz, 7H). ^13^C NMR (151 MHz, DMSO‐*d*_6_) *δ* 170.11, 164.60, 146.23, 141.66, 136.31, 129.06, 128.38, 124.61, 120.17, 118.19, 64.62, 52.10, 42.50, 35.99, 35.22, 32.24, 28.13, 17.83.MS (ESI) (*m/z*): 474.4 (M+H)^+^.HRMS (ESI) calcd for C_30_H_40_N_3_O_2_ [M+H]^+^: 474.3121; found: 474.3126. Purity: 98.67%

### *N*‐(4‐((3r,5r,7r)‐Adamantan‐1‐yl)Phenyl)Picolinamide(1‐4a)

It was obtained as a white solid in 76% yield.^1^H NMR (400 MHz, Chloroform‐*d*) *δ* 9.90 (s, 1H), 8.52 (d, *J* = 4.8 Hz, 1H), 8.21 (d, *J* = 7.8 Hz, 1H), 7.81 (t, *J* = 8.0 Hz, 1H), 7.70–7.59 (m, 2H), 7.43–7.35 (m, 1H), 7.34–7.25 (m, 2H), 2.02 (s, 3H), 1.84 (s, 6H), 1.69 (s, 6H). ^13^C NMR (151 MHz, DMSO‐*d*_6_) *δ* 162.70, 150.45, 148.88, 147.06, 138.60, 136.25, 127.32, 125.33, 122.77, 120.54, 43.12, 36.67, 35.91, 28.80. MS (ESI) (*m*/*z*): 333.3 (M+H)^+^.HRMS (ESI) calcd for C_22_H_24_N_2_O [M+Na]^+^: 355.1781; found: 355.1777. Purity: 99.93%

### *N*‐(4‐((3r,5r,7r)‐Adamantan‐1‐yl)Phenyl)Nicotinamide(1‐4b)

It was obtained as a white solid in 81% yield.^1^H NMR (400 MHz, DMSO‐*d*_6_) *δ* 10.39 (s, 1H), 9.10 (s, 1H), 8.74 (s, 1H), 8.29 (d, *J* = 8.0 Hz, 1H), 7.70 (d, *J* = 8.4 Hz, 2H), 7.57 (dd, *J* = 8.0, 4.4 Hz, 1H), 7.35 (d, *J* = 8.4 Hz, 2H), 2.06 (s, 3H), 1.86 (s, 6H), 1.74 (s, 6H). ^13^C NMR (151 MHz, DMSO‐*d*_6_) *δ* 164.29, 152.50, 149.10, 147.12, 136.77, 135.87, 131.10, 125.32, 123.96, 120.66, 43.14, 36.67, 35.91, 28.80.MS (ESI) (*m/z*): 333.3 (M+H)^+^HRMS (ESI) calcd for C_22_H_24_N_2_O [M+H]^+^: 333.1961; found: 333.1964. Purity: 98.98%

### *N*‐(4‐((3r,5r,7r)‐Adamantan‐1‐yl)Phenyl)Isonicotinamide(1‐4c)

It was obtained as a white solid in 74% yield.^1^H NMR (400 MHz, DMSO‐*d*_6_) *δ* 10.41 (d, *J* = 8.4 Hz, 1H), 8.82–8.73 (m, 2H), 7.88–7.80 (m, 2H), 7.75–7.63 (m, 2H), 7.39–7.29 (m, 2H), 2.05 (s, 3H), 1.86 (s, 6H), 1.74 (s, 6H). ^13^C NMR (151 MHz, DMSO‐*d*_6_) *δ* 163.56, 150.09, 146.70, 141.81, 135.90, 124.73, 121.39, 120.11, 42.48, 36.01, 35.29, 28.15.MS (ESI) (*m/z*): 333.3 (M+H)^+^331.4 (M‐H)^−^.HRMS (ESI) calcd for C_22_H_24_N_2_O [M+H]^+^: 333.1961; found: 333.1964. Purity: 99.68%

### *N*‐(4‐((3r,5r,7r)‐Adamantan‐1‐yl)Phenyl)Pyrimidine‐4‐Carboxamide(1‐4d)

It was obtained as a yellow solid in 80% yield.^1^H NMR (400 MHz, Chloroform‐*d*) *δ* 9.77 (s, 1H), 9.23 (s, 1H), 8.96 (t, *J* = 4.0 Hz, 1H), 8.16 (s, 1H), 7.64 (d, *J* = 5.6 Hz, 2H), 7.33 (d, *J* = 5.6 Hz, 2H), 2.04 (s, 3H), 1.85 (s, 6H), 1.71 (s, 6H). ^13^C NMR (151 MHz, DMSO‐*d*_6_) *δ* 161.61, 160.33, 158.31, 157.27, 147.64, 135.82, 125.39, 120.93, 119.35, 43.08, 36.65, 28.79.MS (ESI) (*m*/*z*): 334.3 (M+H)^+^.HRMS (ESI) calcd for C_21_H_23_N_3_O [M+H]^+^: 334.1914; found: 334.1913. Purity: 98.71%

### *N*‐(4‐((3r,5r,7r)‐Adamantan‐1‐yl)Phenyl)−5‐Methoxypicolinamide(1‐4e)

It was obtained as a white solid in 78% yield.^1^H NMR (400 MHz, Chloroform‐*d*) *δ* 9.77 (d, *J* = 12.4 Hz, 1H), 8.32–8.12 (m, 2H), 7.76–7.62 (m, 2H), 7.41–7.19 (m, 4H), 3.92 (d, *J* = 12.4 Hz, 3H), 2.07 (s, 3H), 1.89 (s, 6H), 1.74 (s, 6H). ^13^C NMR (151 MHz, Chloroform‐*d*) *δ* 161.28, 157.40, 146.70, 142.03, 135.73, 134.78, 124.82, 122.95, 119.78, 118.77, 55.20, 42.61, 36.18, 35.30, 28.35.MS (ESI) (*m/z*): 363.3 (M+H)^+^.HRMS (ESI) calcd for C_23_H_26_N_2_O_2_ [M+H]^+^: 363.2067; found: 363.2058. Purity: 95.92%

### *N*‐(4‐((3r,5r,7r)‐Adamantan‐1‐yl)Phenyl)−5‐Methylpyrazine‐2‐Carboxamide(1‐4f)

It was obtained as a yellow solid in 73% yield.^1^H NMR (400 MHz, Chloroform‐*d*) *δ* 9.58 (s, 1H), 9.37 (d, *J* = 6.8 Hz, 1H), 8.43 (d, *J* = 6.8 Hz, 1H), 7.76–7.60 (m, 2H), 7.45–7.30 (m, 2H), 2.67 (s, 3H), 2.09 (s, 3H), 1.91 (s, 6H), 1.77 (s, 6H). ^13^C NMR (151 MHz, Chloroform‐*d*) *δ* 160.24, 156.67, 147.36, 142.99, 141.48, 141.24, 134.19, 124.95, 118.94, 42.58, 36.15, 35.35, 28.32, 21.27.MS (ESI) (*m/z*): 348.3 (M+H)^+^.HRMS (ESI) calcd for C_22_H_25_N_3_O [M+H]^+^: 348.2070; found: 348.2061. Purity: 99.72%

### *N*‐(4‐((3r,5r,7r)‐Adamantan‐1‐yl)Phenyl)−4‐Methoxypicolinamide(1‐4g)

It was obtained as a white solid in 75% yield.^1^H NMR (400 MHz, Chloroform‐*d*) *δ* 10.00 (s, 1H), 8.39 (d, *J* = 6.0 Hz, 1H), 7.83 (s, 1H), 7.71 (d, *J* = 7.2 Hz, 2H), 7.38 (d, *J* = 7.6 Hz, 2H), 6.95 (s, 1H), 3.93 (s, 3H), 2.10 (s, 3H), 1.92 (s, 6H), 1.77 (s, 6H). ^13^C NMR (151 MHz, DMSO‐*d*_6_) *δ* 167.20, 162.44, 152.39, 150.29, 147.09, 136.18, 125.35, 120.48, 113.27, 108.51, 56.24, 43.11, 36.66, 35.91, 28.80. MS (ESI) (*m/z*): 363.3 (M+H)^+^.HRMS (ESI) calcd for C_23_H_26_N_2_O_2_ [M+H]^+^: 363.2067; found: 363.2055. Purity: 96.04%

### *N*‐(4‐((3r,5r,7r)‐Adamantan‐1‐yl)Phenyl)−4‐Chloropicolinamide(1‐4h)

It was obtained as a white solid in 78% yield.^1^H NMR (400 MHz, Chloroform‐*d*) *δ* 9.85 (d, *J* = 10.8 Hz, 1H), 8.55–8.44 (m, 1H), 8.29 (d, *J* = 12.0 Hz, 1H), 7.75–7.62 (m, 2H), 7.52–7.42 (m, 1H), 7.39 (d, *J* = 9.2 Hz, 2H), 2.10 (s, 3H), 1.92 (s, 6H), 1.77 (s, 6H). ^13^C NMR (151 MHz, Chloroform‐*d*) *δ* 148.21, 125.89, 124.93, 122.42, 118.95, 42.59, 36.15, 35.35, 28.32.MS (ESI) (*m/z*): 367.2 (M+H)^+^.HRMS (ESI) calcd for C_22_H_23_ClN_2_O [M+H]^+^: 367.1572; found: 367.1559. Purity: 97.62%

### *N*‐(4‐((3r,5r,7r)‐Adamantan‐1‐yl)Phenyl)thiazole‐5‐Carboxamide(1‐4i)

It was obtained as a yellow solid in 75% yield.^1^H NMR (400 MHz, DMSO‐*d*_6_) *δ* 10.39 (d, *J* = 7.6 Hz, 1H), 9.29 (d, *J* = 7.6 Hz, 1H), 8.67 (d, *J* = 7.6 Hz, 1H), 7.71–7.48 (m, 2H), 7.44–7.21 (m, 2H), 2.04 (s, 3H), 1.86 (s, 6H), 1.73 (s, 6H). ^13^C NMR (151 MHz, DMSO‐*d*_6_) *δ* 159.01, 147.27, 144.64, 136.43, 125.41, 120.73, 43.11, 36.65, 35.92, 28.79. MS (ESI) (*m/z*): 339.2 (M+H)^+^. HRMS (ESI) calcd for C_20_H_22_N_2_OS [M+H]^+^: 339.1526; found: 339.1527. Purity: 99.94%

### *N*‐(4‐((3r,5r,7r)‐Adamantan‐1‐yl)Phenyl)Thiophene‐2‐Carboxamide(1‐4j)

It was obtained as a white solid in 85% yield.^1^H NMR (400 MHz, DMSO‐*d*_6_) *δ* 10.17 (s, 1H), 8.01 (d, *J* = 4.0 Hz, 1H), 7.85 (d, *J* = 4.8 Hz, 1H), 7.64 (d, *J* = 7.6 Hz, 2H), 7.33 (d, *J* = 8.4 Hz, 2H), 7.23 (d, *J* = 4.4 Hz, 1H), 2.06 (s, 3H), 1.86 (s, 6H), 1.74 (s, 6H). ^13^C NMR (151 MHz, DMSO‐*d*_6_) *δ* 160.18, 146.86, 140.70, 136.65, 132.14, 129.37, 128.49, 125.30, 120.65, 43.14, 36.67, 35.89, 28.80. MS (ESI) (*m/z*): 338.25 (M+H)^+^. HRMS (ESI) calcd for C_21_H_23_NOS [M+H]^+^: 338.1573; found: 338.1570. Purity: 99.03%

### *N*‐(4‐((3r,5r,7r)‐Adamantan‐1‐yl)Phenyl)−1*H*‐Imidazole‐4‐Carboxamide(1‐4k)

It was obtained as a white solid in 81% yield.^1^H NMR (600 MHz, DMSO‐*d*_6_) *δ* 13.38 (s, 1H), 9.91 (s, 1H), 7.89 (s, 1H), 7.72 (d, *J* = 8.4 Hz, 2H), 7.30 (d, *J* = 8.4 Hz, 2H), 6.76 (s, 1H), 2.06 (s, 3H), 1.86 (s, 6H), 1.74 (s, 6H).^13^C NMR (151 MHz, DMSO‐*d*_6_) *δ* 160.75, 147.25, 146.45, 136.76, 130.80, 125.16, 120.49, 106.02, 43.15, 36.68, 35.84, 28.81. MS (ESI) (*m/z*): 322.25 (M+H)^+^320.35 (M‐H)^−^. HRMS (ESI) calcd for C_20_H_23_N_3_O [M+H]^+^: 322.1914; found: 322.1912. Purity: 99.68%

### *N*‐(4‐((3r,5r,7r)‐Adamantan‐1‐yl)Phenyl)−1‐Methyl‐1*H*‐Imidazole‐5‐Carboxamide(1‐4l)

It was obtained as a pink solid in 76% yield.^1^H NMR (400 MHz, DMSO‐*d*_6_) *δ* 9.97 (s, 1H), 7.88–7.75 (m, 2H), 7.61 (d, *J* = 7.2 Hz, 2H), 7.30 (d, *J* = 7.2 Hz, 2H), 3.85 (s, 3H), 2.05 (s, 3H), 1.85 (d, *J* = 3.1 Hz, 6H), 1.74 (s, 6H). ^13^C NMR (151 MHz, DMSO‐*d*_6_) *δ* 158.86, 146.64, 142.88, 136.72, 133.29, 126.41, 125.23, 120.46, 43.15, 36.67, 35.86, 34.07, 28.80. MS (ESI) (*m/z*): 336.28 (M+H)^+^. HRMS (ESI) calcd for C_21_H_25_N_3_O [M+H]^+^: 336.2005; found: 336.2003. Purity: 98.02%

### *N*‐(4‐((3r,5r,7r)‐Adamantan‐1‐yl)Phenyl)−1*H*‐Pyrazole‐3‐Carboxamide(1‐4m)

It was obtained as a white solid in 64% yield.^1^H NMR (400 MHz, DMSO‐*d*_6_) *δ* 13.40 (s, 1H), 9.95 (s, 1H), 7.89 (s, 1H), 7.78–7.64 (m, 2H), 7.42–7.25 (m, 2H), 6.76 (s, 1H), 2.04 (s, 3H), 1.85 (s, 6H), 1.72 (s, 6H). ^13^C NMR (151 MHz, DMSO‐*d*_6_) *δ* 160.75, 147.25, 146.45, 136.76, 130.81, 125.15, 120.49, 106.01, 43.15, 36.68, 35.83, 28.81. MS (ESI) (*m/z*): 322.20 (M+H)^+^320.33 (M‐H)^−^. HRMS (ESI) calcd for C_20_H_23_N_3_O [M+H]^+^: 322.1914; found: 322.1911. Purity: 99.40%

### *N*‐(4‐((3r,5r,7r)‐Adamantan‐1‐yl)Phenyl)−1‐Methyl‐1*H*‐Pyrrole‐2‐Carboxamide(1‐4n)

It was obtained as a white solid in 81% yield.^1^H NMR (600 MHz, DMSO‐*d*_6_) *δ* 9.66 (s, 1H), 7.63 (d, *J* = 7.2 Hz, 2H), 7.31–7.26 (m, 2H), 7.00 (d, *J* = 10.8 Hz, 2H), 6.08 (s, 1H), 3.88 (d, *J* = 3.0 Hz, 3H), 2.06 (s, 3H), 1.86 (s, 6H), 1.74 (s, 6H). ^13^C NMR (151 MHz, DMSO‐*d*_6_) *δ* 160.22, 146.10, 137.26, 129.11, 125.95, 125.09, 120.35, 113.94, 107.19, 43.19, 36.69, 35.81, 28.81. MS (ESI) (*m/z*): 335.25 (M+H)^+^. HRMS (ESI) calcd for C_22_H_26_N_2_O [M+H]^+^: 335.2118; found: 335.2116. Purity: 99.07%

### *N*‐(4‐((3r,5r,7r)‐Adamantan‐1‐yl)Phenyl)Thiophene‐3‐Carboxamide(1‐4o)

It was obtained as a white solid in 83% yield.^1^H NMR (400 MHz, DMSO‐*d*_6_) *δ* 9.96 (s, 1H), 8.31 (s, 1H), 7.71–7.58 (m, 4H), 7.30 (d, *J* = 8.4 Hz, 2H), 2.04 (s, 3H), 1.84 (s, 6H), 1.72 (s, 6H). ^13^C NMR (151 MHz, DMSO‐*d*_6_) *δ* 161.17, 146.69, 138.39, 136.92, 129.93, 127.66, 127.32, 125.23, 120.60, 43.16, 36.67, 35.87, 28.80. MS (ESI) (*m/z*): 338.22 (M+H)^+^ HRMS (ESI) calcd for C_21_H_23_NOS [M+H]^+^: 338.1573; found: 338.1572. Purity: 98.07%

### *N*‐(4‐((3r,5r,7r)‐Adamantan‐1‐yl)Phenyl)−4‐Aminopicolinamide(1‐4p)

It was obtained as a white solid in 74% yield.^1^H NMR (400 MHz, DMSO‐*d*_6_) *δ* 10.30 (s, 1H), 8.05 (s, 1H), 7.71 (s, 2H), 7.26 (s, 3H), 6.59 (d, *J* = 5.2 Hz, 1H), 6.38 (d, *J* = 6.4 Hz, 2H), 2.00 (s, 3H), 1.79 (s, 6H), 1.68 (s, 6H). ^13^C NMR (151 MHz, DMSO‐*d*_6_) *δ* 163.23, 156.24, 150.63, 148.75, 146.76, 136.31, 125.33, 120.15, 111.08, 107.53, 43.13, 36.67, 35.88, 28.80.MS (ESI) (*m/z*): 348.3 (M+H)^+^.HRMS (ESI) calcd for C_22_H_25_N_3_O [M+H]^+^: 348.2070; found: 348.2055. Purity: 95.76%

### *N*‐(4‐((3r,5r,7r)‐Adamantan‐1‐yl)Phenyl)−6‐Aminopicolinamide(1‐4q)

It was obtained as a white solid in 69% yield.^1^H NMR (400 MHz, DMSO‐*d*_6_) *δ* 10.14 (s, 1H), 7.72 (d, *J* = 8.0 Hz, 2H), 7.62 (t, *J* = 8.0 Hz, 1H), 7.37 (d, *J* = 8.0 Hz, 2H), 7.29 (d, *J* = 7.2 Hz, 1H), 6.71 (d, *J* = 8.0 Hz, 1H), 6.33 (s, 2H), 2.08 (s, 3H), 1.88 (s, 6H), 1.76 (s, 6H). ^13^C NMR (151 MHz, DMSO‐*d*_6_) *δ* 162.89, 159.04, 148.23, 146.84, 138.92, 136.15, 125.55, 119.69, 112.25, 110.64, 43.14, 36.66, 35.91, 28.80. MS (ESI) (*m/z*): 348.3 (M+H)^+^.HRMS (ESI) calcd for C_22_H_25_N_3_O [M+H]^+^: 348.2070; found: 348.2061. Purity: 99.55%

### *N*‐(4‐((3r,5r,7r)‐Adamantan‐1‐yl)Phenyl)−3‐Aminopicolinamide(1‐4r)

It was obtained as a white solid in 65% yield.^1^H NMR (400 MHz, DMSO‐*d*_6_) *δ* 10.41 (s, 1H), 7.95 (s, 1H), 7.79 (d, *J* = 7.2 Hz, 2H), 7.37 (d, *J* = 8.0 Hz, 3H), 7.29 (d, *J* = 8.4 Hz, 1H), 7.00 (s, 2H), 2.12 (s, 3H), 1.92 (s, 6H), 1.80 (s, 6H). ^13^C NMR (151 MHz, DMSO‐*d*_6_) *δ* 166.17, 147.33, 146.57, 136.30, 136.06, 128.68, 128.19, 125.40, 125.29, 120.21, 43.15, 36.67, 35.86, 28.81.MS (ESI) (*m/z*): 348.3 (M+H)^+^.HRMS (ESI) calcd for C_22_H_25_N_3_O [M+H]^+^: 348.2070; found: 348.2057. Purity: 97.99%

### General Procedure for the Synthesis of Compounds 2‐3a–2‐3e, 3‐2a–3‐2i

The synthetic routes of 3‐2a–3‐2i are similar to Scheme 1. 2‐3a–2‐3e are synthesized as Scheme 2.

Add methyl 4‐aminobenzoate (755 mg, 5 mmol) and DIPEA (4 mL, 25 mmol) to a mixture of 2‐picolinic acid (800 mg, 6.5 mmol) and HATU (2.85 g, 7.5 mmol) in dry DMF (15 mL) and stir vigorously for 8–12 h. The reaction was stopped by adding water and extracted with ethyl acetate, the organic phase was washed with brine twice, dried over anhydrous Na_2_SO_4_, and filtered. The residue was concentrated in vacuo and purified by silica chromatography to get 2‐1. 2‐1 (256 mg, 1 mmol) was added to a solution of MeOH (3 mL) and 2 m NaOH (3 mL) and stirred vigorously for 5–8 h at 50 °C, the reaction was concentrated in vacuo after reactant was disappeared monitored by TLC and 10% aqueous HCl was dropped into the mixture while it was stirred gently, lots of white precipitations (2‐2) were filtered and dried in a vacuum oven for 24 h at 37 °C. 2‐3a–2‐3f were synthesized by 2‐2 with corresponding aminos.

### *N*‐(4‐(((1R,3S,5r,7r)‐Adamantan‐2‐yl)Carbamoyl)Phenyl)Picolinamide(2‐3a)

It was obtained as a white solid in 67% yield.^1^H NMR (400 MHz, DMSO‐*d*_6_) *δ* 10.84 (d, *J* = 9.2 Hz, 1H), 8.85–8.72 (m, 1H), 8.19 (t, *J* = 8.2 Hz, 1H), 8.14–8.06 (m, 1H), 8.01 (t, *J* = 8.4 Hz, 2H), 7.87 (t, *J* = 8.8 Hz, 3H), 7.71 (s, 1H), 4.04 (s, 1H), 2.12 (d, *J* = 13.2 Hz, 2H), 1.99 (s, 2H), 1.92–1.78 (m, 6H), 1.73 (d, *J* = 9.2 Hz, 2H), 1.52 (d, *J* = 14.4 Hz, 2H). ^13^C NMR (151 MHz, DMSO‐*d*_6_) *δ* 166.33, 163.22, 150.18, 148.95, 141.20, 138.69, 130.78, 128.79, 127.58, 122.99, 119.79, 54.49, 37.70, 37.43, 31.74, 31.60, 27.31. MS (ESI) (*m/z*): 376.3 (M+H)^+^. HRMS (ESI) calcd for C_23_H_25_N_3_O_2_ [M+H]^+^: 376.2020; found: 376.2030. Purity: 97.75%

### *N*‐(4‐(2‐((3r,5r,7r)‐Adamantan‐1‐yl)Acetamido)Phenyl)Picolinamide(2‐3b)

It was obtained as a white solid in 73% yield.^1^H NMR (400 MHz, DMSO‐*d*_6_) *δ* 10.55 (s, 1H), 9.75 (s, 1H), 8.74 (s, 1H), 8.15 (d, *J* = 8.0 Hz, 1H), 8.11–8.02 (m, 1H), 7.80 (d, *J* = 6.4 Hz, 2H), 7.72–7.62 (m, 1H), 7.61–7.53 (m, 2H), 2.04 (s, 2H), 1.93 (s, 3H), 1.71–1.55 (m, 12H). ^13^C NMR (151 MHz, DMSO‐*d*_6_) *δ* 168.96, 162.04, 149.68, 148.27, 137.97, 135.16, 133.28, 126.70, 122.09, 120.47, 119.29, 50.64, 41.89, 36.23, 32.56, 27.86.MS (ESI) (*m/z*): 390.3 (M+H)^+^.HRMS (ESI) calcd for C_24_H_27_N_3_O_2_ [M+H]^+^: 390.2176; found: 390.2178. Purity: 98.81%

### *N*‐(4‐((1‐((3r,5r,7r)‐Adamantan‐1‐yl)Ethyl)Carbamoyl)Phenyl)Picolinamide(2‐3c)

It was obtained as a pink solid in 75% yield.^1^H NMR (400 MHz, DMSO‐*d*_6_) *δ* 10.82 (d, *J* = 8.8 Hz, 1H), 8.75 (s, 1H), 8.22–8.13 (m, 1H), 8.12–8.03 (m, 1H), 8.03–7.94 (m, 2H), 7.91–7.77 (m, 3H), 7.74–7.64 (m, 1H), 3.87–3.75 (m, 1H), 1.93 (s, 3H), 1.71–1.41 (m, 12H), 1.11–0.98 (m, 3H). ^13^C NMR (151 MHz, DMSO‐*d*_6_) *δ* 165.46, 162.63, 149.39, 148.35, 140.44, 138.07, 130.09, 127.98, 126.97, 122.33, 119.26, 52.38, 37.99, 36.49, 36.04, 27.64, 13.79.MS (ESI) (*m/z*): 404.4 (M+H)^+^.HRMS (ESI) calcd for C_25_H_29_N_3_O_2_ [M+H]^+^: 404.2333; found: 404.2335. Purity: 99.77%

### *N*‐(4‐((1r,3s,5R,7S)−3‐Hydroxyadamantane‐1‐Carboxamido)Phenyl)Picolinamide(2‐3d)

It was obtained as a pink solid in 68% yield.^1^H NMR (600 MHz, DMSO‐*d*_6_) *δ* 10.55 (s, 1H), 9.15 (s, 1H), 8.74 (d, *J* = 4.8 Hz, 1H), 8.15 (d, *J* = 7.8 Hz, 1H), 8.07 (t, *J* = 7.8 Hz, 1H), 7.81 (d, *J* = 8.4 Hz, 2H), 7.70–7.60 (m, 3H), 4.53 (s, 1H), 2.19 (s, 2H), 1.83–1.75 (m, 6H), 1.64–1.50 (m, 6H).^13^C NMR (151 MHz, DMSO‐*d*_6_) *δ* 175.43, 162.62, 150.47, 148.88, 138.60, 135.86, 134.13, 127.30, 122.74, 120.99, 67.16, 46.80, 44.77, 37.89, 35.35, 30.40. MS (ESI) (*m/z*): 392.31 (M+H)^+^. HRMS (ESI) calcd for C_23_H_25_N_3_O_3_ [M+H]^+^: 392.2969; found: 392.1972.Purity: 96.08%

### *N*‐(4‐(((1r,3R,5S,7r)−3,5‐Dimethyladamantan‐1‐yl)Carbamoyl)Phenyl)Picolinamide(2‐3e)

It was obtained as a white solid in 75% yield.^1^H NMR (600 MHz, DMSO‐*d*_6_) *δ* 10.79 (s, 1H), 8.75 (s, 1H), 8.17 (d, *J* = 7.8 Hz, 1H), 8.09 (t, *J* = 7.8 Hz, 1H), 7.97 (d, *J* = 7.2 Hz, 2H), 7.80 (d, *J* = 6.0 Hz, 2H), 7.72–7.67 (m, 1H), 7.53 (s, 1H), 2.12 (s, 1H), 1.91 (s, 2H), 1.73 (q, *J* = 12.0 Hz, 4H), 1.36 (d, *J* = 12.6 Hz, 2H), 1.28 (d, *J* = 12.6 Hz, 2H), 1.14 (t, *J* = 13.8 Hz, 2H), 0.84 (s, 6H).^13^C NMR (151 MHz, DMSO‐*d*_6_) *δ* 165.96, 163.19, 150.18, 148.95, 141.00, 138.69, 131.53, 128.50, 127.57, 122.97, 119.72, 53.48, 50.82, 47.47, 42.88, 32.42, 30.64, 30.09. MS (ESI) (*m/z*): 404.33 (M+H)^+^. HRMS (ESI) calcd for C_25_H_29_N_3_O_2_ [M+H]^+^: 404.2333; found: 404.2339. Purity: 97.38%

### *N*‐(4‐((3r,5r,7r)‐Adamantan‐1‐yl)−2,6‐Dimethylphenyl)−3‐Chlorobenzamide(3‐2a)

It was obtained as a yellow solid in 54% yield.^1^H NMR (600 MHz, DMSO‐*d*_6_) *δ* 9.80 (s, 1H), 8.01 (s, 1H), 7.97–7.93 (m, 1H), 7.70–7.64 (m, 1H), 7.57 (t, *J* = 7.8 Hz, 1H), 7.10 (s, 2H), 2.17 (s, 6H), 2.08–2.04 (m, 3H), 1.88 (d, *J* = 3.0 Hz, 6H), 1.78–1.70 (m, 6H). ^13^C NMR (151 MHz, DMSO‐*d*_6_) *δ* 164.20, 149.75, 136.91, 135.29, 133.79, 132.89, 131.76, 130.97, 127.75, 126.70, 124.63, 43.13, 36.70, 35.86, 28.82, 18.79. MS (ESI) (*m/z*): 394.27 (M+H)^+^. HRMS (ESI) calcd for C_25_H_28_ClNO [M+H]^+^: 394.1932; found: 394.1930. Purity: 98.04%

### *N*‐(4‐((3r,5r,7r)‐Adamantan‐1‐yl)−2,6‐Diisopropylphenyl)−3‐Chlorobenzamide(3‐2b)

It was obtained as a yellow solid in 48% yield.^1^H NMR (400 MHz, DMSO‐*d*_6_) *δ* 9.75 (s, 1H), 7.99 (s, 1H), 7.93 (d, *J* = 7.2 Hz, 1H), 7.67 (d, *J* = 8.0 Hz, 1H), 7.57 (t, *J* = 8.0 Hz, 1H), 7.15 (s, 2H), 3.09–2.96 (m, 2H), 2.07 (s, 3H), 1.90 (s, 6H), 1.75 (s, 6H), 1.16 (d, *J* = 6.8 Hz, 6H), 1.10 (d, *J* = 7.2 Hz, 6H). ^13^C NMR (151 MHz, DMSO‐*d*_6_) *δ* 165.35, 150.37, 145.65, 136.88, 133.83, 132.00, 131.81, 131.06, 130.38, 129.14, 127.70, 126.70, 119.60, 43.18, 36.70, 36.37, 28.84, 24.13, 23.80. MS (ESI) (*m/z*): 450.31 (M+H)^+^. HRMS (ESI) calcd for C_29_H_36_ClNO [M+H]^+^: 450.2558; found: 450.2562. Purity: 99.10%

### *N*‐(5‐((3r,5r,7r)‐Adamantan‐1‐yl)Pyrimidin‐2‐yl)−3‐Chlorobenzamide(3‐2c)

It was obtained as a yellow solid in 42% yield.^1^H NMR (600 MHz, DMSO‐*d*_6_) *δ* 8.71 (d, *J* = 4.8 Hz, 2H), 7.33 (t, *J* = 4.8 Hz, 1H), 7.28–7.24 (m, 1H), 7.17 (t, *J* = 7.8 Hz, 1H), 7.14 (t, *J* = 1.8 Hz, 1H), 7.08 (dt, *J* = 7.2, 1.8 Hz, 1H), 2.19 (s, 6H), 2.08 (s, 3H), 1.65 (s, 6H). ^13^C NMR (151 MHz, DMSO‐*d*_6_) *δ* 168.96, 160.65, 158.94, 140.76, 132.78, 130.18, 129.48, 127.42, 126.22, 120.59, 60.57, 36.32, 29.85.MS (ESI) (*m/z*): 368.20(M+H)^+^. HRMS (ESI) calcd for C_21_H_22_ClN_3_O [M+H]^+^: 368.1524; found: 368.1524. Purity: 99.52%

### 4‐Acetamido‐*N*‐(4‐((1S,3R,5S,6S,7S)−4,6‐Dimethyladamantan‐1‐yl)Phenyl)Benzamide(3‐2d)

It was obtained as a yellow solid in 63% yield.^1^H NMR (400 MHz, DMSO‐*d*_6_) *δ* 10.39 (s, 1H), 10.09 (s, 1H), 7.92 (d, *J* = 8.4 Hz, 2H), 7.71 (dd, *J* = 17.2, 8.0 Hz, 4H), 7.30 (d, *J* = 8.4 Hz, 2H), 2.17–2.06 (m, 4H), 1.68 (s, 2H), 1.53–1.32 (m, 8H), 1.18 (s, 2H), 0.85 (s, 6H). ^13^C NMR (151 MHz, DMSO‐*d*_6_) *δ* 168.63, 164.54, 145.30, 142.10, 136.65, 128.86, 128.35, 124.62, 120.04, 117.92, 50.20, 48.94, 42.28, 41.10, 37.07, 31.00, 30.42, 29.27, 23.96.MS (ESI) (*m/z*): 417.4 (M+H)^+^415.5 (M‐H)^−^.HRMS (ESI) calcd for C_27_H_32_N_2_O_2_ [M+H]^+^: 417.2537; found: 417.2540. Purity: 97.34%

### 3‐Chloro‐*N*‐(4‐((1r,3R,5S,7r)−3,5‐Dimethyladamantan‐1‐yl)Phenyl)Benzamide(3‐2e)

It was obtained as a yellow solid in 70% yield.^1^H NMR (400 MHz, DMSO‐*d*_6_) *δ* 10.27 (s, 1H), 8.00 (s, 1H), 7.91 (d, *J* = 8.0 Hz, 1H), 7.72–7.61 (m, 3H), 7.55 (t, *J* = 8.0 Hz, 1H), 7.32 (d, *J* = 8.4 Hz, 2H), 2.13 (s, 1H), 1.67 (s, 2H), 1.52–1.30 (m, 8H), 1.17 (s, 2H), 0.85 (s, 6H). ^13^C NMR (151 MHz, DMSO‐*d*_6_) *δ* 164.23, 146.39, 137.42, 136.86, 133.66, 131.76, 130.84, 127.83, 126.91, 125.37, 120.71, 49.53, 42.89, 41.71, 37.74, 31.63, 31.05, 29.90. MS (ESI) (*m*/*z*): 394.3 (M+H)^+^. HRMS (ESI) calcd for C_25_H_28_ClNO [M+H]+: 394.1932; found: 394.1930. Purity: 99.86%

### 3‐Chloro‐*N*‐(4‐((1r,3R,5S,7r)−3,5‐Dimethyladamantan‐1‐yl)−2,6‐Dimethylphenyl)Benzamide(3‐2f)

It was obtained as a white solid in 59% yield.^1^H NMR (400 MHz, DMSO‐*d*_6_) *δ* 9.80 (s, 1H), 8.01 (s, 1H), 7.94 (d, *J* = 7.2 Hz, 1H), 7.67 (d, *J* = 8.4 Hz, 1H), 7.57 (td, *J* = 8.0, 3.2 Hz, 1H), 7.09 (s, 2H), 2.15 (s, 6H), 1.70 (s, 2H), 1.57–1.31 (m, 9H), 1.19 (s, 2H), 0.87 (d, *J* = 3.2 Hz, 6H). ^13^C NMR (151 MHz, DMSO‐*d*_6_) *δ* 164.15, 149.14, 136.92, 135.32, 133.78, 132.93, 131.77, 131.05, 130.97, 127.75, 126.71, 124.72, 50.84, 49.49, 42.92, 41.74, 37.73, 31.65, 31.07, 29.91, 18.76. MS (ESI) (*m/z*): 422.3 (M+H)^+^.HRMS (ESI) calcd for C_27_H_32_ClNO [M+H]^+^: 422.2245; found: 422.2241. Purity: 97.74%

### 3‐Chloro‐*N*‐(5‐((1r,3R,5S,7r)−3,5‐Dimethyladamantan‐1‐yl)Pyrimidin‐2‐yl)Benzamide(3‐2g)

It was obtained as a white solid in 53% yield.^1^H NMR (600 MHz, DMSO‐*d*_6_) *δ* 8.74–8.69 (m, 2H), 7.33 (t, *J* = 4.8 Hz, 1H), 7.28–7.24 (m, 1H), 7.17 (t, *J* = 7.8 Hz, 1H), 7.15–7.12 (m, 1H), 7.09–7.05 (m, 1H), 2.14 (s, 1H), 1.98 (s, 2H), 1.86 (s, 4H), 1.38–1.33 (m, 2H), 1.31–1.21 (m, 3H), 1.14 (s, 2H), 0.82 (s, 6H). ^13^C NMR (151 MHz, DMSO‐*d*_6_) *δ* 169.03, 160.67, 158.97, 140.73, 132.78, 130.17, 129.48, 127.41, 126.19, 120.63, 62.10, 50.45, 45.89, 42.56, 38.69, 32.90, 30.65, 30.39. MS (ESI) (*m/z*): 396.14 (M+H)^+^. HRMS (ESI) calcd for C_23_H_26_ClN_3_O [M+H]^+^: 396.1837; found: 396.1840. Purity: 98.90%

### 3‐Chloro‐*N*‐(4‐((1r,3R,5S,7r)−3,5‐Dimethyladamantan‐1‐yl)−2,6‐Diethylphenyl)Benzamide(3‐2h)

It was obtained as a white solid in 47% yield.^1^H NMR (600 MHz, DMSO‐*d*_6_) *δ* 9.78 (s, 1H), 8.00 (s, 1H), 7.94 (d, *J* = 7.8 Hz, 1H), 7.68 (d, *J* = 7.8 Hz, 1H), 7.58 (t, *J* = 7.8 Hz, 1H), 7.11 (s, 2H), 2.55–2.51 (m, 4H), 2.16 (s, 1H), 1.73 (s, 2H), 1.55 (d, *J* = 12.0 Hz, 2H), 1.49 (d, *J* = 12.6 Hz, 2H), 1.43 (d, *J* = 12.0 Hz, 2H), 1.37 (d, *J* = 12.0 Hz, 2H), 1.21 (s, 2H), 1.10 (t, *J* = 7.8 Hz, 6H), 0.88 (s, 6H). ^13^C NMR (151 MHz, DMSO‐*d*_6_) *δ* 164.99, 149.70, 141.50, 136.96, 133.82, 131.79, 131.77, 131.04, 127.68, 126.66, 123.17, 50.85, 49.49, 42.92, 41.76, 37.93, 31.67, 31.08, 29.91, 25.36, 15.33.MS (ESI) (*m/z*): 450.32 (M+H)^+^. HRMS (ESI) calcd for C_29_H_36_ClNO [M+H]^+^: 450.2558; found: 450.2555. Purity: 98.42%

### *N*‐(4‐((3r,5r,7r)‐Adamantan‐1‐yl)−2,6‐Diethylphenyl)−3‐Chlorobenzamide(3‐2i)

It was obtained as a white solid in 43% yield.^1^H NMR (600 MHz, DMSO‐*d*_6_) *δ* 9.78 (s, 1H), 8.00 (s, 1H), 7.94 (d, *J* = 7.8 Hz, 1H), 7.68 (d, *J* = 7.8 Hz, 1H), 7.59 (t, *J* = 7.8 Hz, 1H), 7.12 (s, 2H), 2.56–2.51 (m, 4H), 2.08 (s, 3H), 1.90 (s, 6H), 1.76 (d, *J* = 3.2 Hz, 6H), 1.11 (t, *J* = 7.8 Hz, 6H). ^13^C NMR (151 MHz, DMSO‐*d*_6_) *δ* 165.01, 150.29, 141.42, 136.97, 133.82, 131.79, 131.74, 131.04, 127.68, 126.66, 123.02, 43.16, 36.71, 36.08, 28.82, 25.33, 15.25.MS (ESI) (*m/z*): 422.33 (M+H)^+^ 420.48(M‐H)^−^. HRMS (ESI) calcd for C_27_H_32_ClNO [M+H]^+^: 422.2245; found: 422.2242. Purity: 96.38%

### Cell Culture

HEK293T and human liver cancer cell line MHCC97L cells were cultured in Dulbecco's Modified Eagle Medium (High Glucose, Basal Media, L110KJ) supplemented with 10% fetal bovine serum (FBS, Sigma) and 1% Double antibody penicillin‐Streptomycin Solution Liquid (100×) at 37.0 °C in 5% CO_2_ in humidified atmosphere. Hypoxia was induced by culturing cells in a specialized, hypoxia chamber (Thermo Fisher) flushed with a mixed gas of 1% O_2_, 5% CO_2_, and 94% N_2_.

### Western Blot Analysis

Cells were lysed with RIPA lysis (Epizyme, PC101) containing 1% cocktail protease inhibitors (MCE), and protein concentrations were determined by BCA kits (Beyotime, P0012S). Samples were denatured using DS‐PAGE Protein Staining and Loading Buffer (5×) (Beyotime, P0015L) followed by boiling for 10 min at 98 °C then the proteins were fractionated by 10% or 12.5% SDS‐PAGE and transferred to Cellulose Nitrate Membrane Filters (WHATMAN). After blocking in 5% non‐fat milk for 2 h at room temperature, the membrane was incubated overnight with specific antibodies at 4 °C, followed by horseradish peroxidase (HRP)‐linked anti‐mouse or anti‐rabbit secondary antibody (Cell Signaling Technology). Finally, An Immobilon Western Chemiluminescent HRP Substrate Kit (Merck Millipore) was used for protein bands detection. In addition, blots were probed with anti‐*β*‐actin (Merck, Darmstadt, Germany) antibody as the internal control.

### High‐Throughput Screening

HEK293T cells were stably transfected with reporter plasmids pGMLV‐HRE‐LUC‐puromycin, pMSCV‐renilla‐hygromycin, and pLenti‐HIF‐1*α*P2A‐neomycin. Stable cell line constructed above, 293‐HIF‐1*α*P2A‐HRE‐Fluc‐Rluc, were seeded in 384‐well plates and cultured in DMEM with 10% FBS. The following day, the cells were pretreated with indicated chemical compounds from the National Compound Library of the Shanghai Institute of Materia Medica, Chinese Academy of Sciences by Mosquito HTS. The plate was then placed in an incubator chamber and incubated at 37 °C for 24 h. The ratio of firefly/renilla luciferase activity was determined by using the Envision (PerkinElmer).

### Luciferase Assay

Stable cell line 293‐HRE‐Fluc‐Rluc expressed luciferase and renilla or HEK293T cells transfected with indicated plasmids (pGL3‐HRE‐luciferase and pSV40‐renilla) were seeded in a 96‐well plate. The cells were co‐incubated with indicated compounds for 24 h and luciferase activities were detected in Microplate Reader (BioTek SYNEGRY H1) using the Dual Luciferase Reporter Assay System Kit (Promega, USA E1910) according to the manufacturer's instruction. The relative Luc activity was normalized by renilla activity. Three independent experiments were performed, and the calculated means and SDs are presented.

### Quantitative Real‐Time PCR

Total RNA was isolated using Trizol reagent (Invitrogen). Complementary DNA was synthesized using a Reverse Transcriptase reagent (Promega). Fluorescence real‐time PCR was performed with the double‐stranded DNA dye SYBR Master Mix (Roche Diagnostics Corporation, Indianapolis, IN, USA) and the Applied Biosystems Step One Plus detection system (ABI 7900). The upstream and downstream gene‐specific primers were as follows.

HIF‐1*α* sense: 5′‐TGCTTGCCAAAAGAGGTGGA‐3′,
HIF‐1*α* antisense: 5′‐GGGGCCAGCAAAGTTAAAGC‐3′;
HIF‐1*β* sense: 5′‐GCAGGAATGGACTTGGCTCT‐3′,
HIF‐1*β* antisense:5′‐GGCAAAACTTGCTTCCCTGG‐3′;
VEGF sense: 5′‐GCAGAATCATCACGAAGTGG‐3′,
VEGF antisense: 5′‐GCATGGTGATGTTGGACTCC‐3′;
PDK1 sense:5′‐GAGAGCCACTATGGAACACCA‐3′,
PDK1 antisense:5′‐GGAGGTCTCAACACGAGGT‐3′;
*β*‐actin sense: 5′‐CATCCTCACCCTGAAGTACCC‐3′,
*β*‐actin antisense: 5′‐AGCCTGGATAGCAACGTACATG‐3′;
ATP5D sense:5′‐ CACGCAGGTGTTCTTCAACG‐3′,
ATP5D antisense: 5′‐AGTATTTGGAGGTGGTGCCG‐3′;
ATP5E sense: 5′‐ GACAGGCTGGACTCAGCTAC‐3′,
ATP5E antisense:5′‐TTTACGTTGCTGCCAGAAGTC‐3′;
ATP5F1 sense: 5′‐ CTGTCCCGGGTGGTACTTTC‐3′,
ATP5F1 antisense:5′‐GGTACAGGGACAAGGTGTGG‐3′.

### Polysome Profiling

Polysome RNA preparations and analysis were carried out as previously described.^[^ ^60^ ^]^ Briefly, cells were seeded in 10 cm dishes, and washed with cold PBS containing 100 µg mL^−1^ cycloheximide, and then lysed in a hypotonic lysis buffer [5 mm *tris*‐HCl (pH 7.5), 2.5 mm MgCl_2_, 1.5 mm KCl, 100 µg mL^−1^ cycloheximide, 2 mm DTT, 0.5% Triton X‐100, and 0.5% sodium deoxycholate]. A lysate sample was used to isolate the cytoplasmic RNA using TRIzol (Invitrogen). Lysates were loaded onto 10–50% (wt vol^−1^) sucrose density gradients [20 mm HEPES‐KOH (pH 7.6), 100 mm KCl, 5 mm MgCl_2_] and centrifuged at 36 000 rpm for 2 h at 4 °C. Gradients were fractionated, and the optical density at 260 nm was continuously detected by a UV detector and fraction collector (Teledyne ISCO). RNA from each fraction was isolated using TRIzol (Invitrogen). Fractions with mRNA associated with polysomes were pooled and HIF‐1*α* mRNA abundance was detected by RT‐PCR amplification in each fraction of the polysome gradient. Fractions 3–4 correspond to the top of the gradient (Free mRNAs) and fractions 19–23 correspond to the bottom of the gradient (Heavy mRNAs). Protein translation level was associated with the heavy mRNAs. Results were normalized to free mRNAs group (fractions 3–4).

### AHA Labeling Assay

HEK293T cells seeded in 10 cm dishes were added with DMSO or HI‐101 at concentrations 10 or 20 µm, then cultured in hypoxia incubator. The next day, cells were washed twice with PBS and incubated in DMEM without methionine for 4 h. At the same time, add l‐homopropargylglycine (AHA) to all groups, and the positive control group treatment with cycloheximide (CHX). AHA concentration and incubation time were 50 µm and 4 h. Cells were collected and washed twice with phosphate‐buffered saline, and then lysed in lysis buffer containing 1% SDS and 50 mm tris, pH 7.5 and 1× protease inhibitors (PMSF, cocktail). Lysates were then sonicated for 1 min and centrifuged to pellet cellular debris. The lysates were subjected to click reaction for 1 h with 40 µm biotin‐PEG4‐alkyne using Click‐iT protein reaction buffer kit according to the manufacturer's instructions (Invitrogen). Total proteins from click reaction were precipitated with methanol/chloroform and resolubilized in 50 mm tris, pH 7.5, 0.01% SDS in a concentration of 1 µg µL^−1^. Biotin‐tagged proteins (1 mg) were then incubated with 10 µL of Streptavidin beads (Dynabeads M‐280 Streptavidin, Invitrogen) for 2 h at room temperature. After extensive washing in PBS with 0.5% SDS for five times to remove nonspecific binding or protein–protein interactions, resin suspensions were incubated in 50 µL of 2× loading buffer for 10 min at 98 °C to separate out the tagged proteins from beads. The immunoprecipitated proteins were subjected to SDS‐PAGE. In parallel, immunoprecipitations were immunoblotted with anti‐biotin antibodies to confirm AHA labeling.

### Pull‐Down and MS Analysis

The HEK293T cells were lysed in 1× RIPA (Epizyme, PC101) lysis buffer containing 1× protease inhibitor (PMSF and cocktail). Lysates were then sonicated for 1 min and centrifuged to pellet cellular debris at 4 °C. Then 293T cell lysates were incubated with biotin or HI‐102 (10 µm) overnight at 4 °C. Then 10 µL of Streptavidin Agarose beads were added and incubated overnight at 4 °C. And the beads were washed five times with 1 mL of RIPA buffer. Then the bead‐bound proteins were separated by SDS‐PAGE and visualized by silver staining. The protein‐containing band in the gel was excised, followed by in‐gel digestion and analysis by LC‐MS/MS.

### Immunofluorescence Staining

HEK293T cells were cultured in a 3.5 cm dish with glass bottom. After waiting for the cells to adhere to the wall, add HI‐102 at the concentration 10 µm or equal volume DMSO. 24 h later, add Mito Tracker Red 200 nm and continue to incubate for 15–45 min. Subsequently, the cultured cells were fixed in 4% formaldehyde and permeabilized with 100% cold methanol, and washed in phosphate‐buffered saline (pH 7.4). Then the cells were performed with DAPI (4′,6‐diamidino‐2‐phenylindole dihydrochloride). Fluorescence images were taken and analysis was performed with a laser scanning confocal microscope.

### Fluorescence Polarization

The absorption wavelength and emission wavelength of HI‐103 were measured by ultraviolet spectrophotometer and the results were 493 nm for absorption and 537 nm for emission. F_1_‐ATPase at indicated concentrations was incubated with HI‐103 (4 nm), bodipy‐1 was as a negative molecule, conduct the experiment after incubating in 96‐well microplates of black polystyrene for 10 min. The FP results were analyzed with GraphPad Prism 9.0.

### ATP5A and ATP5B Knockout Cell Lines by CRISR‐Cas9

CRISPR‐Cas9 technology was used to construct a stable transfection cell line. And the specific method was as follows. ATP5A‐KO and ATP5B‐KO cell lines were generated according to a previously published protocol. Briefly, gRNA targeting ATP5A, ACCAACTCGCCTACGCGTCT, and gRNA targeting ATP5B TGGCAAGACTGTACTGATCA, were cloned into the vector lentiCRISPR v2 (Addgene plasmid no. 52961). The lentiCRISPR v2 vector containing gRNA, psPAX2, and pMD2G was mixed and transfected with Lipofectamine 2000 (Thermo Fisher Scientific) into HEK293T cells. Lentiviruses were harvested for 48 h after transfection. Viral supernatant was used to infect the indicated cells with 8 µg mL^−1^ polybrene (Sigma Aldrich). The infected cells were selected in a medium containing puromycin (Sigma Aldrich). The stable cell lines were examined by western blotting.

### ATP Synthase Subunits Knockdown Cell Lines Construction by shRNA

ShRNA oligonucleotides targeting ATP5A/ATP5B/ATP5C/ATP5D/ATP5E were designed and synthesized. For gene silencing, shRNA was cloned into the pLK0.1‐puro vector. The lentivirused supernatant was packaged in HEK293T cells by co‐transfecting with psPAX2 and pMD2G. The viral supernatant was collected after transfection for 48 h. The cells were treated with viral supernatant and polybrene (8 µg mL^−1^).

### Complex V Activity Assay

The activity of complex V was determined using the MitoTox Complex V OXPHOS Activity Assay kit from Abcam (ab109907, Cambridge, MA, USA), according to the instructions. The activity of complex V was measured by monitoring the change in absorbance at 340 nm over a period of 2 h at 30 °C. Oligomycin (Selleck, S1478) was used as a positive control for the assay.

### Tumor Xenograft Experiment

MHCC97L wildtype and ATP5A or ATP5B stable knock‐out cells (3 × 10^6^) were implanted subcutaneously into the right flanks of 6‐week‐old female BALB/c nude mice. After most of the tumors had reached a threshold size of 100 mm^3^, the mice received an intraperitoneal injection of vehicle alone (10% DMSO, 10% Cremophor, and 80% saline, pH 7–8) or HI‐104 over 10 days. The tumor volume was monitored every other day with calipers. After 10 days of treatment or if the mice met humane endpoint criteria, the mice were sacrificed, and tumors were dissected, photographed, and weighed. Tumor tissues were either collected and fixed in 10% neutral‐buffered formalin or snap‐frozen in liquid N_2_ and stored at −80 °C for subsequent analyses. All animals used in this study were handled in accordance with federal and institutional guidelines under a protocol approved by the Institutional Animal Care and Use Committee at Shanghai Jiao Tong University School of Medicine.

## Conflict of Interest

The authors declare no conflict of interest.

## Supporting information

Supporting Information

## Data Availability

The data that support the findings of this study are available in the Supporting Information of this article.
