# Supplementary material for: Adamantaniline Derivatives Target ATP5B to Inhibit Translation of Hypoxia Inducible Factor‐1α
Source: Adv Sci (Weinh). 2023 Jul 3;10(25):2301071. doi: 10.1002/advs.202301071 (PMC10477886; doi:10.1002/advs.202301071)
Supplement: Supplementary file 1 — Supporting Information [file ADVS-10-2301071-s001.pdf]

ADVANCED SCIENCE
Open Access

Supporting Information

for *Adv. Sci.*, DOI 10.1002/advs.202301071

Adamantaniline Derivatives Target ATP5B to Inhibit Translation of Hypoxia Inducible Factor-1α

Huiti Li, Yali Liu, Zian Xue, Li Zhang, Xiaoxue Ruan, Jintong Yang, Zhongjiao Fan, Hongfang Zhao, Yu Cao, Guoqiang Chen, Ying Xu and Lu Zhou**

Contents of SI

Figure S1-S15...1-16
The specific synthesis routes..17-18
^{1}H & ^{13}C NMR spectra...19-97
HPLC data 98-120

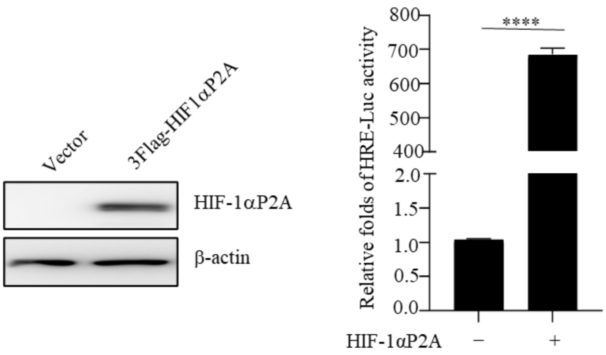

Figure S1. Validation of dual luciferase reporter assay screening system

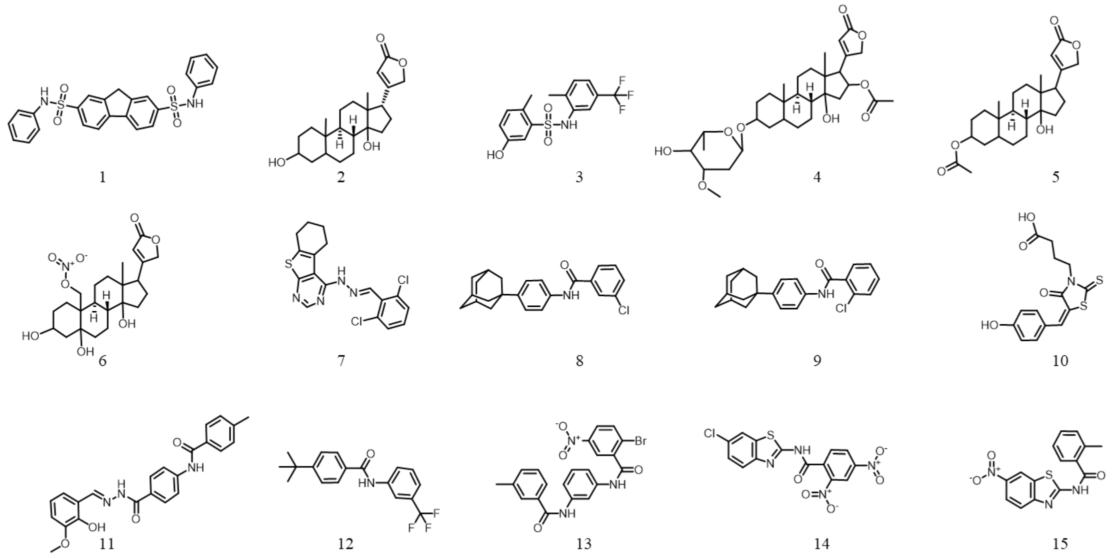

Figure S2. The structures of fifteen HIF-1α transcriptional activity inhibitors obtained from high throughput screening.

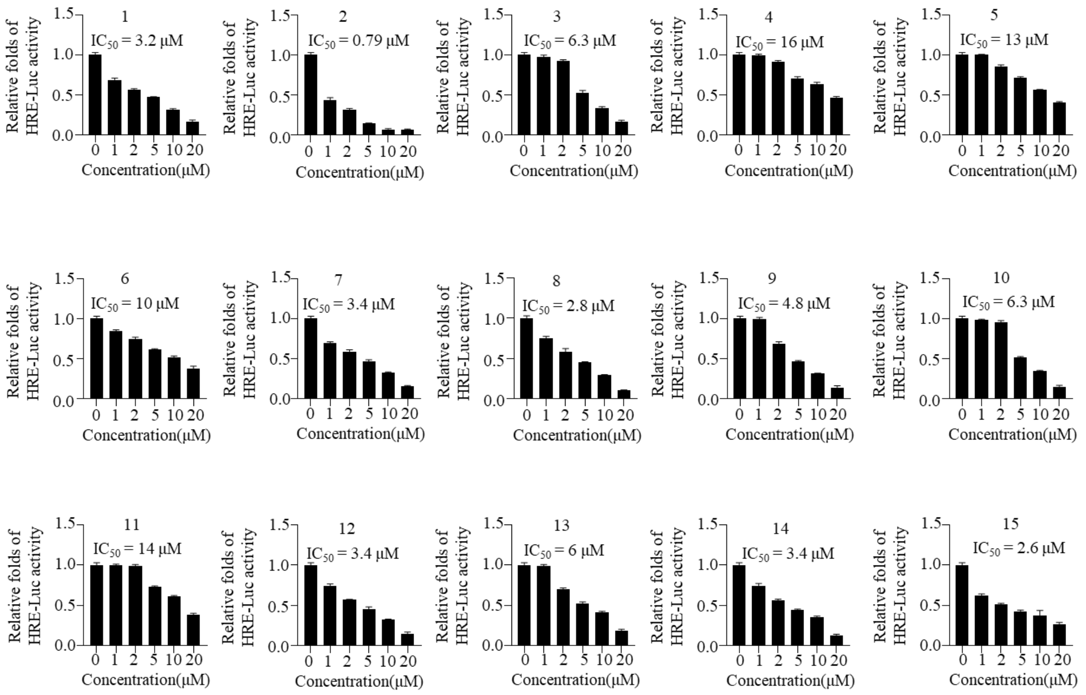

Figure S3. The activity of fifteen hit compounds on dual luciferase reporter assay in transfection system.

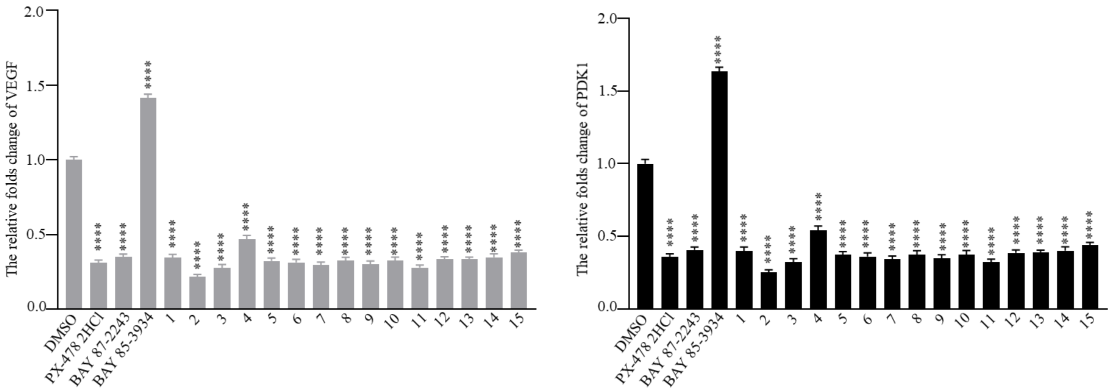

Figure S4. The activity of fifteen hit compounds on HIF-1α target genes expression in transfection system.

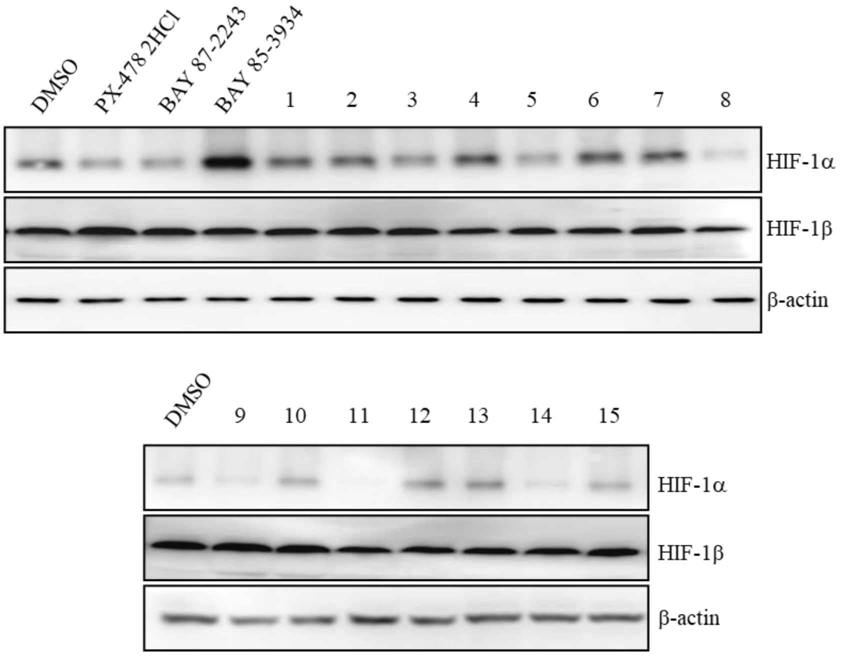

Figure S5. Protein quantification of HIF-1α with the treatment of fifteen hit compounds.

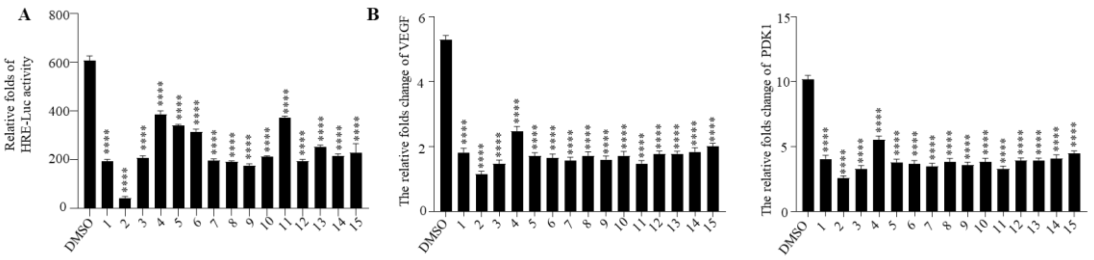

Figure S6. The activity of fifteen hit compounds on dual luciferase reporter assay and HIF-1α target genes expression under hypoxia condition.

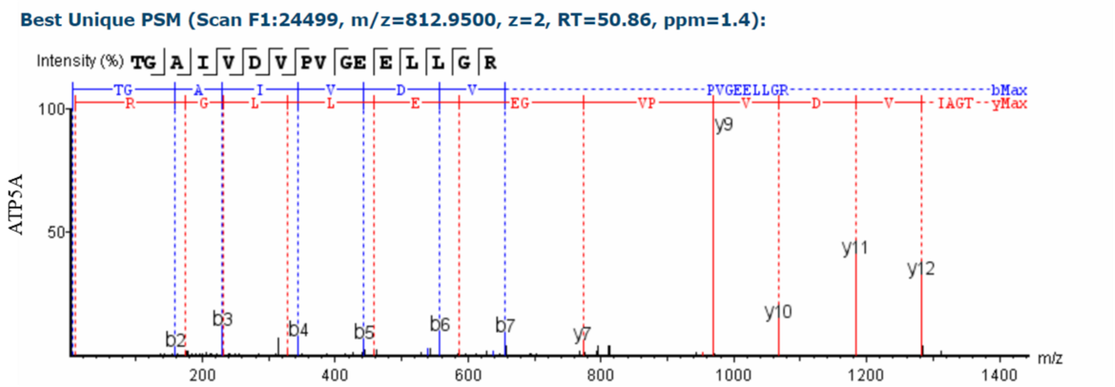

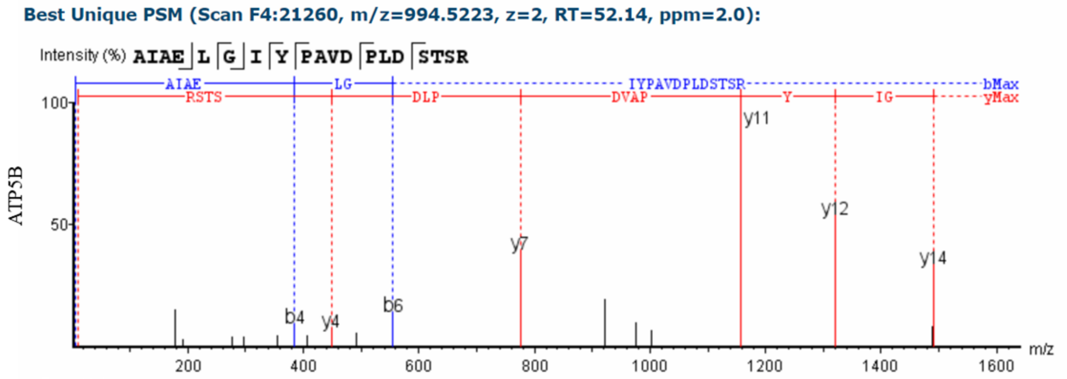

Figure S7. Mass spectrometry analysis of pull-down protein by **HI-102** from HEK293T cells.

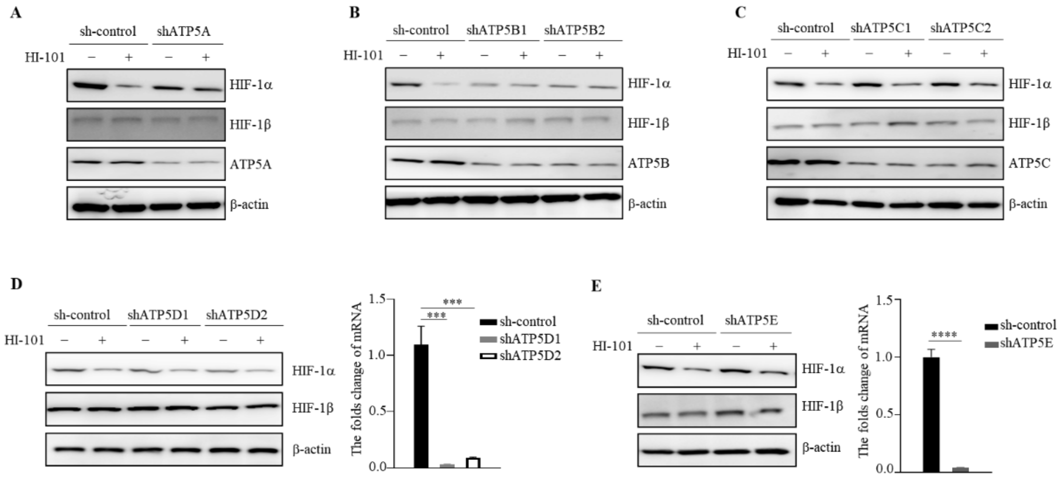

Figure S8. Protein quantification of HIF-1α under **HI-101** treatment (10 μM) in cell lines with different ATP synthase subunits knocked down separately (A) ATP5A (B) ATP5B (C) ATP5C (D) ATP5D (E) ATP5E

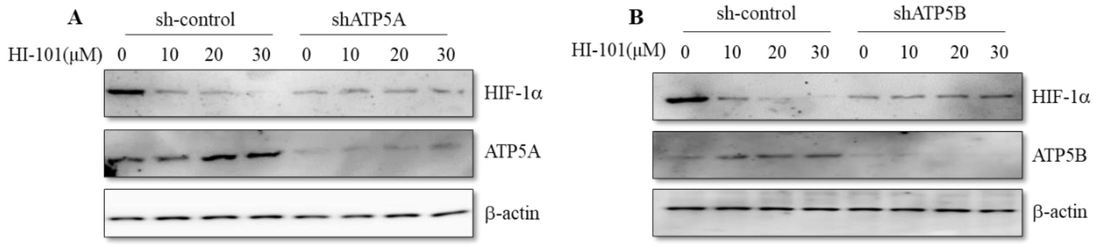

Figure S9. Protein quantification of HIF-1α under **HI-101** treatment at indicated concentrations in cell lines with ATP5A (A) or ATP5B (B) knocked down separately.

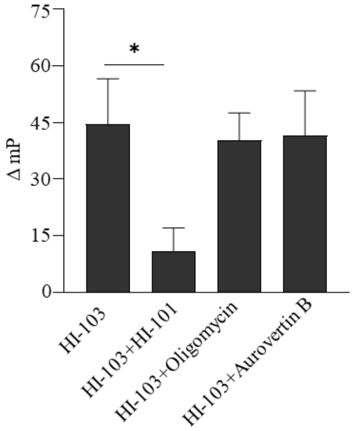

Figure S10. Competitive fluorescence polarization assay. F_1-ATPase at 30 nM was incubated with compounds at indicated concentrations (**HI-103** 4 nM; **HI-101** 1μM; **Oligomycin** 1μM; **Aurovertin B** 1μM).

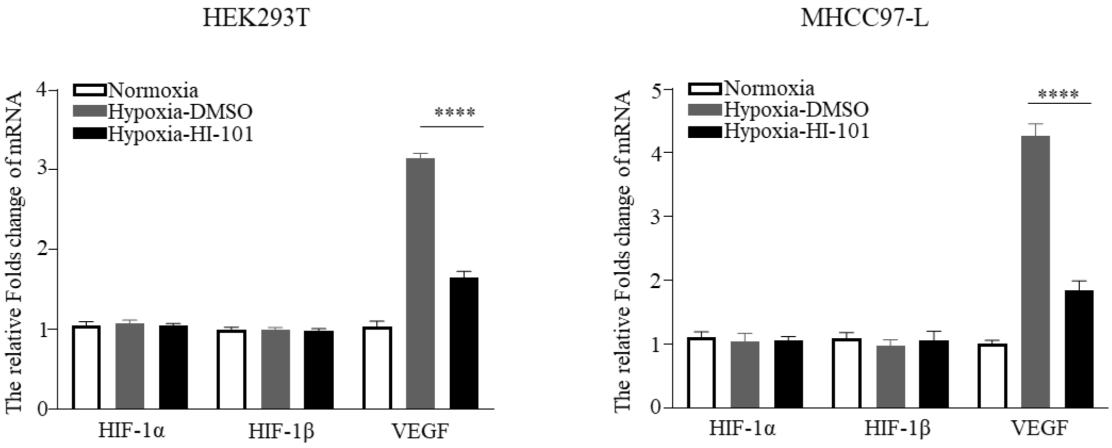

Figure S11. **HI-101** reduced VEGF transcript level with no effect on HIF-1α and HIF-1β transcript level.

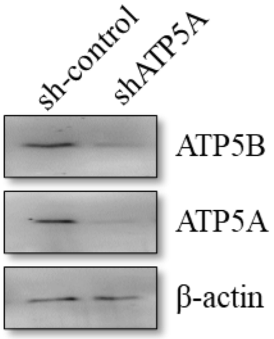

Figure S12. Western blot analysis of ATP5B protein expression in ATP5A knock-down HEK293T cells.

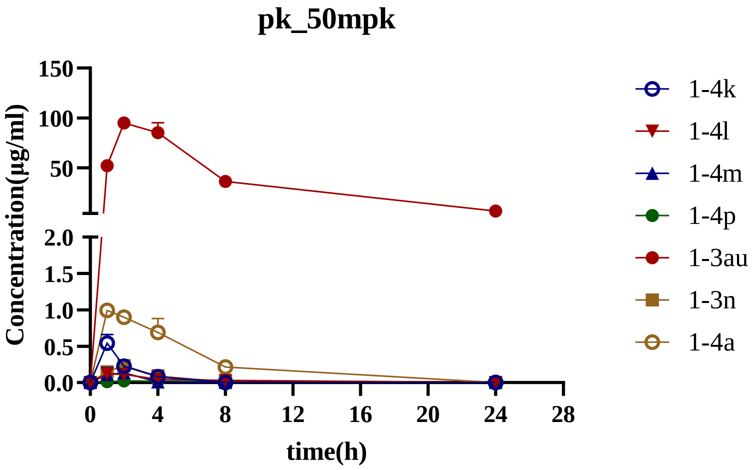

Figure S13. Plasma concentration–time curves of seven selected **HI** compounds in ICR

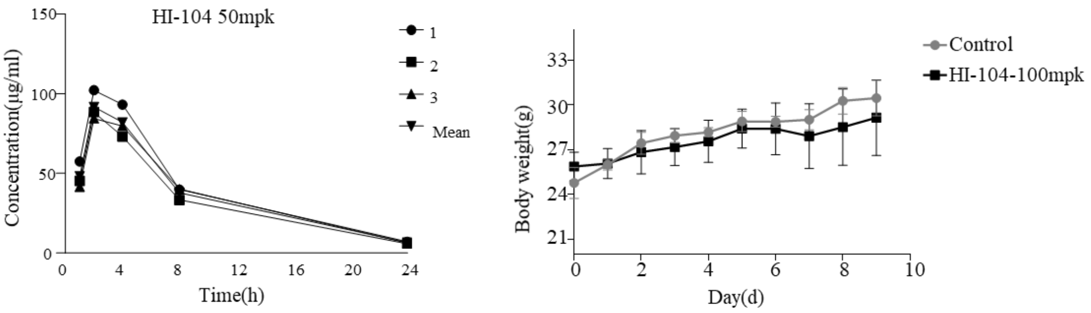

Figure S14. Plasma concentration–time curve and toxicity test of **HI-104** in ICR.

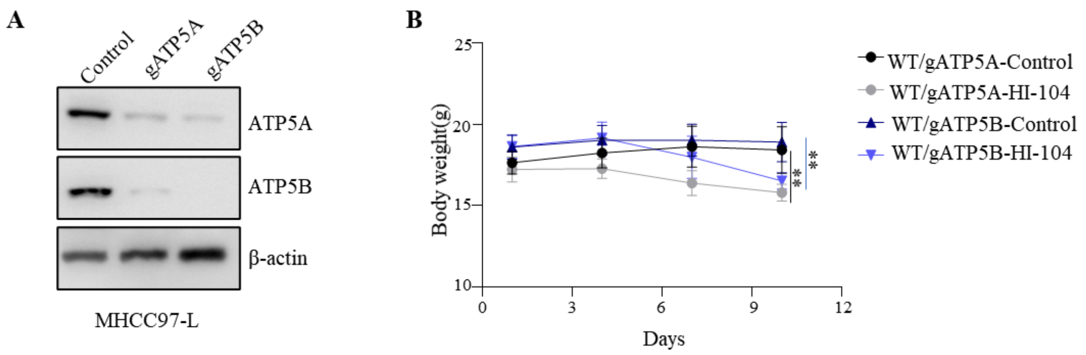

Figure S15. Construction of ATP5A or ATP5B knockout stable MHCC97-L cell line (A)Body weight of nude mice after treatment with DMSO or **HI-104** in MHCC97-L xenograft model (B)

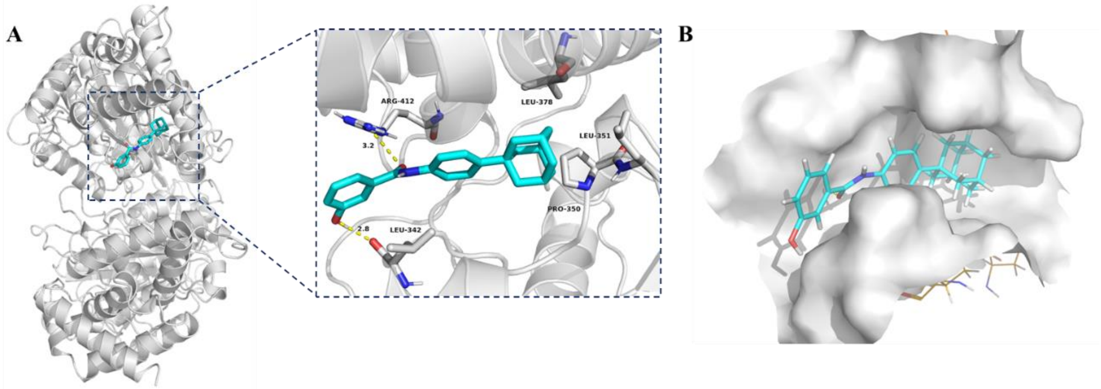

Figure S16. Binding mode of compound **HI-105**. (A) Binding mode of **HI-105** with ATP5B (PDB: 1COW) revealed by molecular docking. Ligand was shown in the cyan stick model. Key residues were shown in the gray stick model. (B) The surface view of ATP5B docked with **HI-105**.

2.The specific synthesis routes

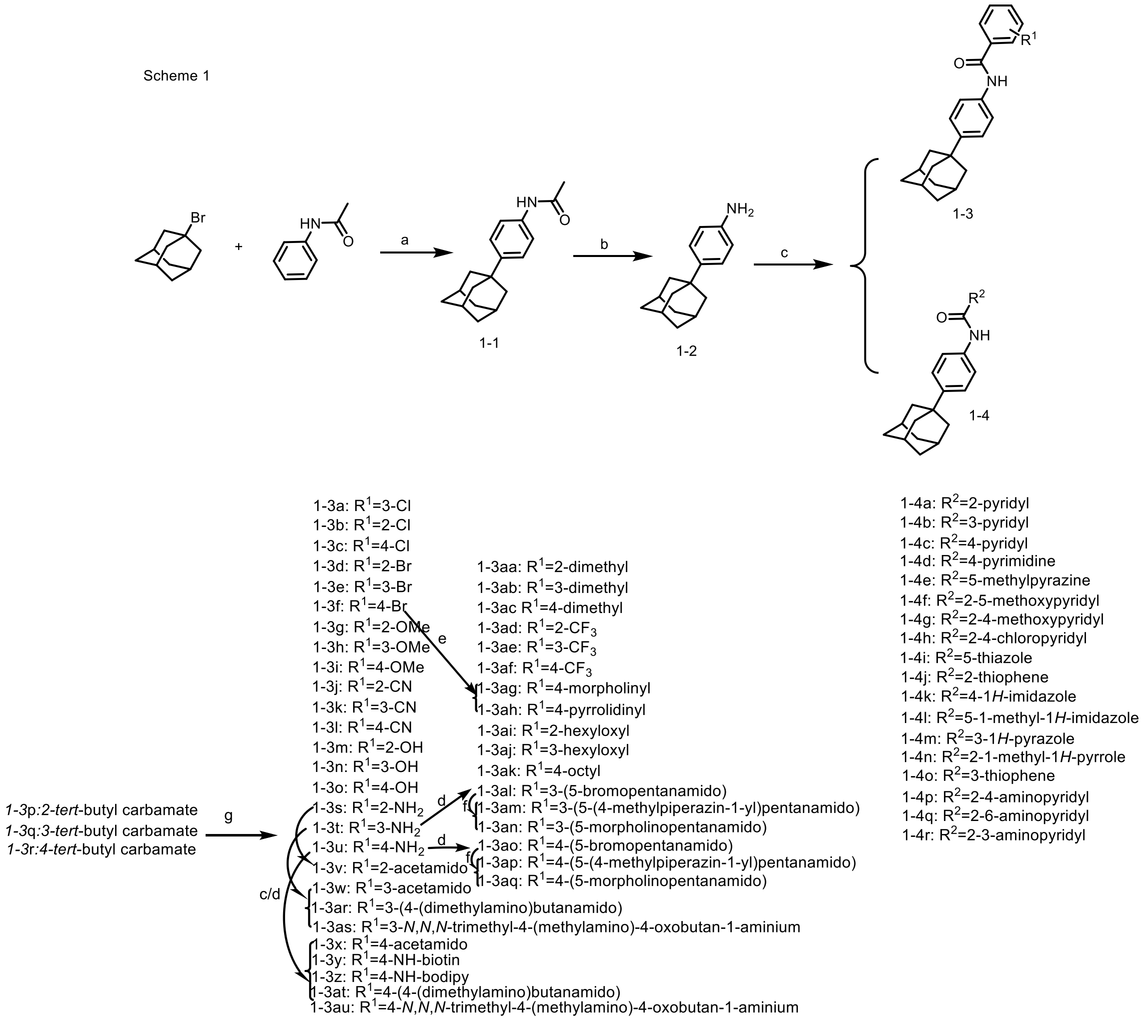

Scheme 1. Synthesis routes to class A compounds

Scheme 1: Reagents and conditions: (a) $AlCl_3$, 140℃, 40-48h; (b) Con HCl, MeOH, reflux,20-30h; (c) HATU, DIPEA, DMF, RT, 5-12h; (d) DCM, triethylamine, RT, overnight; (e) $Pd_2(dba)_3$, BINAP, NaOBu-t, dioxane, 90℃; (f) K_2CO_3, KI, DMF, 80℃; (g) DCM, TFA, RT, 5-12h.

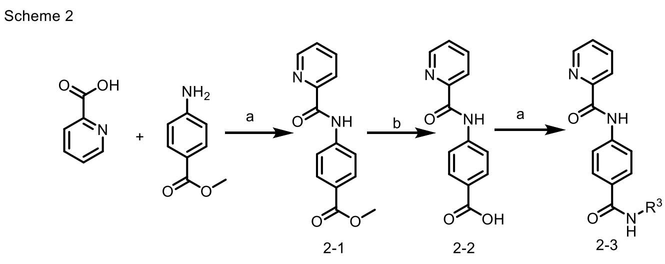

Scheme 2: Synthesis routes to class B compounds

Scheme 2：Reagents and conditions: (a) HATU, DIPEA, DMF, RT, corresponding amino; (b) 2M NaOH, MeOH, 50℃

Scheme3

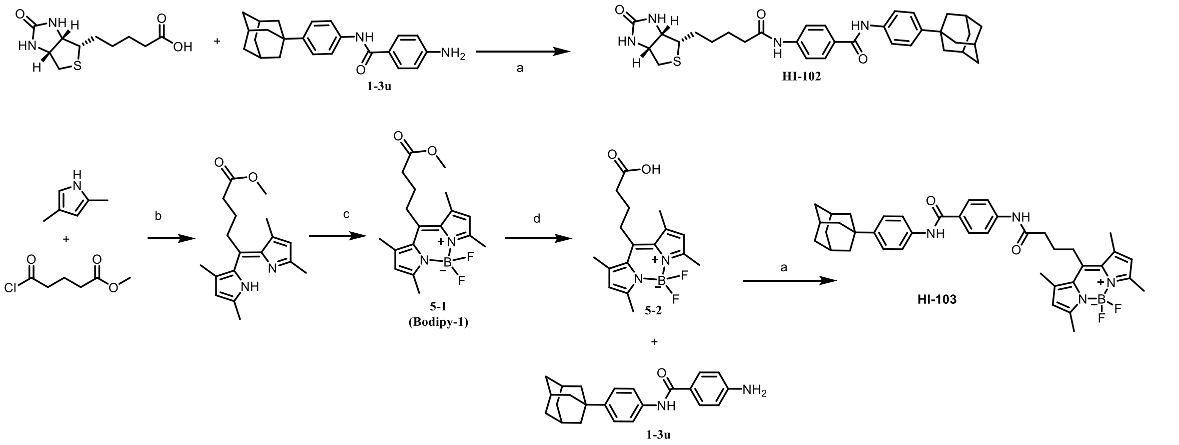

Scheme3: Synthesis routes to probes **HI-102** and **HI-103**

Reagents and conditions: (a) HATU, DIPEA, DMF, RT, 5-12h; (b) N_2, 0℃, 30min; RT, 3h; RT - 0℃ (c)Et_3N, 30min, 0℃; $BF_3 \cdot OEt_2$, 0℃-RT; (d) LiOH, MeOH, 75℃.

3. ^{1}H NMR and ^{13}C NMR

*N-(4-((3r,5r,7r)-adamantan-1-yl)phenyl)-3-chlorobenzamide***(HI-101, 1-3a)**

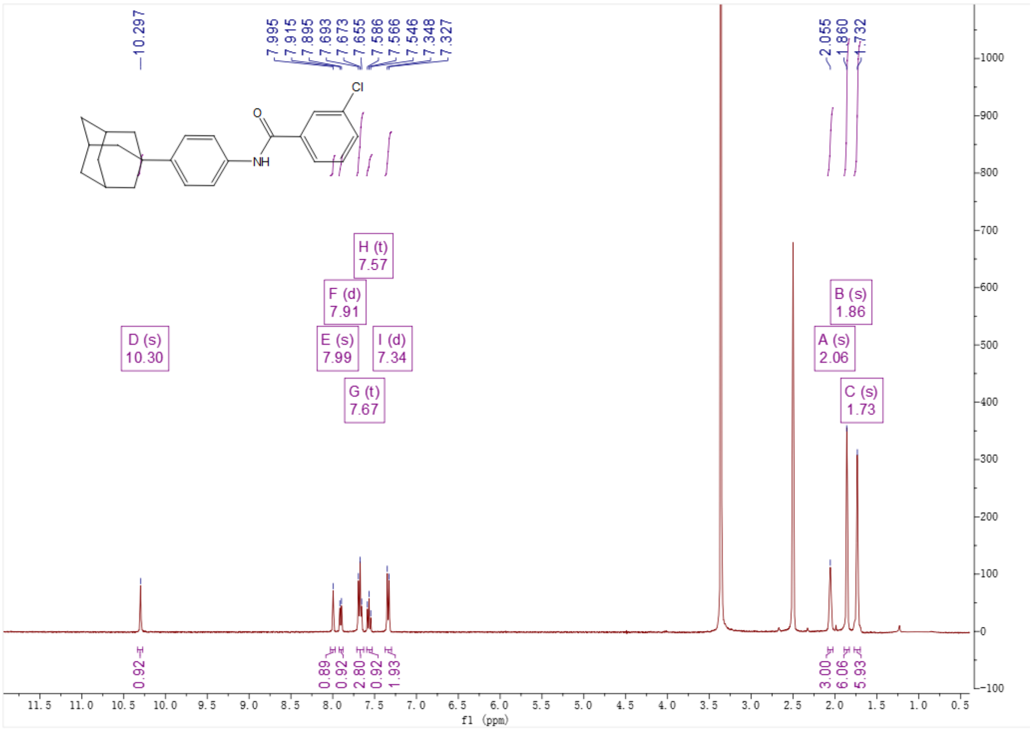

^{1}H NMR

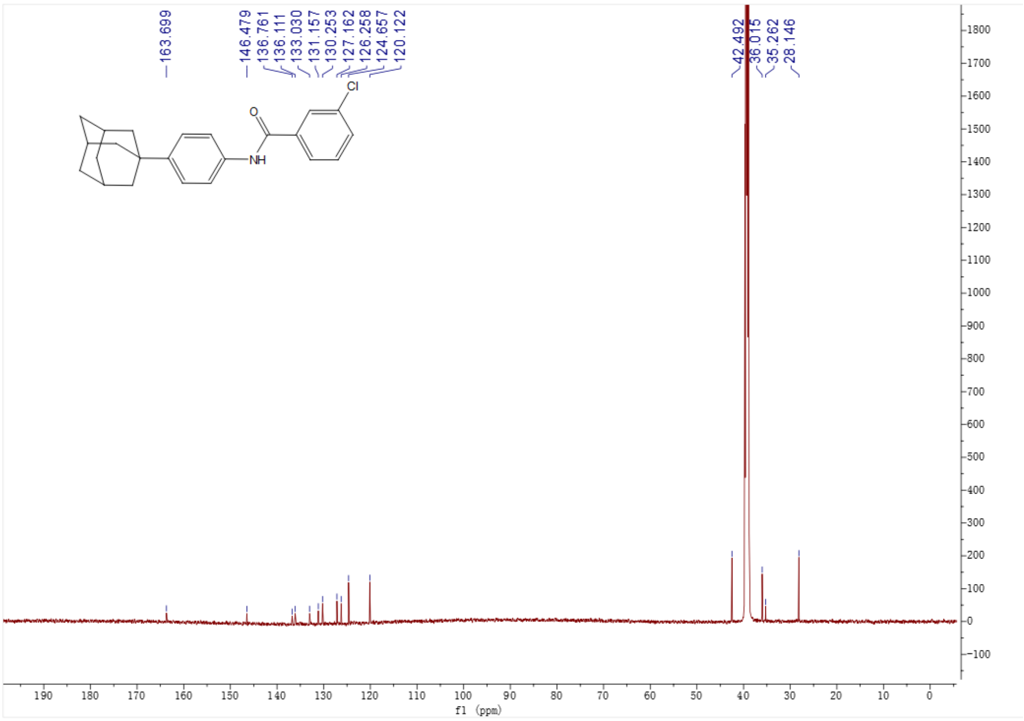

^{13}C NMR

*N-(4-((3r,5r,7r)-adamantan-1-yl)phenyl)-2-chlorobenzamide(**1-3b**)*

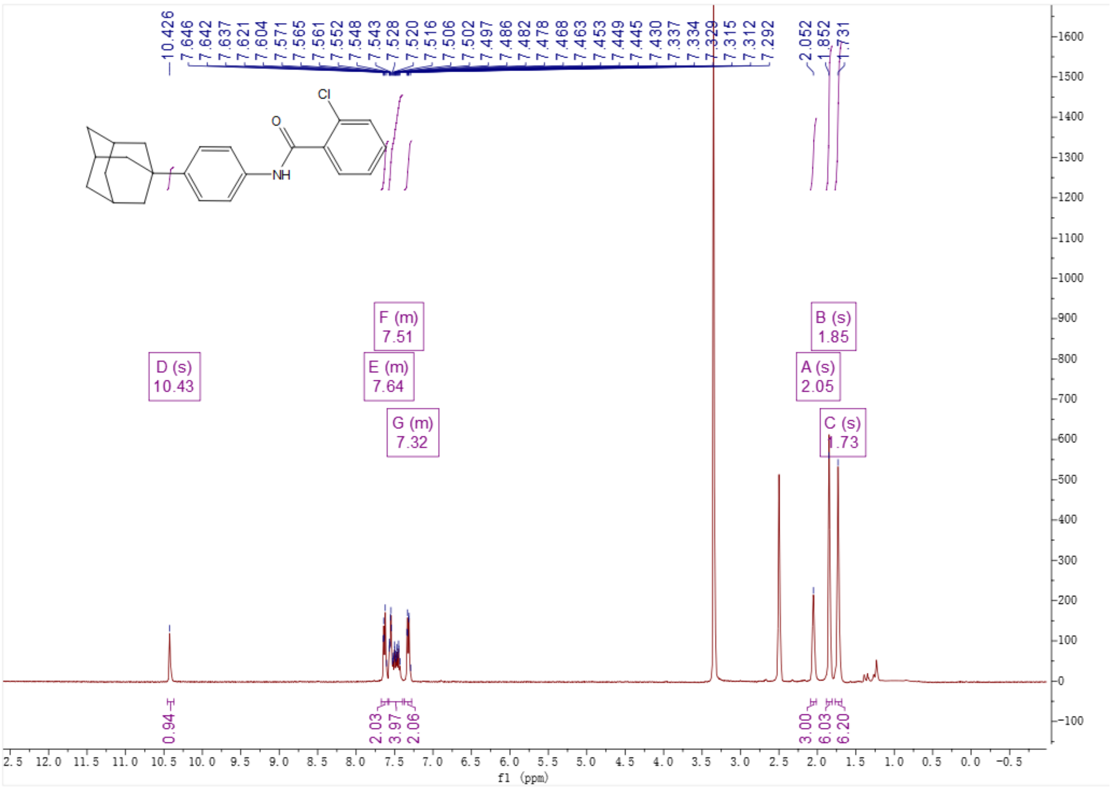

^{1}H NMR

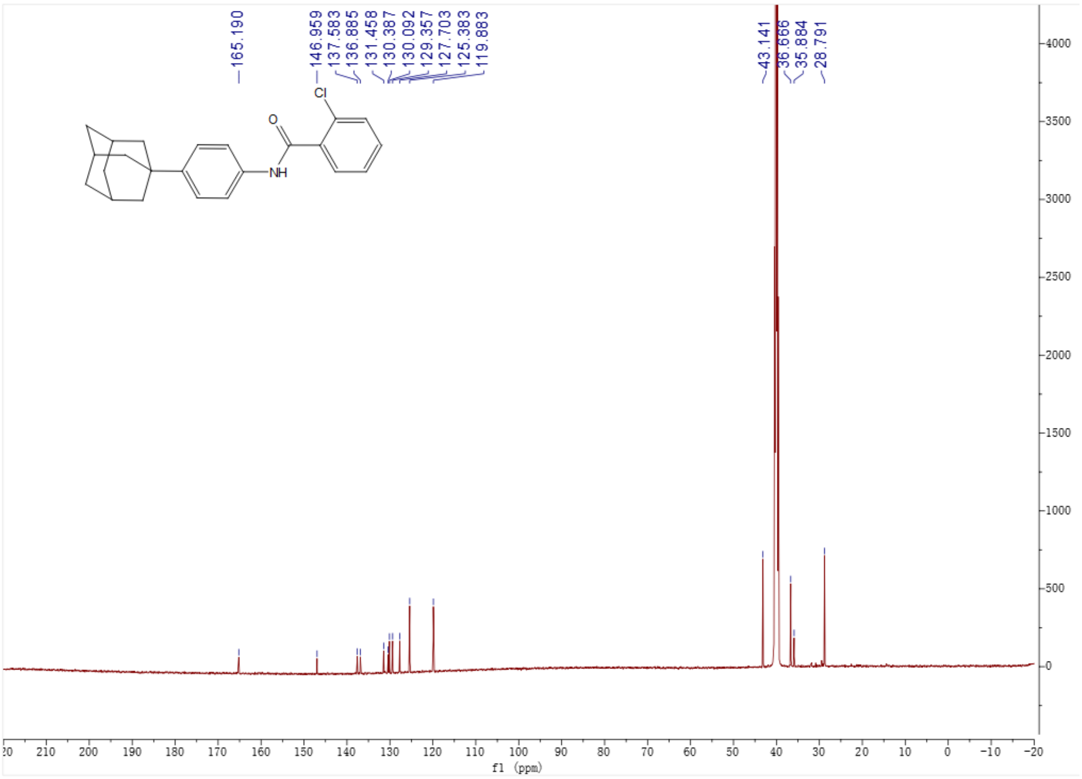

^{13}C NMR

*N-(4-((3r,5r,7r)-adamantan-1-yl)phenyl)-4-chlorobenzamide**(1-3c)***

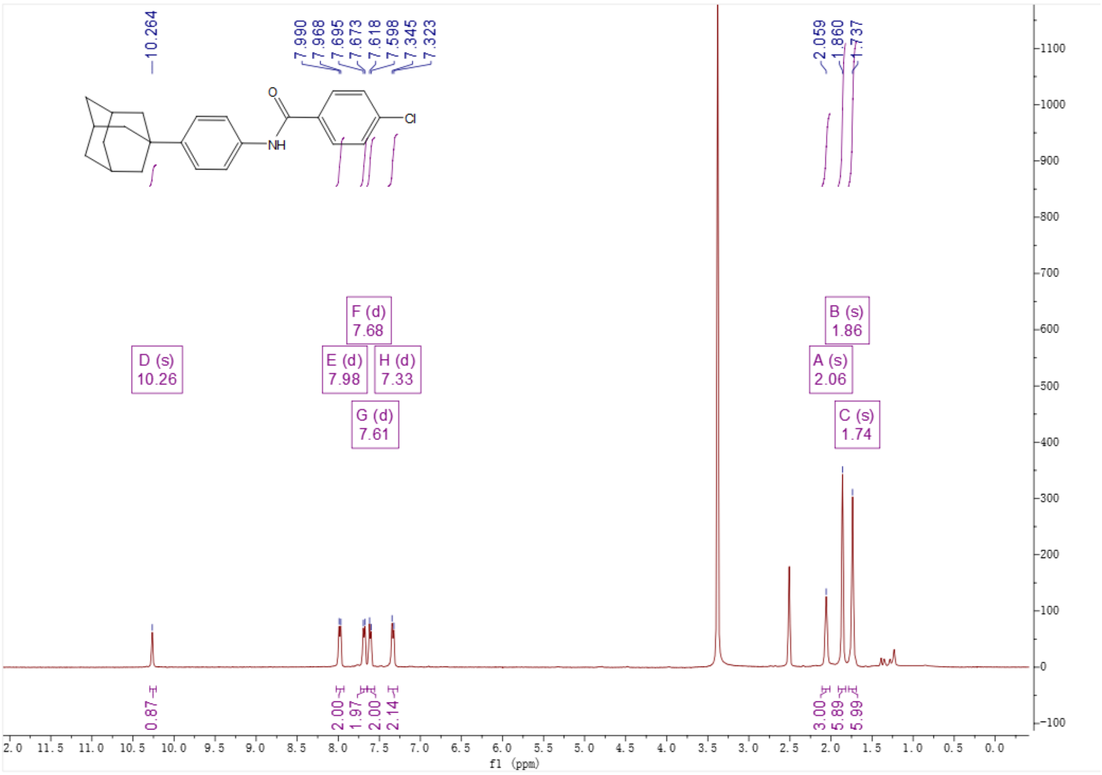

^{1}H NMR

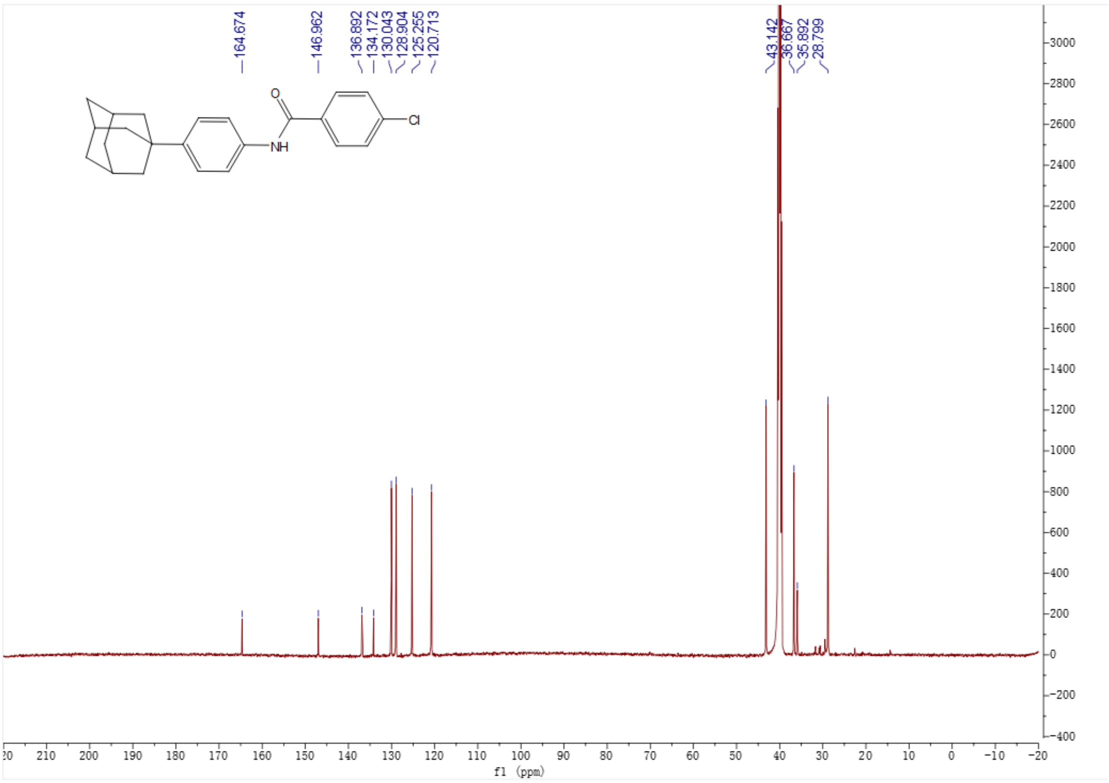

^{13}C NMR

*N-(4-((3r,5r,7r)-adamantan-1-yl)phenyl)-2-bromobenzamide**(1-3d)***

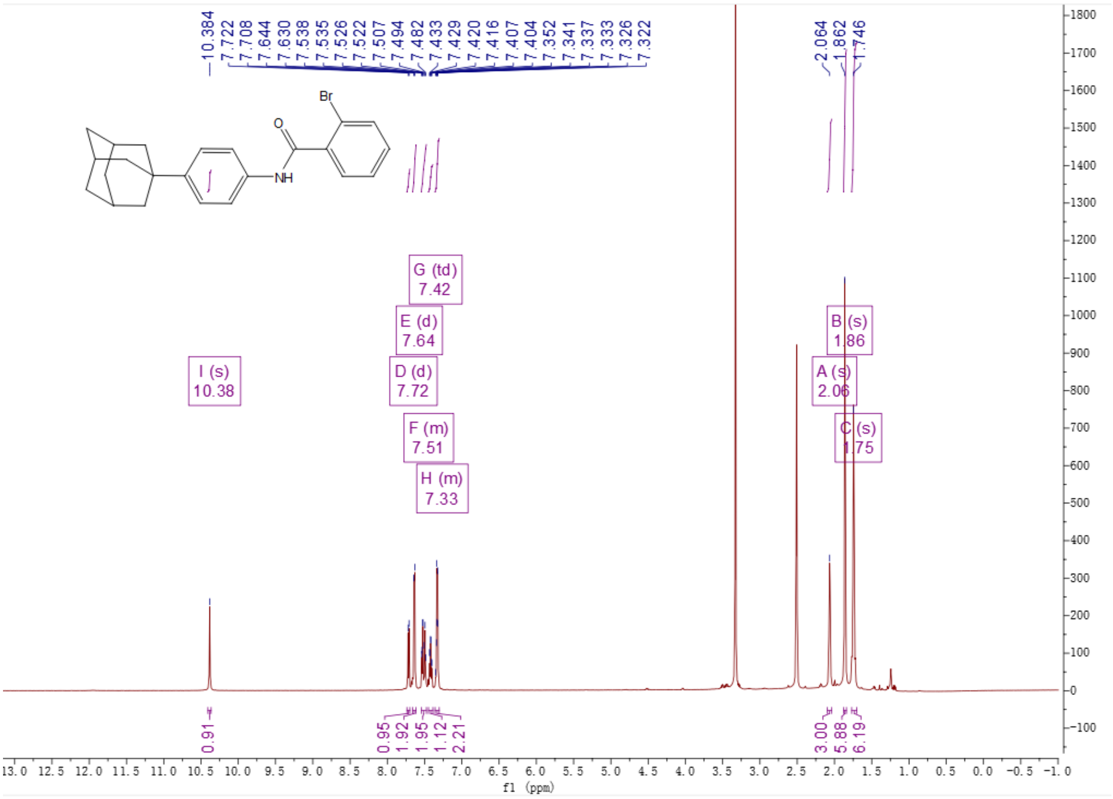

^{1}H NMR

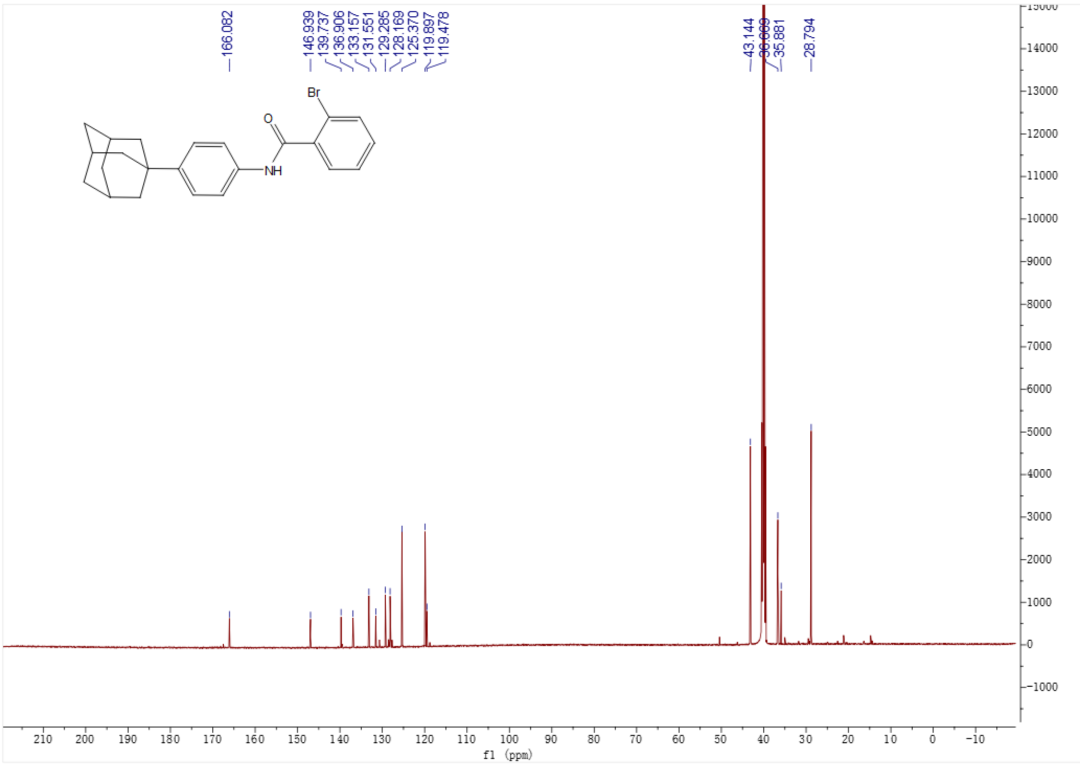

^{13}C NMR

*N-(4-((3r,5r,7r)-adamantan-1-yl)phenyl)-3-bromobenzamide(**1-3e**)*

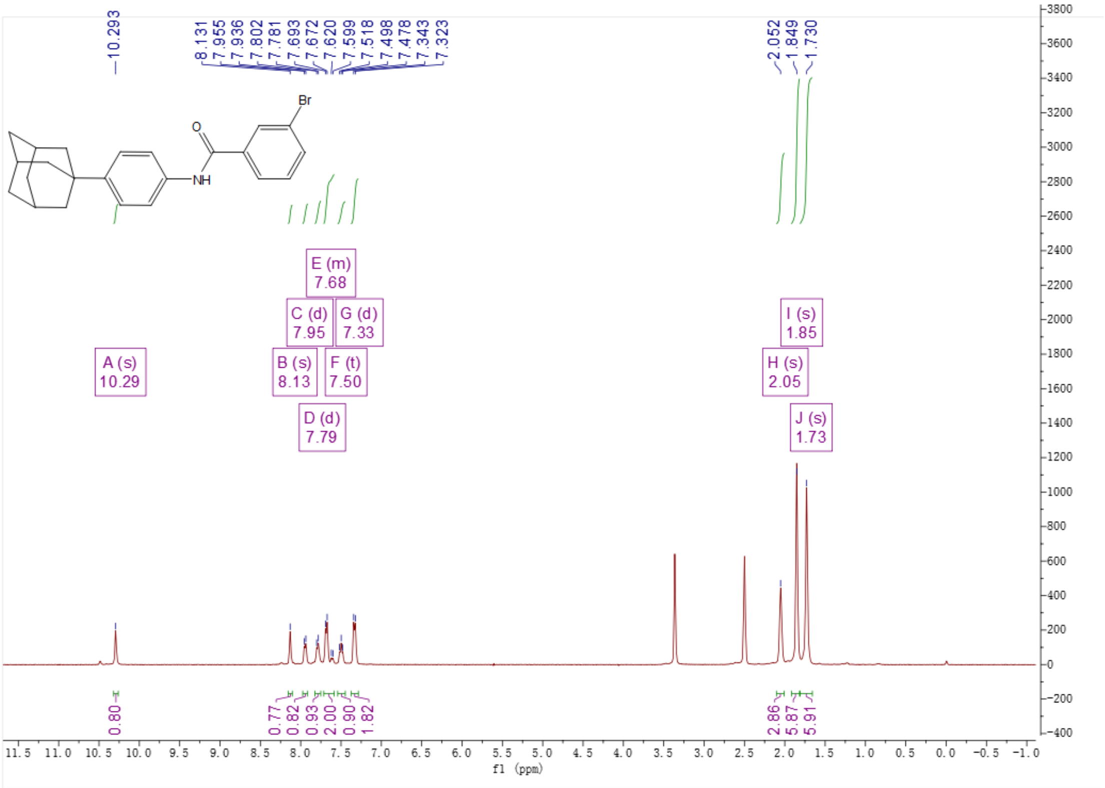

^{1}H NMR

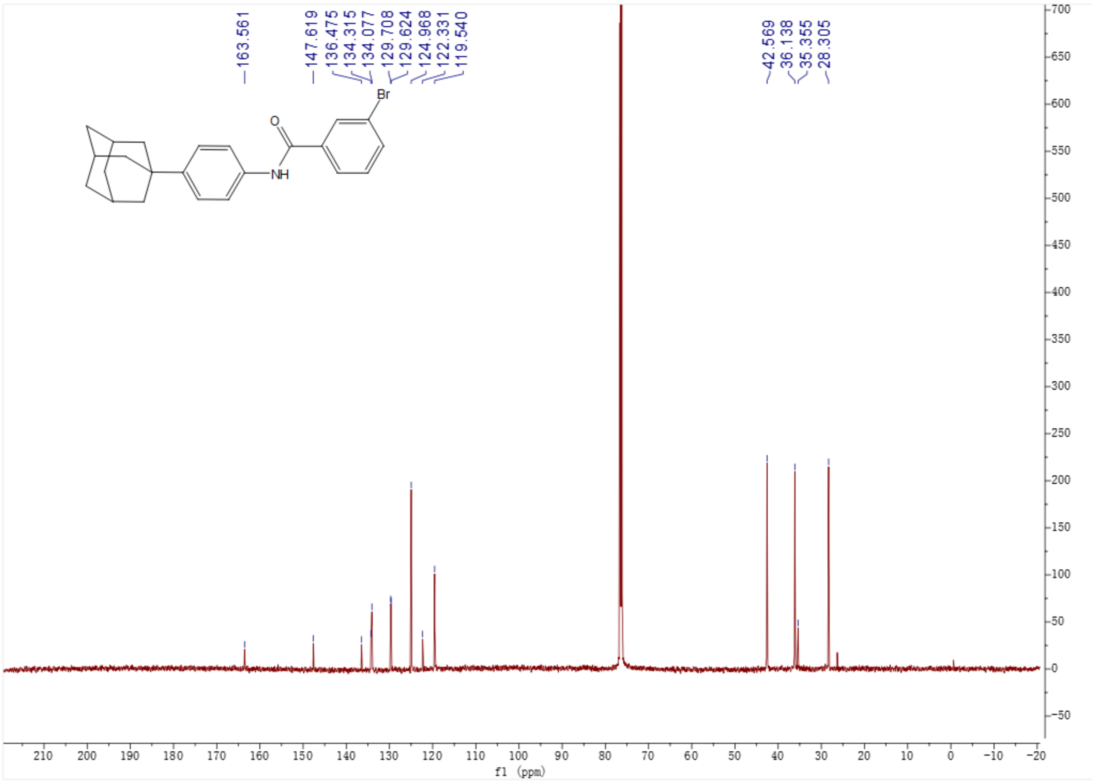

^{13}C NMR

*N-(4-((3r,5r,7r)-adamantan-1-yl)phenyl)-4-bromobenzamide(**1-3f**)*

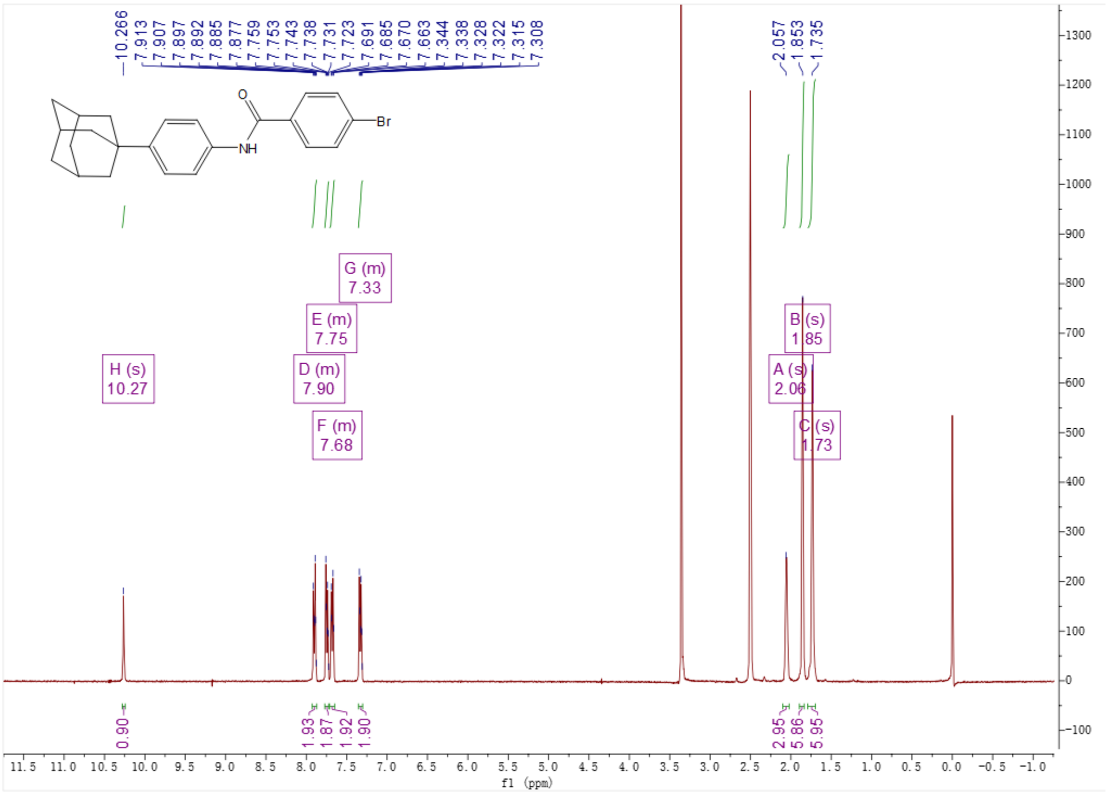

^{1}H NMR

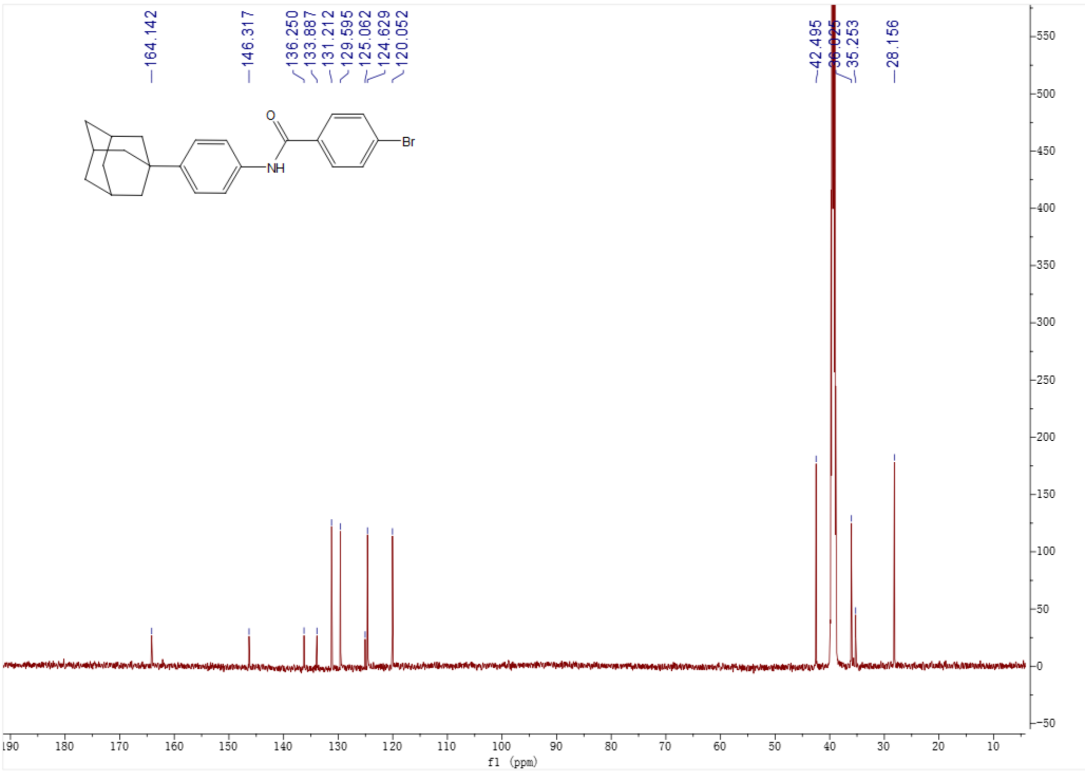

^{13}C NMR

*N-(4-((3r,5r,7r)-adamantan-1-yl)phenyl)-2-methoxybenzamide(**1-3g**)*

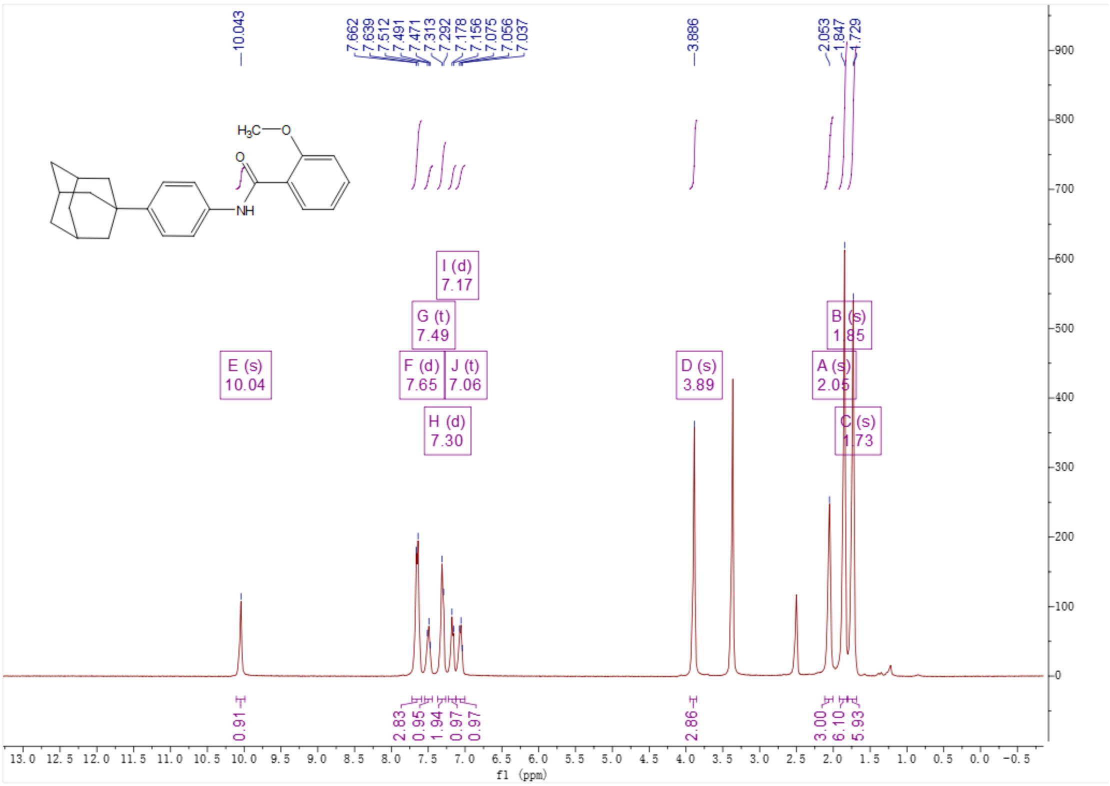

^{1}H NMR

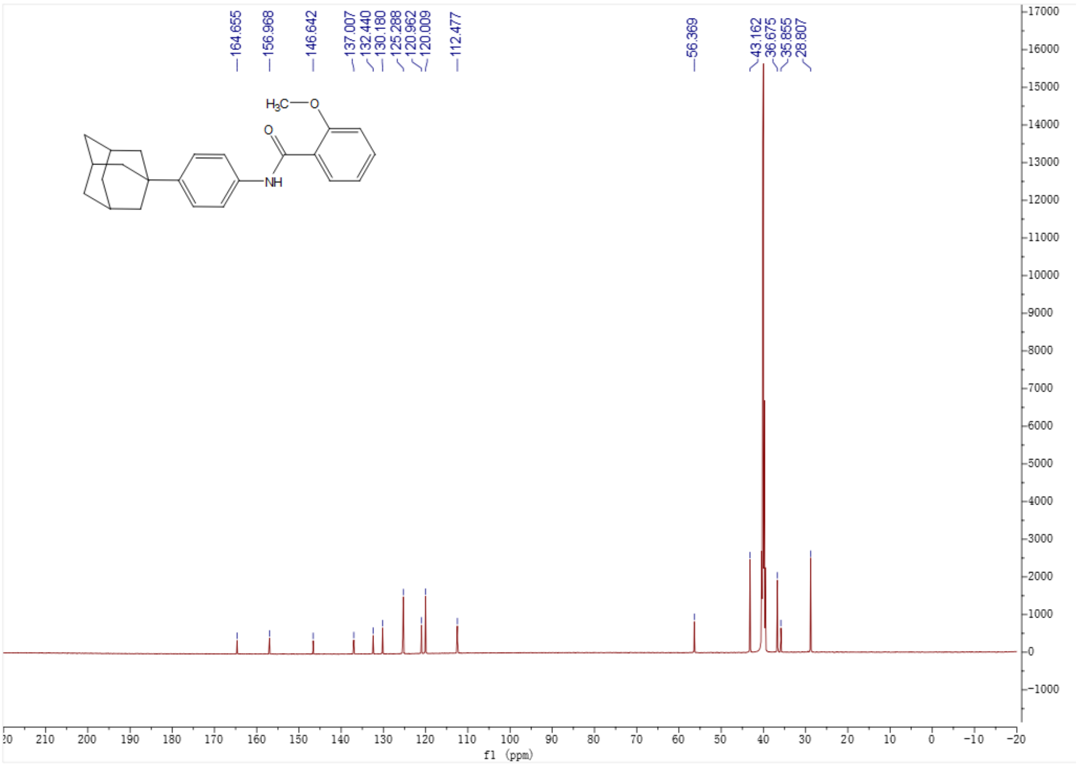

^{13}C NMR

*N-(4-((3r,5r,7r)-adamantan-1-yl)phenyl)-3-methoxybenzamide**(1-3h)***

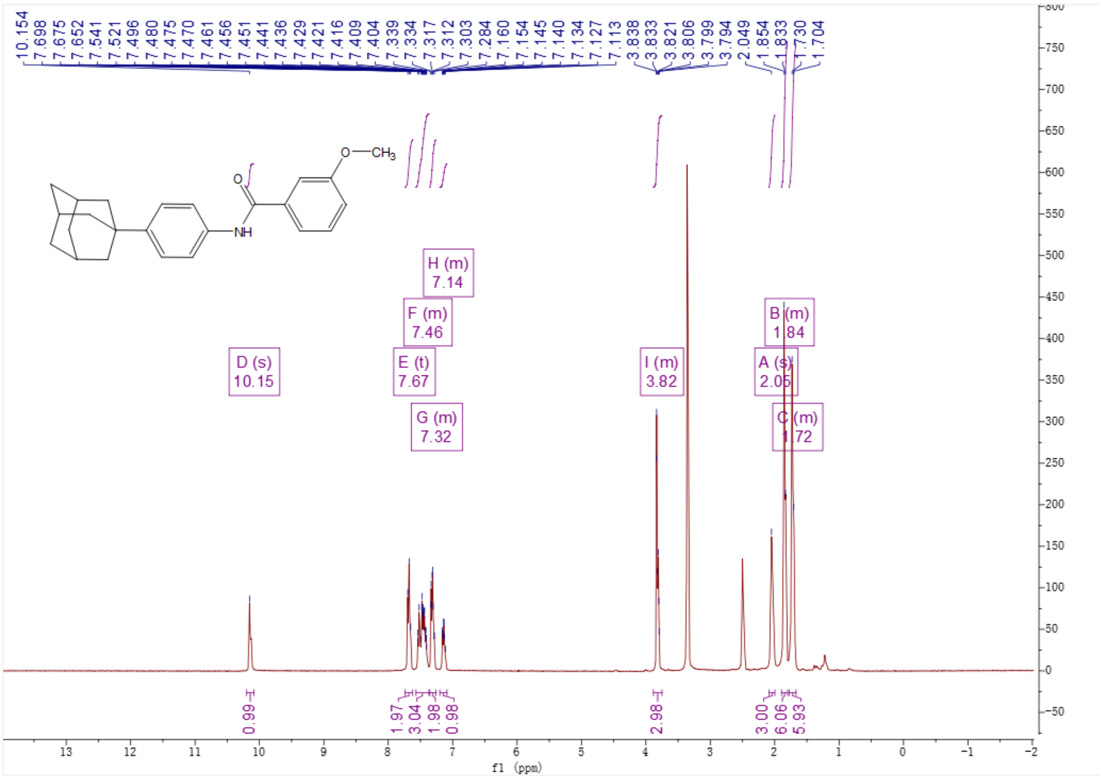

^{1}H NMR

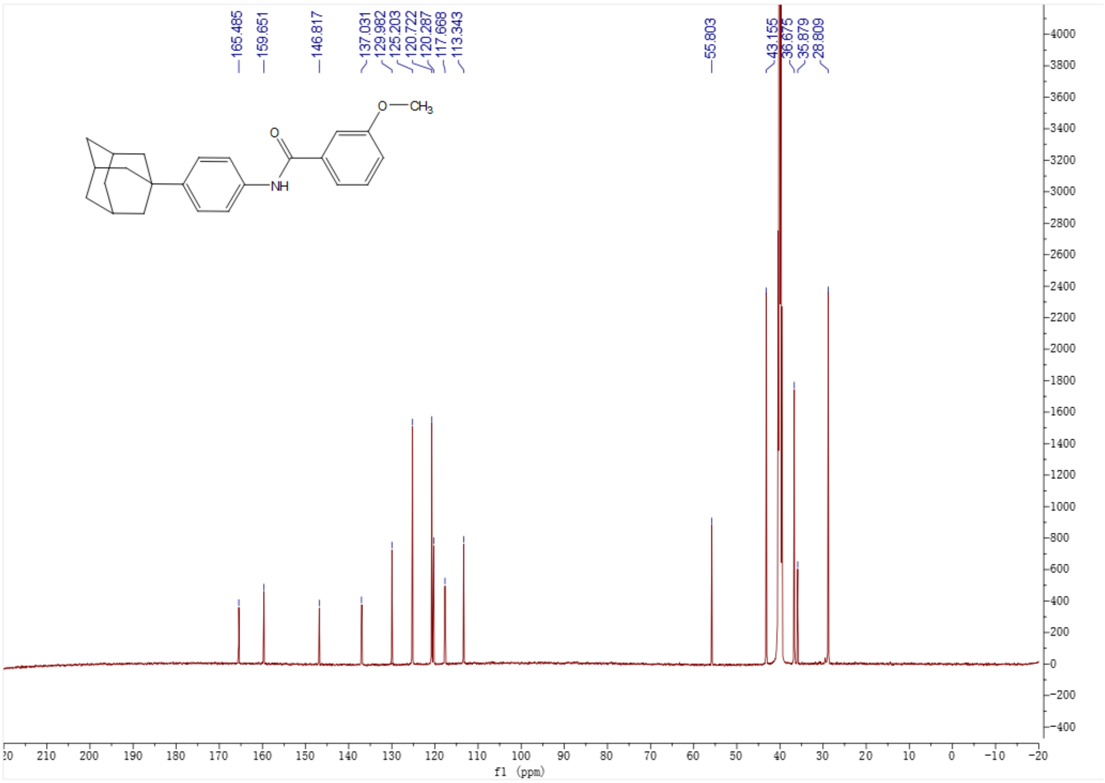

^{13}C NMR

*N-(4-((3r,5r,7r)-adamantan-1-yl)phenyl)-4-methoxybenzamide**(1-3i)***

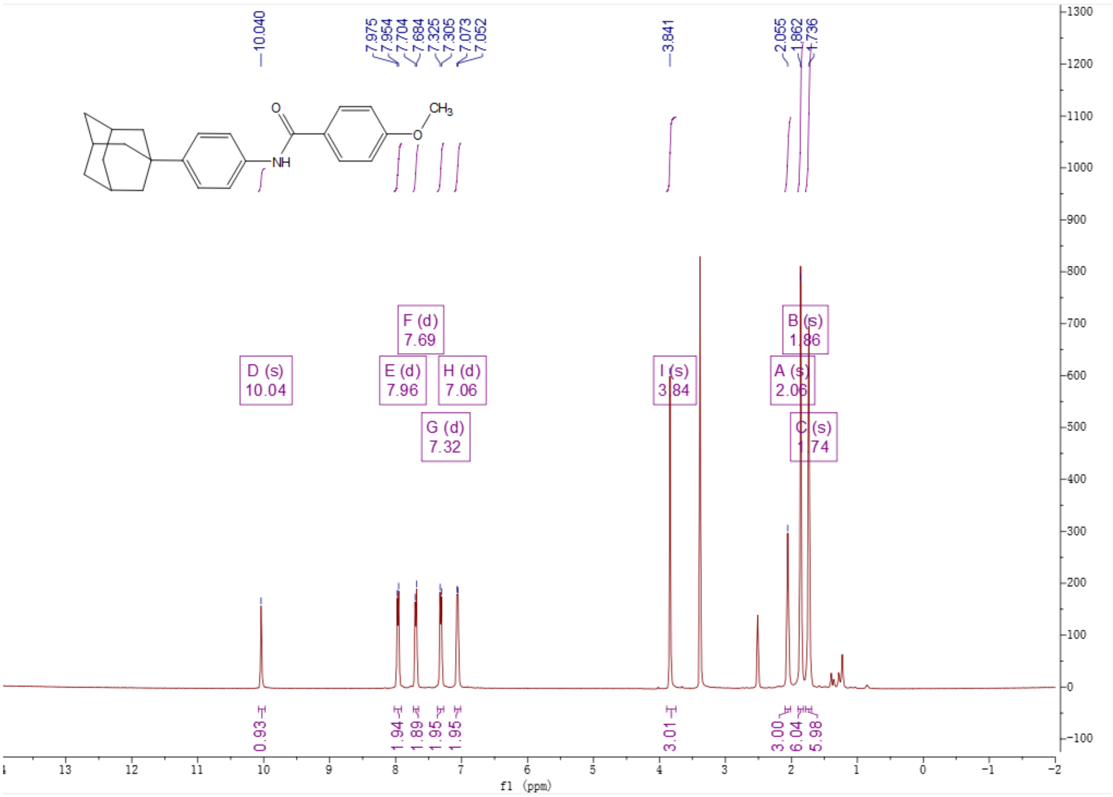

^{1}H NMR

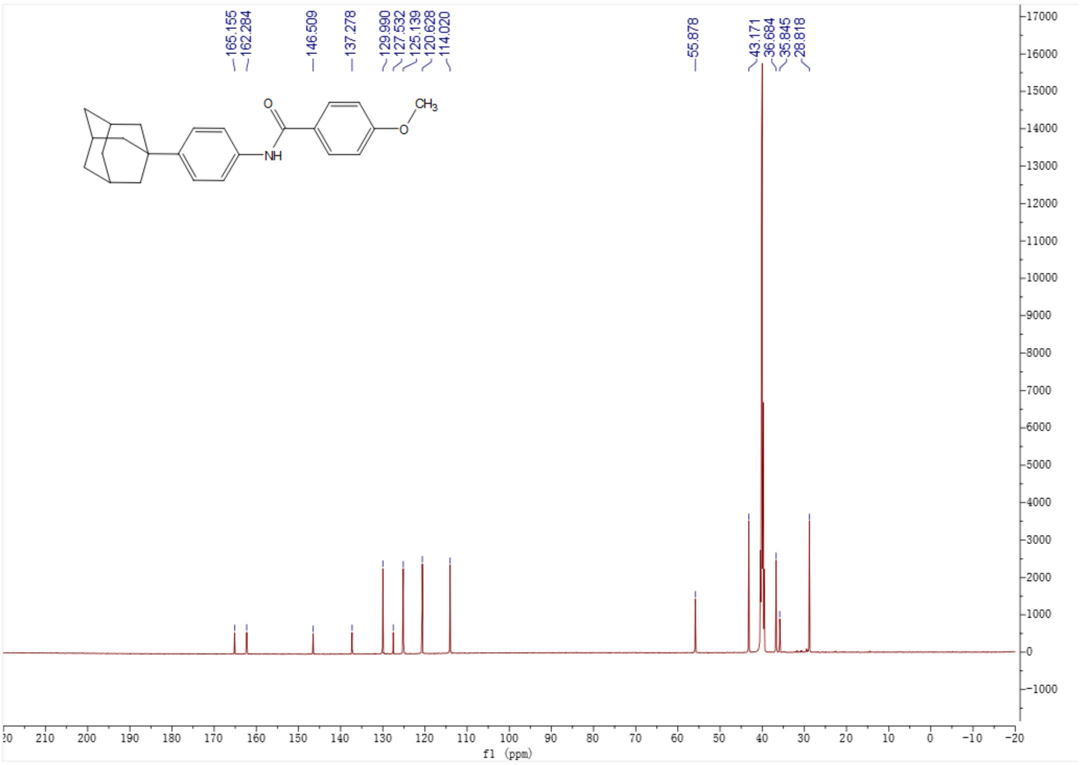

^{13}C NMR

*N-(4-((3r,5r,7r)-adamantan-1-yl)phenyl)-2-cyanobenzamide(**1-3j**)*

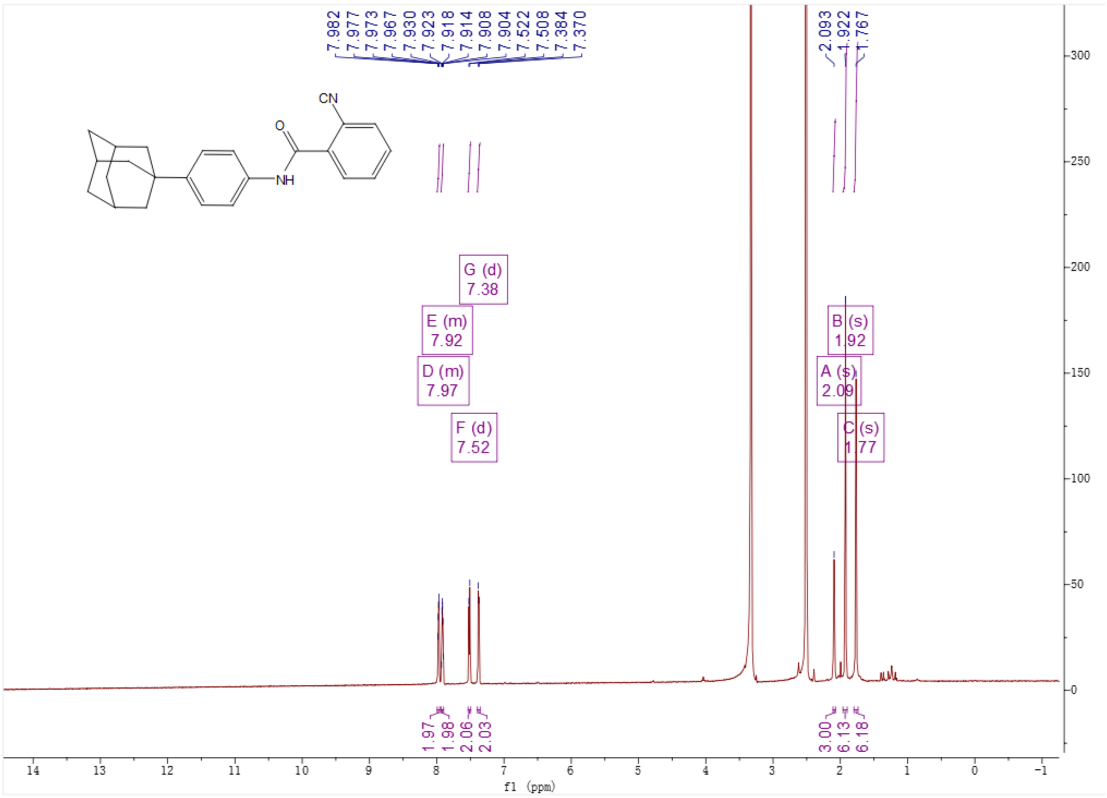

^{1}H NMR

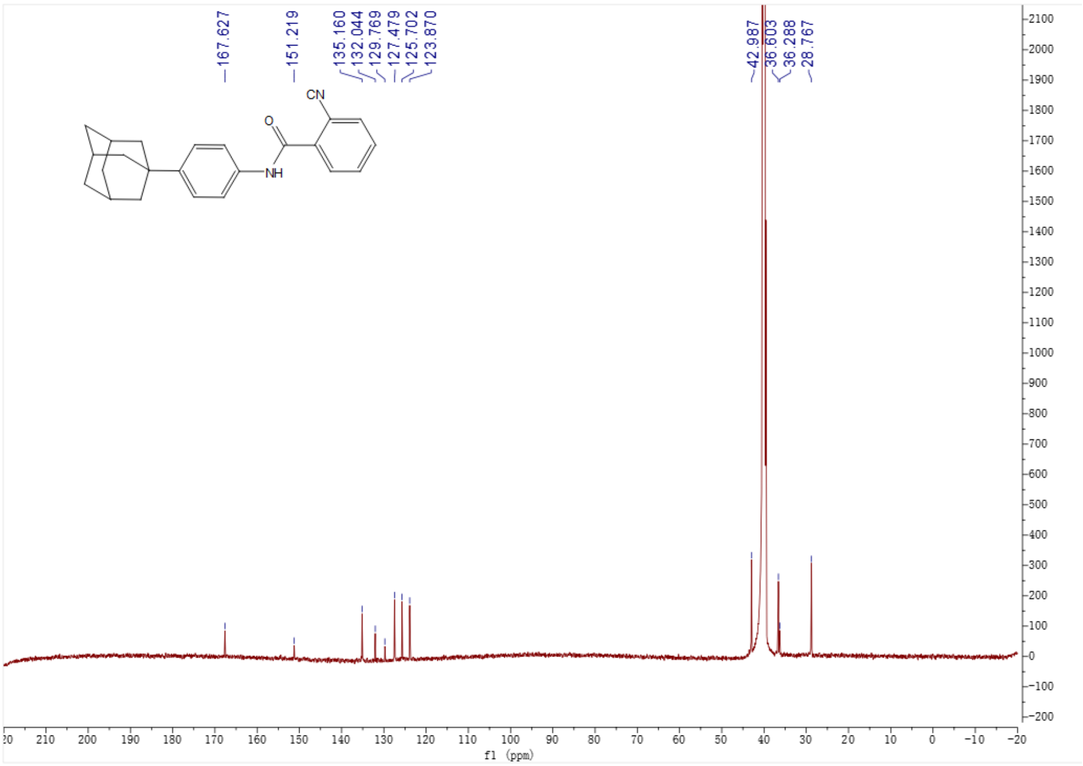

^{13}C NMR

*N-(4-((3r,5r,7r)-adamantan-1-yl)phenyl)-3-cyanobenzamide(**1-3k**)*

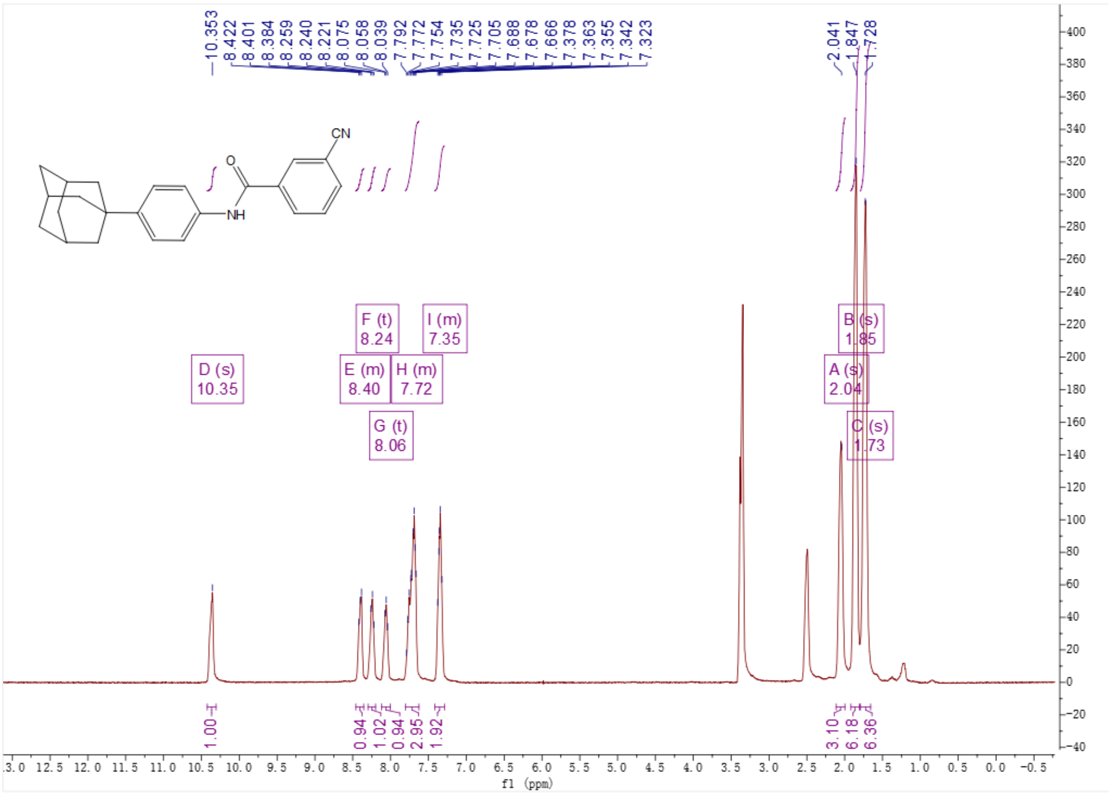

^{1}H NMR

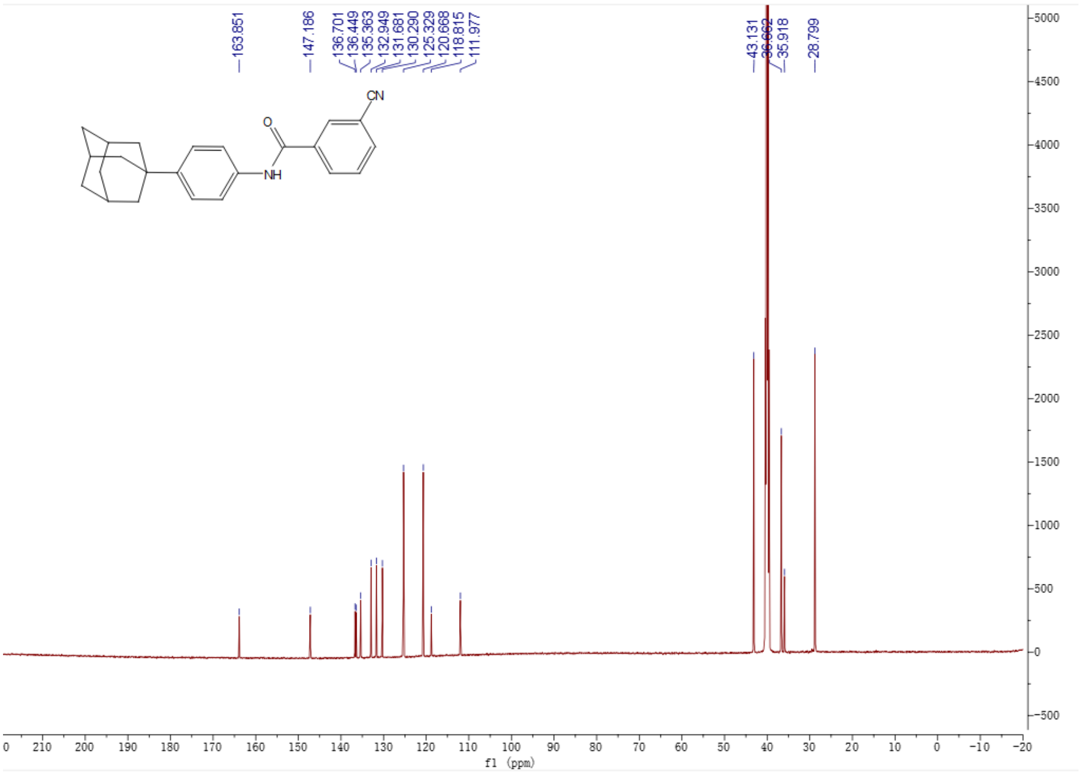

^{13}C NMR

*N-(4-((3r,5r,7r)-adamantan-1-yl)phenyl)-4-cyanobenzamide(**1-3l**)*

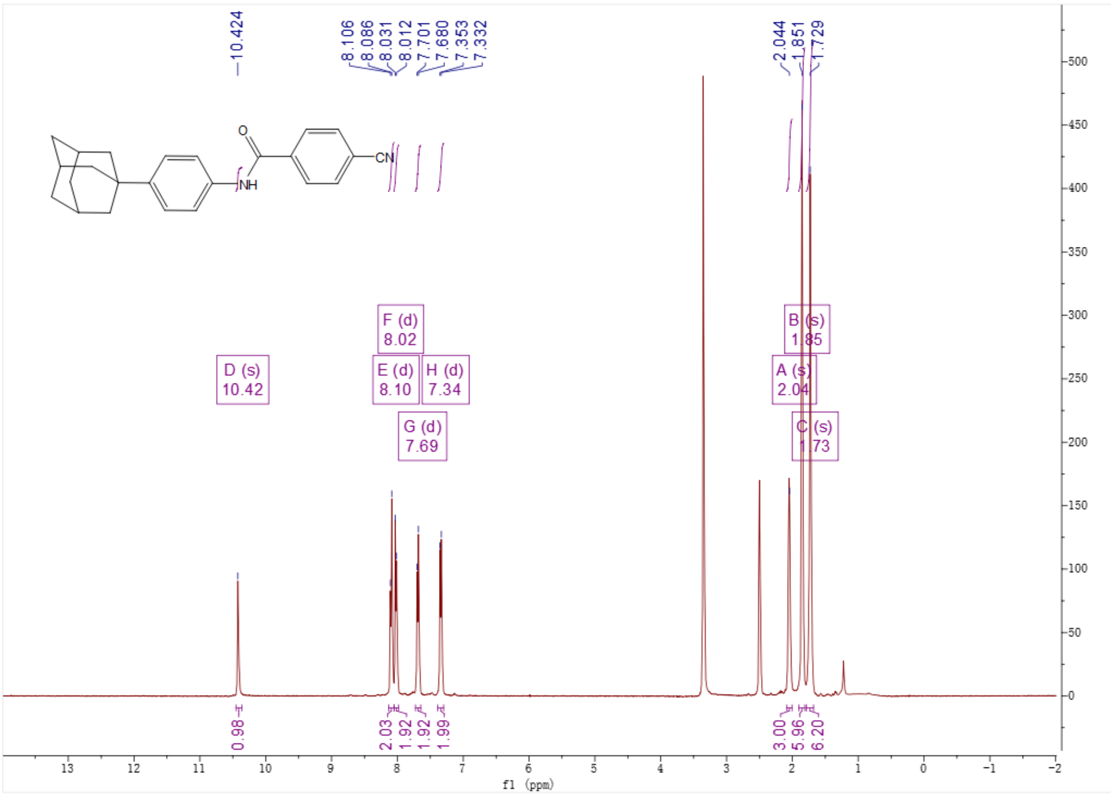

^{1}H NMR

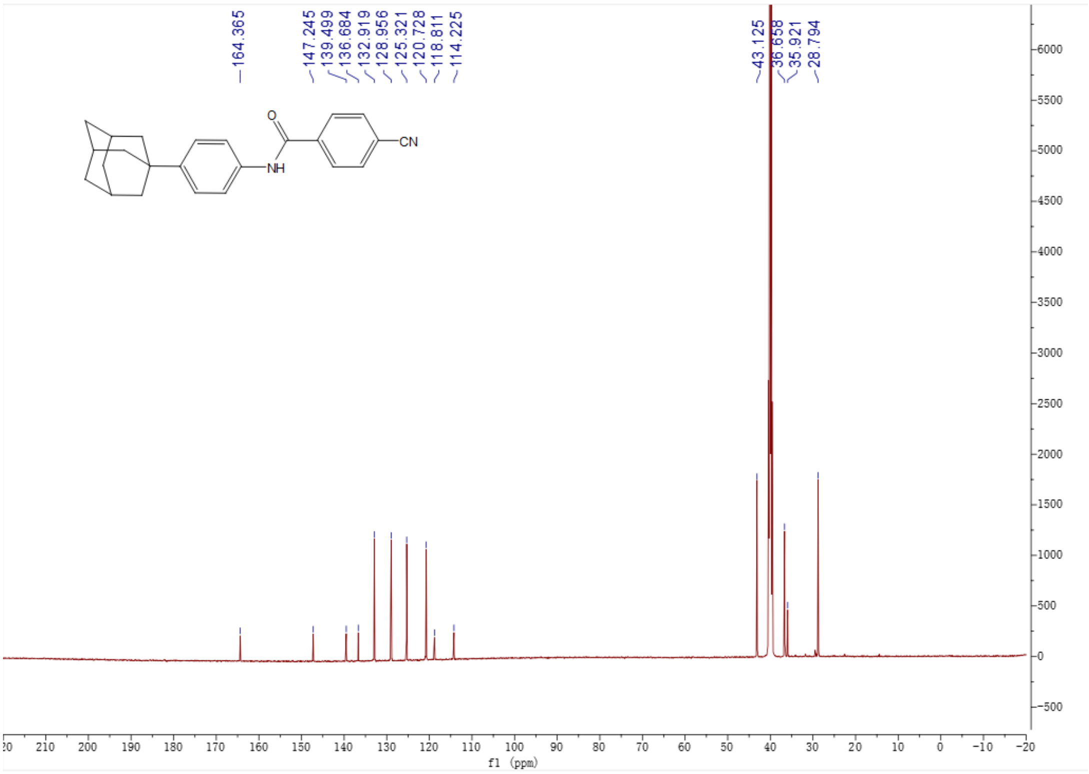

^{13}C NMR

*N-(4-((3r,5r,7r)-adamantan-1-yl)phenyl)-2-hydroxybenzamide(**1-3m**)*

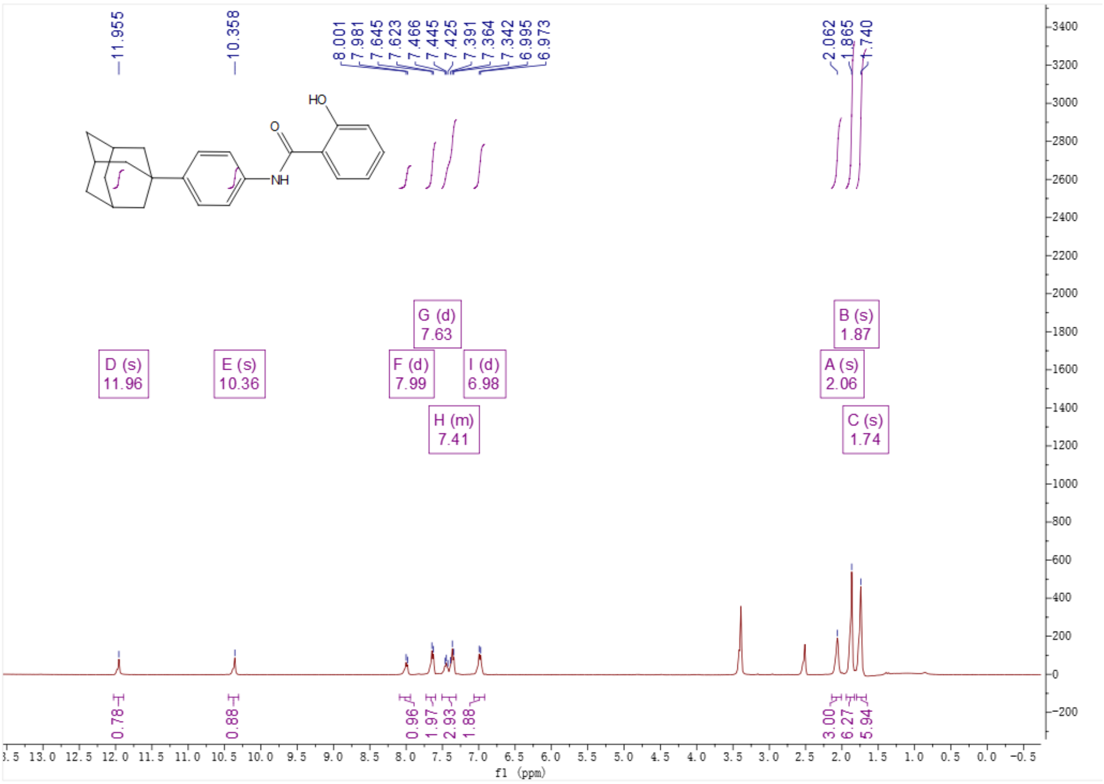

^{1}H NMR

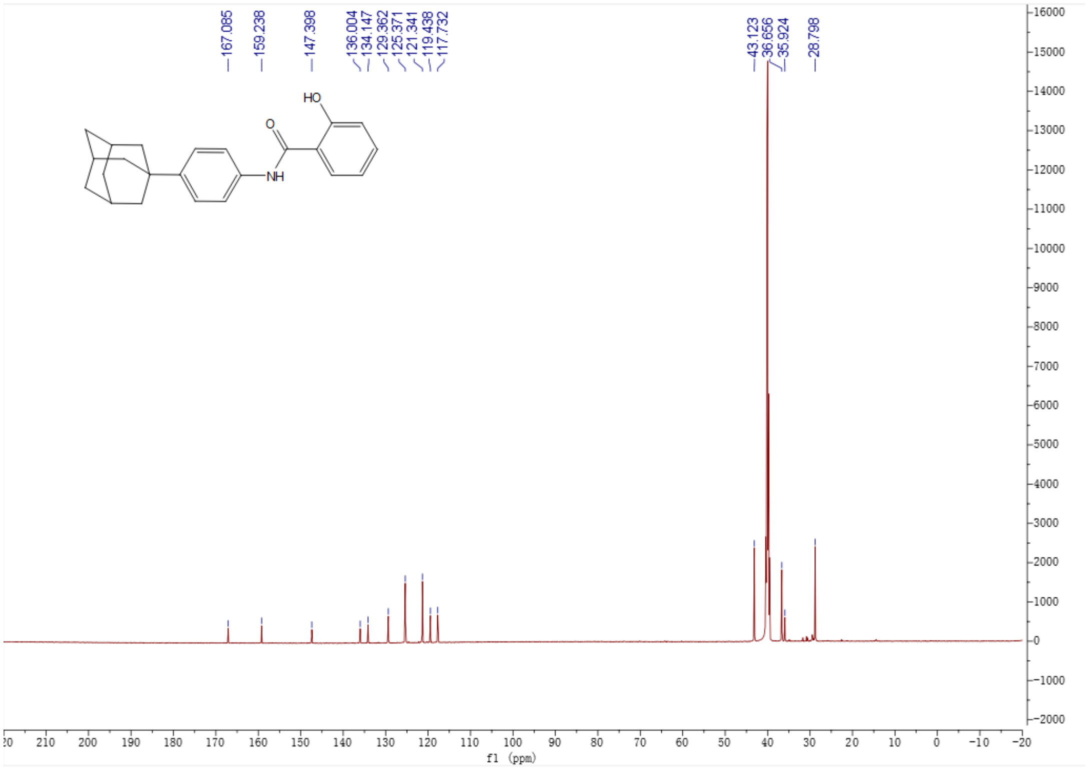

^{13}C NMR

*N-(4-((3r,5r,7r)-adamantan-1-yl)phenyl)-3-hydroxybenzamide(**1-3n**)*

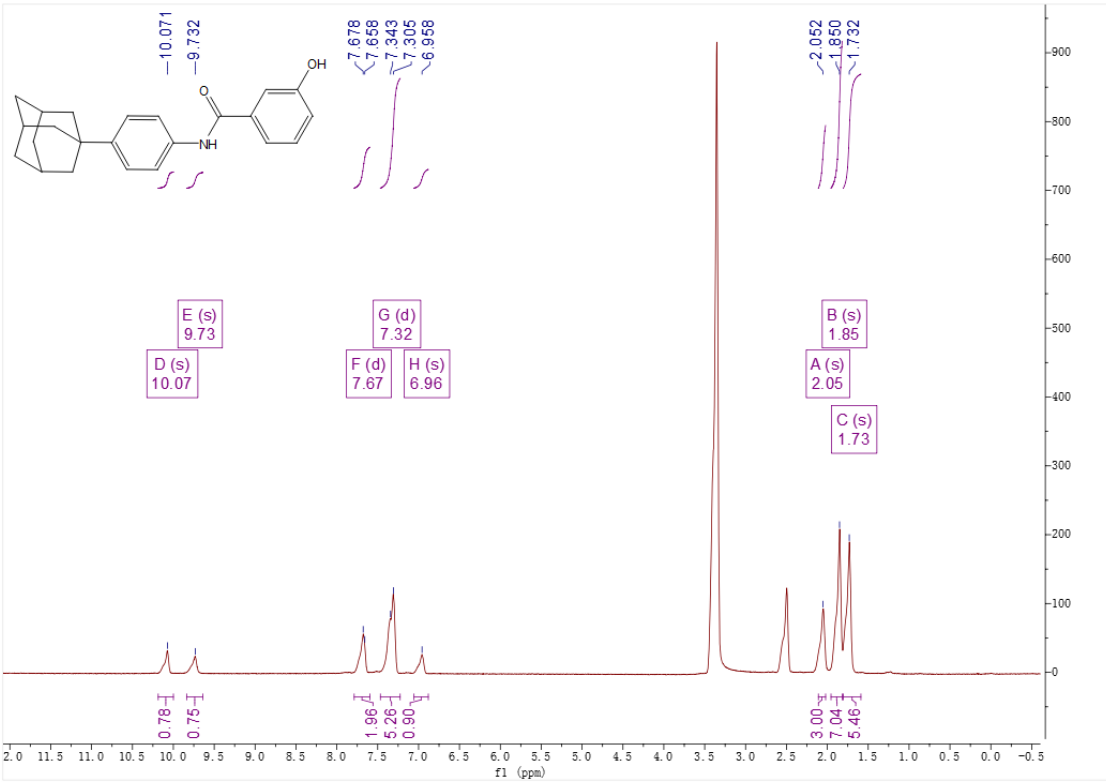

^{1}H NMR

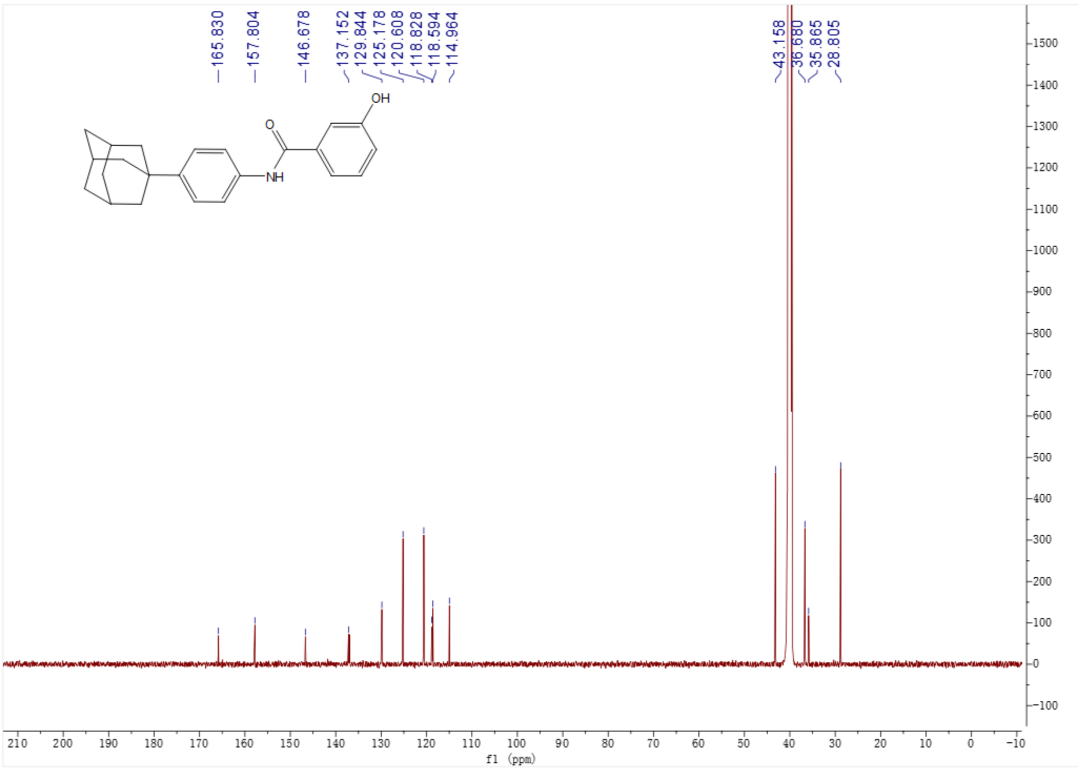

^{13}C NMR

*N-(4-((3r,5r,7r)-adamantan-1-yl)phenyl)-4-hydroxybenzamide(**1-3o**)*

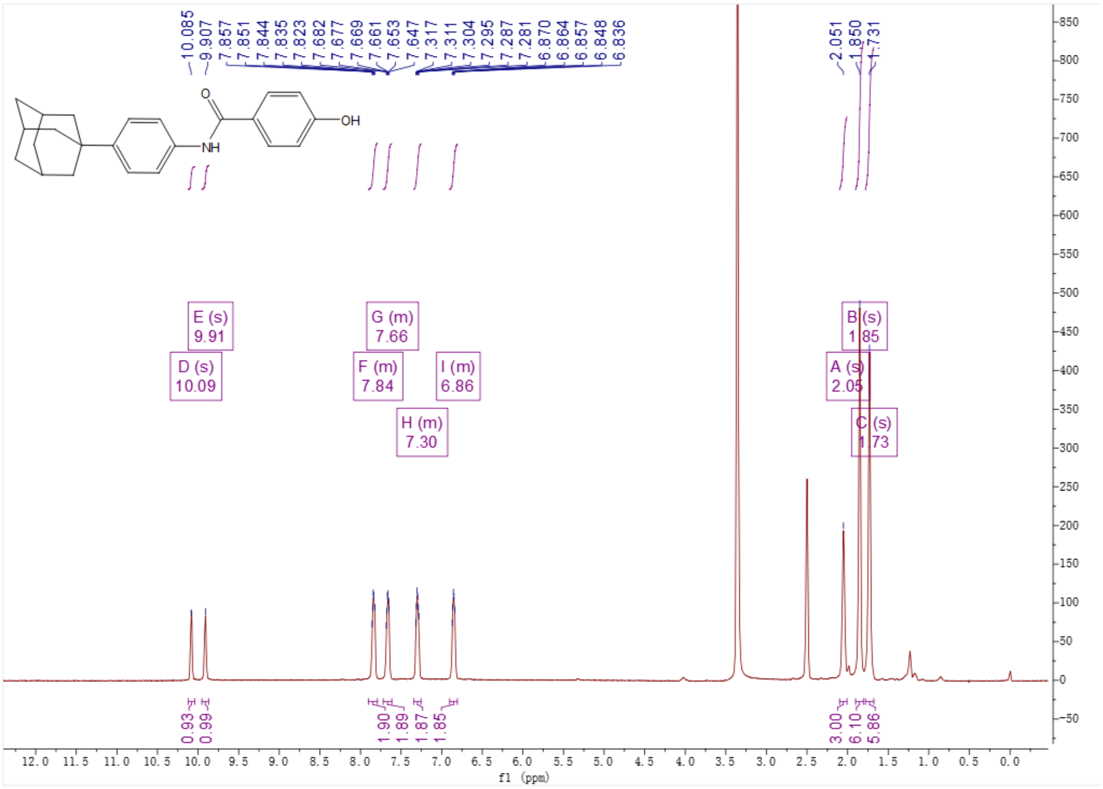

^{1}H NMR

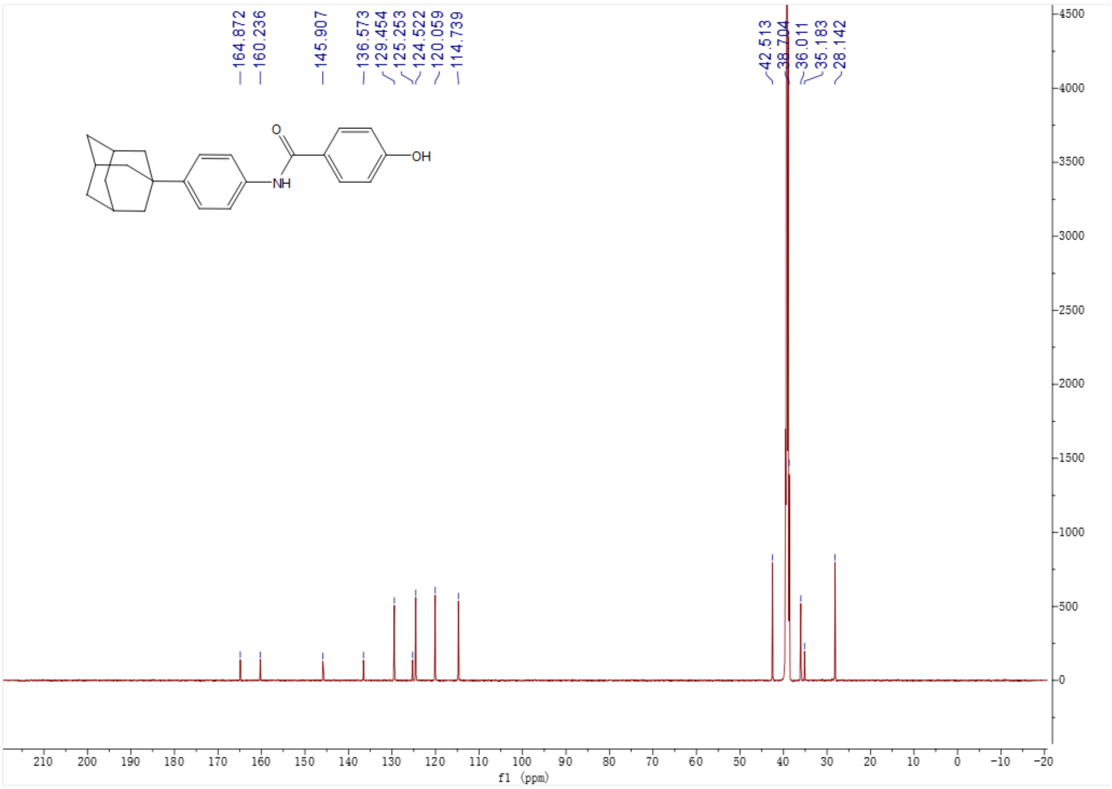

^{13}C NMR

*tert-Butyl 2-(2-((4-((3r,5r,7r)-adamantan-1-yl)phenyl)carbamoyl)phenyl)carbamate (**1-3p**)*

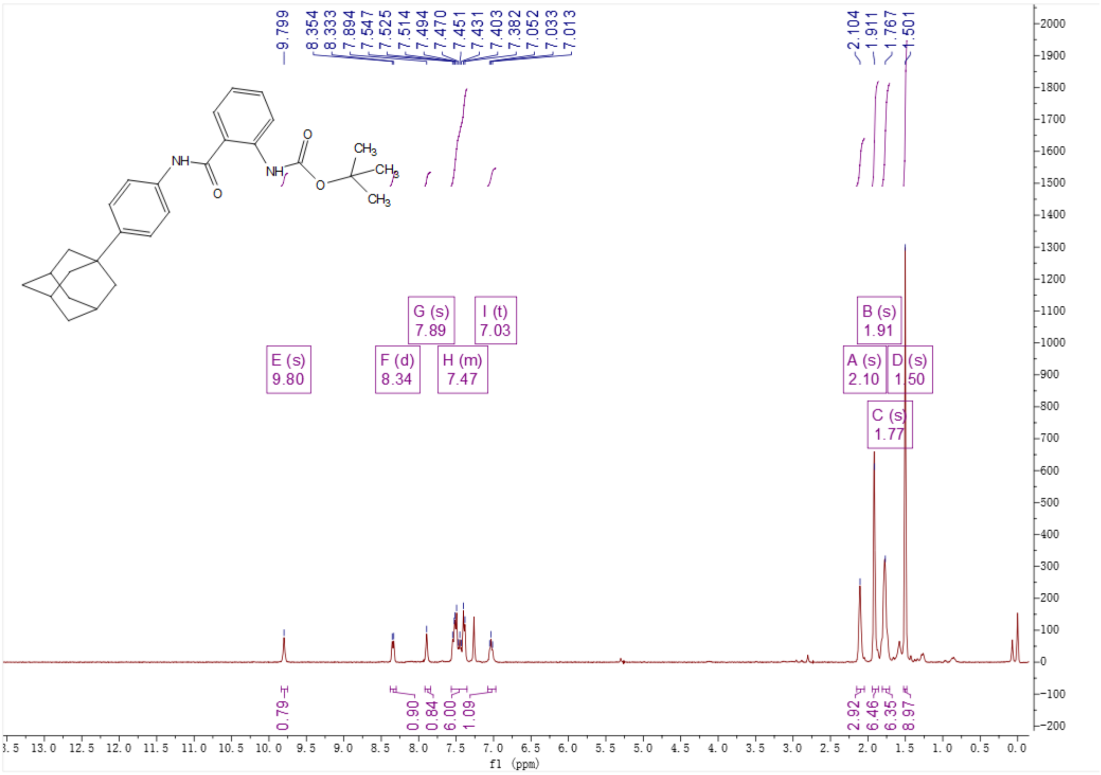

^{1}H NMR

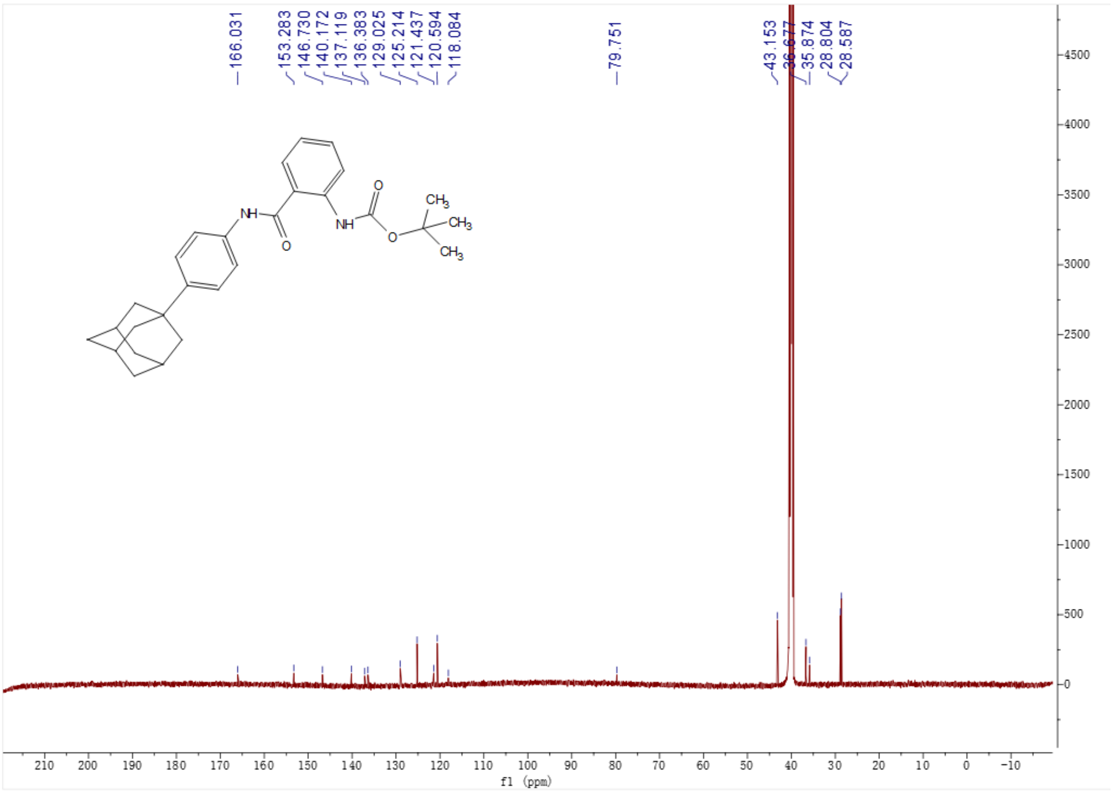

^{13}C NMR

*tert-Butyl (3-((4-((3r,5r,7r)-adamantan-1-yl)phenyl)carbamoyl)phenyl)carbamate (**1-3q**)*

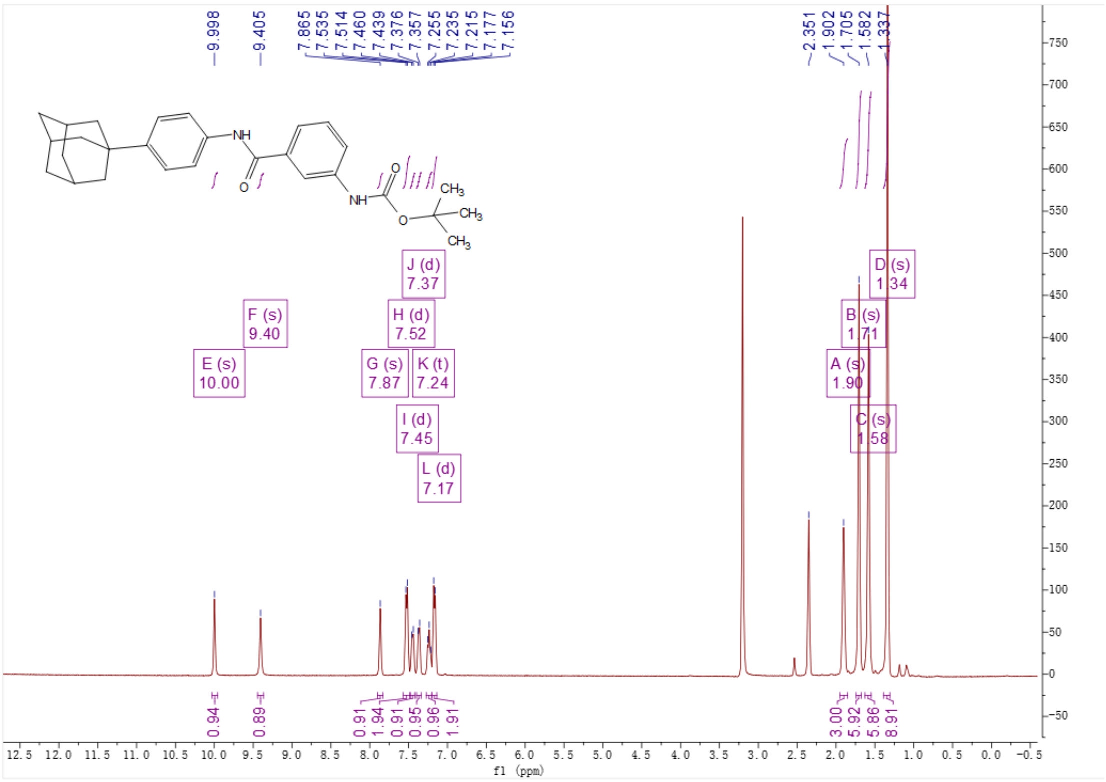

^{1}H NMR

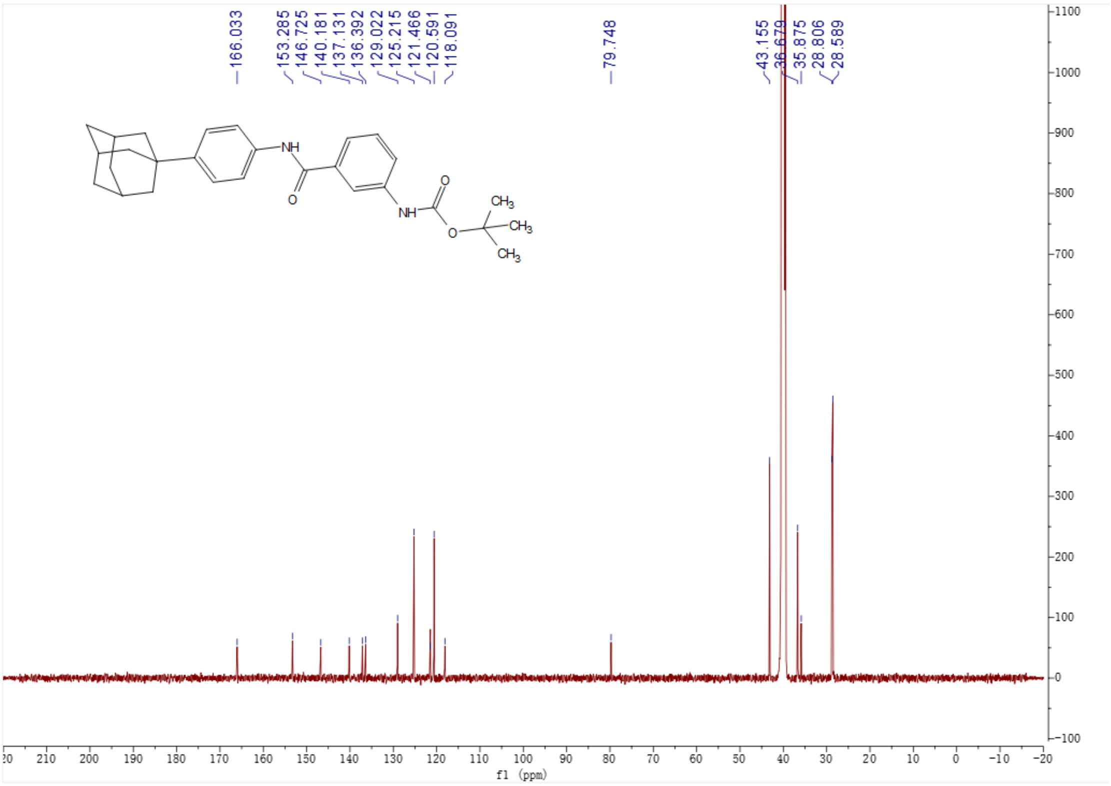

^{13}C NMR

*tert-Butyl (4-((4-((3r,5r,7r)-adamantan-1-yl)phenyl)carbamoyl)phenyl)carbamate (**1-3r**)*

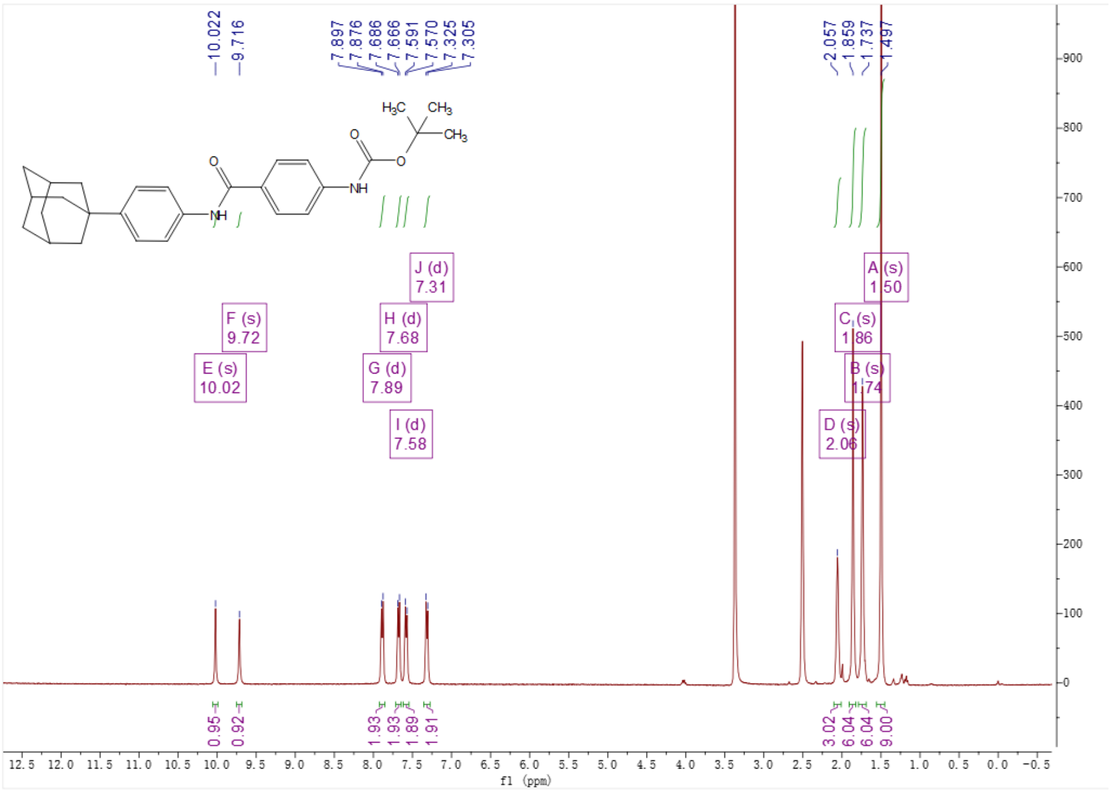

^{1}H NMR

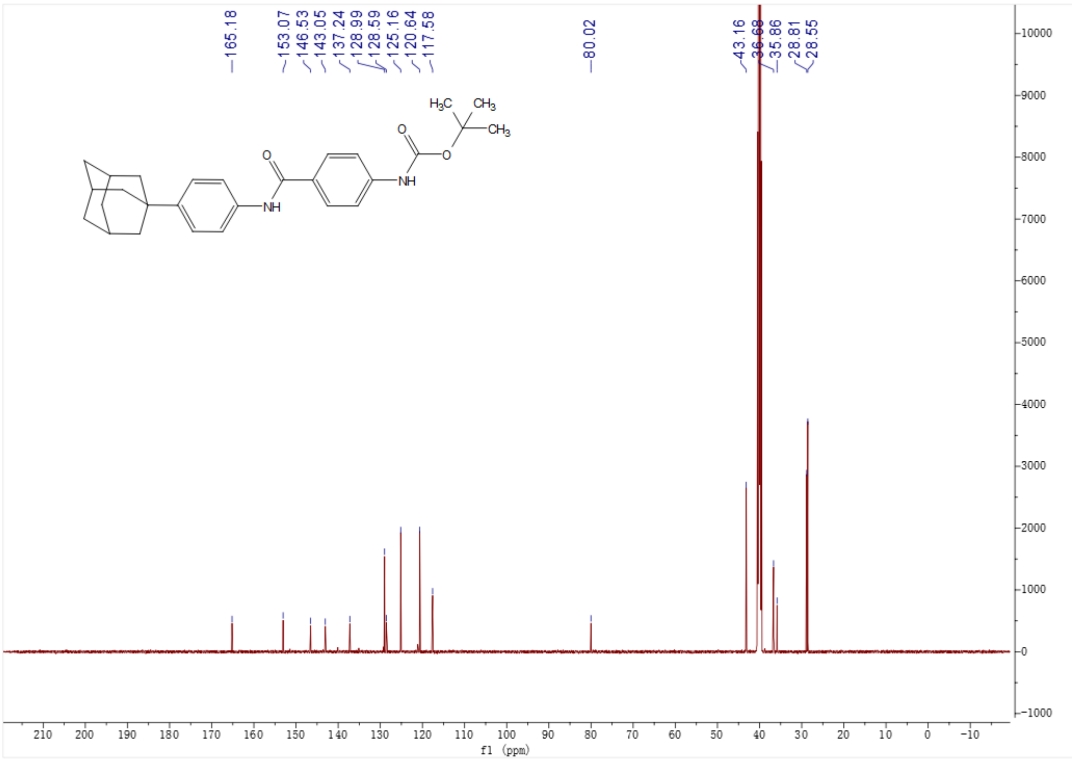

^{13}C NMR

*N-(4-((3r,5r,7r)-adamantan-1-yl)phenyl)-2-aminobenzamide(**1-3s**)*

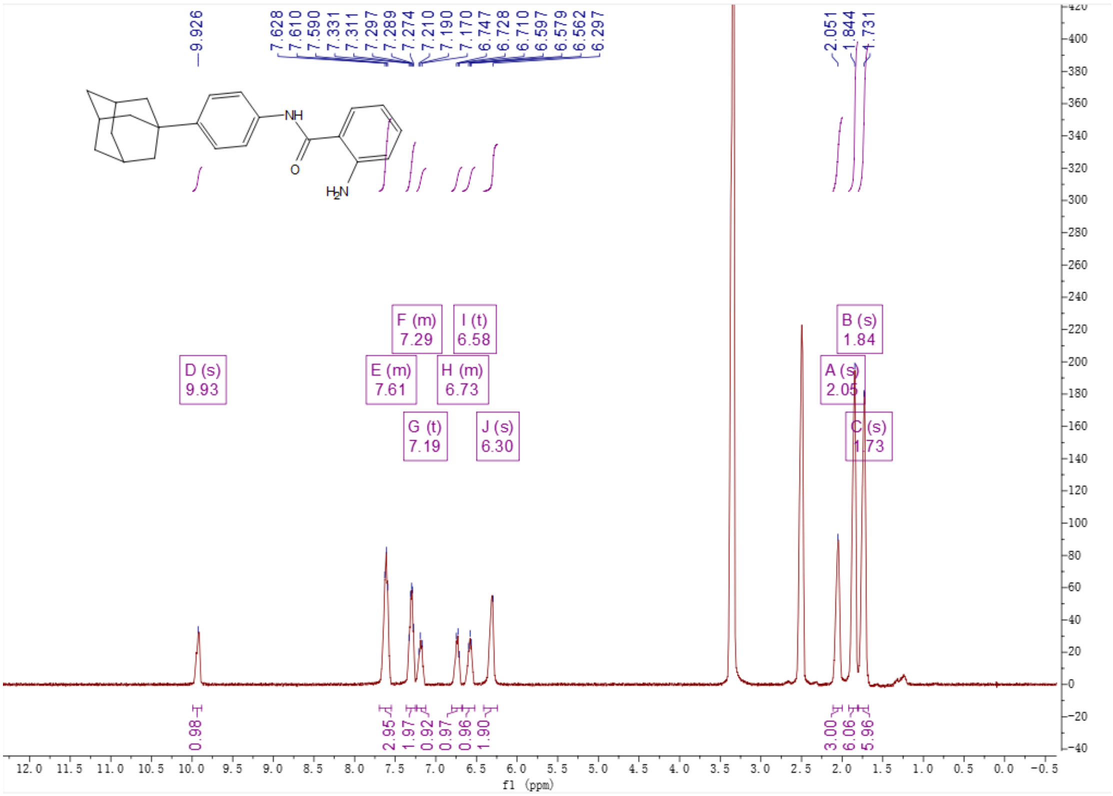

^{1}H NMR

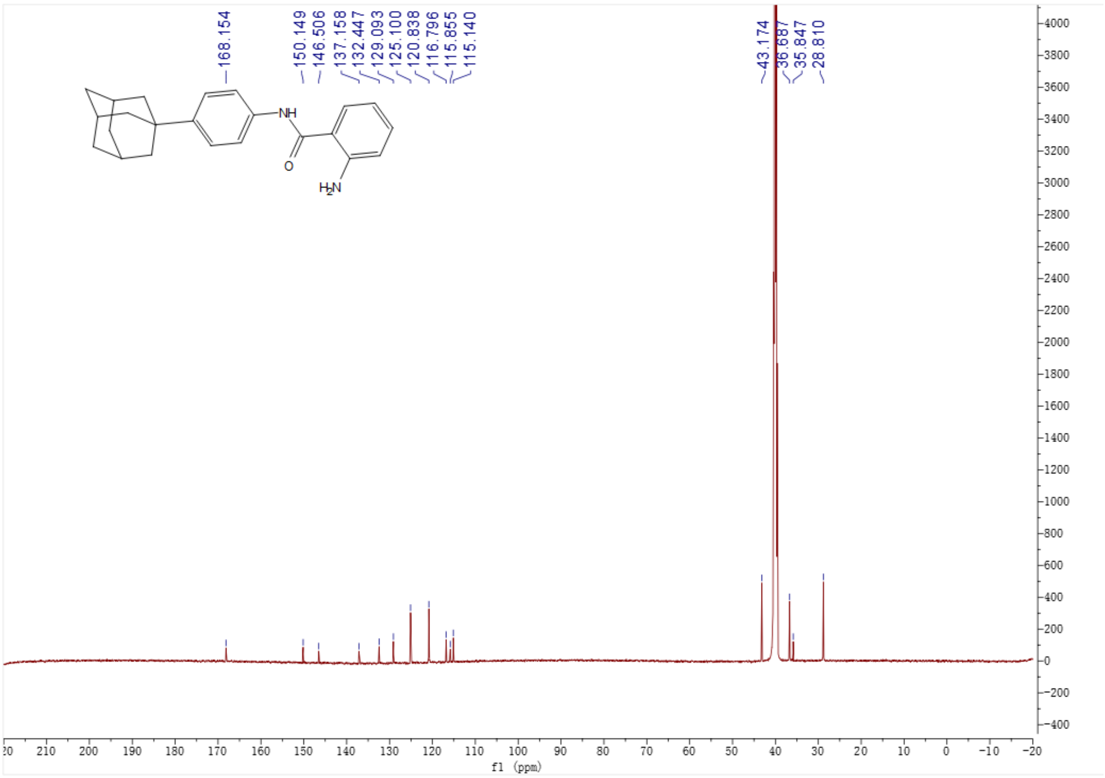

^{13}C NMR

*N-(4-((3r,5r,7r)-adamantan-1-yl)phenyl)-3-aminobenzamide(**1-3t**)*

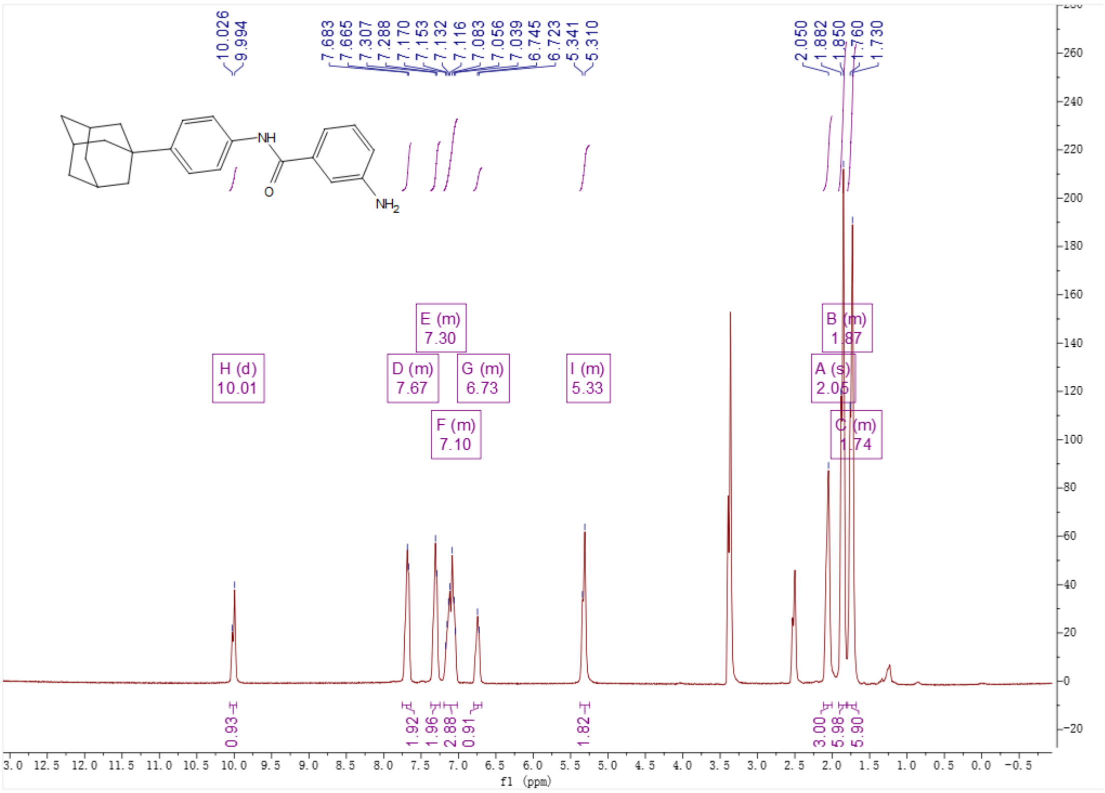

^{1}H NMR

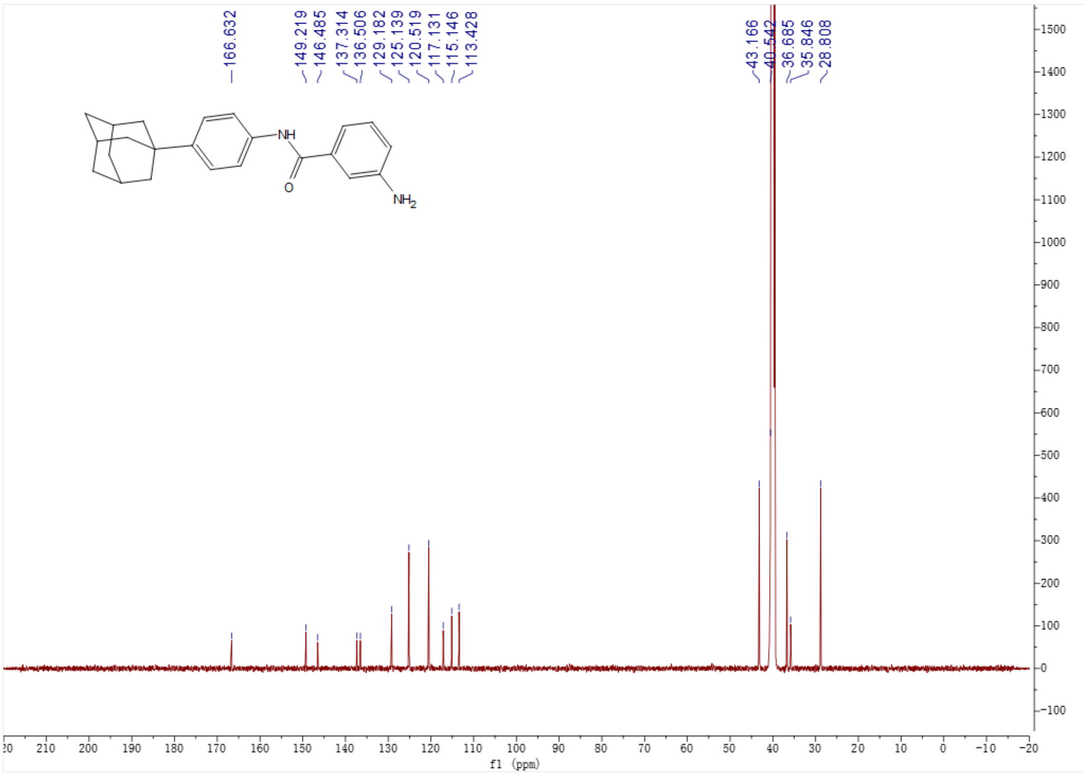

^{13}C NMR

*N-(4-((3r,5r,7r)-adamantan-1-yl)phenyl)-4-aminobenzamide(**1-3u**)*

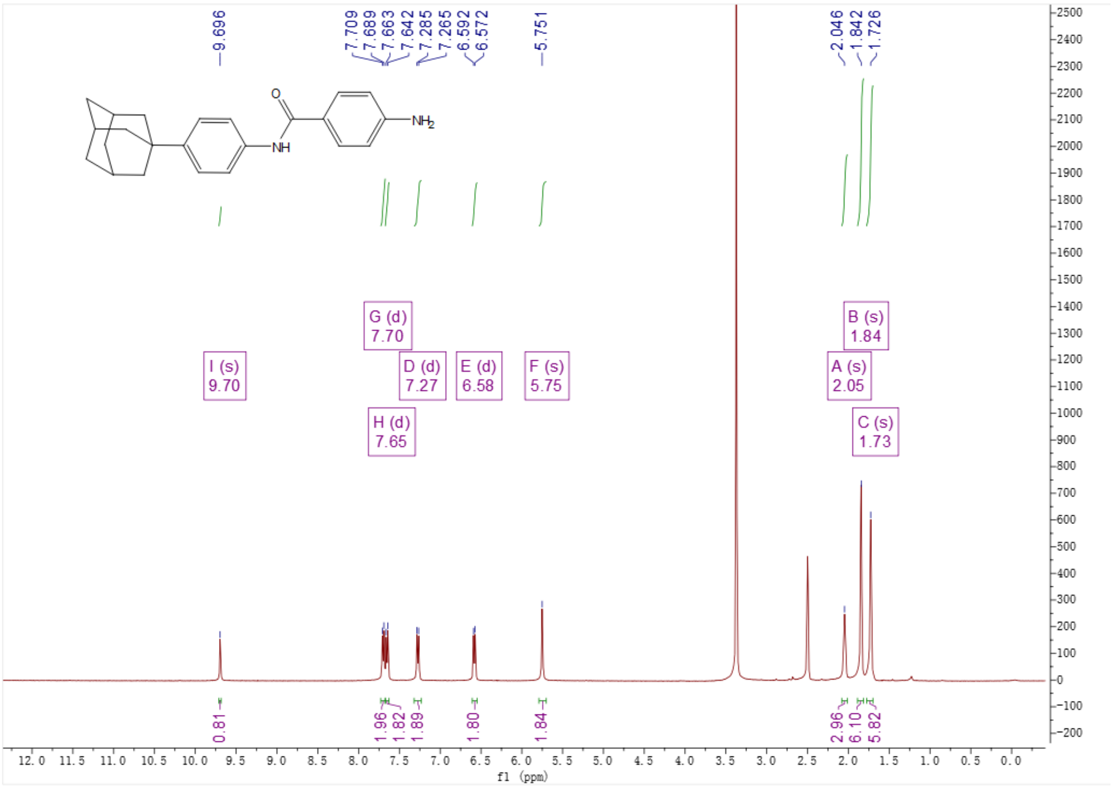

^{1}H NMR

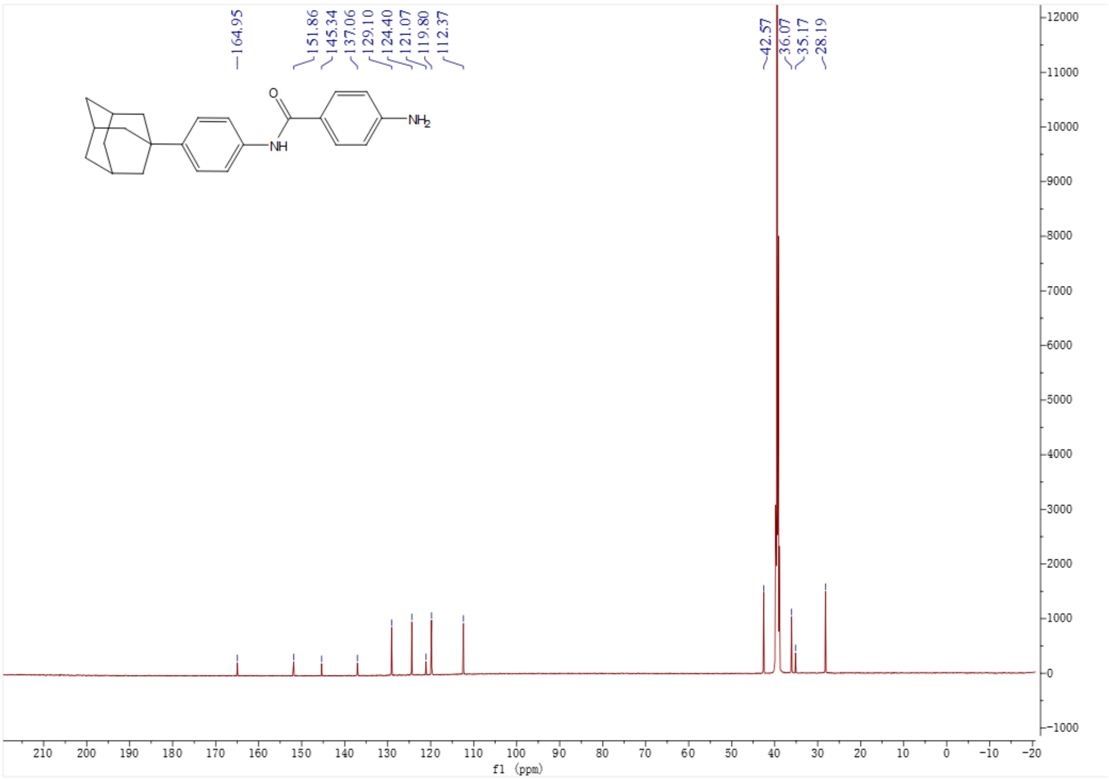

^{13}C NMR

*2-Acetamido-N-(4-((3r,5r,7r)-adamantan-1-yl)phenyl)benzamide(**1-3v**)*

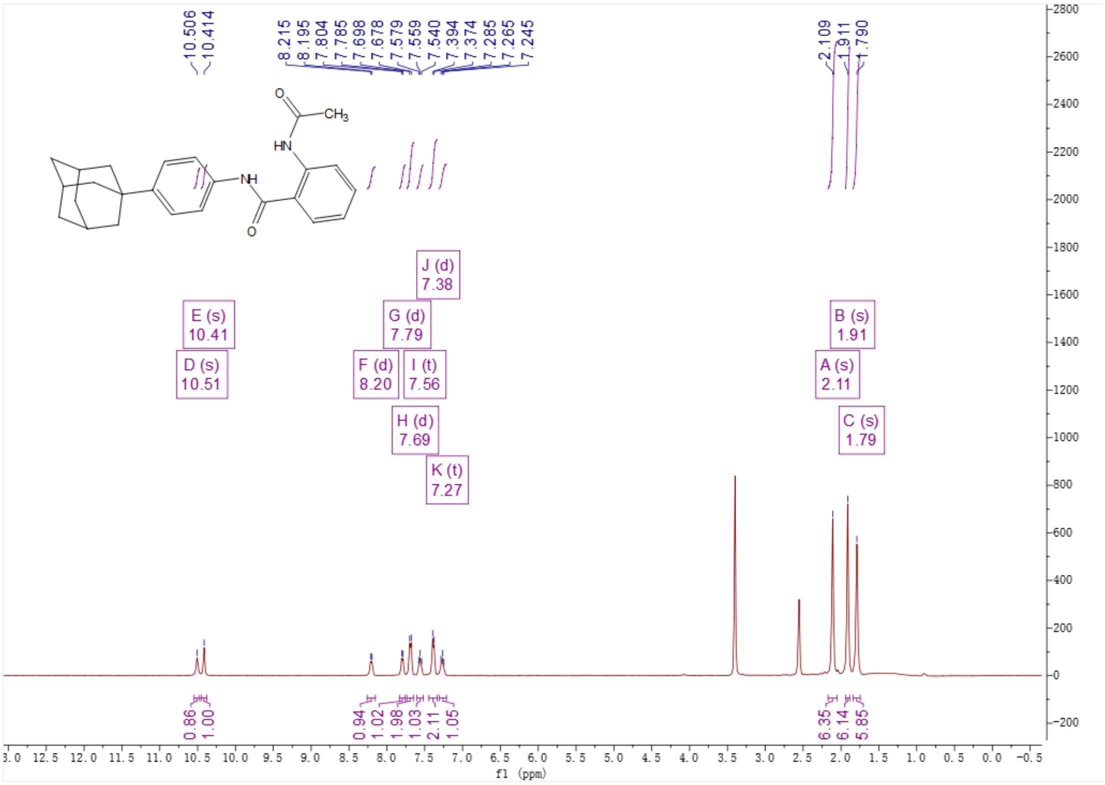

^{1}H NMR

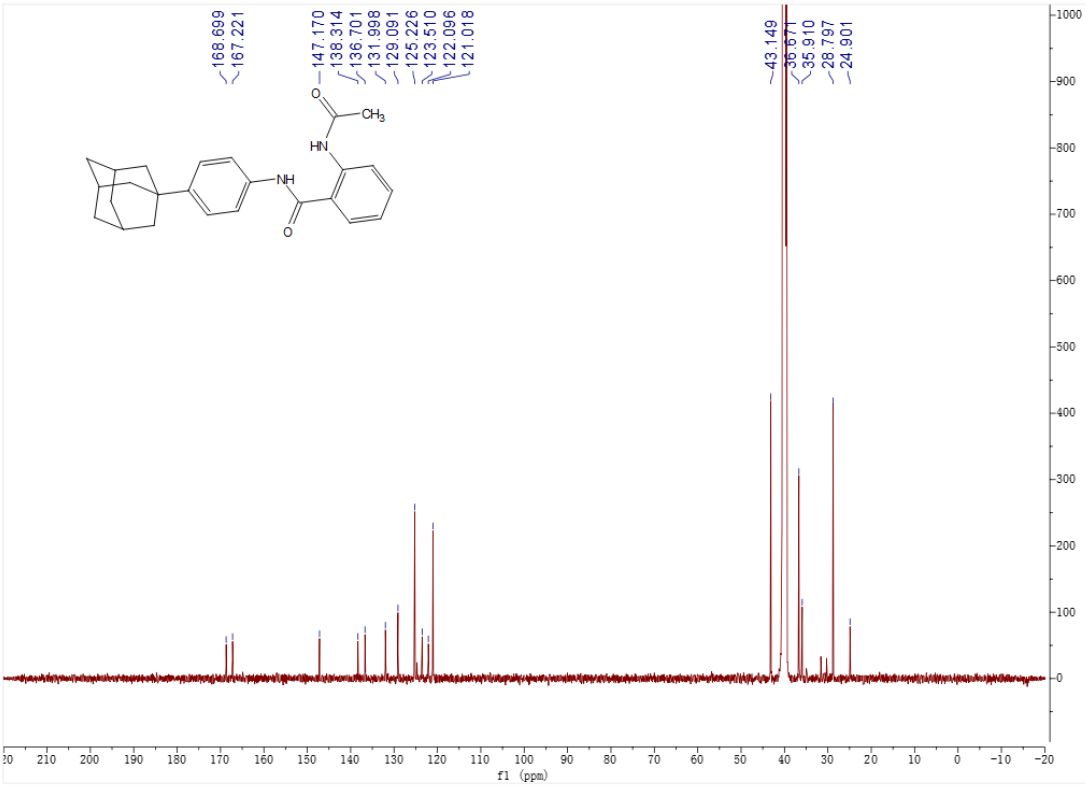

^{13}C NMR

*3-Acetamido-N-(4-((3r,5r,7r)-adamantan-1-yl)phenyl)benzamide(**1-3w**)*

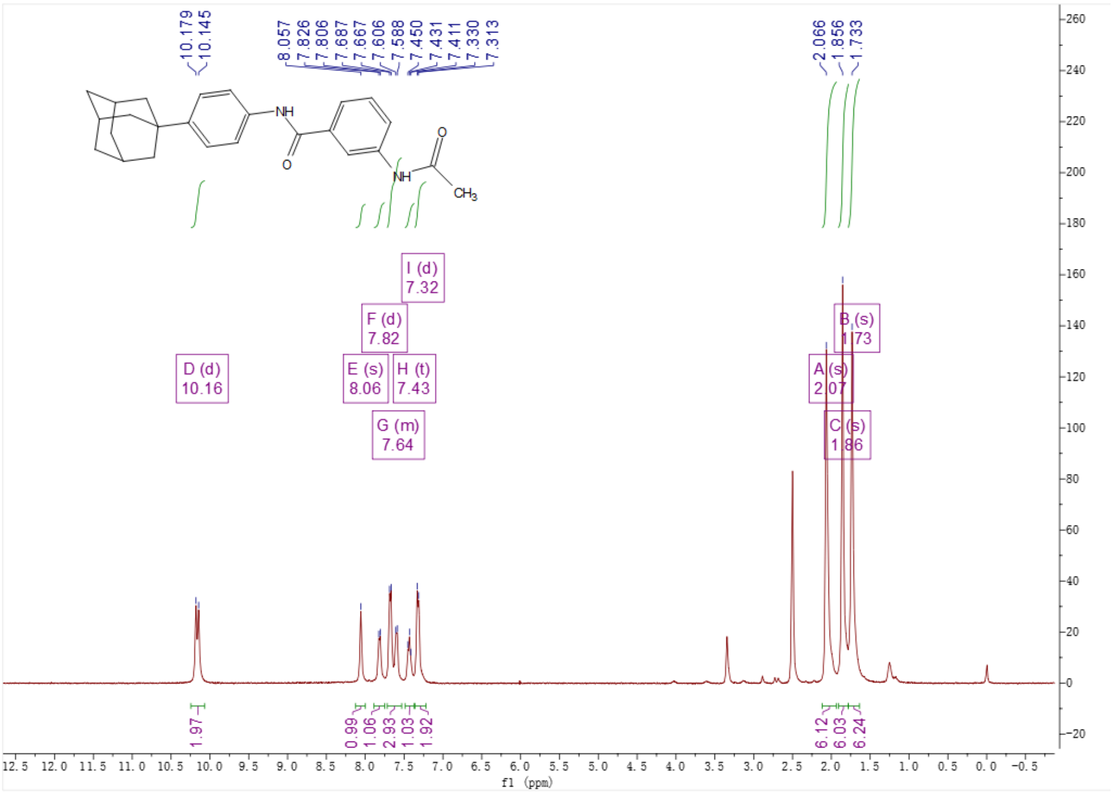

^{1}H NMR

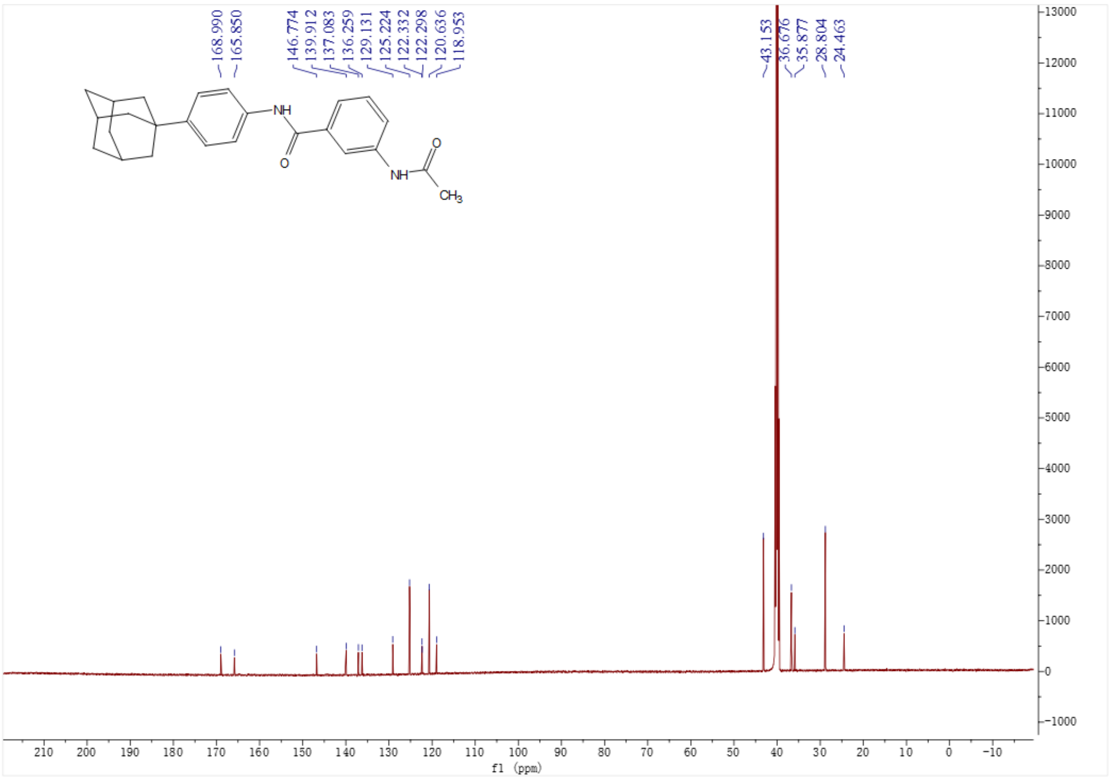

^{13}C NMR

4-Acetamido-N-(4-((3r,5r,7r)-adamantan-1-yl)phenyl)benzamide ***(1-3x)***

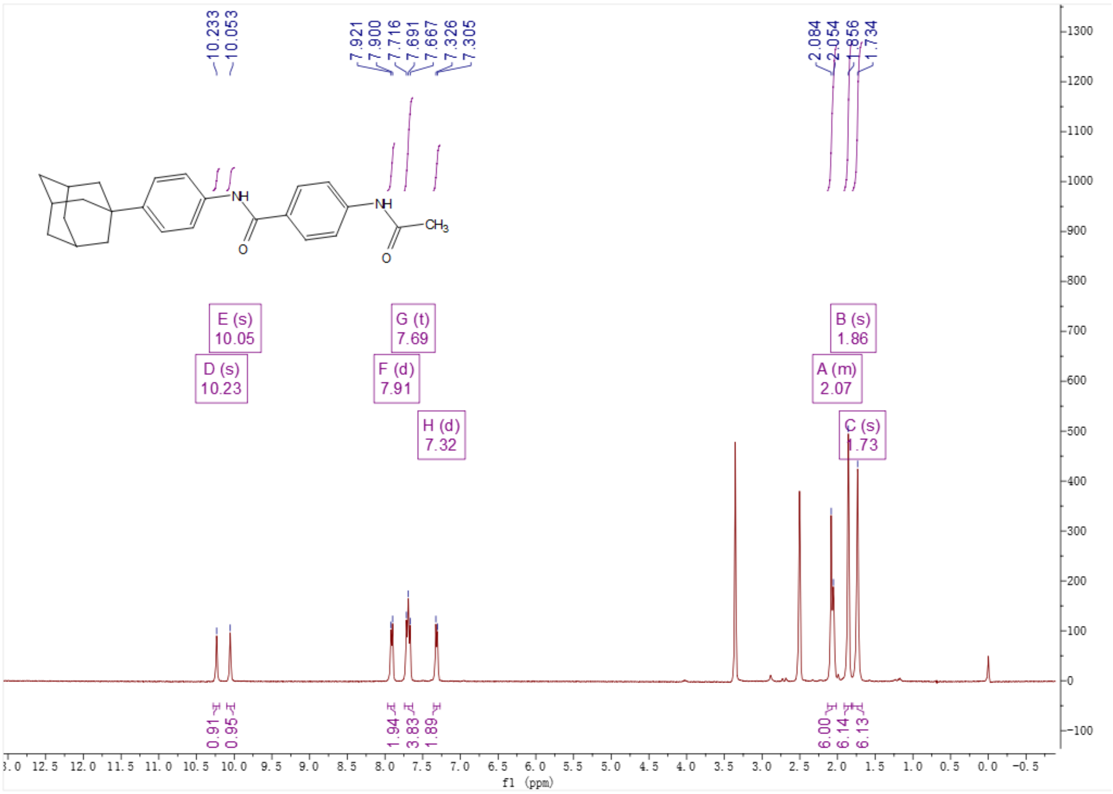

^{1}H NMR

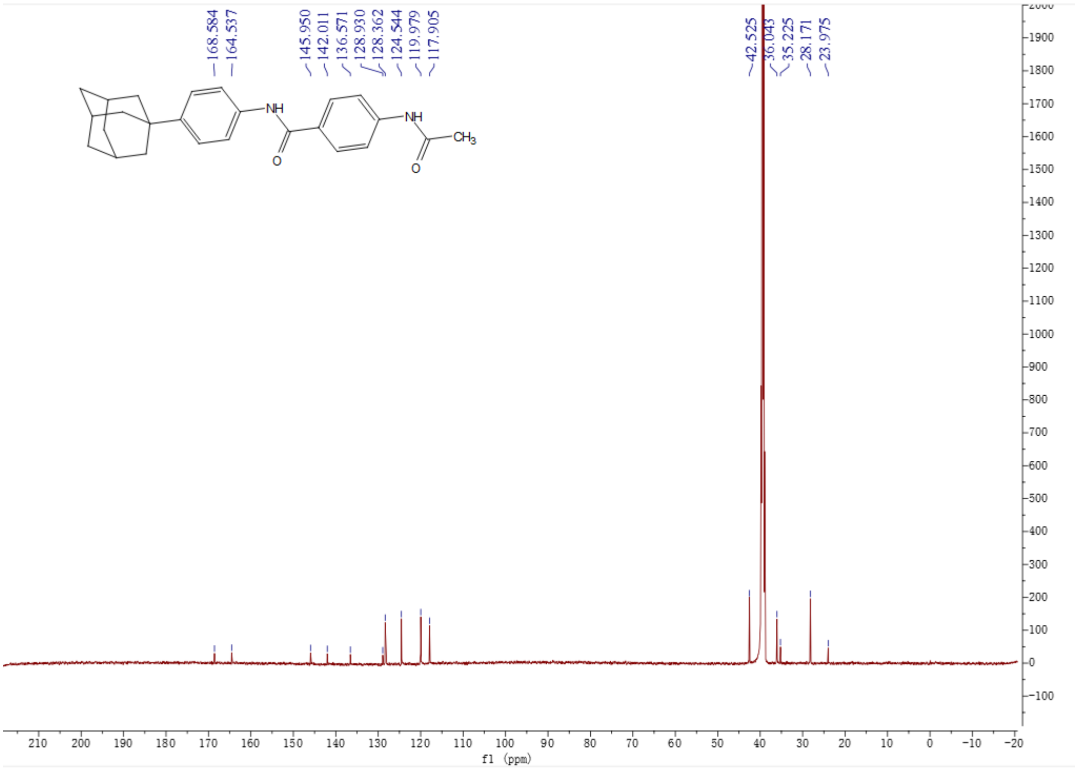

^{13}C NMR

N-(4-((3r,5r,7r)-adamantan-1-yl)phenyl)-4-(5-((3aS,4S,6aR)-2-oxohexahydro-1H-thieno[3,4-d]imidazol-4-yl)pentanamido)benzamide ***(1-3y, HI-102)***

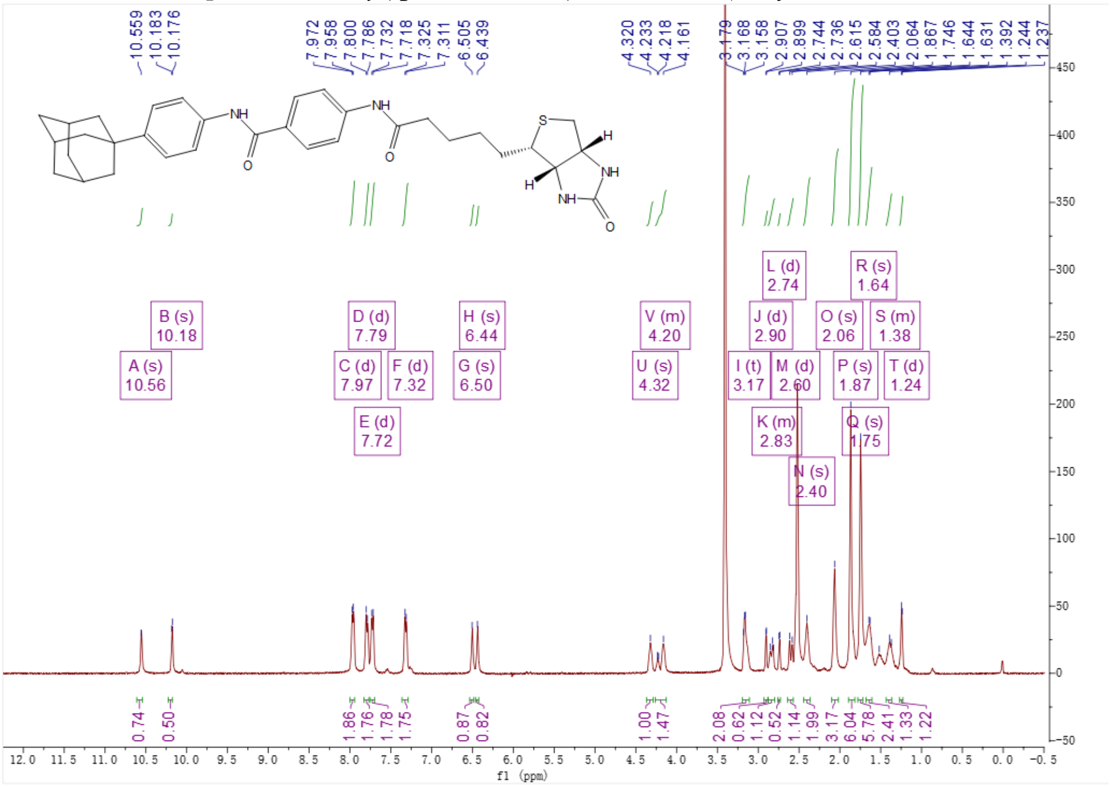

^{1}H NMR

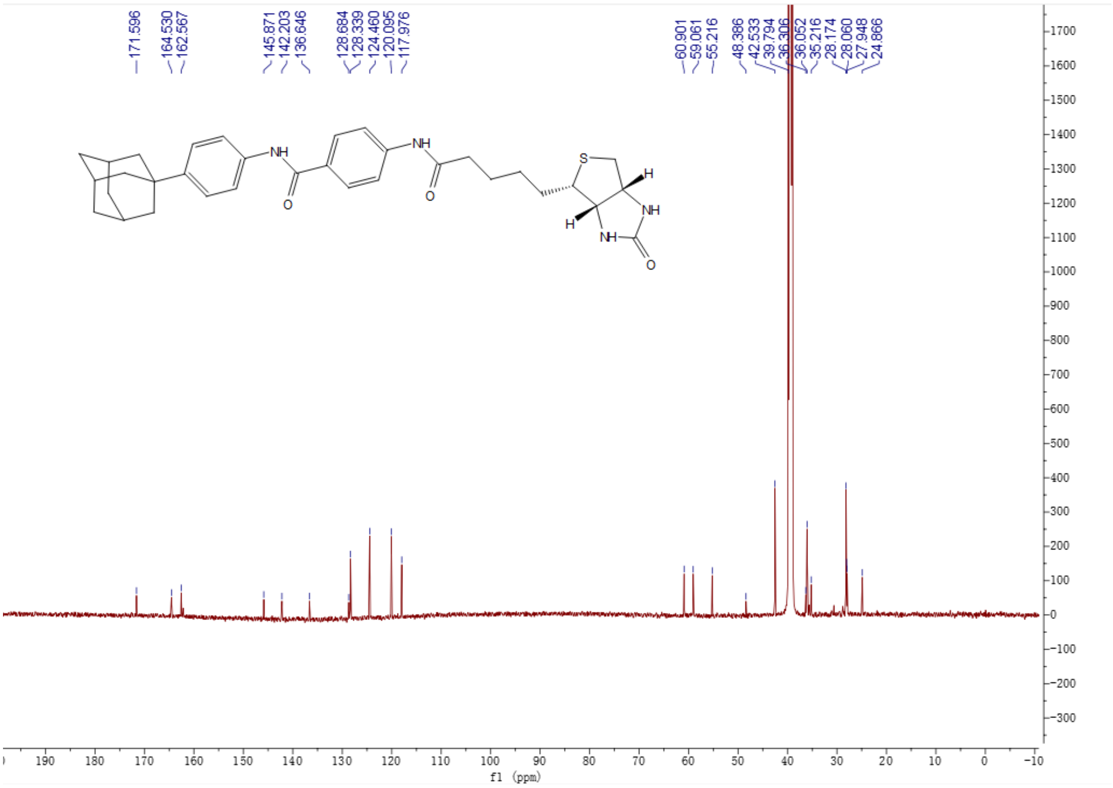

^{13}C NMR

*N-(4-((3r,5r,7r)-adamantan-1-yl)phenyl)-4-(4-(5,5-difluoro-1,3,7,9-tetramethyl-5H-4l4,5l4-dipyrrolo[1,2-c:2',1'-f][1,3,2]diazaborinin-10-yl)butanamido)benzamide(**1-3z, HI-103**)*

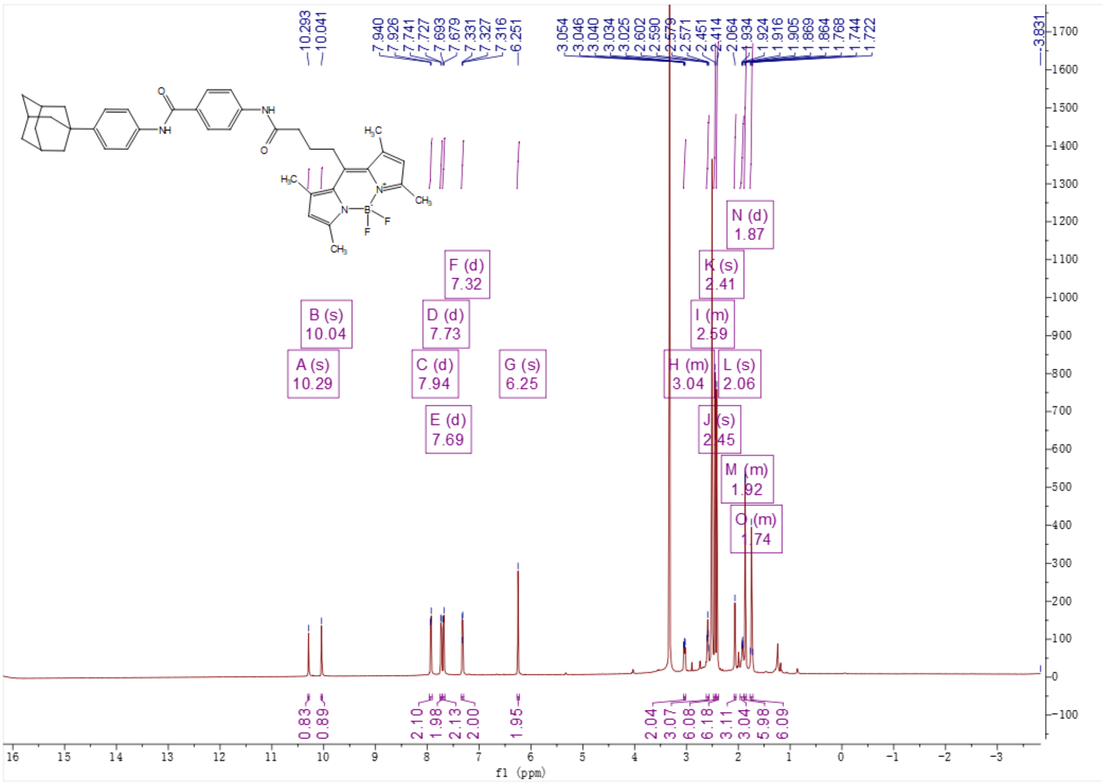

^{1}H NMR

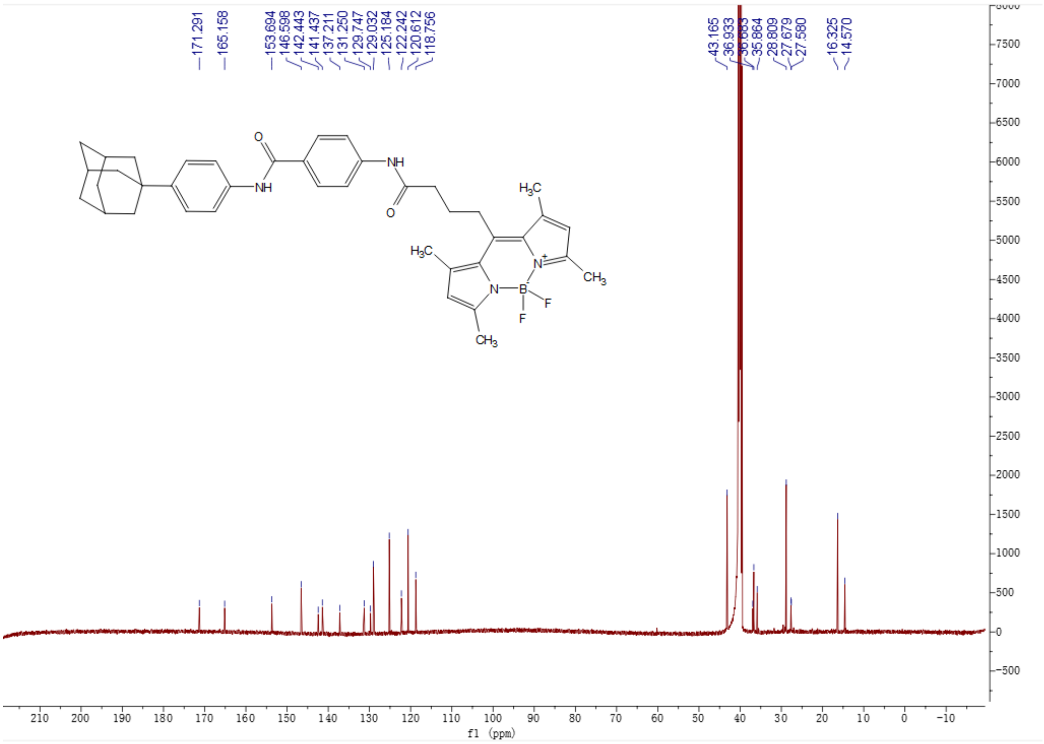

^{13}C NMR

*N-(4-((3r,5r,7r)-adamantan-1-yl)phenyl)-2-(dimethylamino)benzamide(**1-3aa**)*

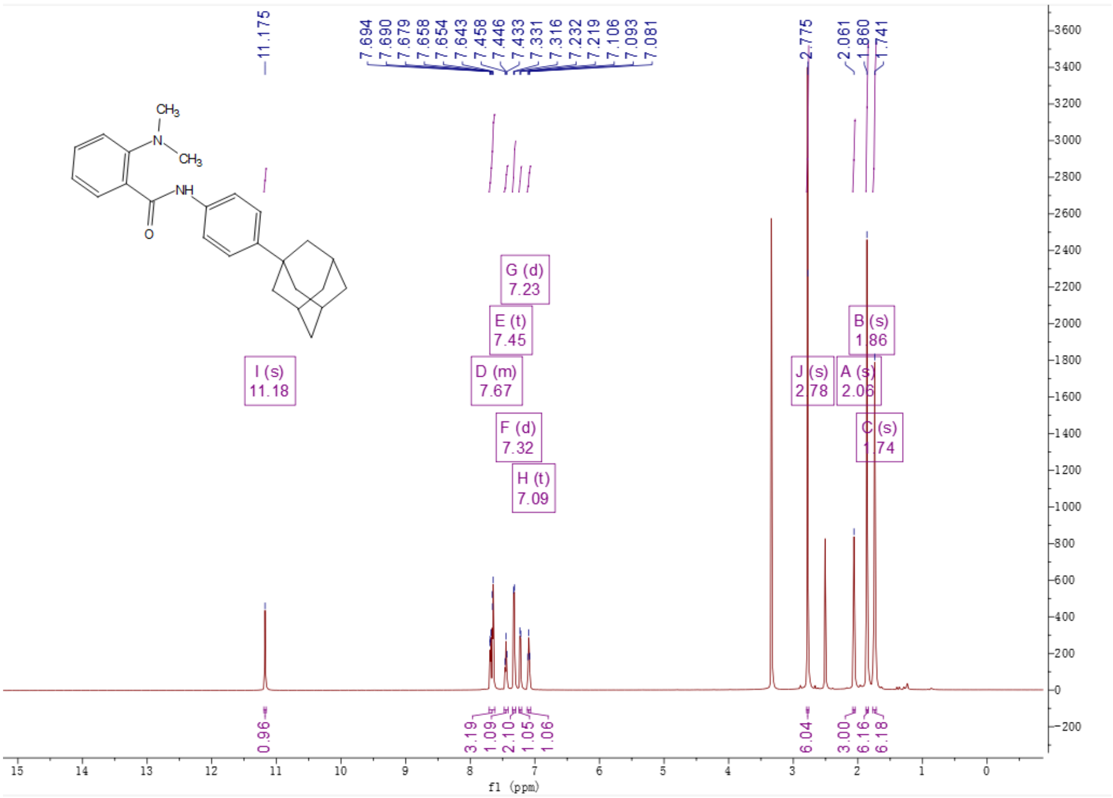

^{1}H NMR

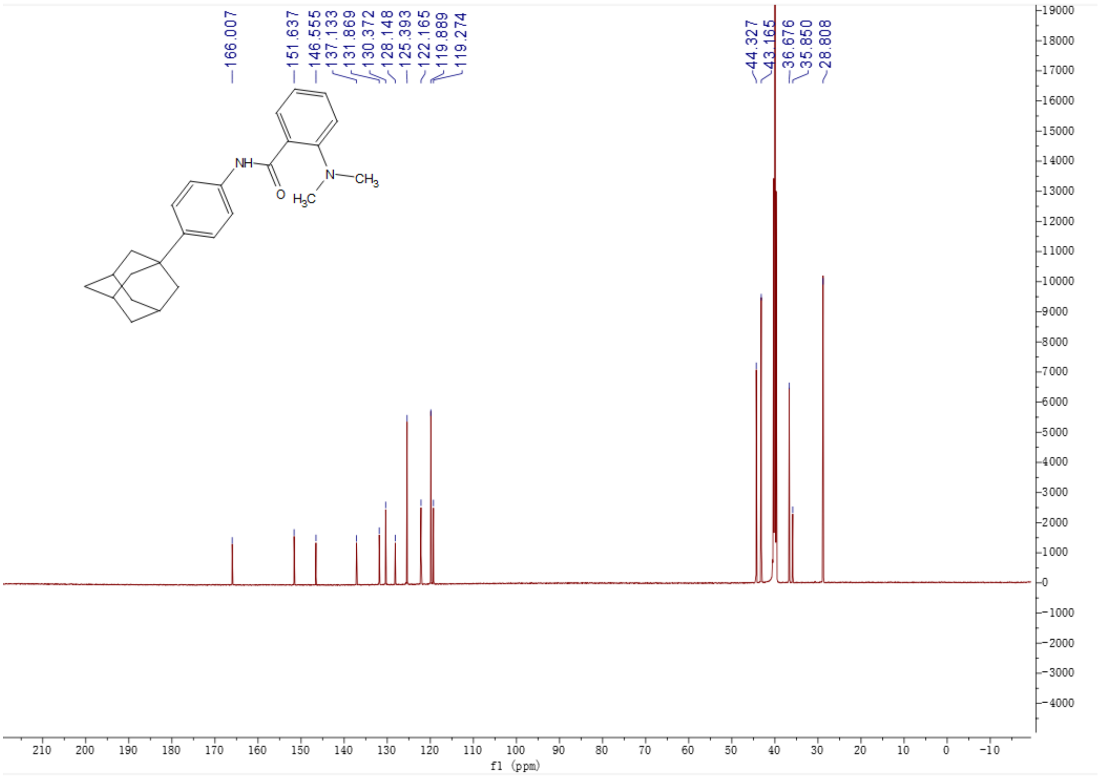

^{13}C NMR

*N-(4-((3r,5r,7r)-adamantan-1-yl)phenyl)-3-(dimethylamino)benzamide(**1-3ab**)*

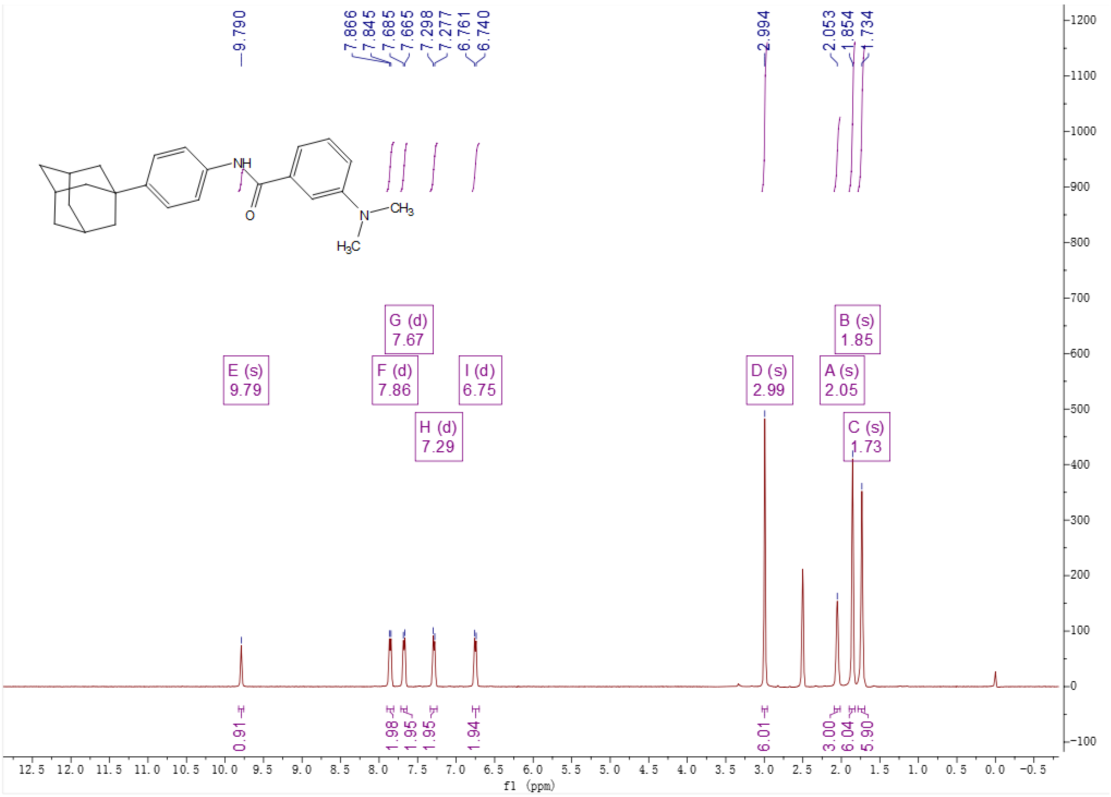

^{1}H NMR

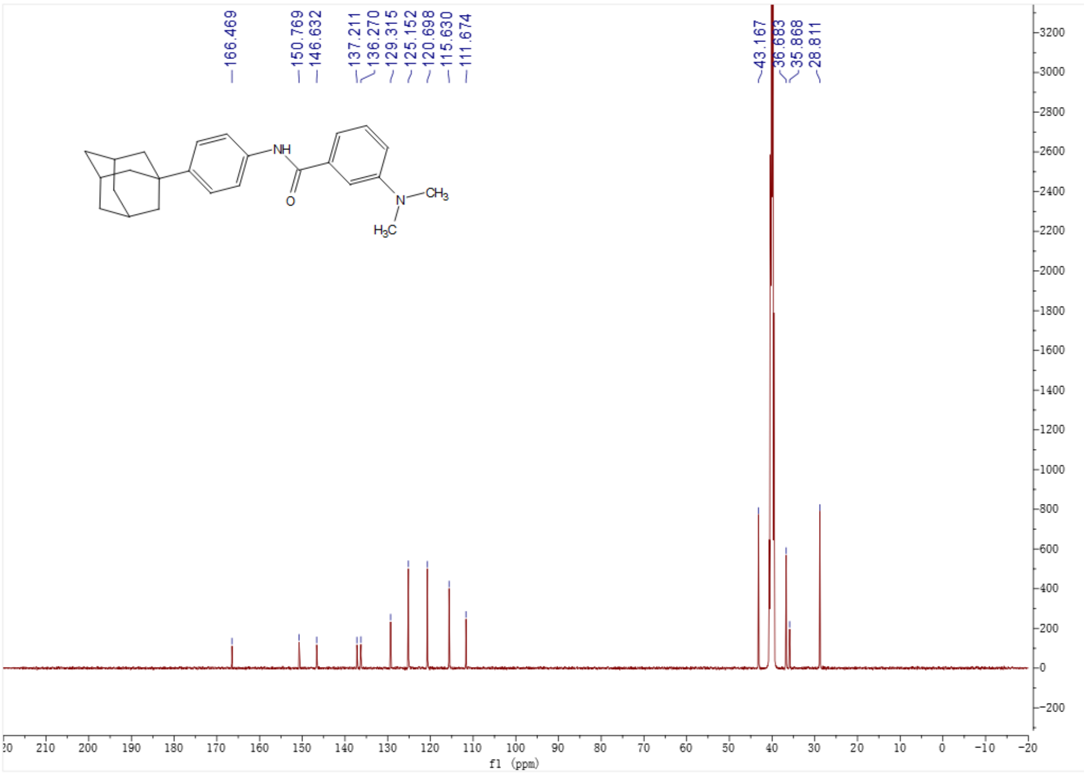

^{13}C NMR

*N-(4-((3r,5r,7r)-adamantan-1-yl)phenyl)-4-(dimethylamino)benzamide(**1-3ac**)*

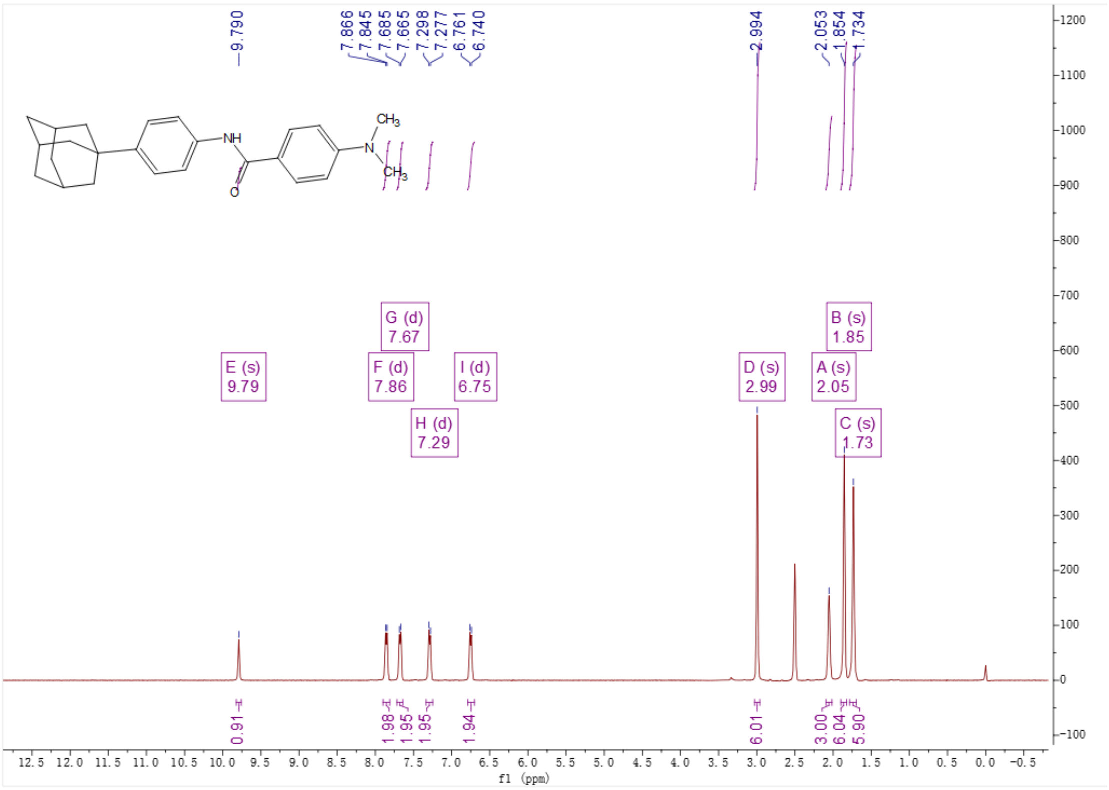

^{1}H NMR

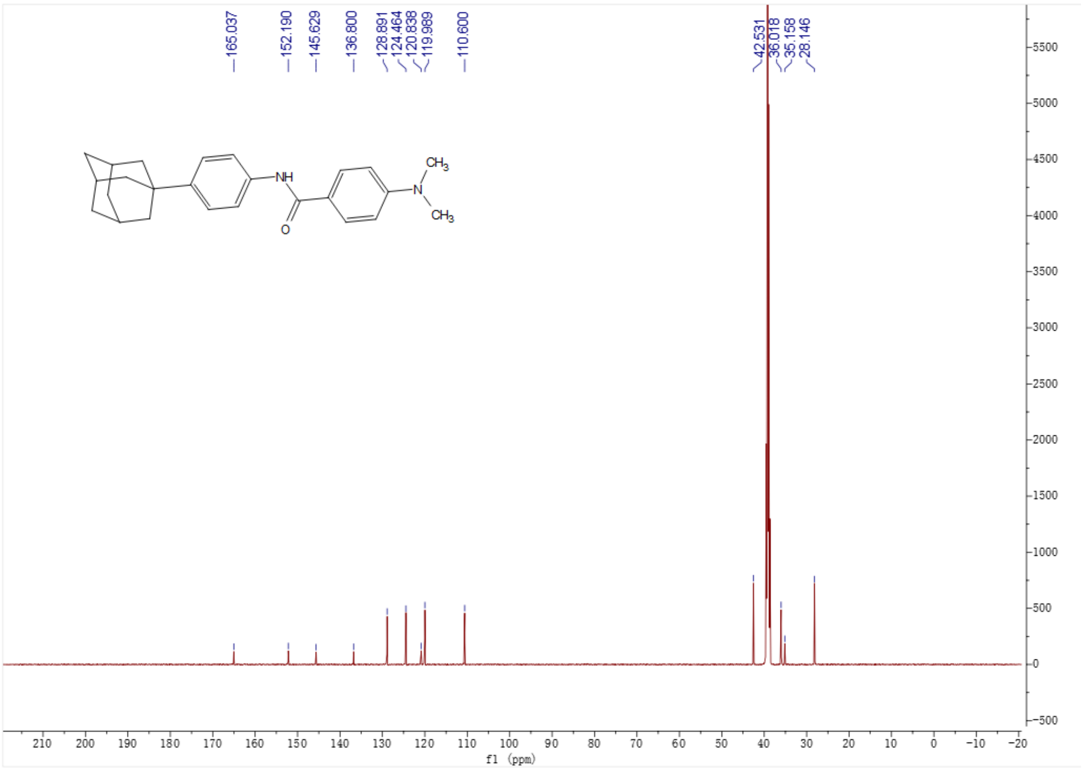

^{13}C NMR

*N-(4-((3r,5r,7r)-adamantan-1-yl)phenyl)-2-(trifluoromethyl)benzamide(**1-3ad**)*

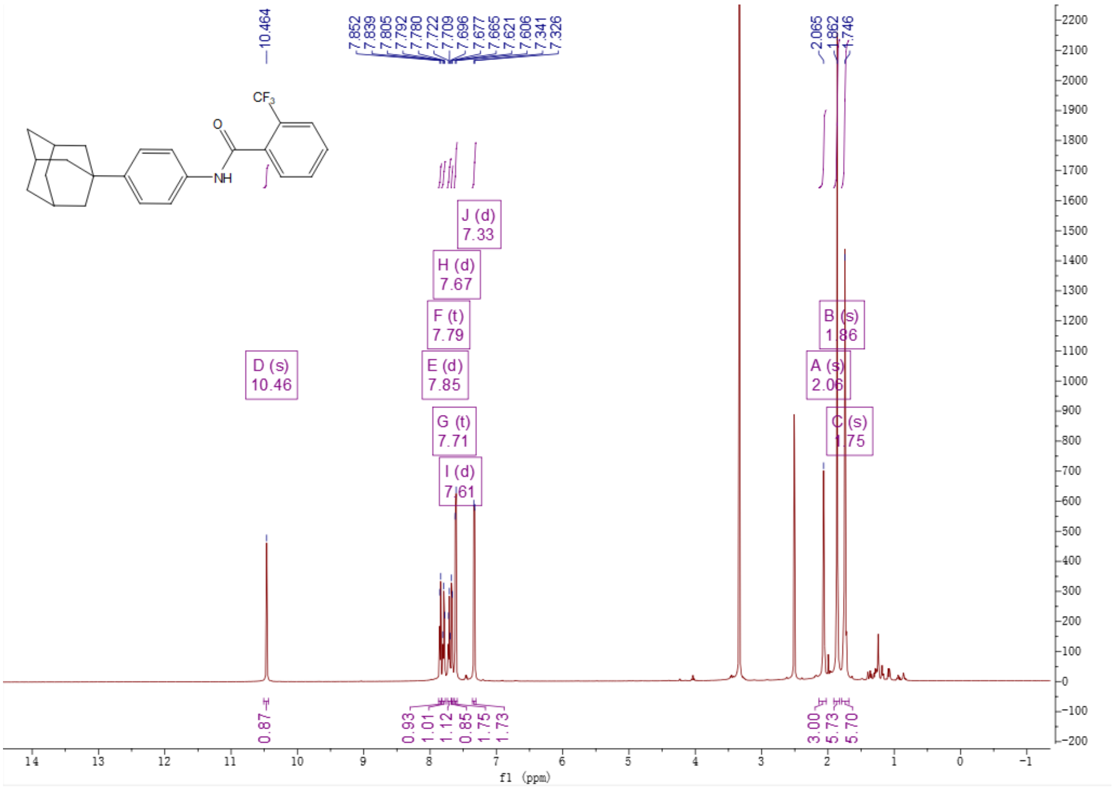

^{1}H NMR

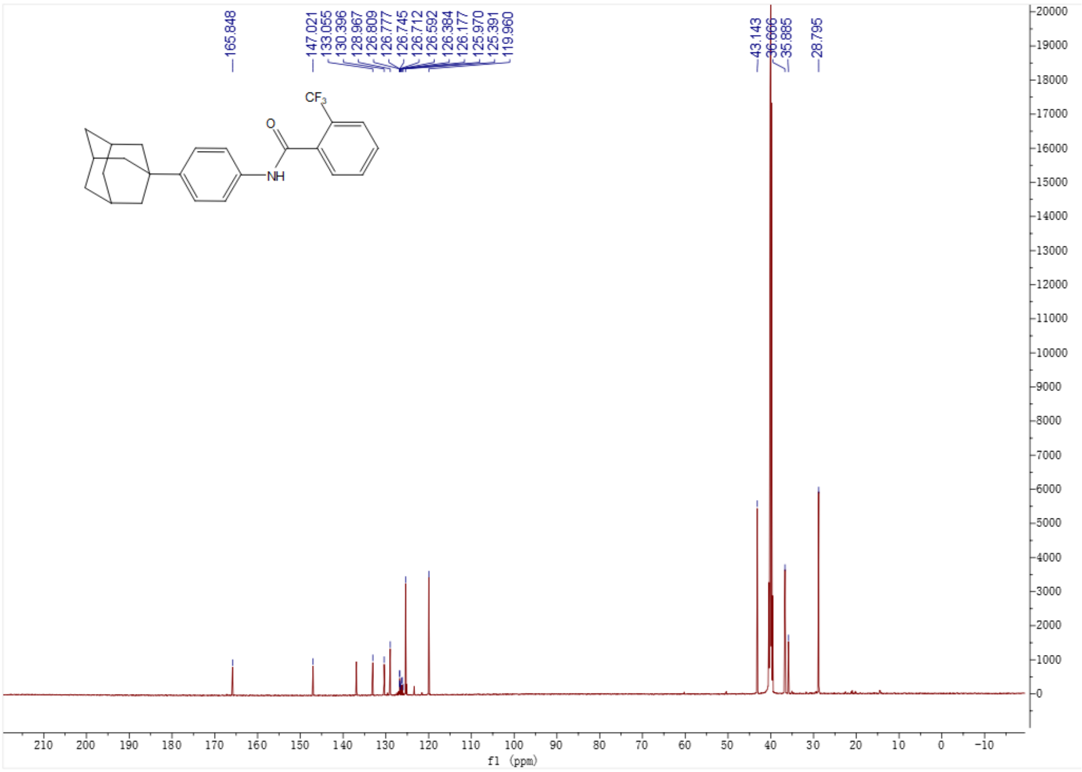

^{13}C NMR

*N-(4-((3r,5r,7r)-adamantan-1-yl)phenyl)-3-(trifluoromethyl)benzamide(**1-3ae**)*

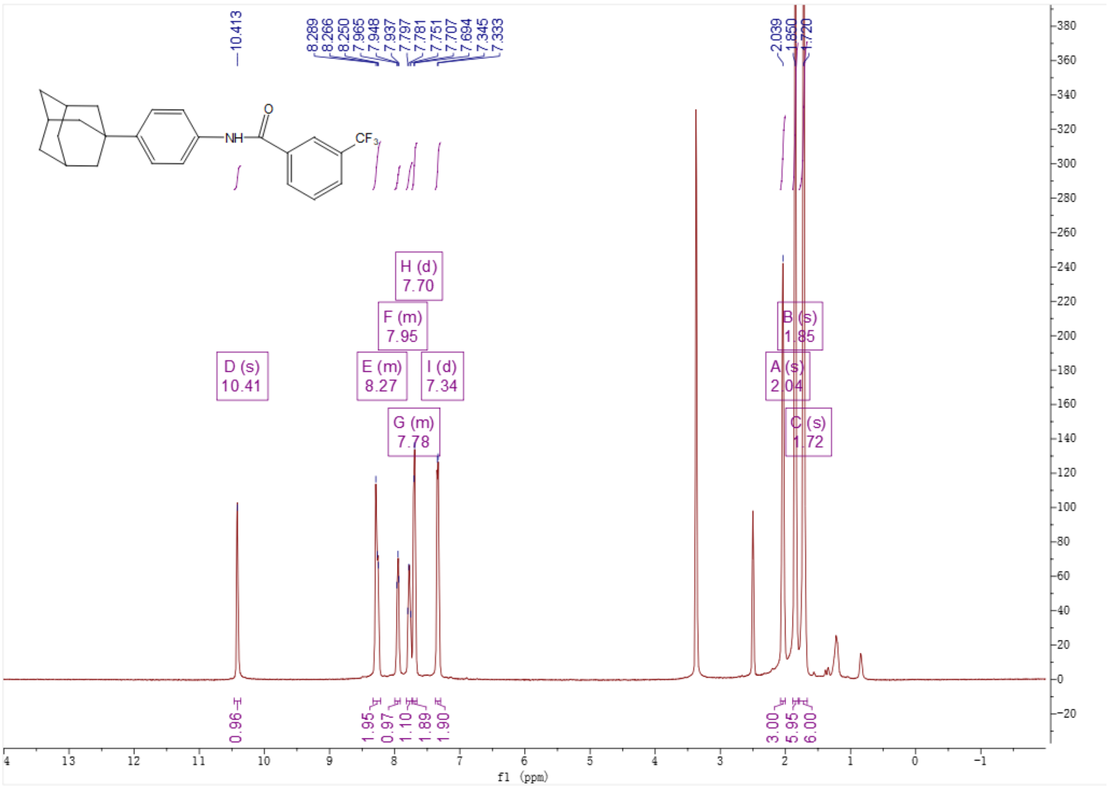

^{1}H NMR

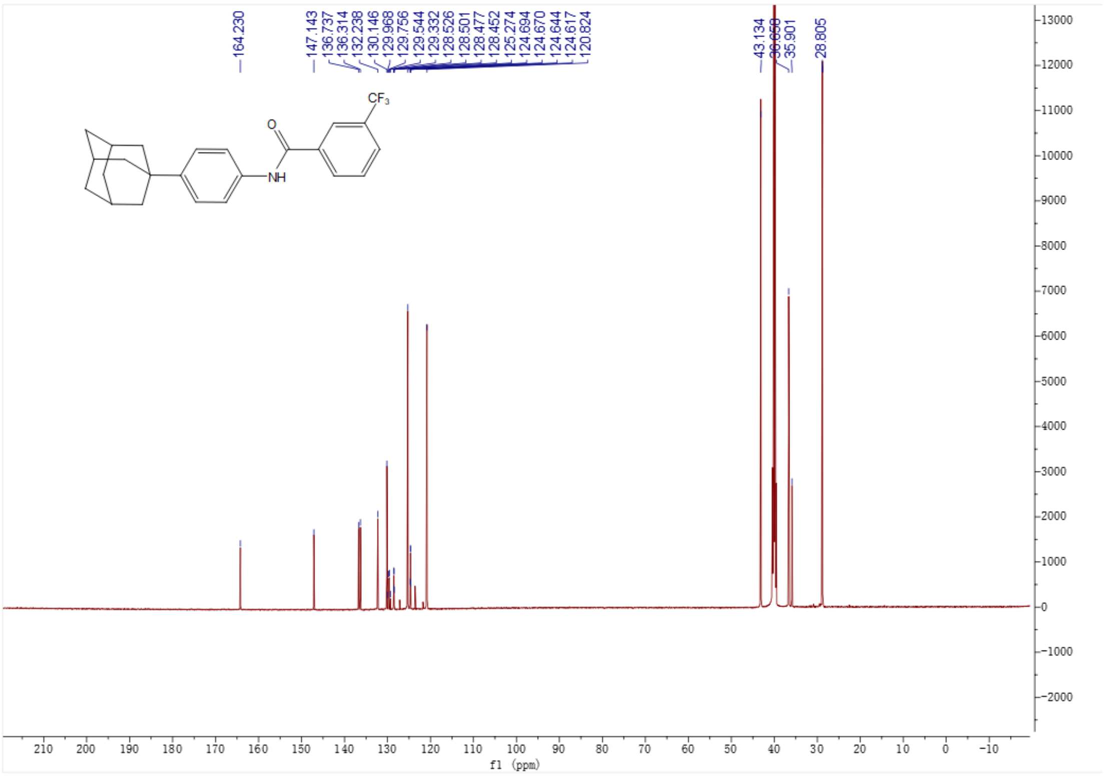

^{13}C NMR

*N-(4-((3r,5r,7r)-adamantan-1-yl)phenyl)-4-(trifluoromethyl)benzamide(**1-3af**)*

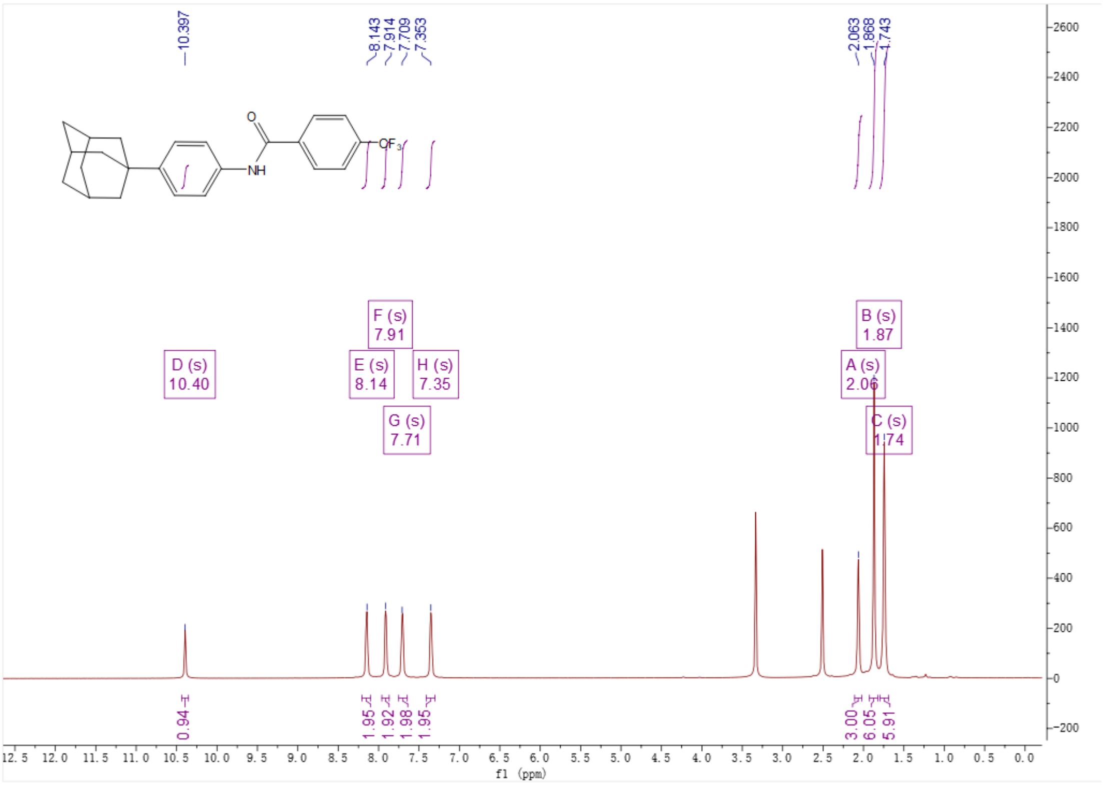

^{1}H NMR

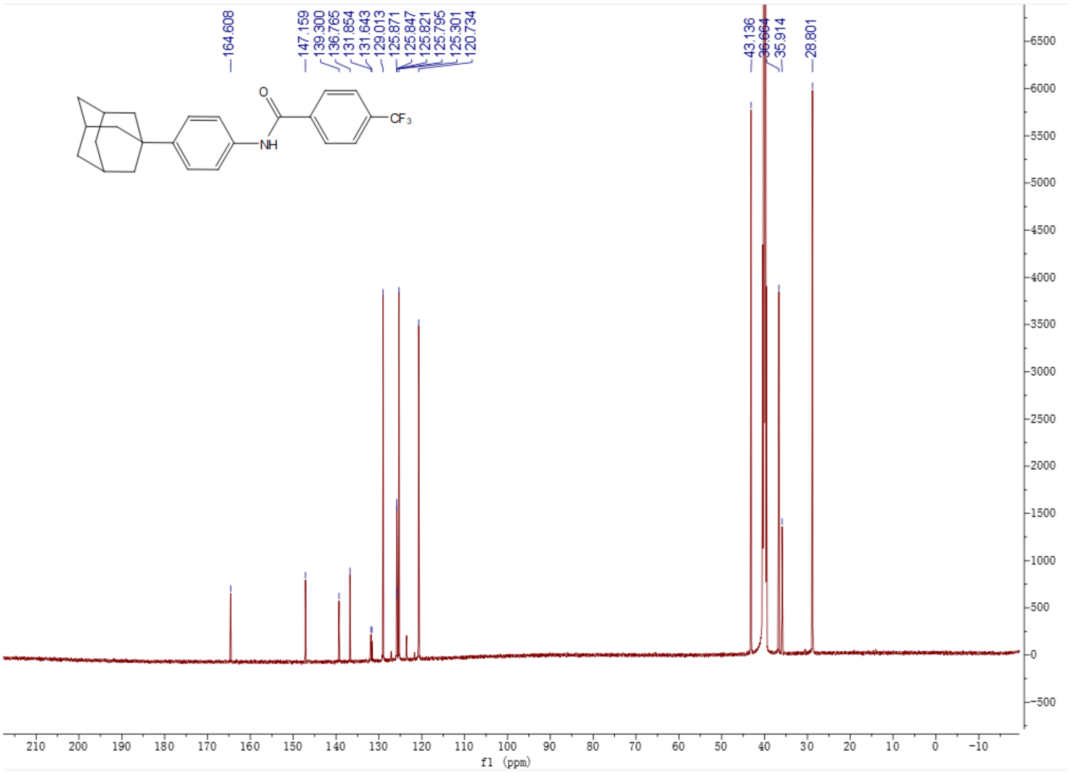

^{13}C NMR

*N-(4-((3r,5r,7r)-adamantan-1-yl)phenyl)-4-morpholinobenzamide(**1-3ag**)*

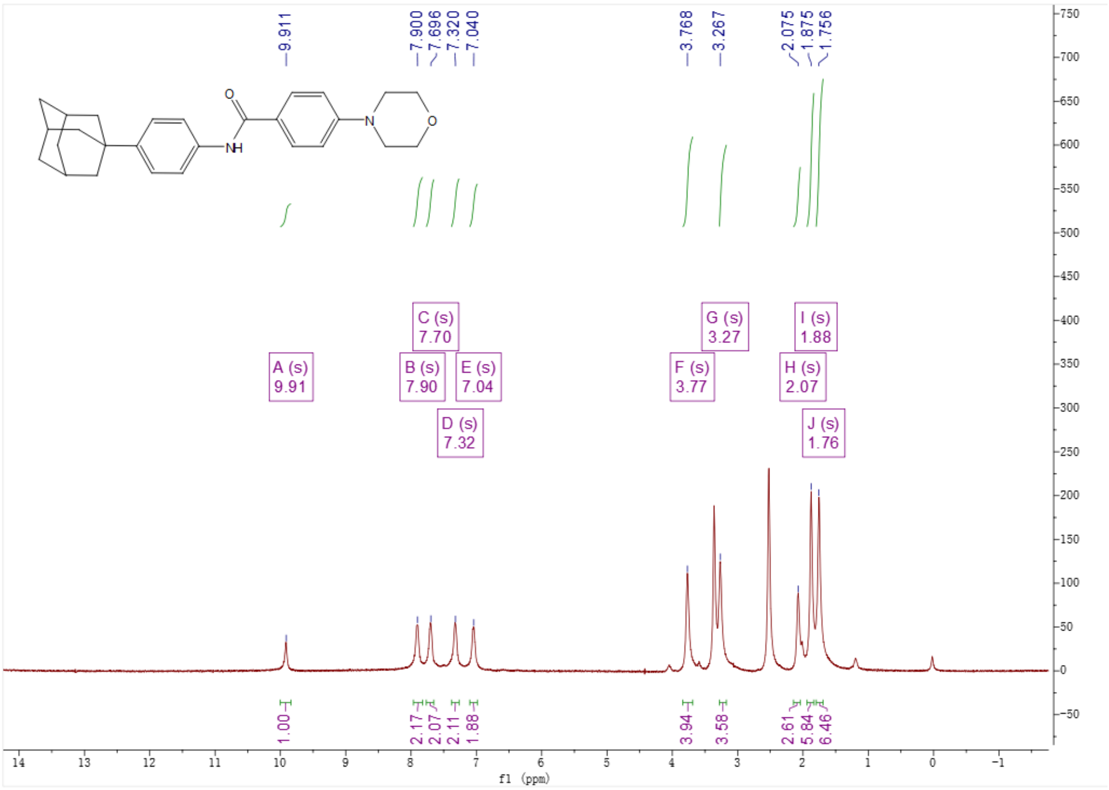

^{1}H NMR

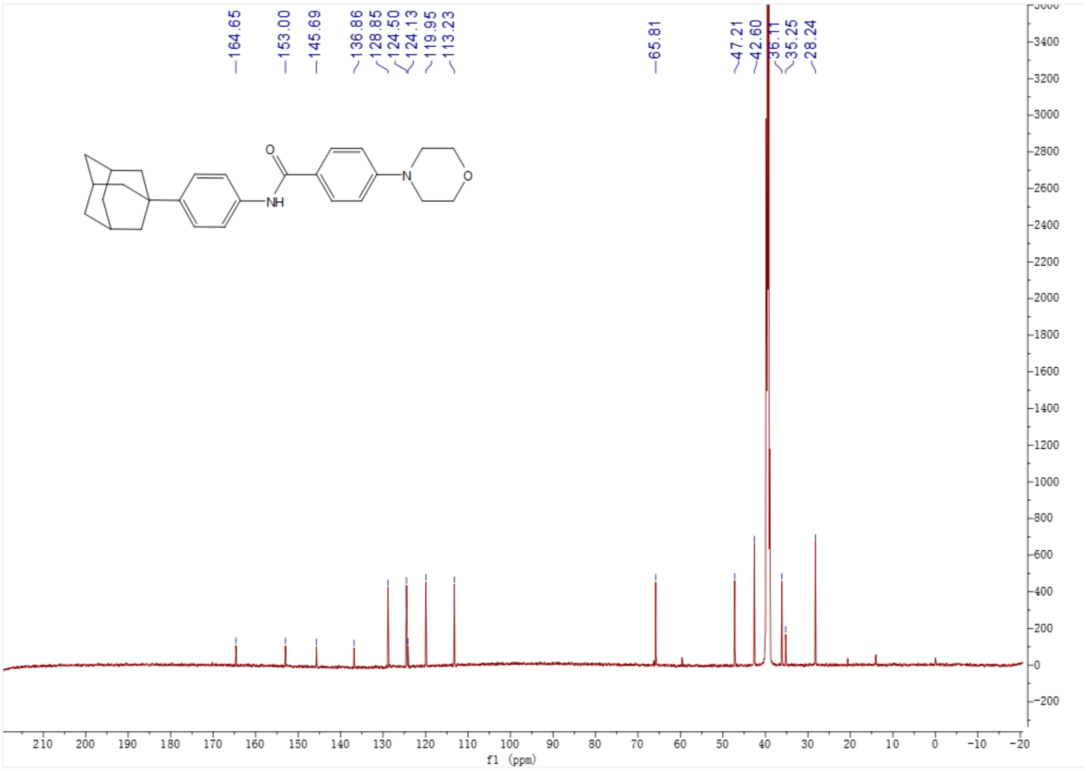

^{13}C NMR

*N-(4-((3r,5r,7r)-adamantan-1-yl)phenyl)-4-(pyrrolidin-1-yl)benzamide(**1-3ah**)*

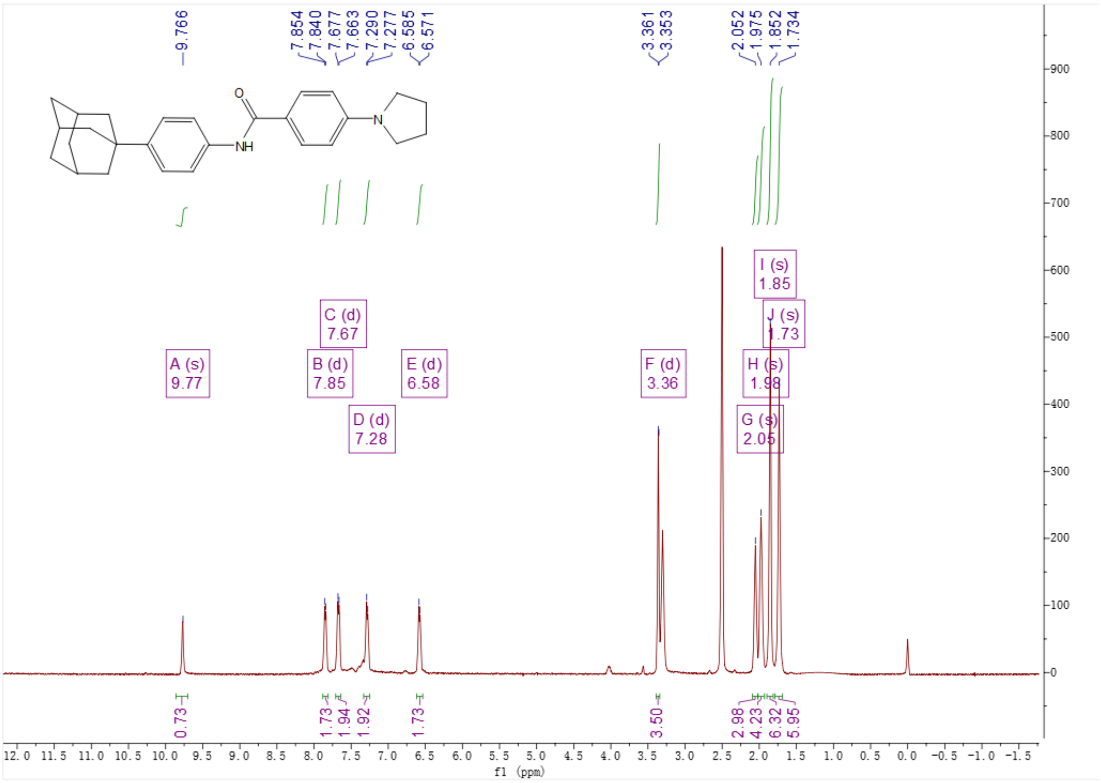

^{1}H NMR

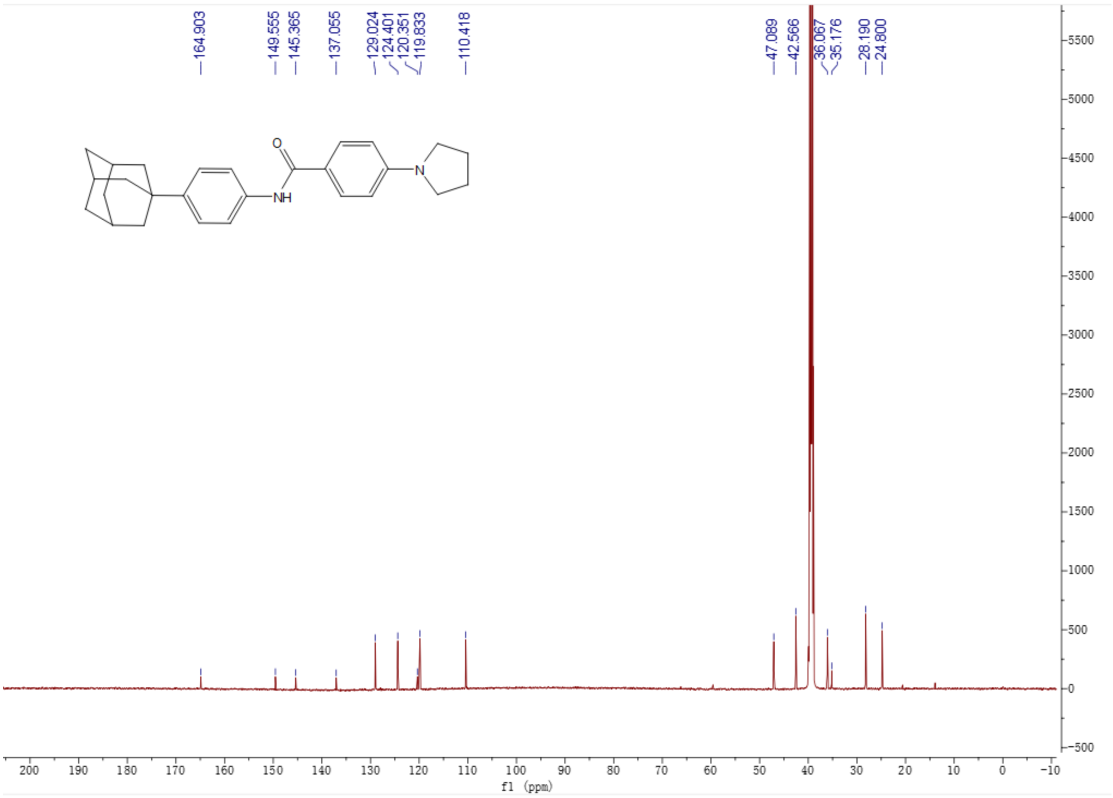

^{13}C NMR

*N-(4-((3r,5r,7r)-adamantan-1-yl)phenyl)-2-(hexyloxy)benzamide(**1-3ai**)*

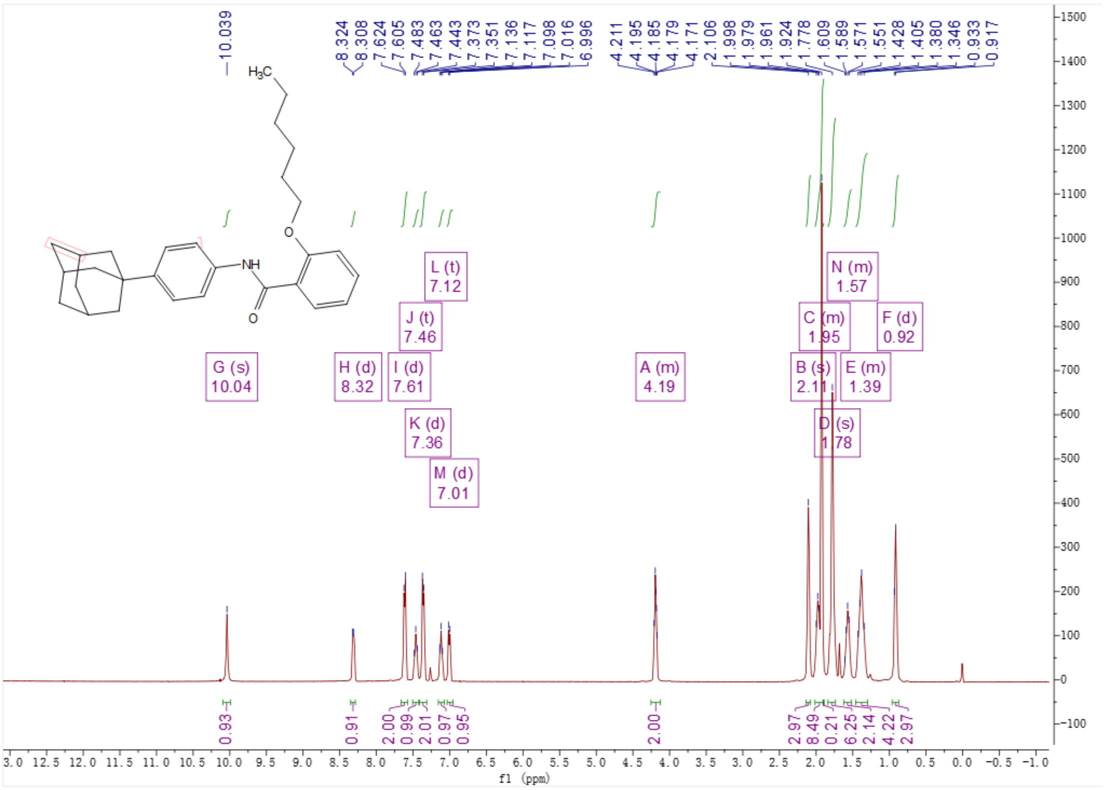

^{1}H NMR

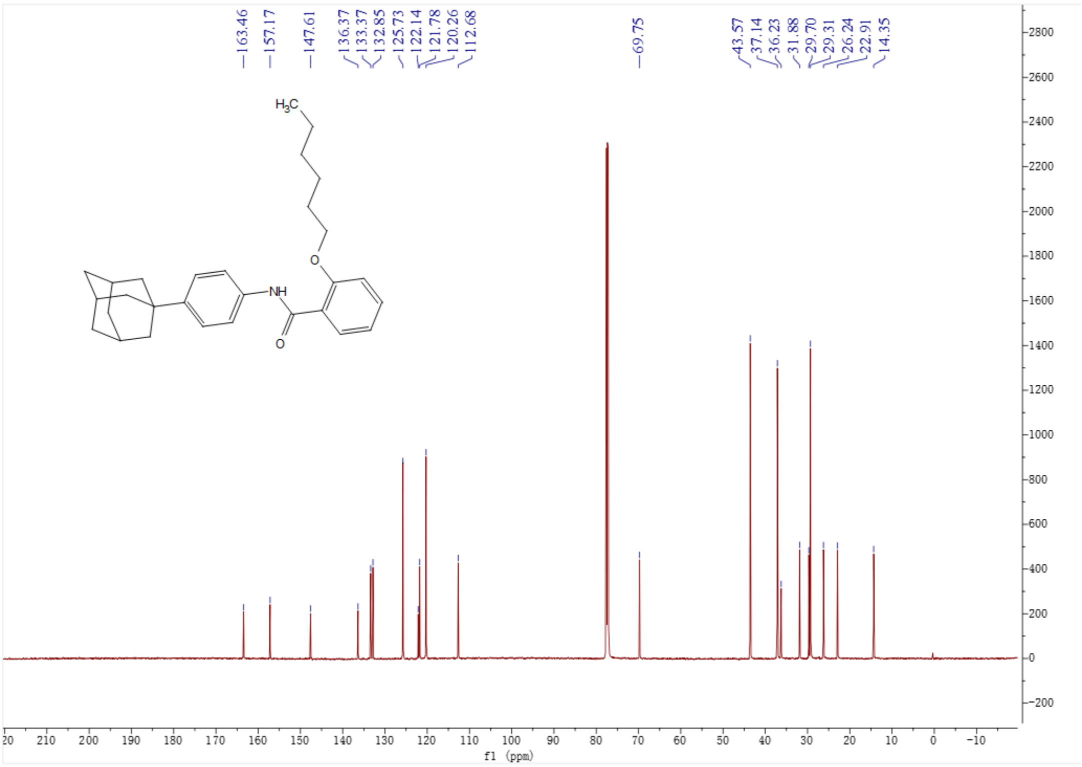

^{13}C NMR

*N-(4-((3r,5r,7r)-adamantan-1-yl)phenyl)-3-(hexyloxy)benzamide(**1-3aj**)*

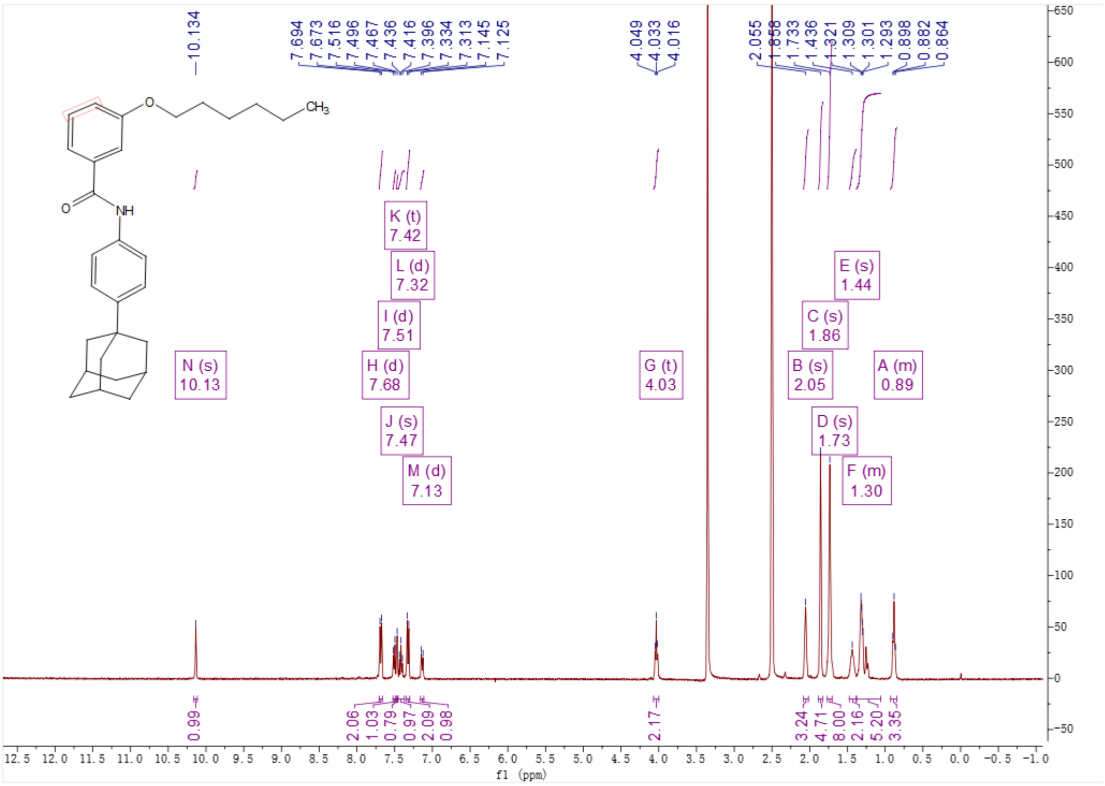

^{1}H NMR

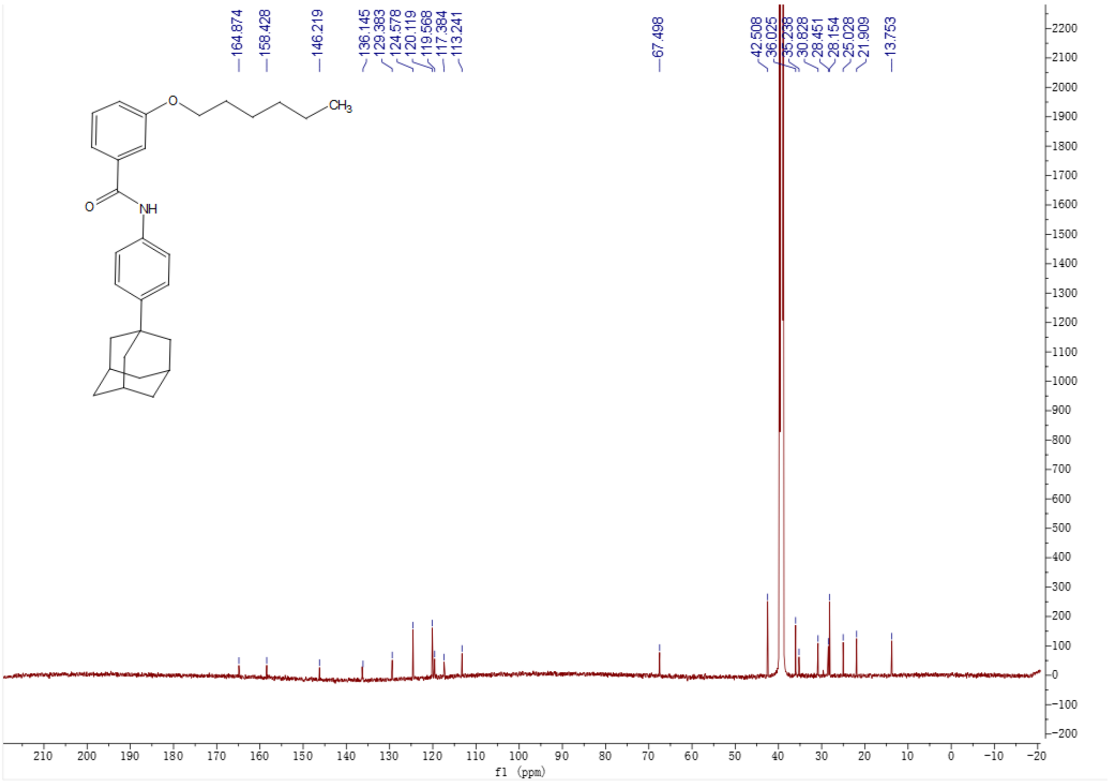

^{13}C NMR

*N-(4-((1s,3s)-adamantan-1-yl)phenyl)-4-octylbenzamide(**1-3ak**)*

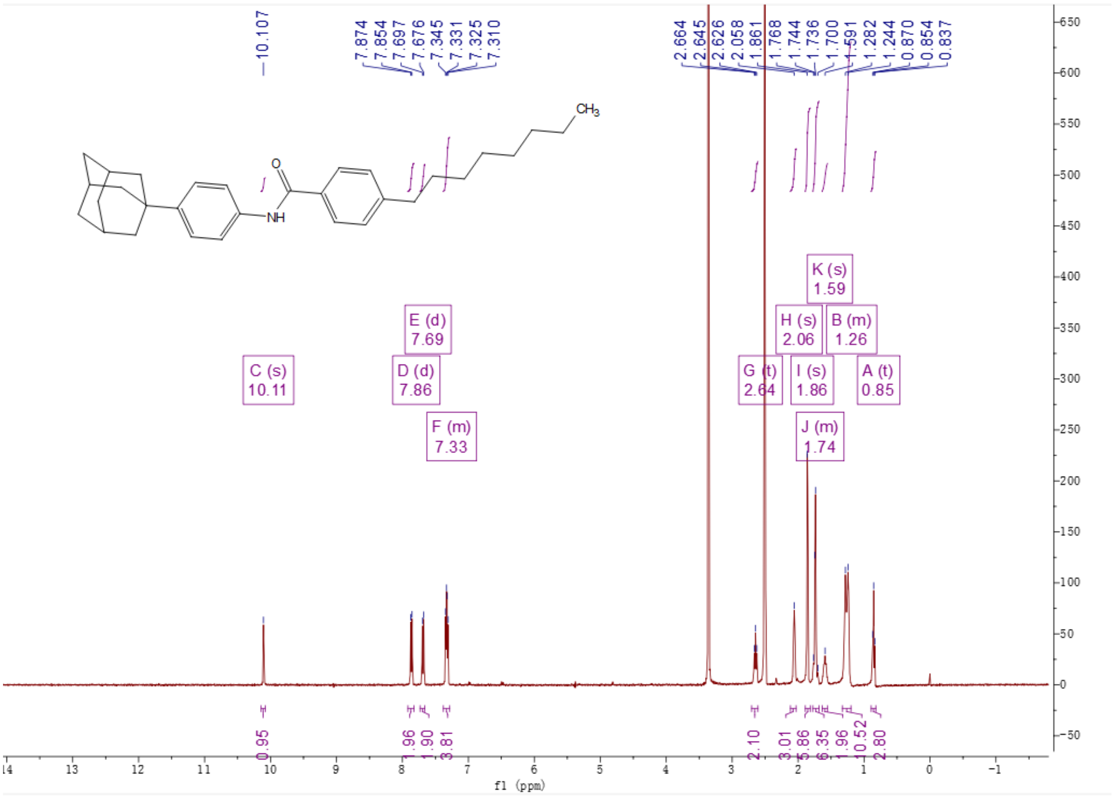

^{1}H NMR

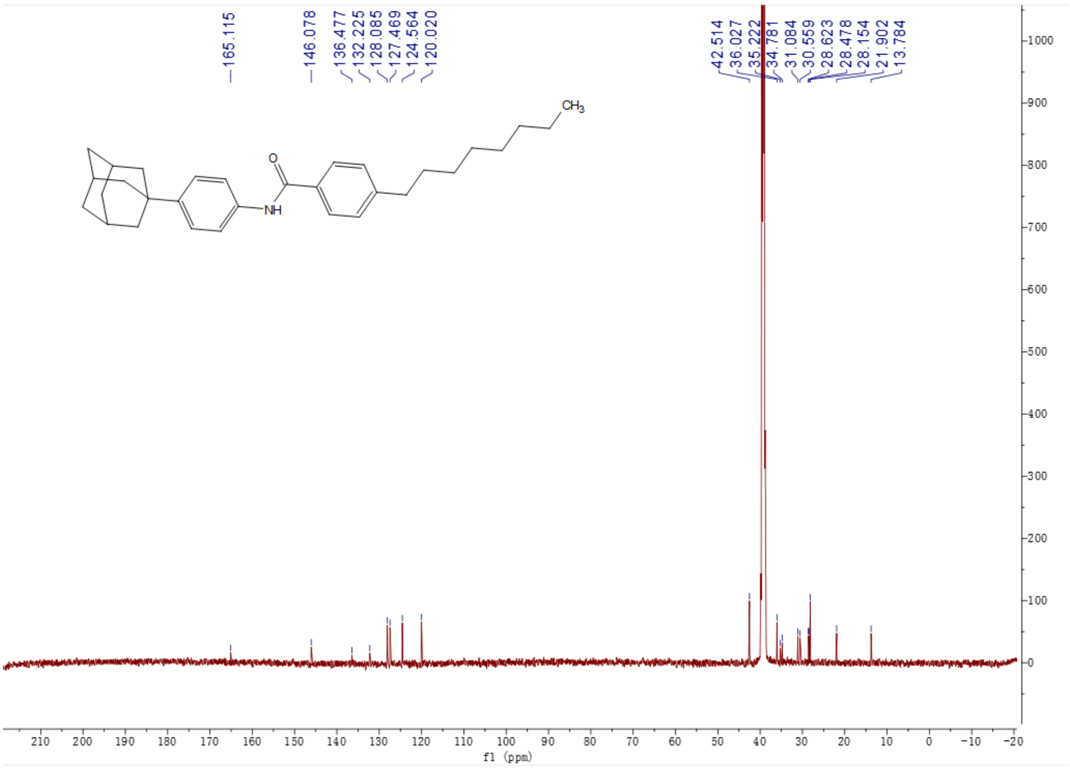

^{13}C NMR

*N-(4-((1s,3s)-adamantan-1-yl)phenyl)-3-(5-bromopentanamido)benzamide(**1-3al**)*

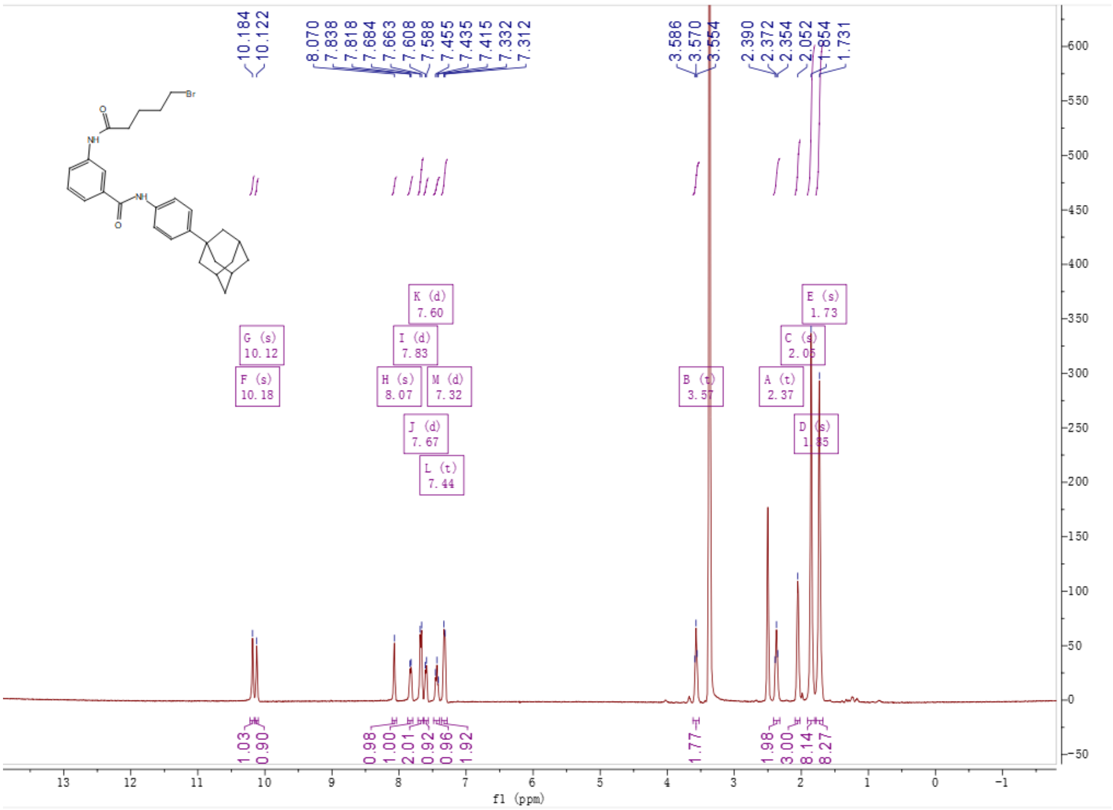

^{1}H NMR

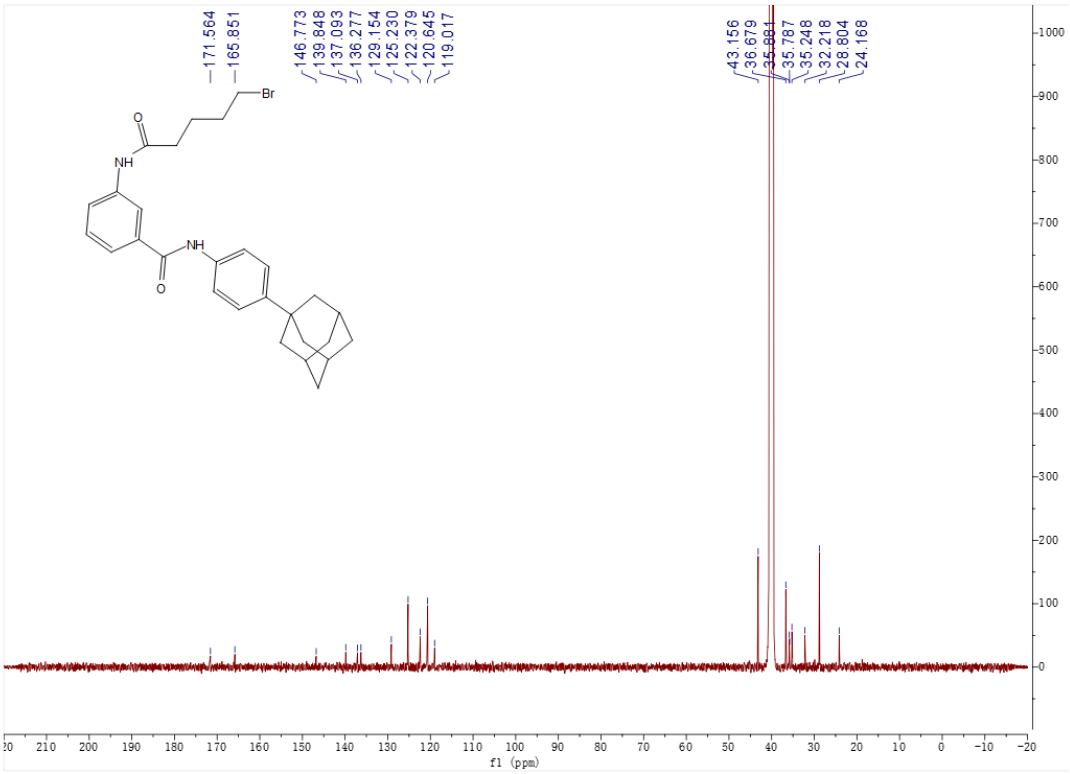

^{13}C NMR

*N-(4-((1s,3s)-adamantan-1-yl)phenyl)-3-(5-(4-methylpiperazin-1-yl)pentanamido)benzamide(**1-3am**)*

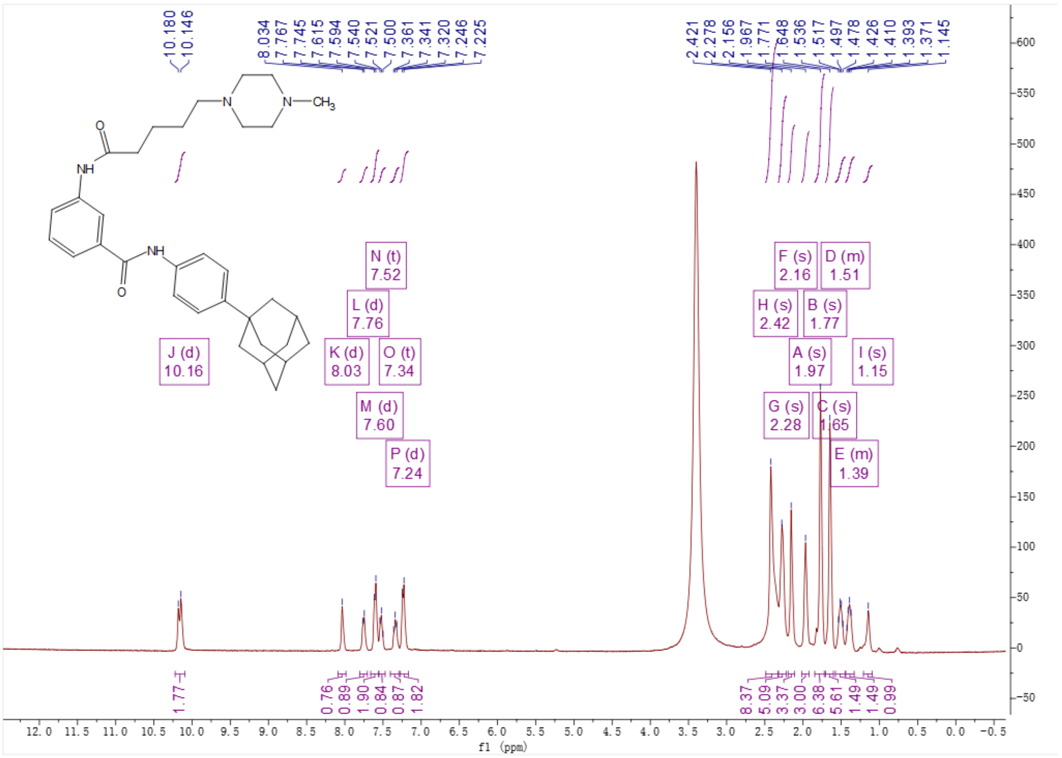

^{1}H NMR

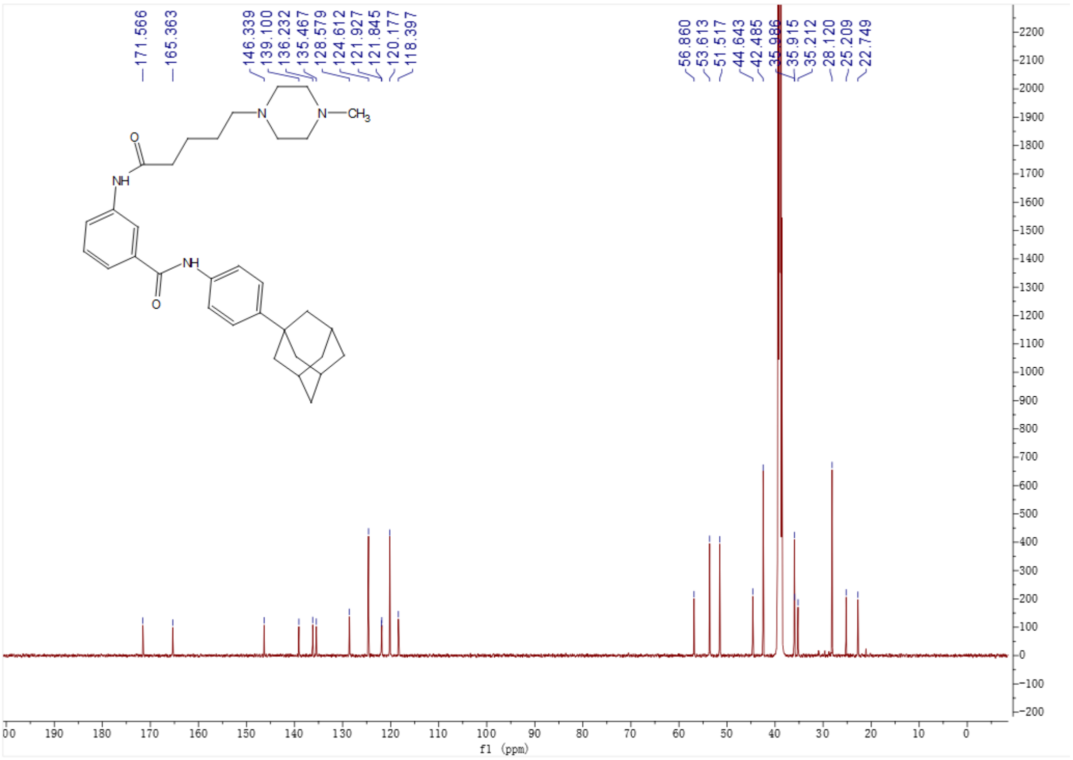

^{13}C NMR

*N-(4-((1s,3s)-adamantan-1-yl)phenyl)-3-(5-morpholinopentanamido)benzamide(**1-3an**)*

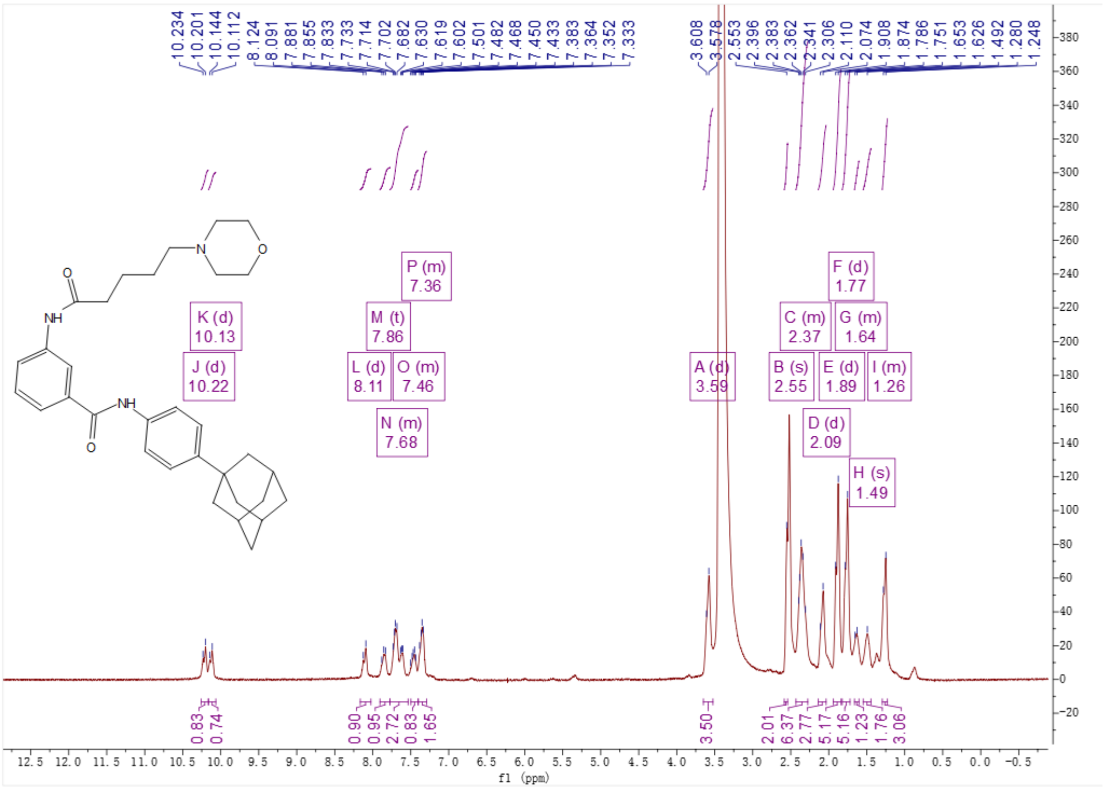

^{1}H NMR

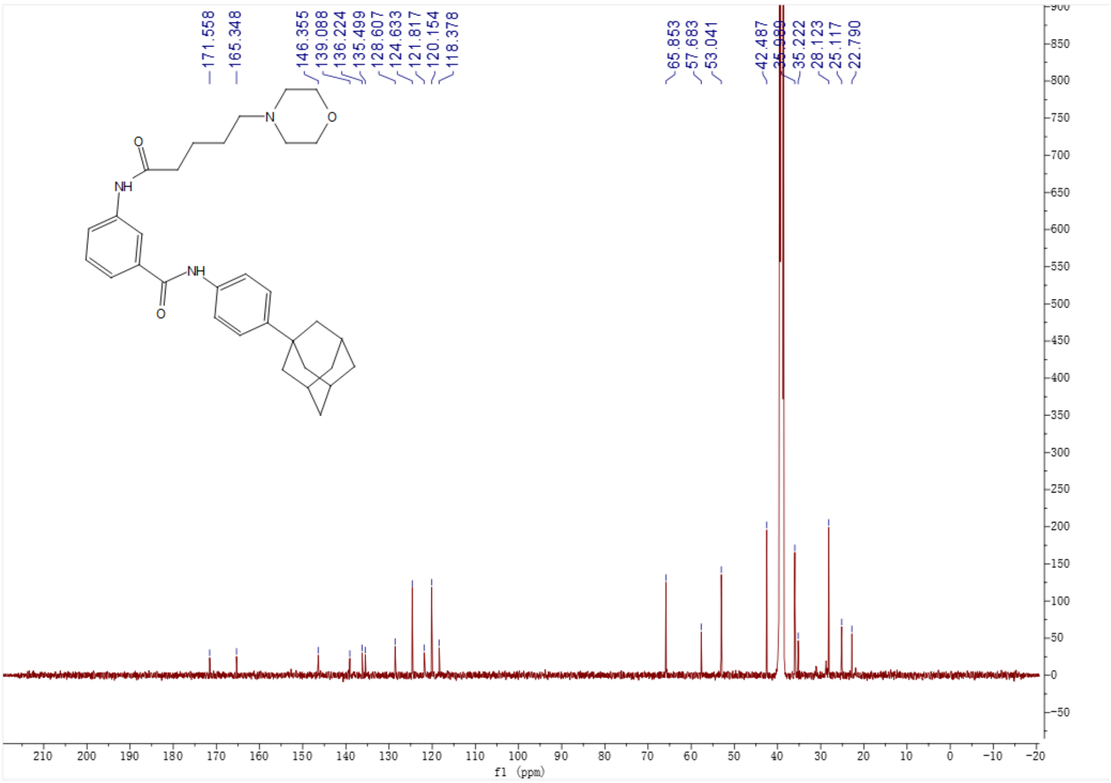

^{13}C NMR

*N-(4-((3r,5r,7r)-adamantan-1-yl)phenyl)-4-(5-bromopentanamido)benzamide(**1-3ao**)*

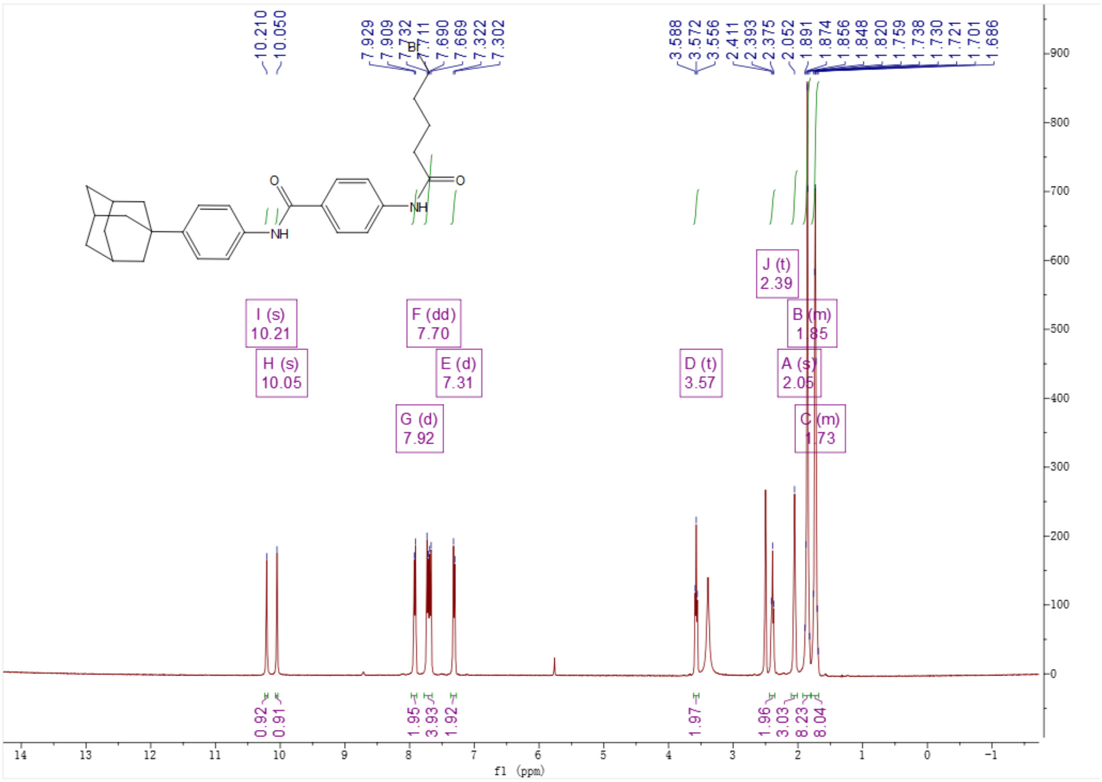

^{1}H NMR

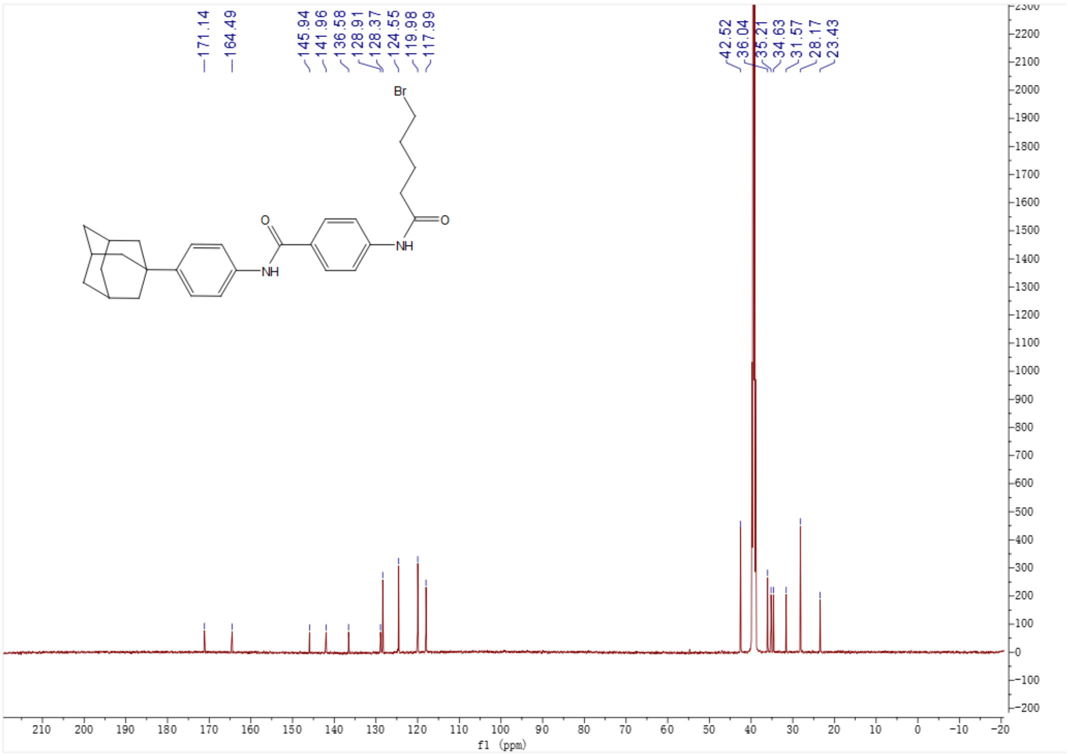

^{13}C NMR

*N-(4-((3r,5r,7r)-adamantan-1-yl)phenyl)-4-(5-(4-methylpiperazin-1-yl)pentanamido)benzamide(**1-3ap**)*

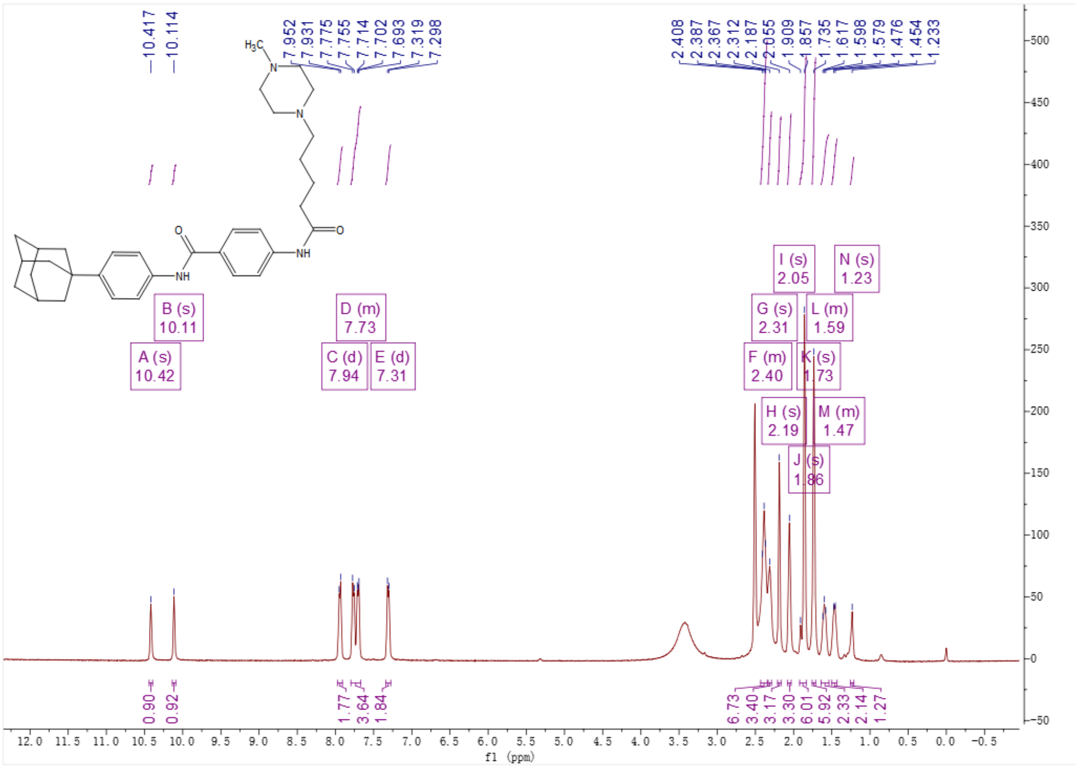

^{1}H NMR

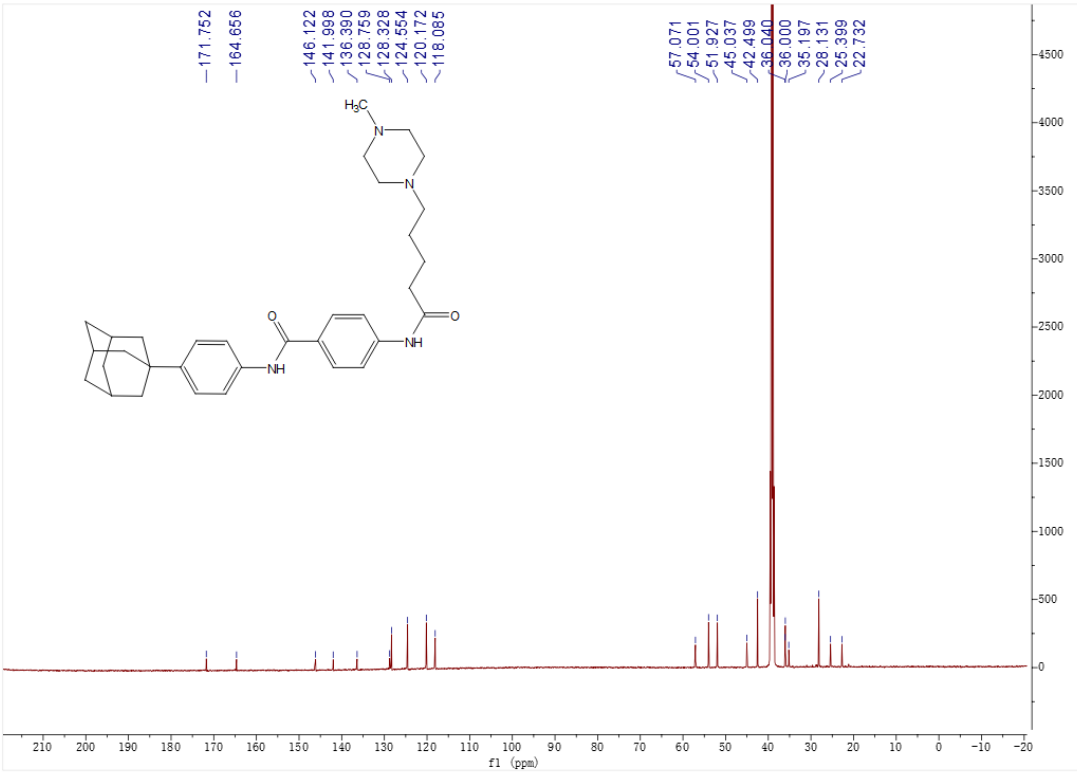

^{13}C NMR

*N-(4-((3r,5r,7r)-adamantan-1-yl)phenyl)-4-(5-morpholinopentanamido)benzamide(**1-3aq**)*

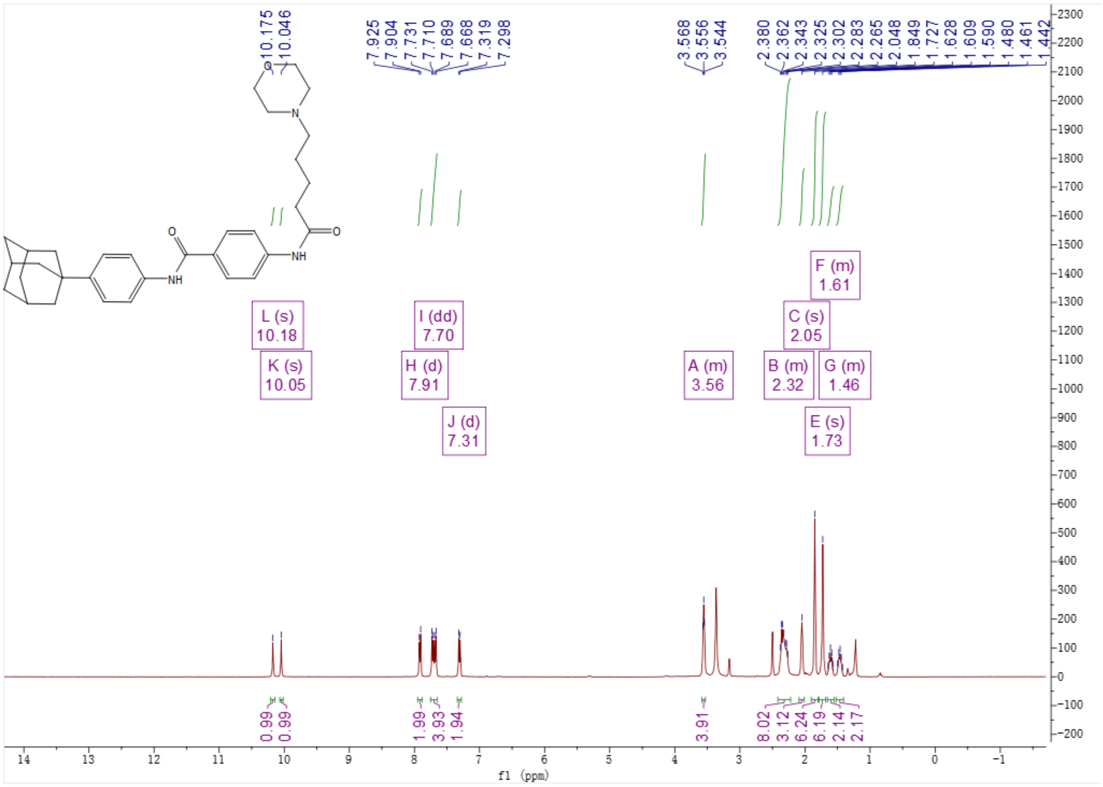

^{1}H NMR

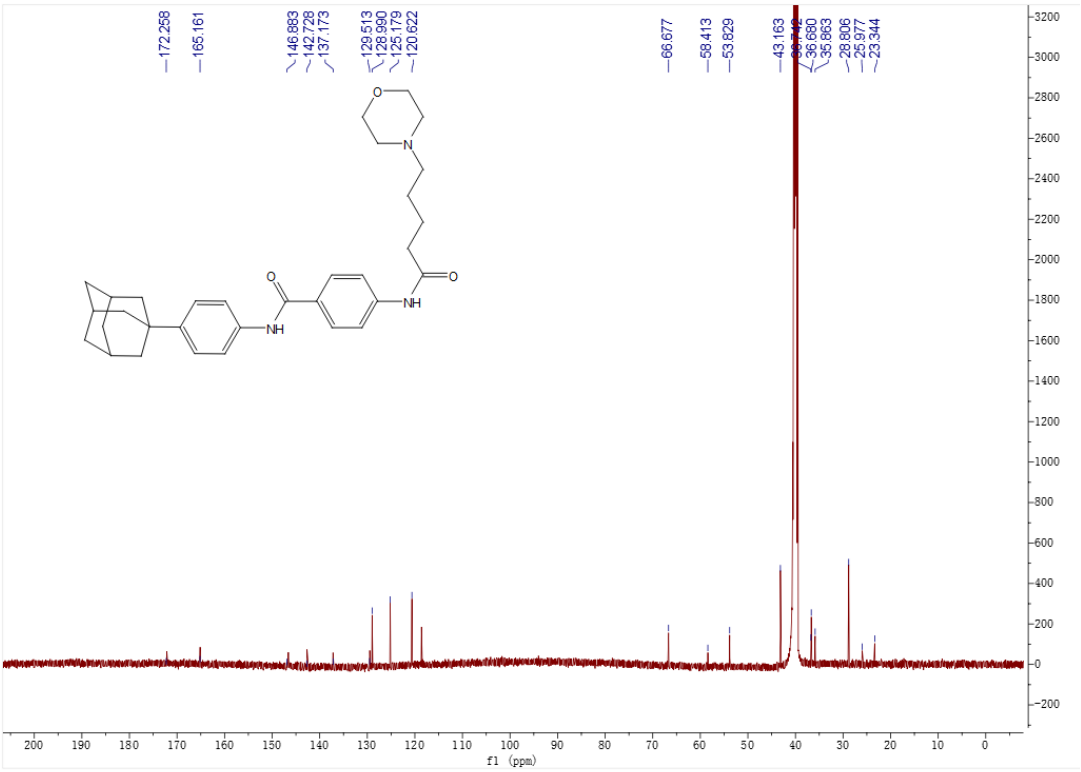

^{13}C NMR

*N-(4-((3r,5r,7r)-adamantan-1-yl)phenyl)-3-(4-(dimethylamino)butanamido)benzamide(**1-3ar**)*

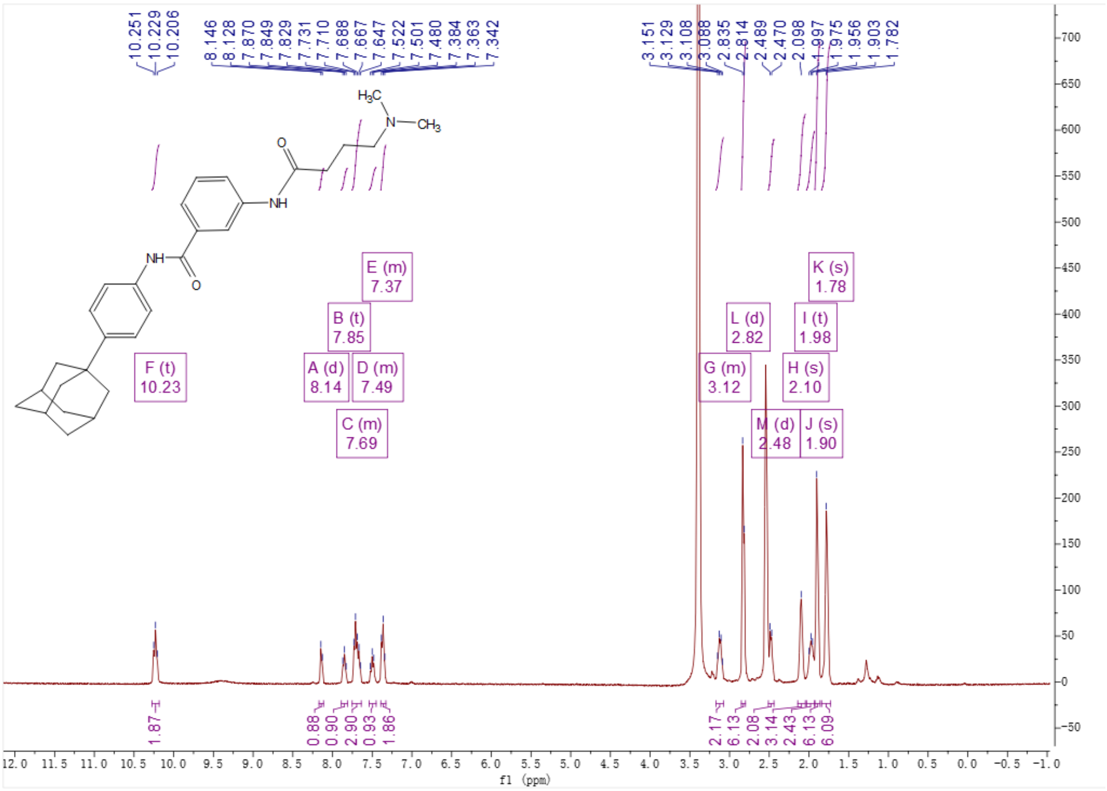

^{1}H NMR

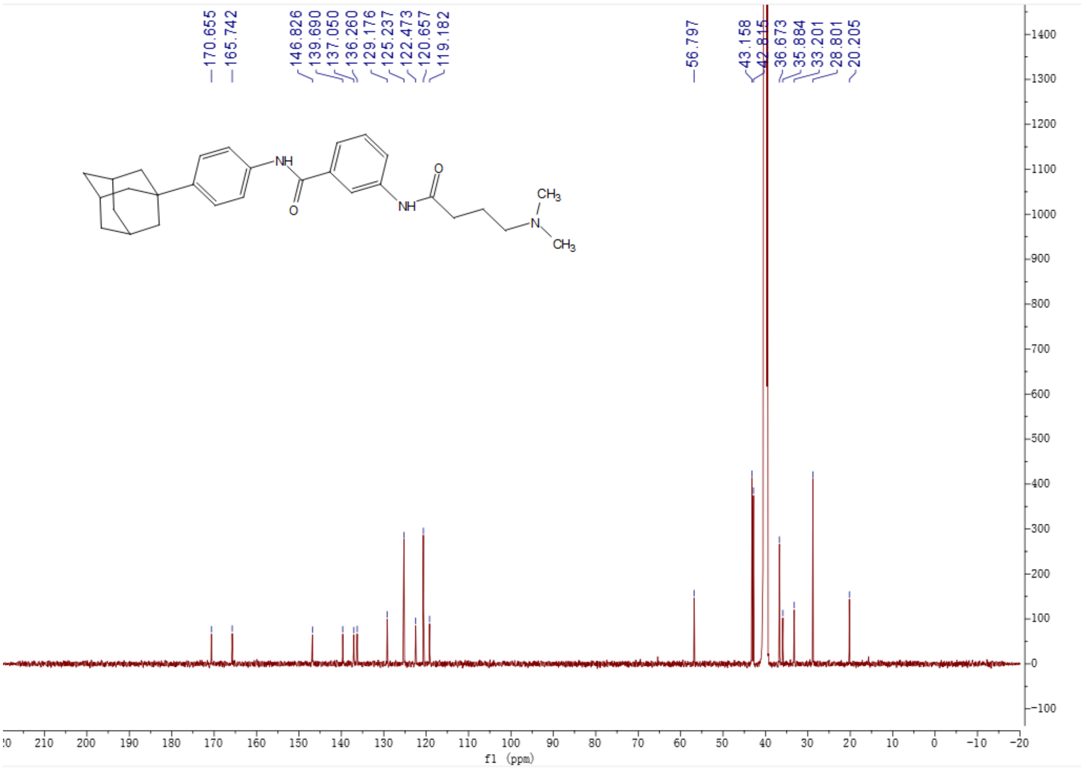

^{13}C NMR

*4-((3-((4-((3r,5r,7r)-adamantan-1-yl)phenyl)carbamoyl)phenyl)amino)-N,N,N-trimethyl-4-oxobutan-1-aminium(**1-3as**)*

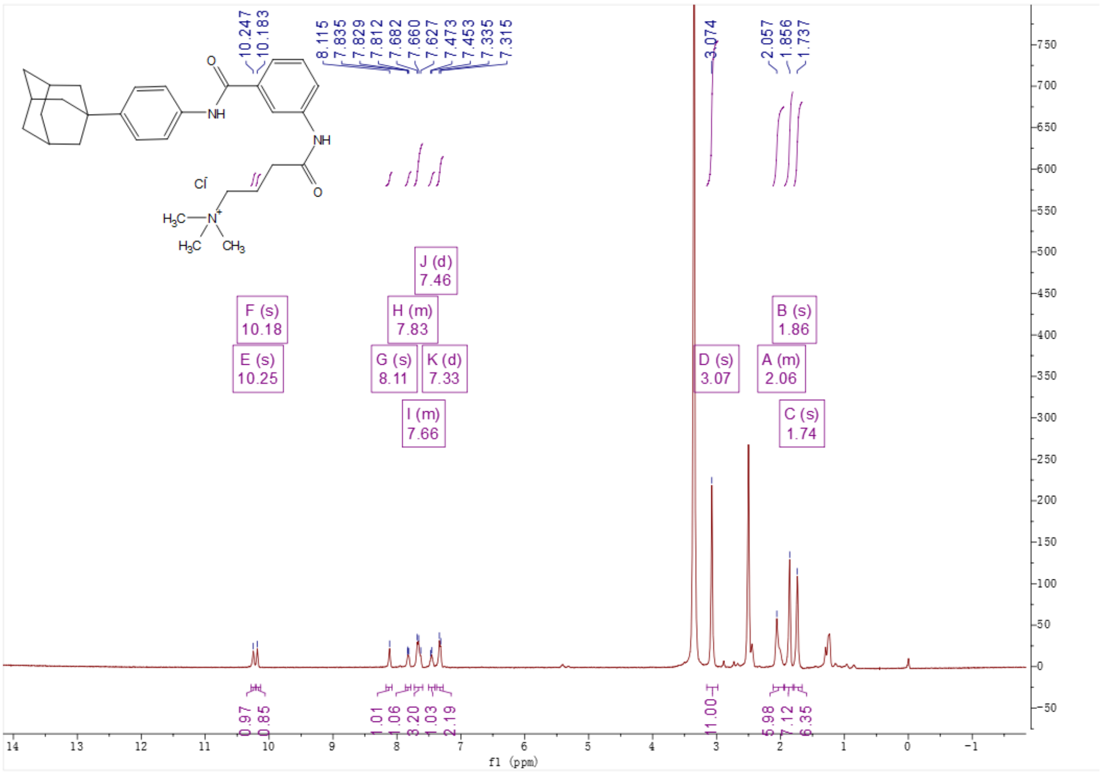

^{1}H NMR

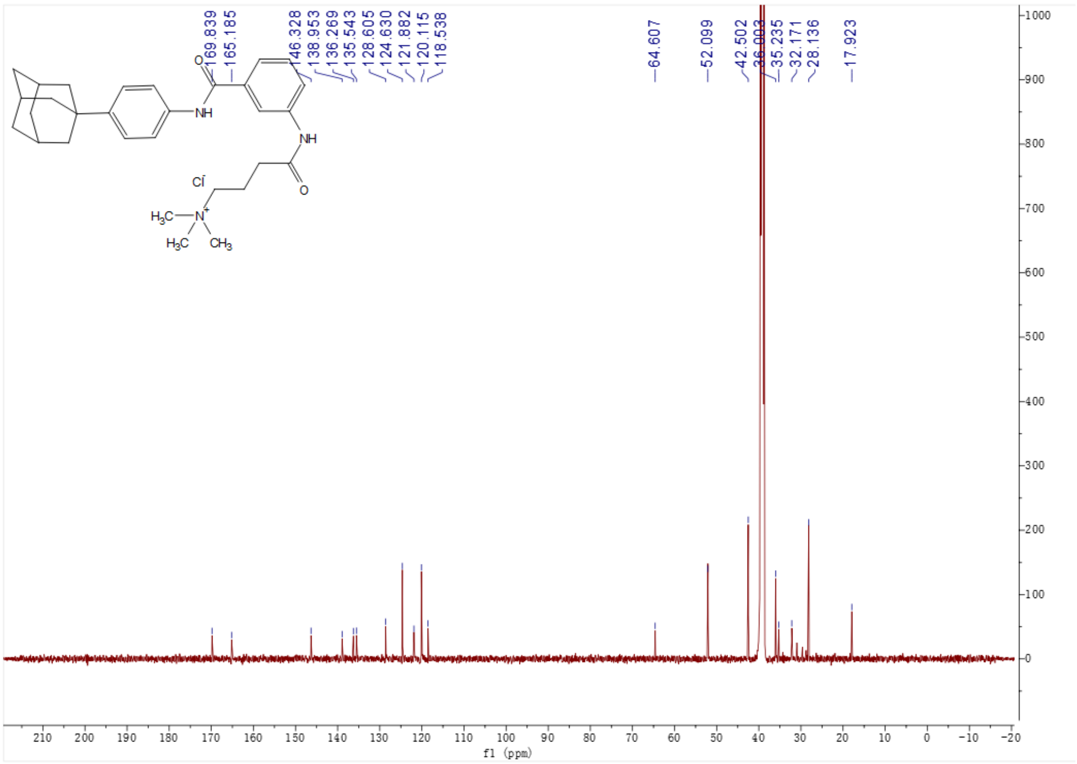

^{13}C NMR

*N-(4-((3r,5r,7r)-adamantan-1-yl)phenyl)-4-(4-(dimethylamino)butanamido)benzamide(**1-3at**)*

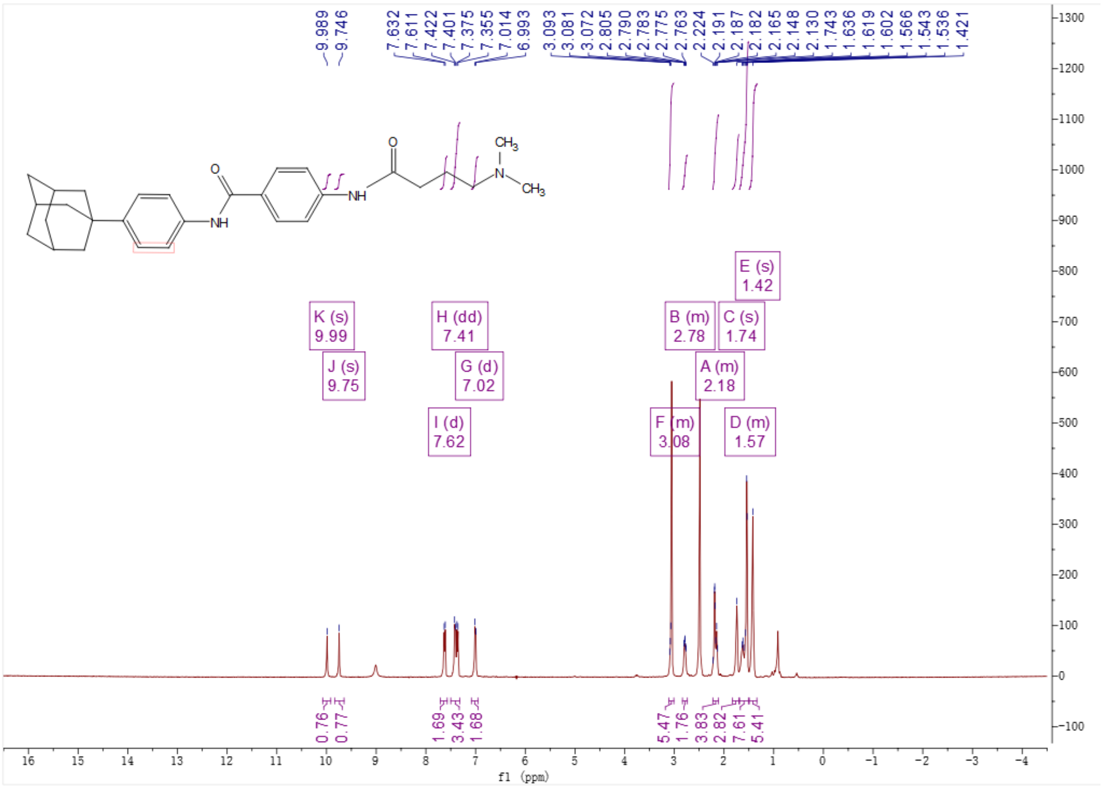

^{1}H NMR

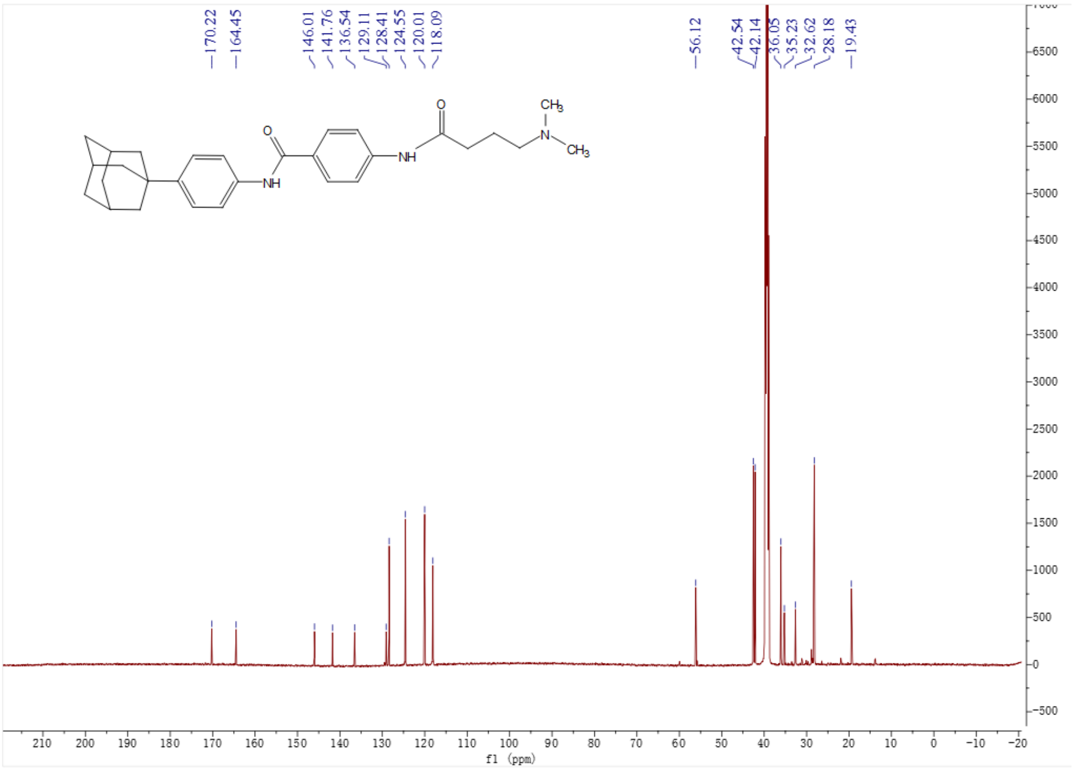

^{13}C NMR

*4-((4-((4-((3r,5r,7r)-adamantan-1-yl)phenyl)carbamoyl)phenyl)amino)-N,N,N-trimethyl-4-oxobutan-1-aminium(**1-3au, HI-104**)*

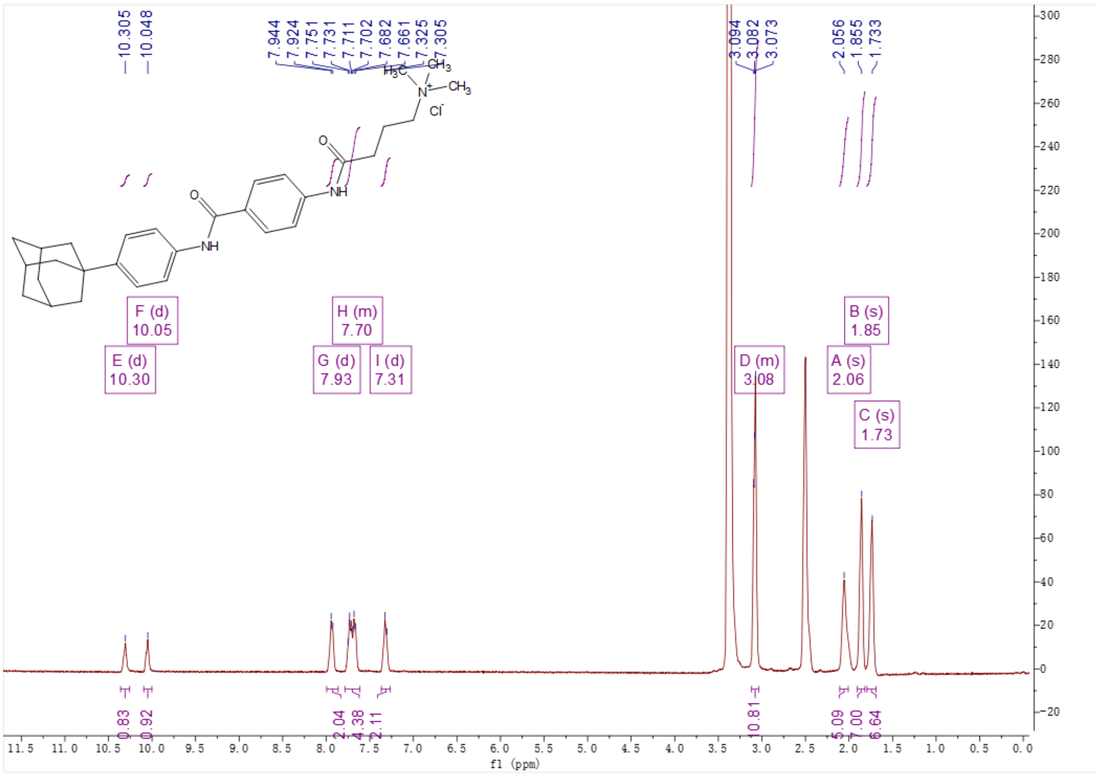

^{1}H NMR

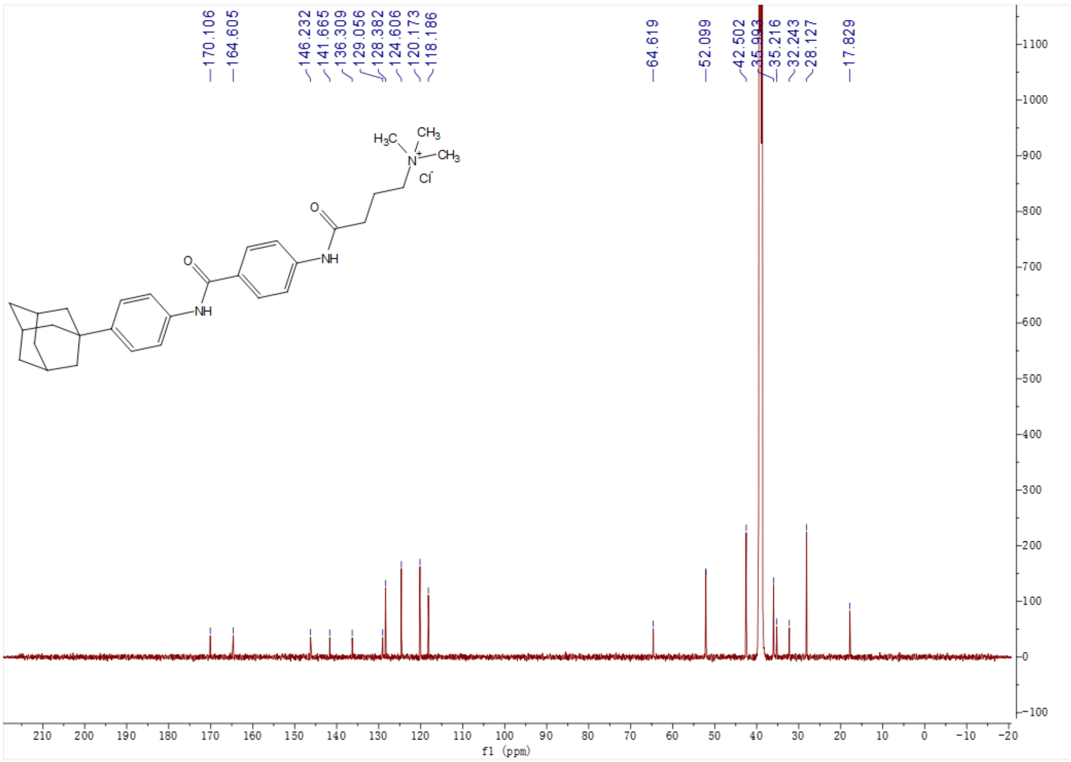

^{13}C NMR

*N-(4-((3r,5r,7r)-adamantan-1-yl)phenyl)picolinamide(**1-4a**)*

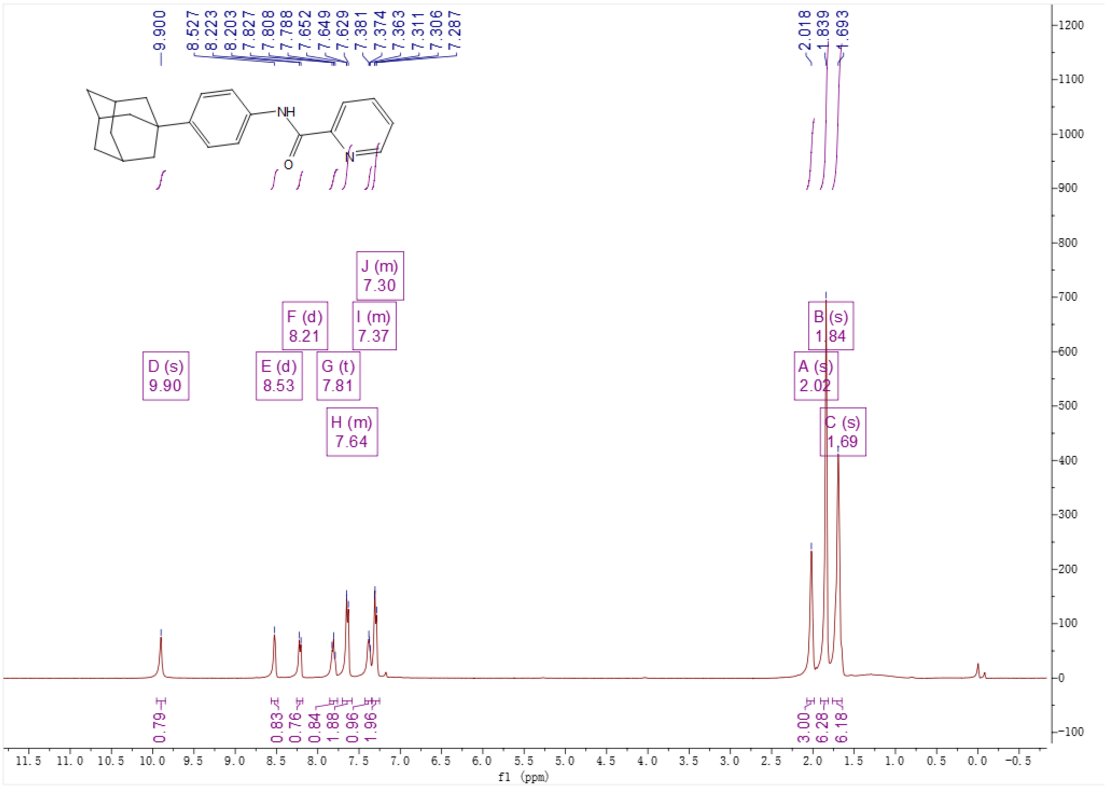

^{1}H NMR

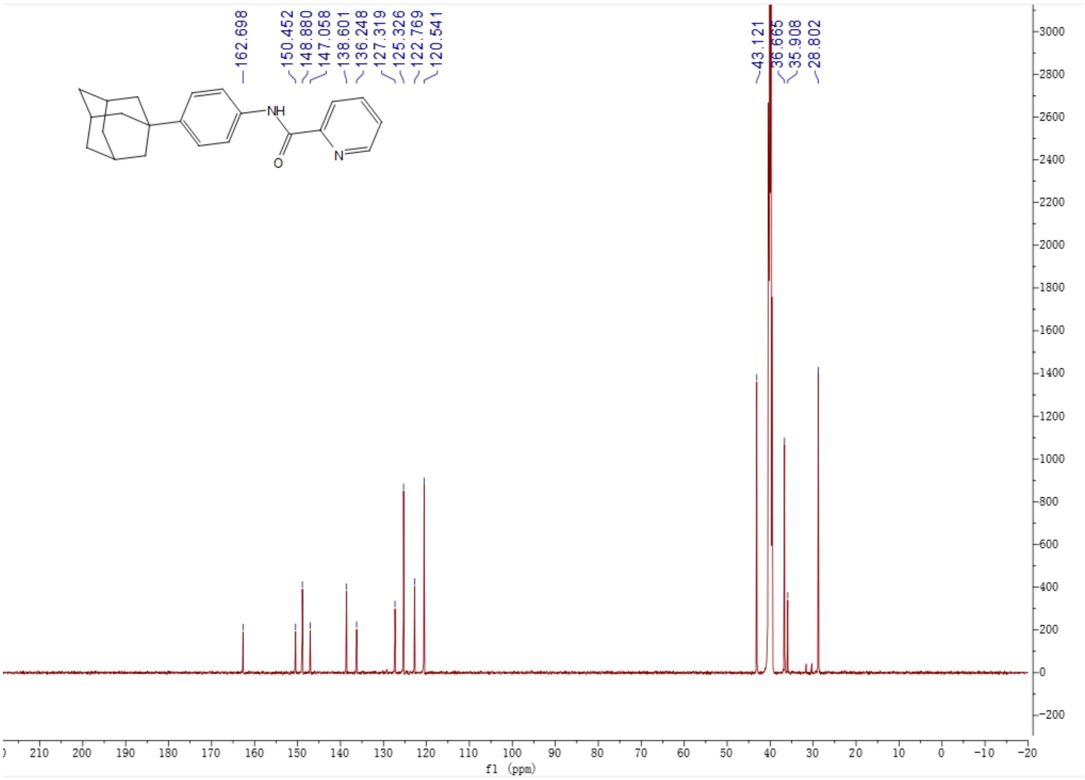

^{13}C NMR

*N-(4-((3r,5r,7r)-adamantan-1-yl)phenyl)nicotinamide (**1-4b**)*

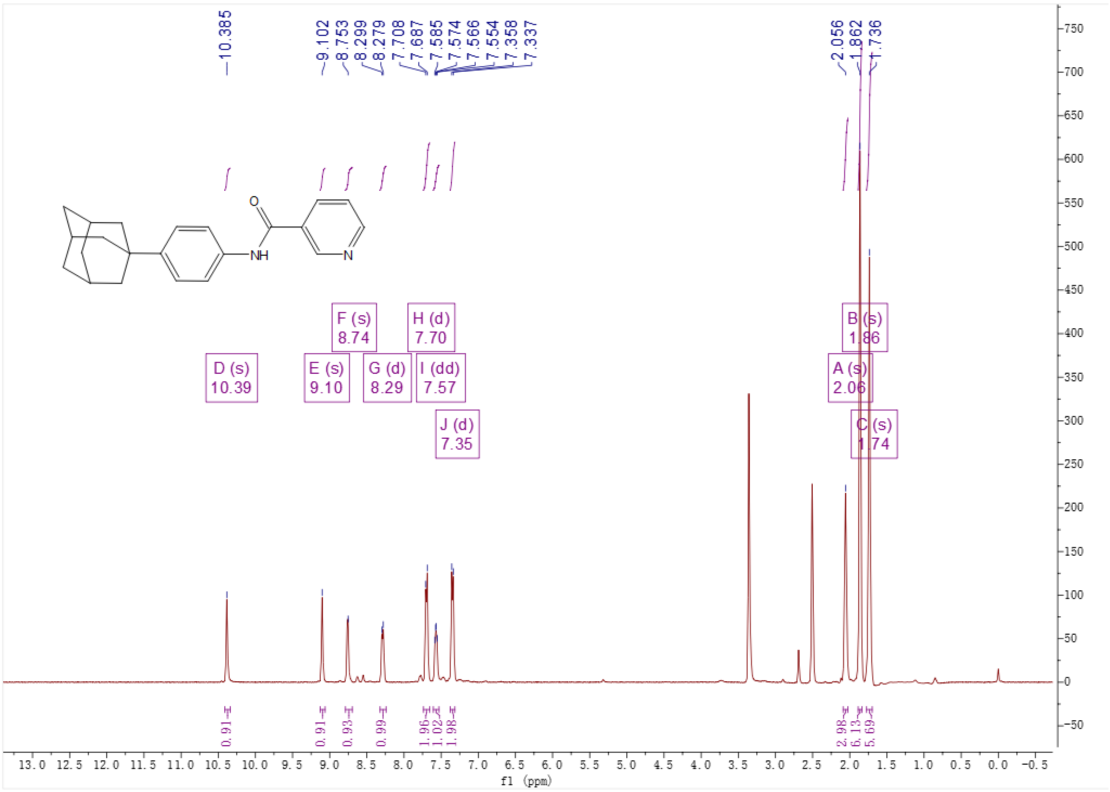

^{1}H NMR

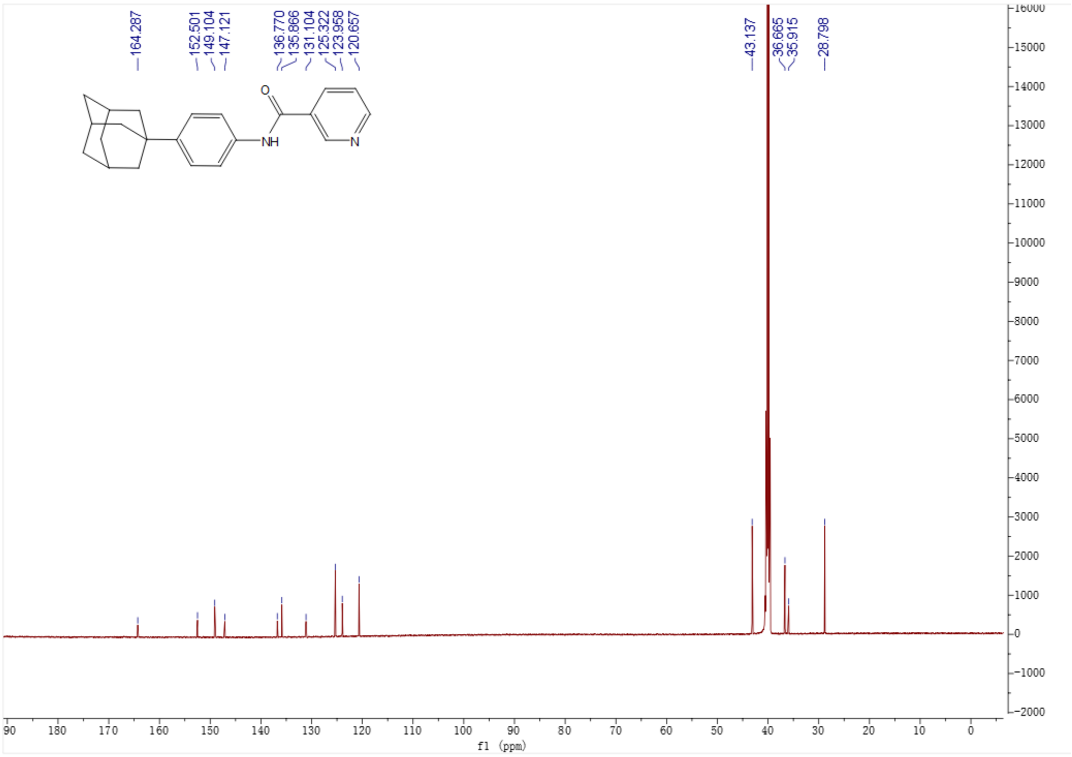

^{13}C NMR

*N-(4-((3r,5r,7r)-adamantan-1-yl)phenyl)isonicotinamide(**1-4c**)*

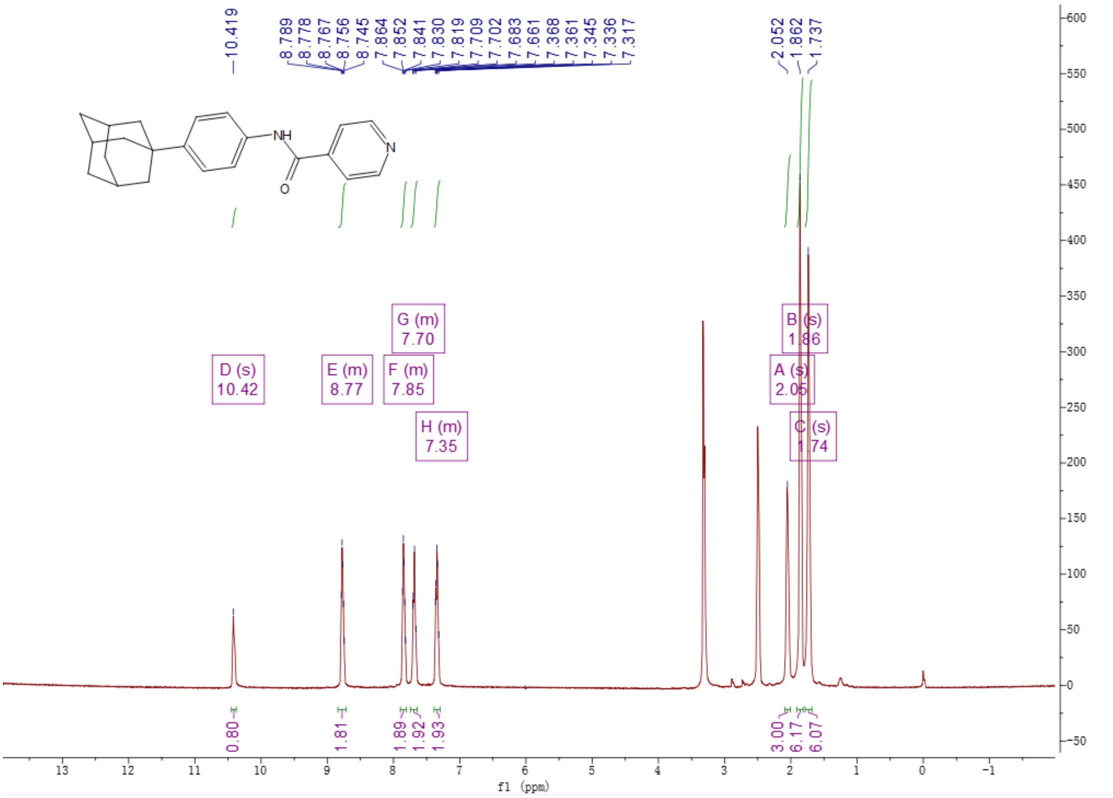

^{1}H NMR

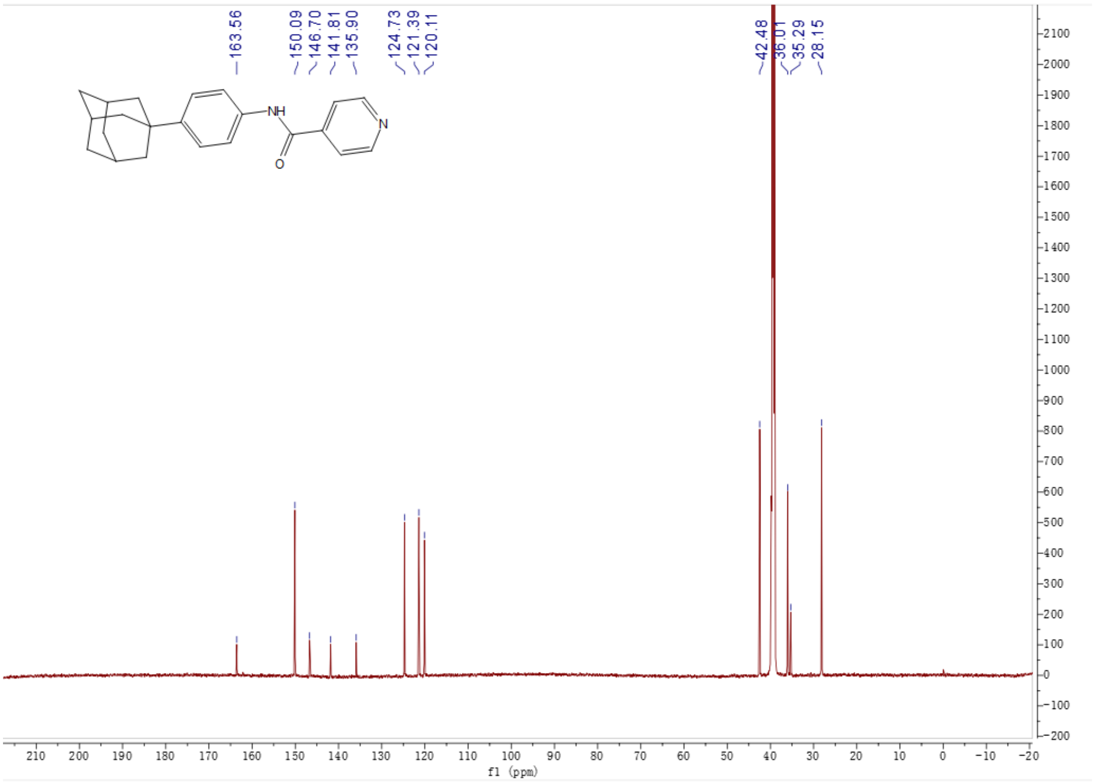

^{13}C NMR

*N-(4-((3r,5r,7r)-adamantan-1-yl)phenyl)pyrimidine-4-carboxamide(**1-4d**)*

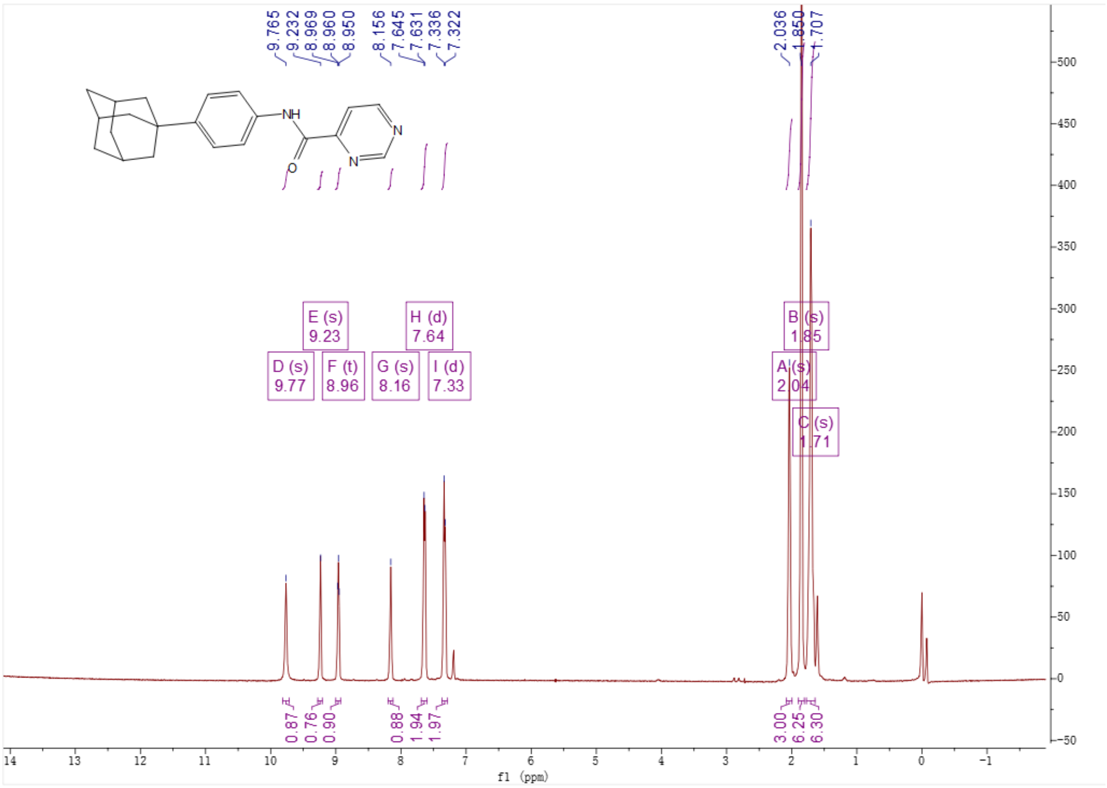

^{1}H NMR

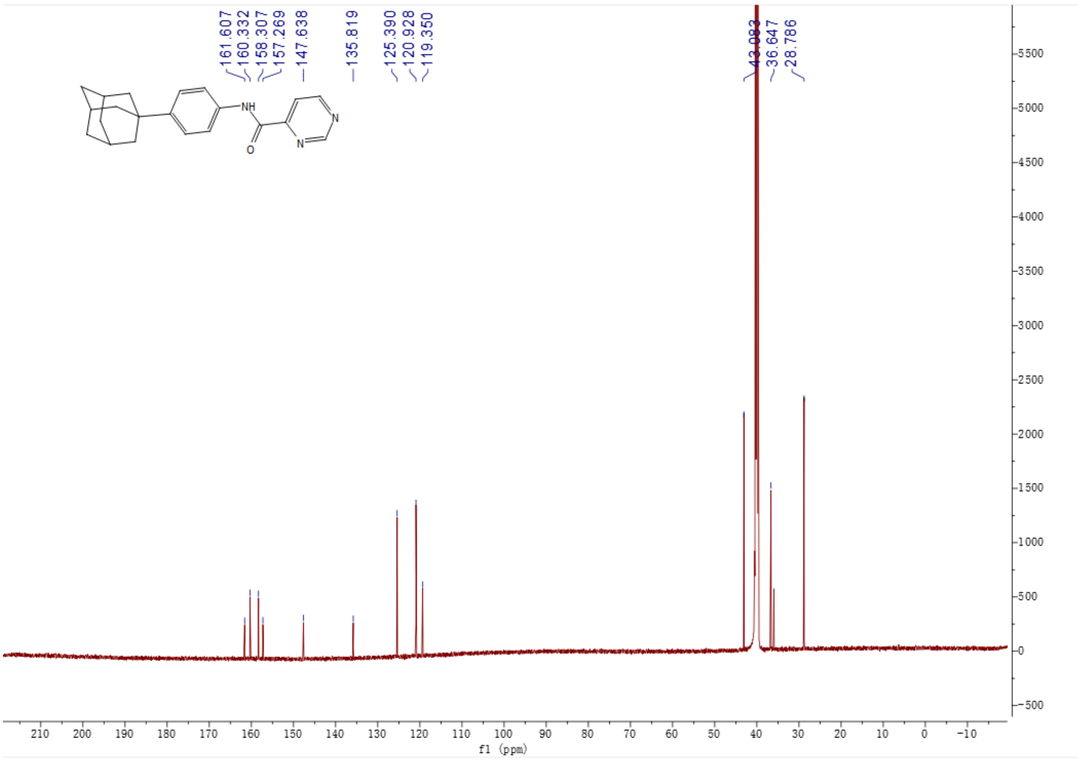

^{13}C NMR

*N-(4-((3r,5r,7r)-adamantan-1-yl)phenyl)-5-methoxypicolinamide(**1-4e**)*

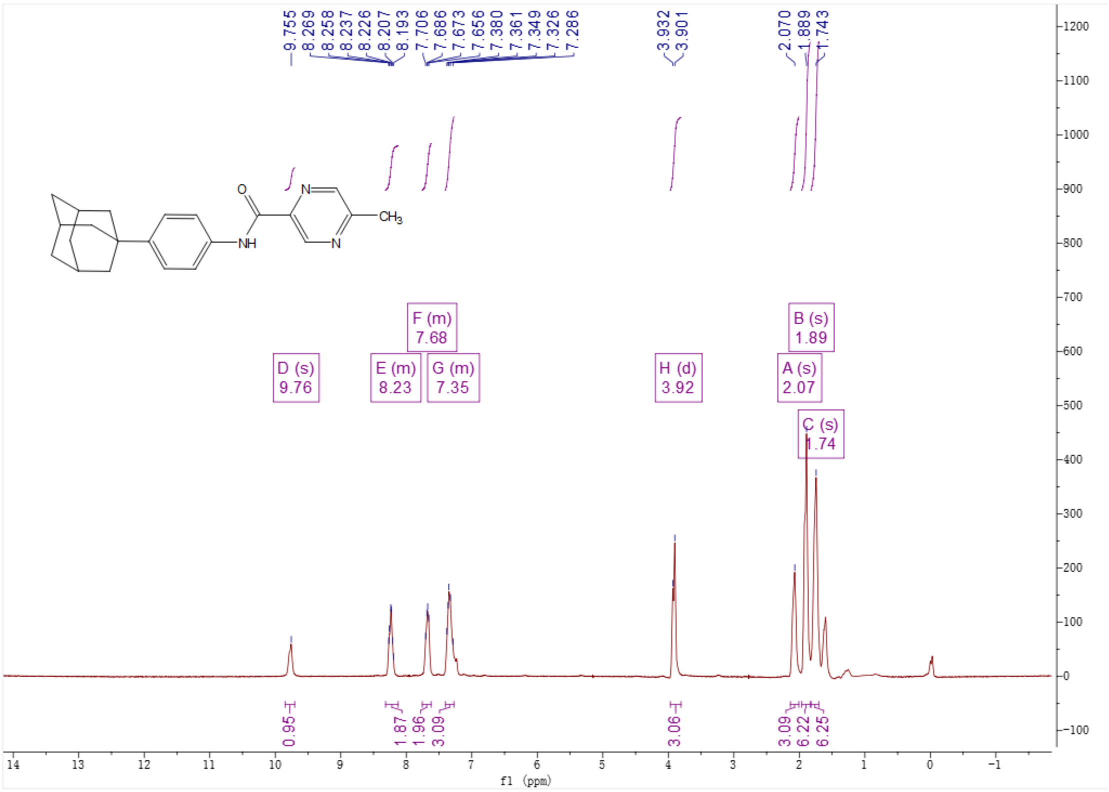

^{1}H NMR

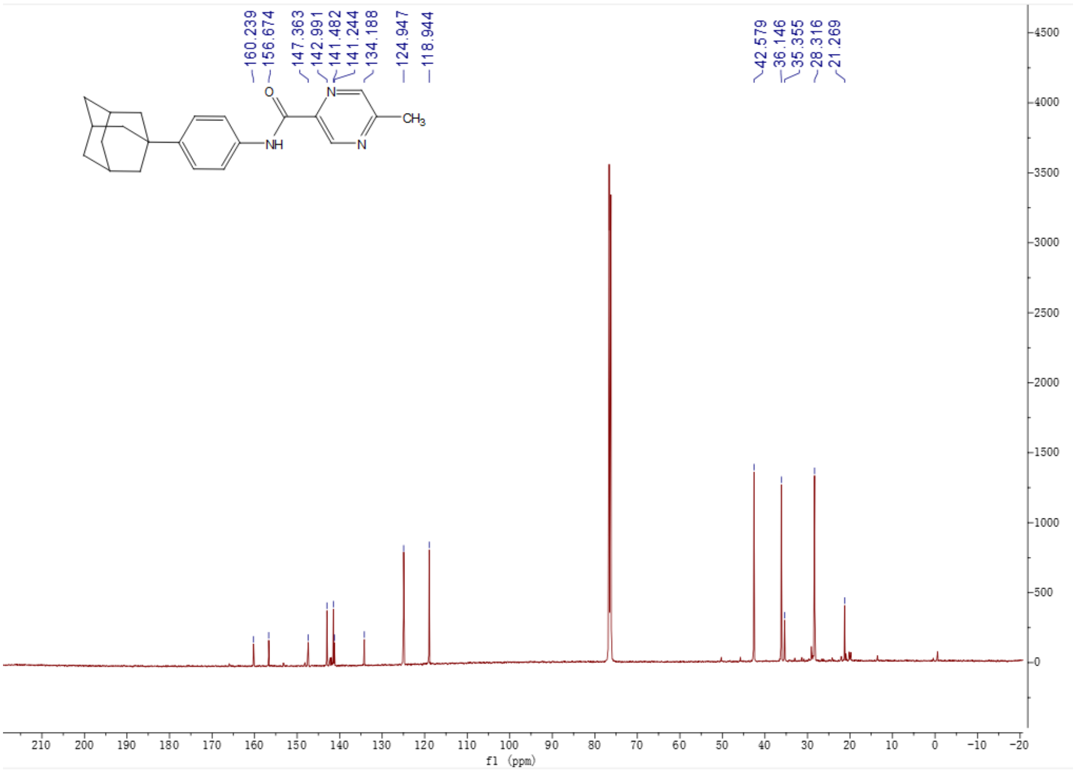

^{13}C NMR

*N-(4-((3r,5r,7r)-adamantan-1-yl)phenyl)-5-methylpyrazine-2-carboxamide(**1-4f**)*

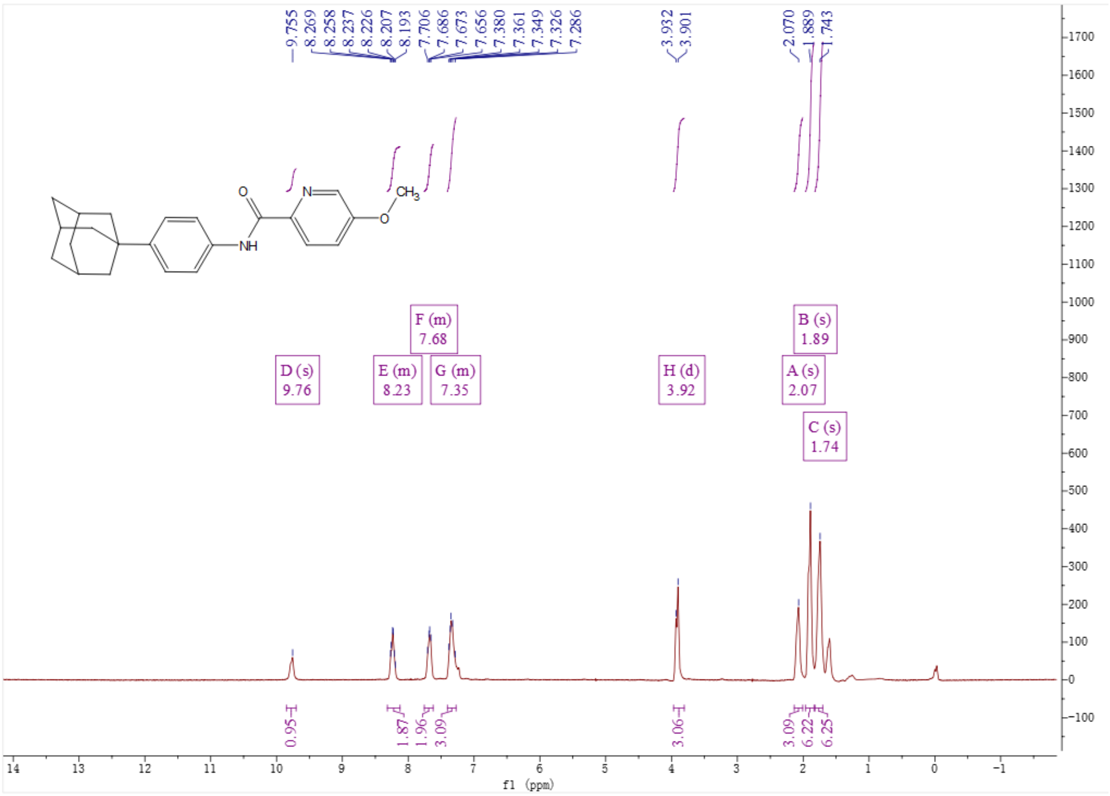

^{1}H NMR

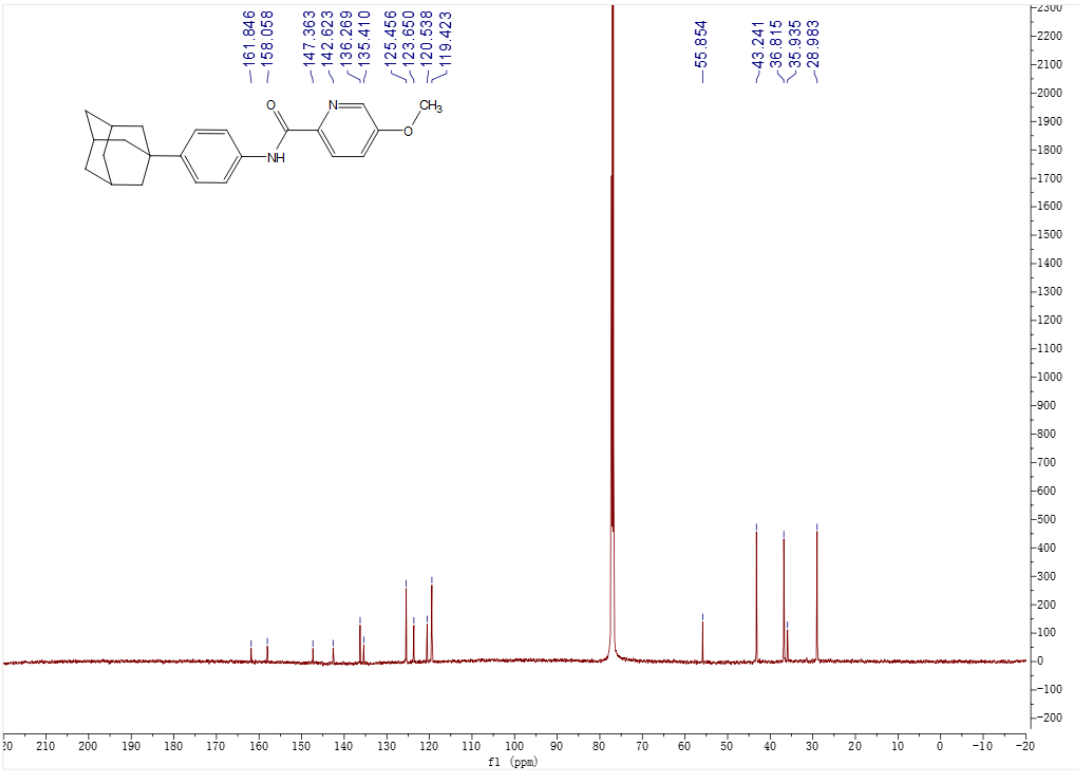

^{13}C NMR

*N-(4-((3r,5r,7r)-adamantan-1-yl)phenyl)-4-methoxypicolinamide(**1-4g**)*

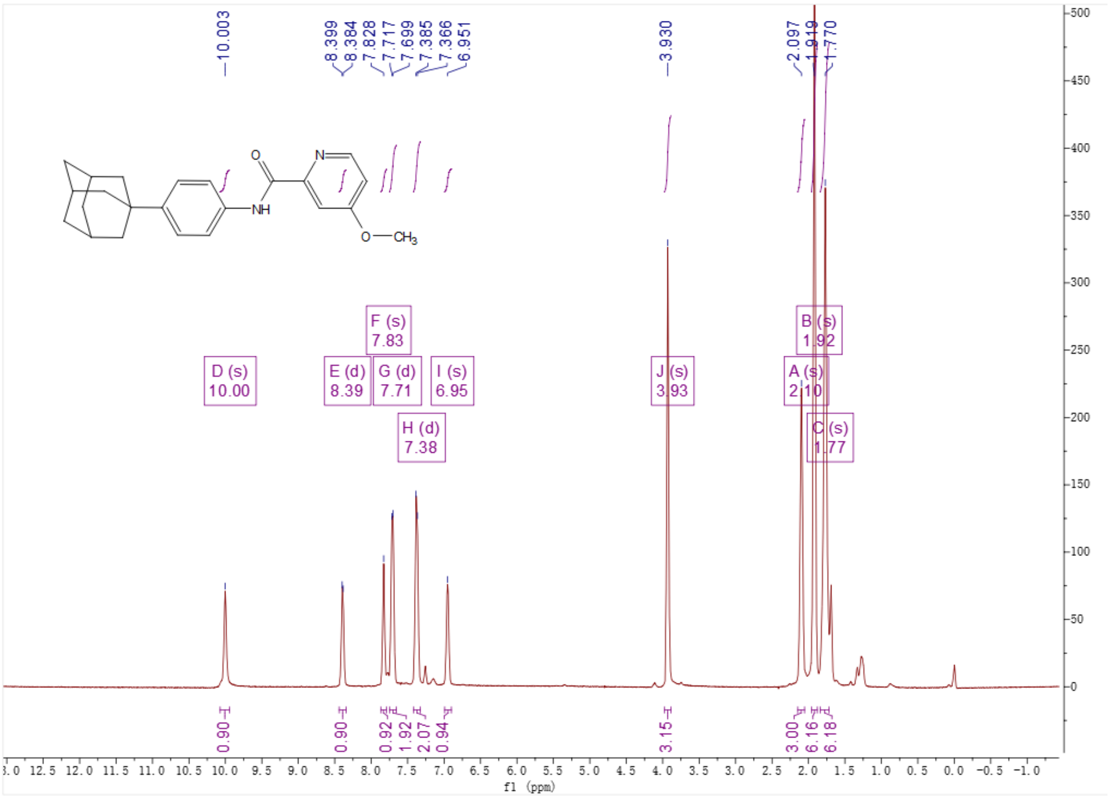

^{1}H NMR

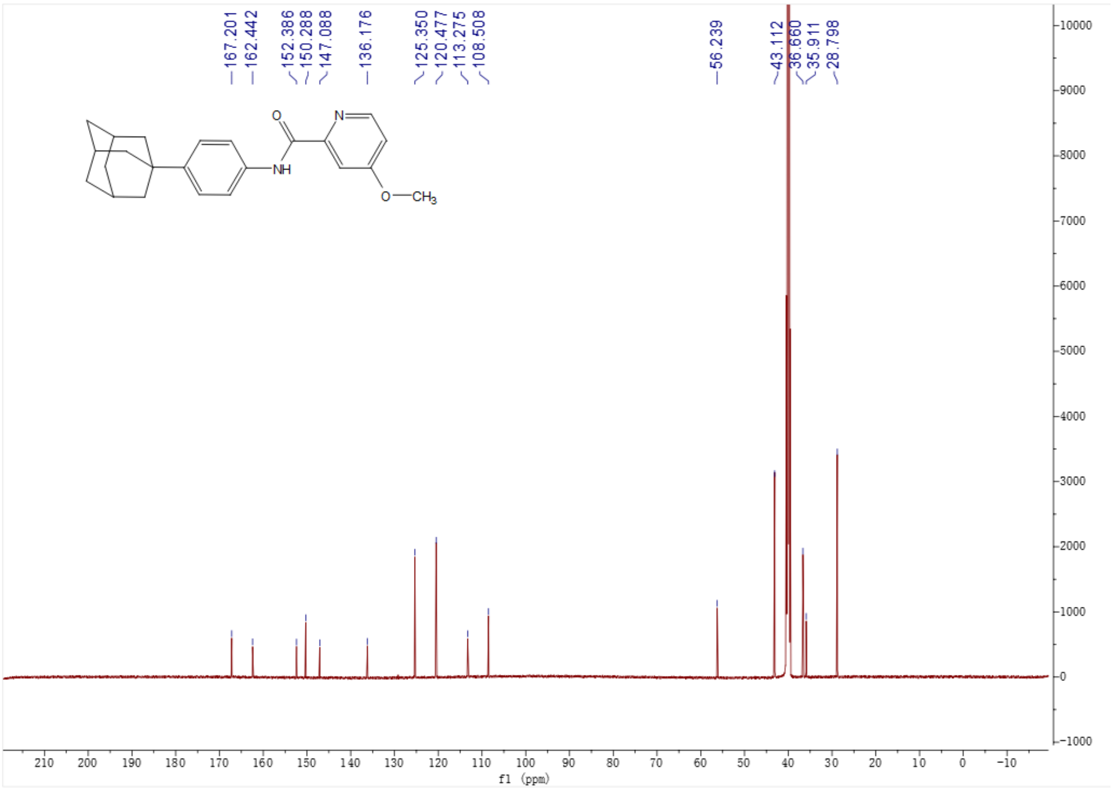

^{13}C NMR

*N-(4-((3r,5r,7r)-adamantan-1-yl)phenyl)-4-chloropicolinamide(**1-4h**)*

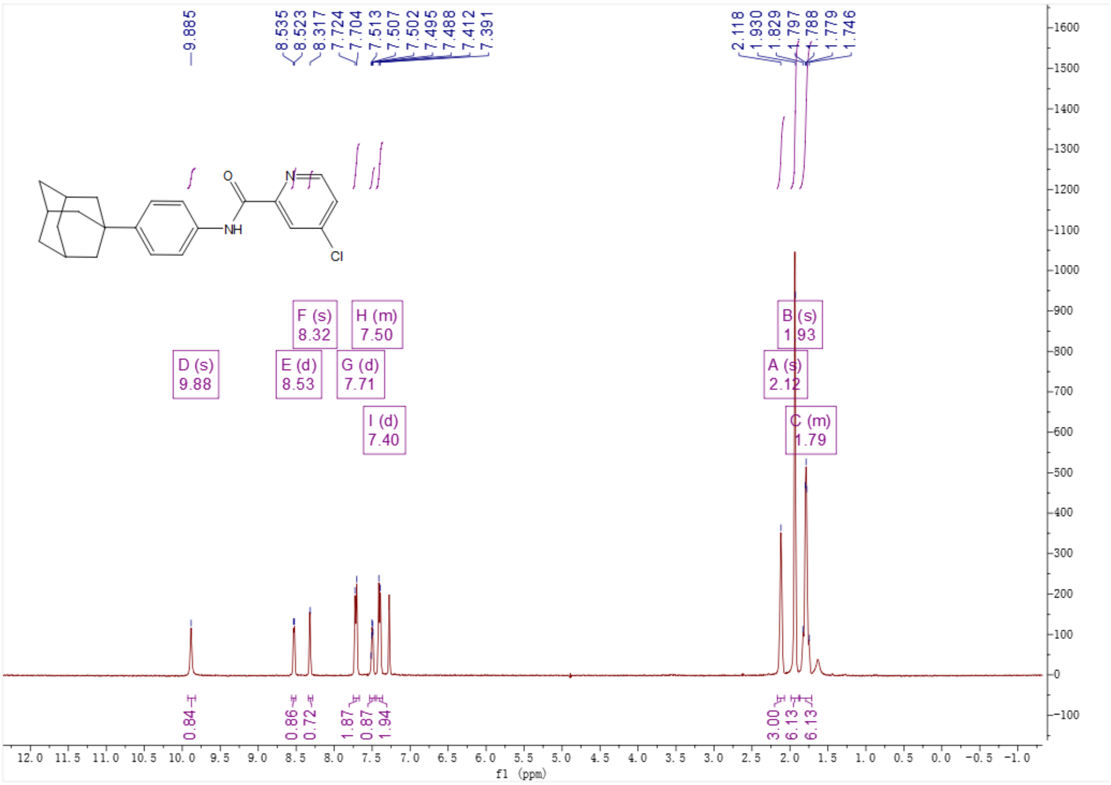

^{1}H NMR

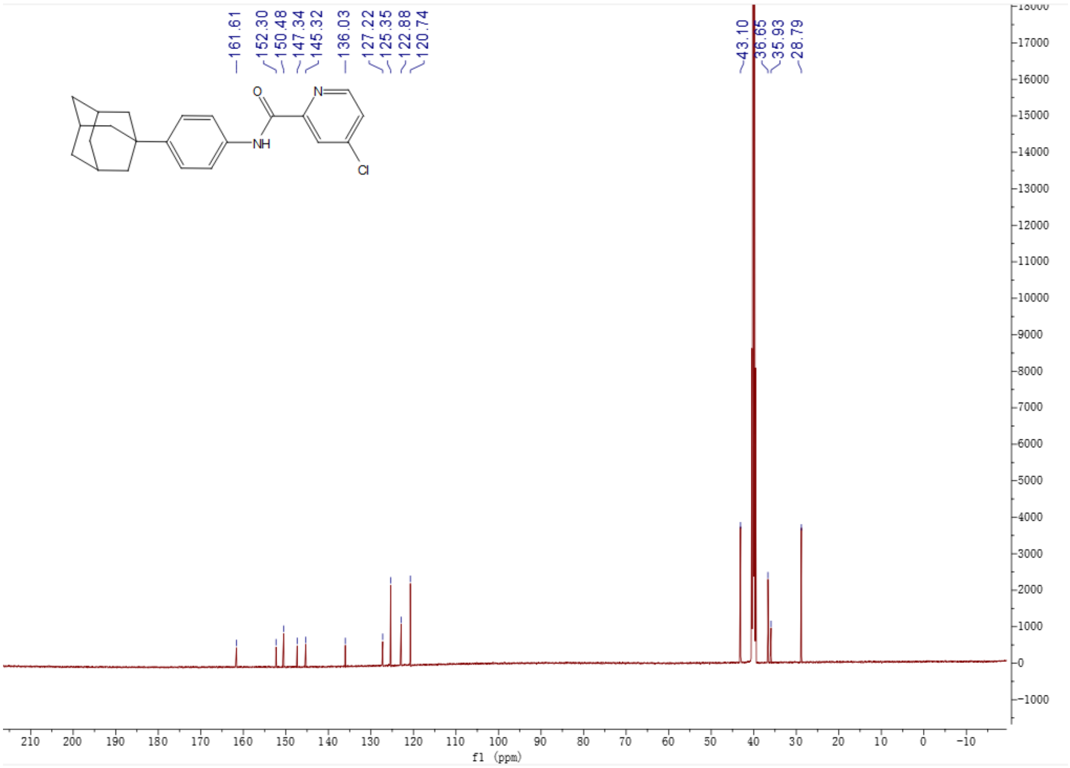

^{13}C NMR

*N-(4-((3r,5r,7r)-adamantan-1-yl)phenyl)thiazole-5-carboxamide(**1-4i**)*

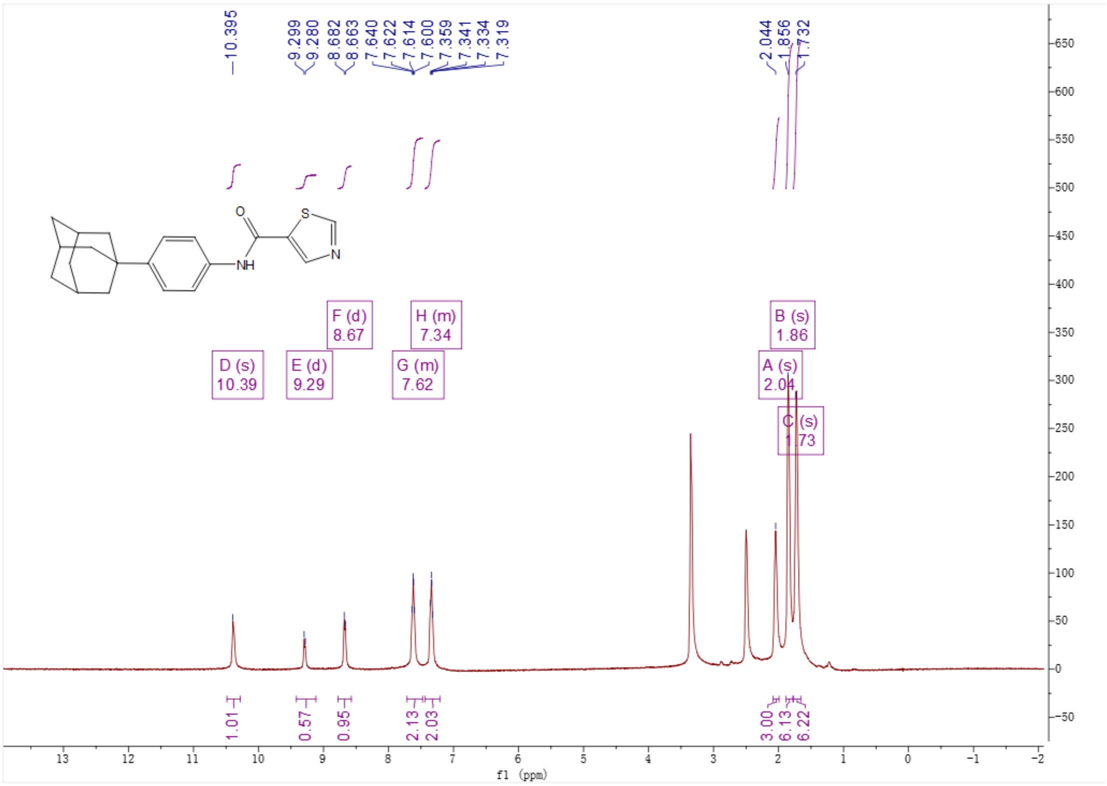

^{1}H NMR

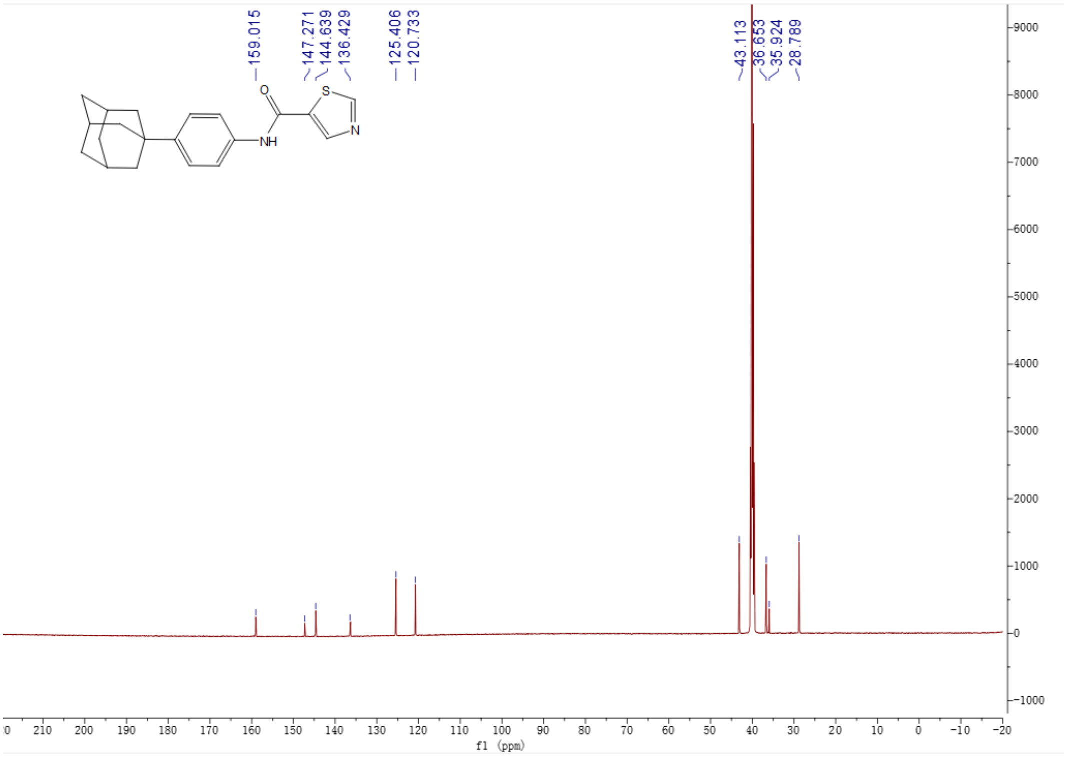

^{13}C NMR

*N-(4-((3r,5r,7r)-adamantan-1-yl)phenyl)thiophene-2-carboxamide(**1-4j**)*

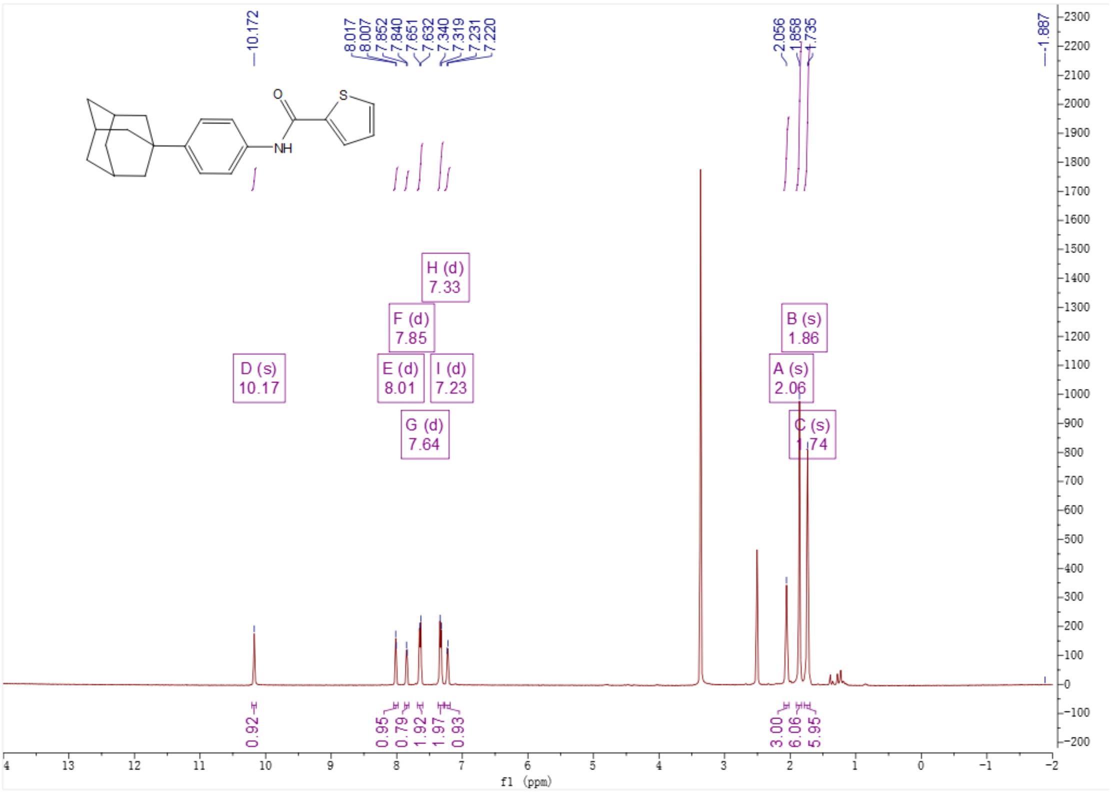

^{1}H NMR

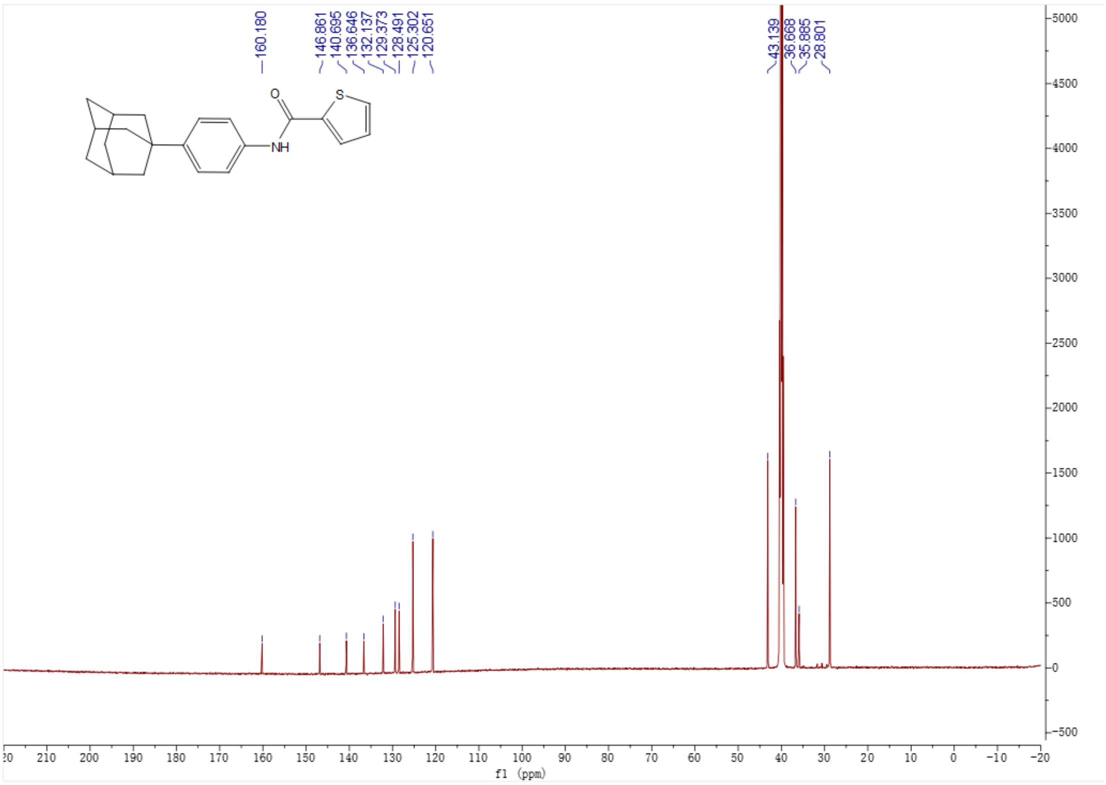

^{13}C NMR

*N-(4-((3r,5r,7r)-adamantan-1-yl)phenyl)-1H-imidazole-4-carboxamide(**1-4k**)*

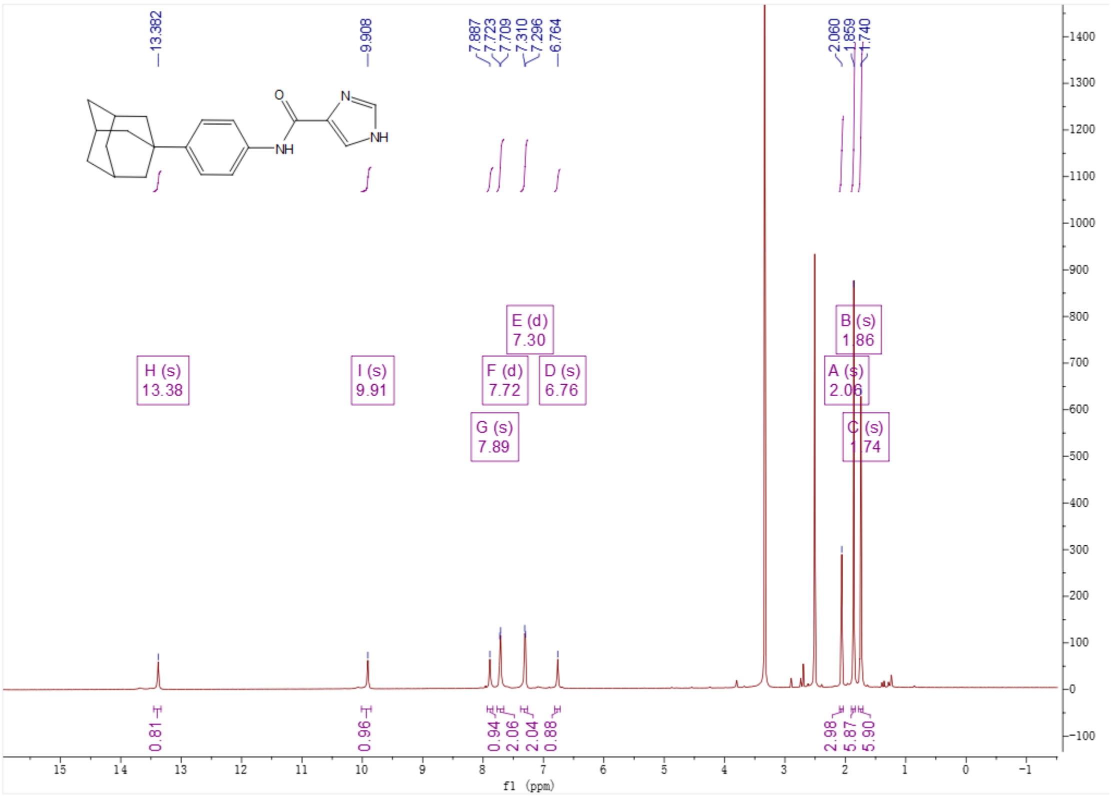

^{1}H NMR

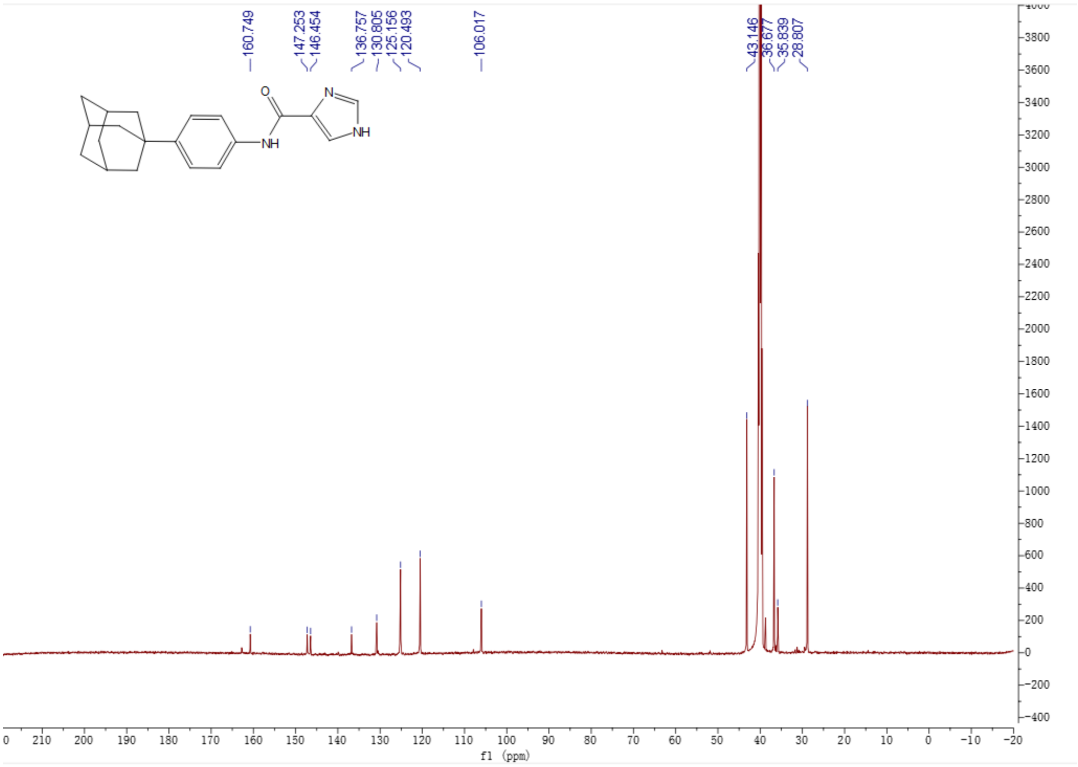

^{13}C NMR

*N-(4-((3r,5r,7r)-adamantan-1-yl)phenyl)-1-methyl-1H-imidazole-5-carboxamide(**1-4l**)*

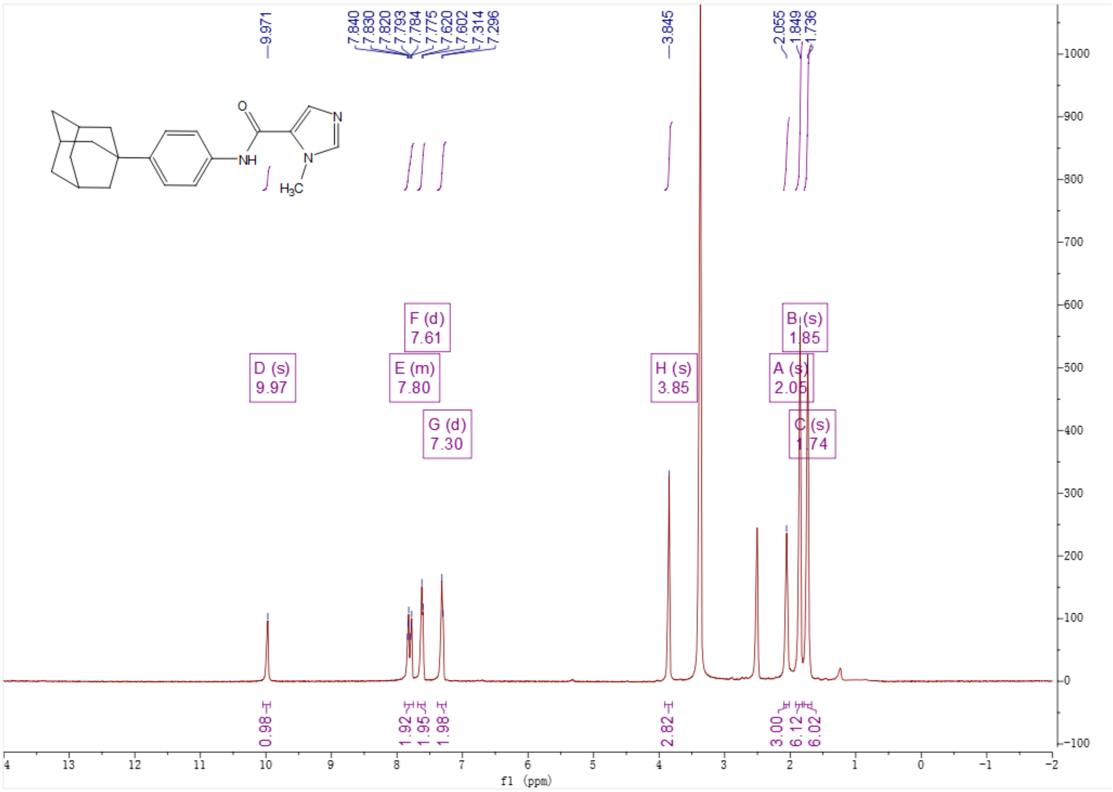

^{1}H NMR

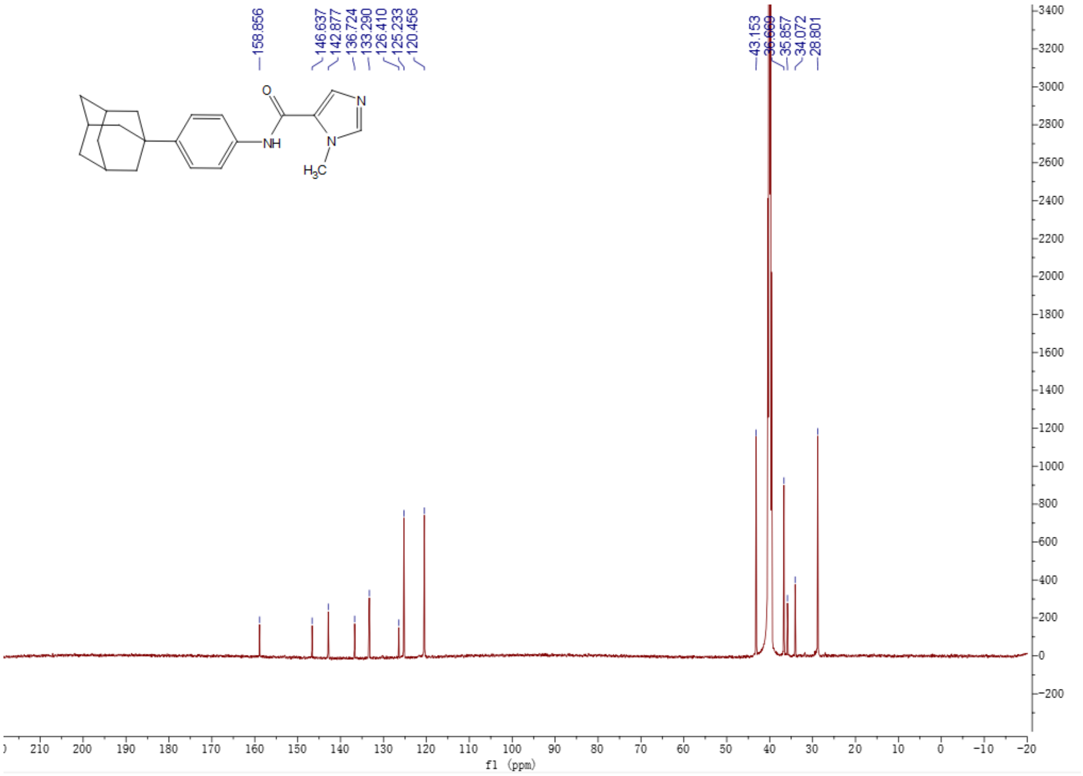

^{13}C NMR

*N-(4-((3r,5r,7r)-adamantan-1-yl)phenyl)-1H-pyrazole-3-carboxamide(**1-4m**)*

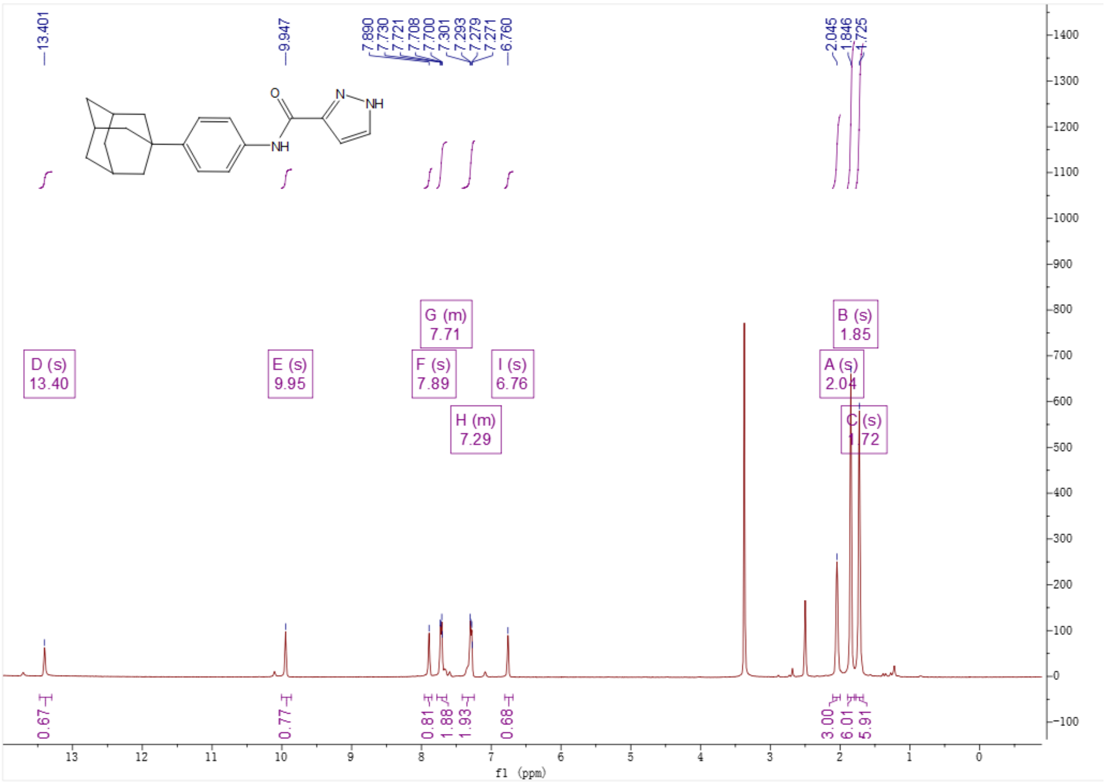

^{1}H NMR

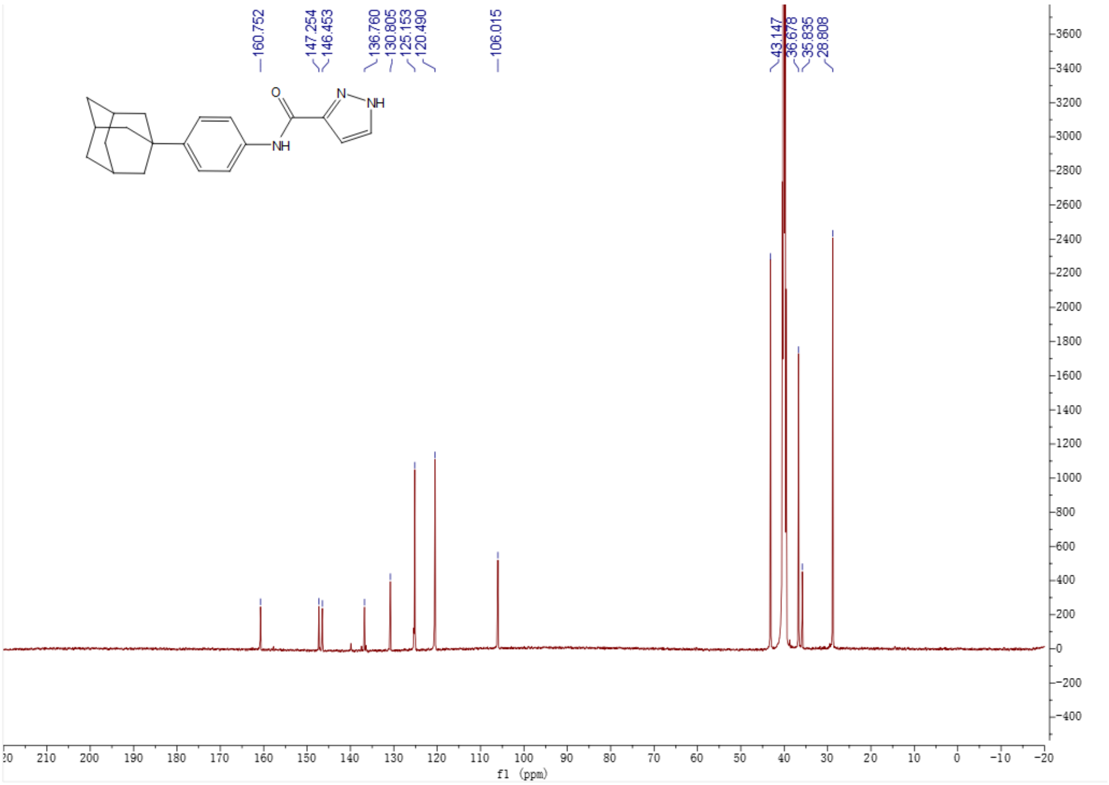

^{13}C NMR

*N-(4-((3r,5r,7r)-adamantan-1-yl)phenyl)-1-methyl-1H-pyrrole-2-carboxamide(**1-4n**)*

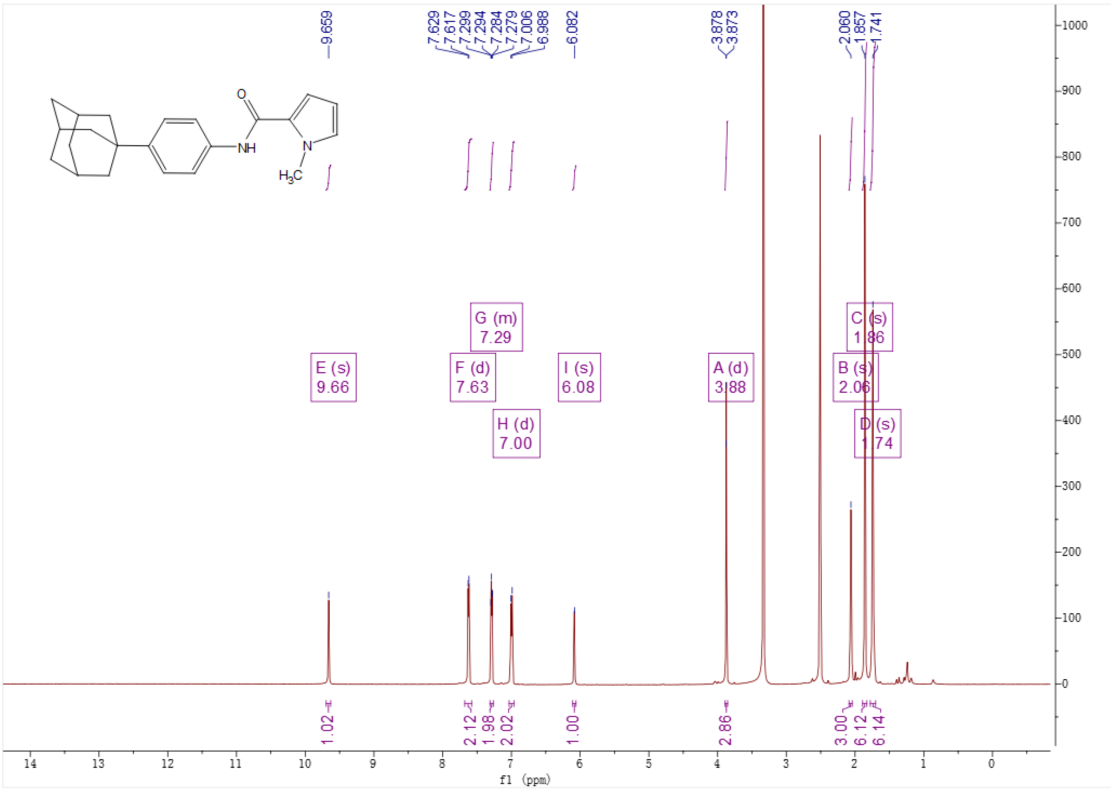

^{1}H NMR

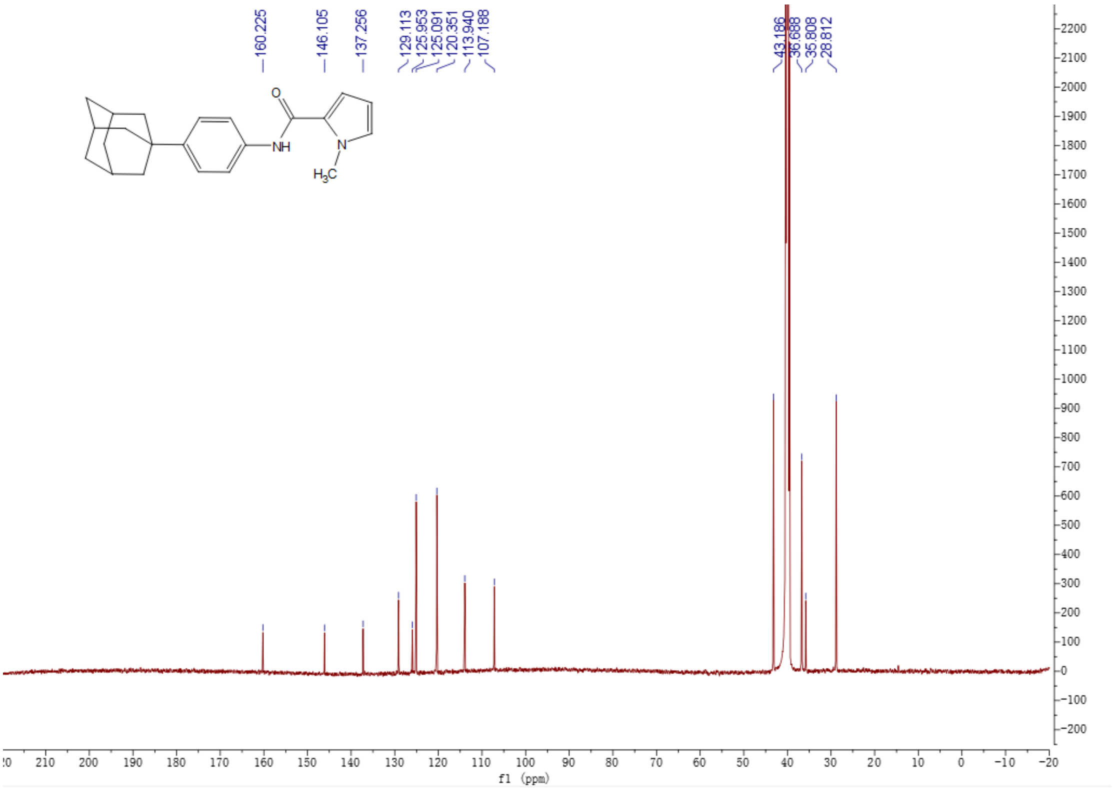

^{13}C NMR

*N-(4-((3r,5r,7r)-adamantan-1-yl)phenyl)thiophene-3-carboxamide(**1-4o**)*

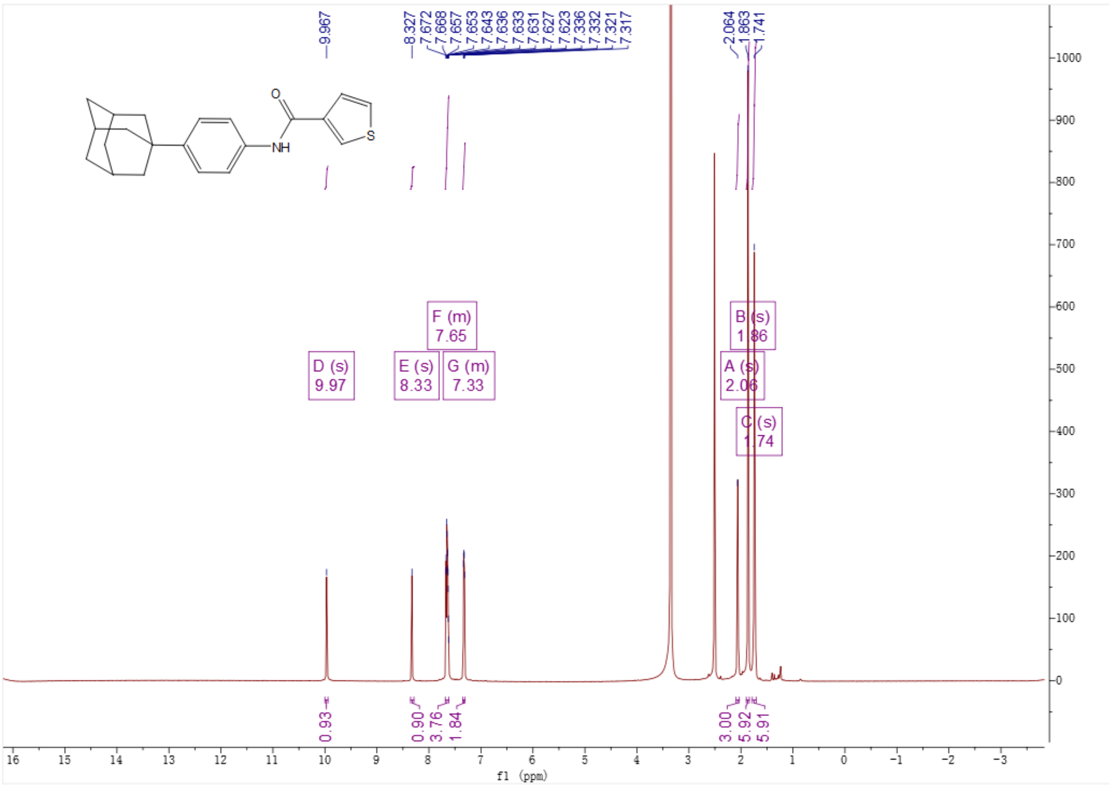

^{1}H NMR

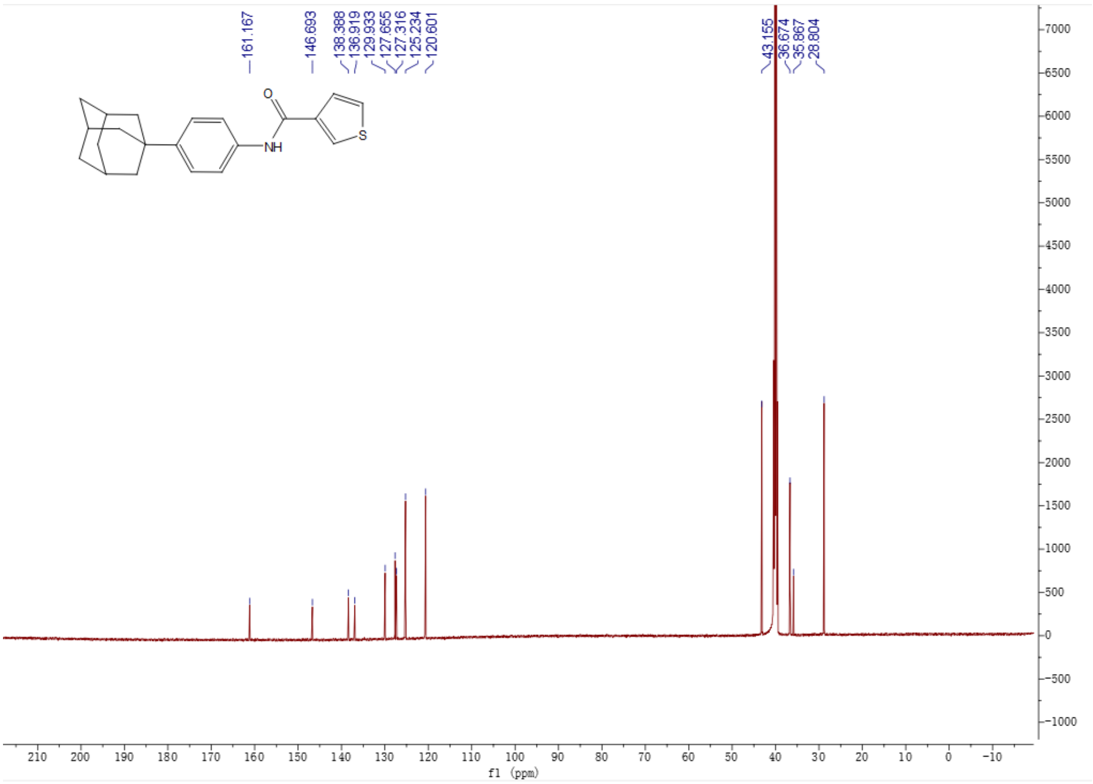

^{13}C NMR

*N-(4-((3r,5r,7r)-adamantan-1-yl)phenyl)-4-aminopicolinamide(**1-4p**)*

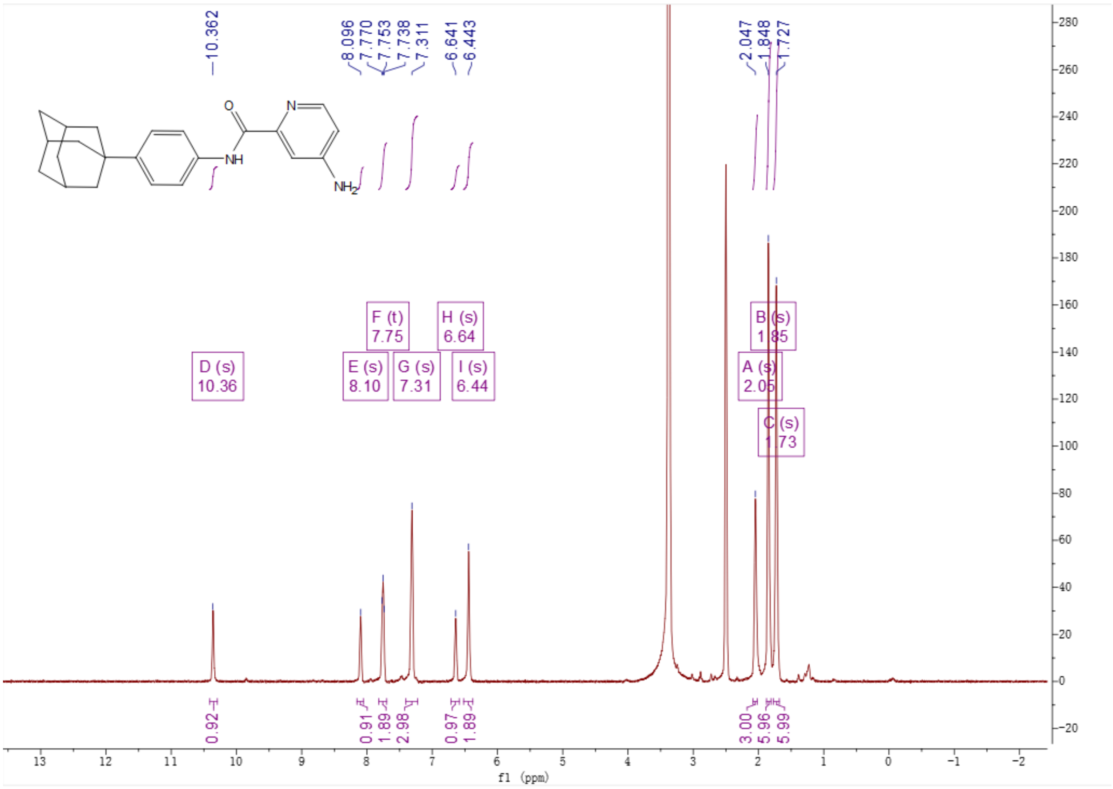

^{1}H NMR

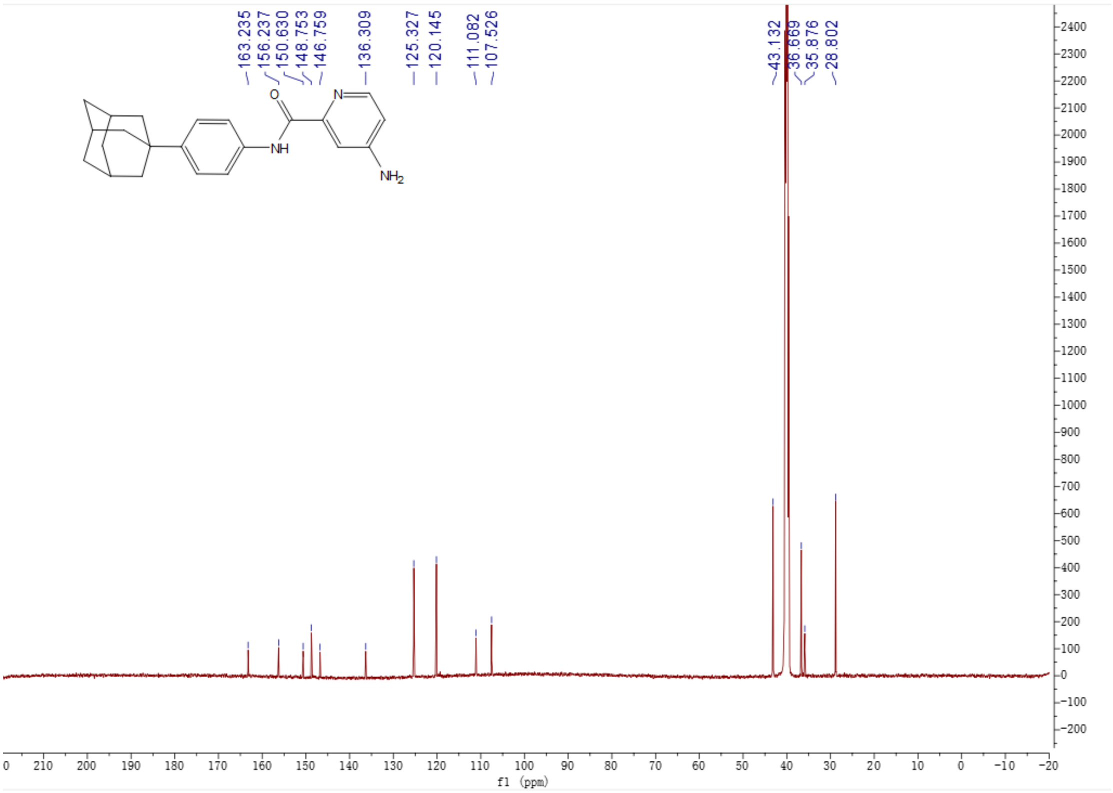

^{13}C NMR

*N-(4-((3r,5r,7r)-adamantan-1-yl)phenyl)-6-aminopicolinamide(**1-4q**)*

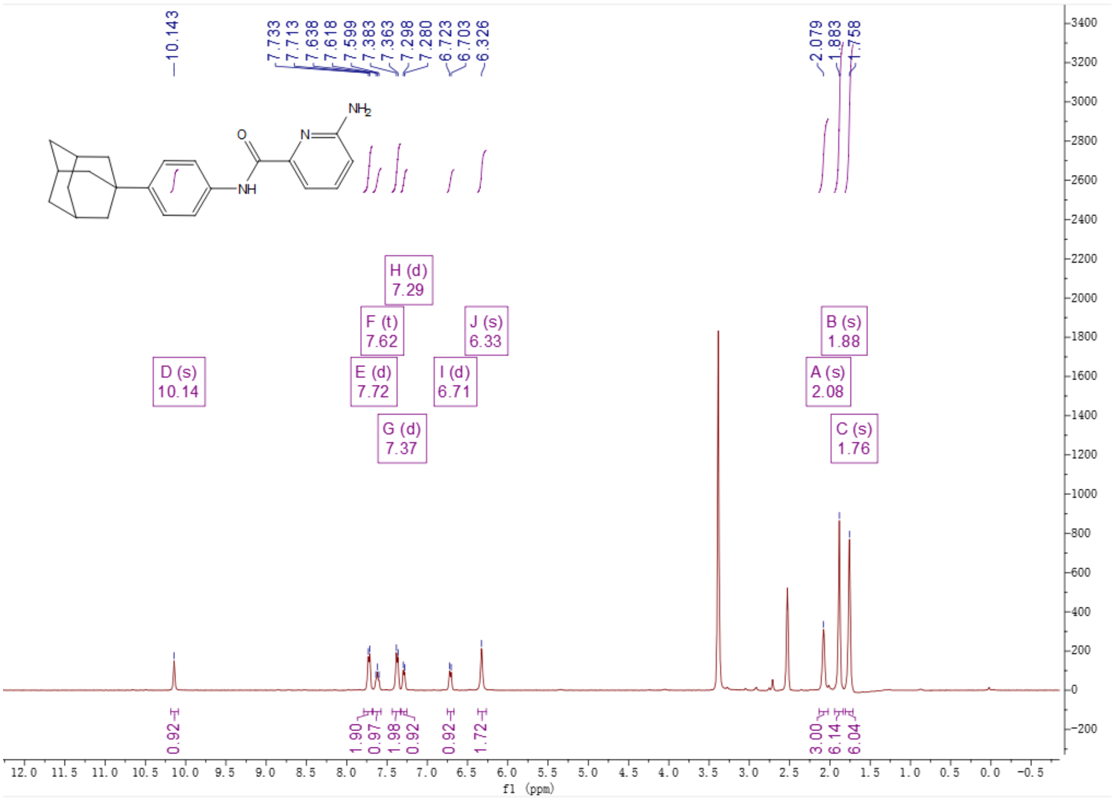

^{1}H NMR

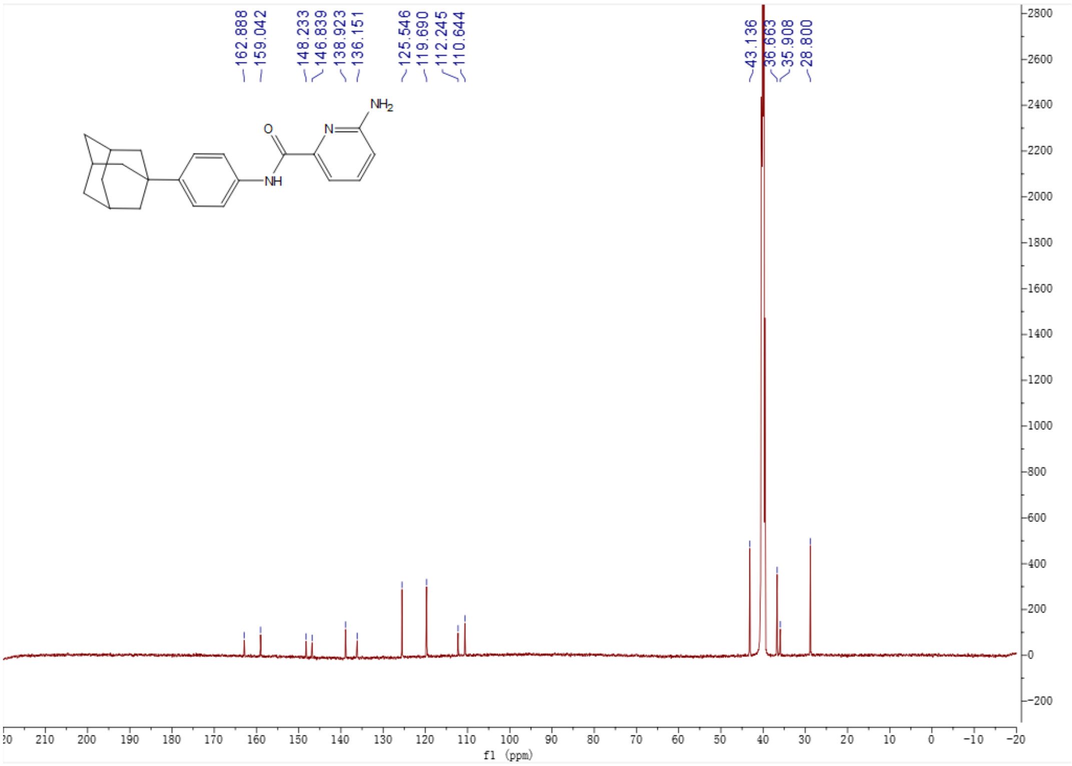

^{13}C NMR

*N-(4-((3r,5r,7r)-adamantan-1-yl)phenyl)-3-aminopicolinamide(**1-4r**)*

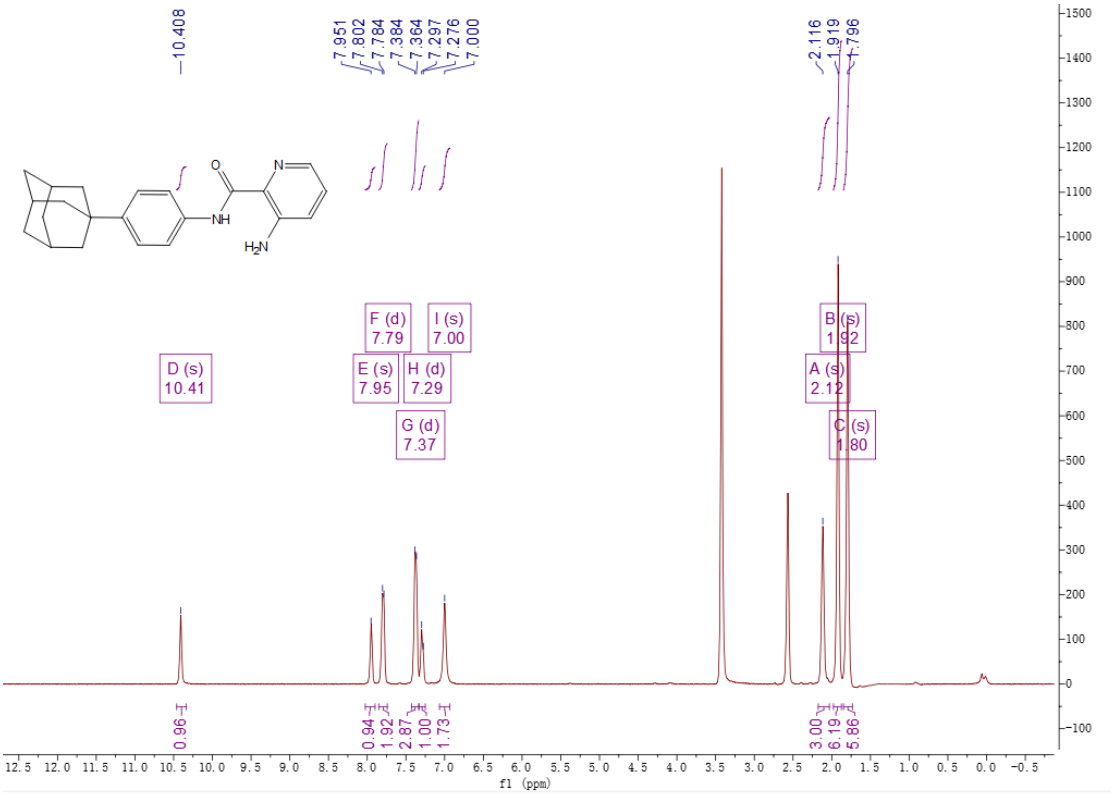

^{1}H NMR

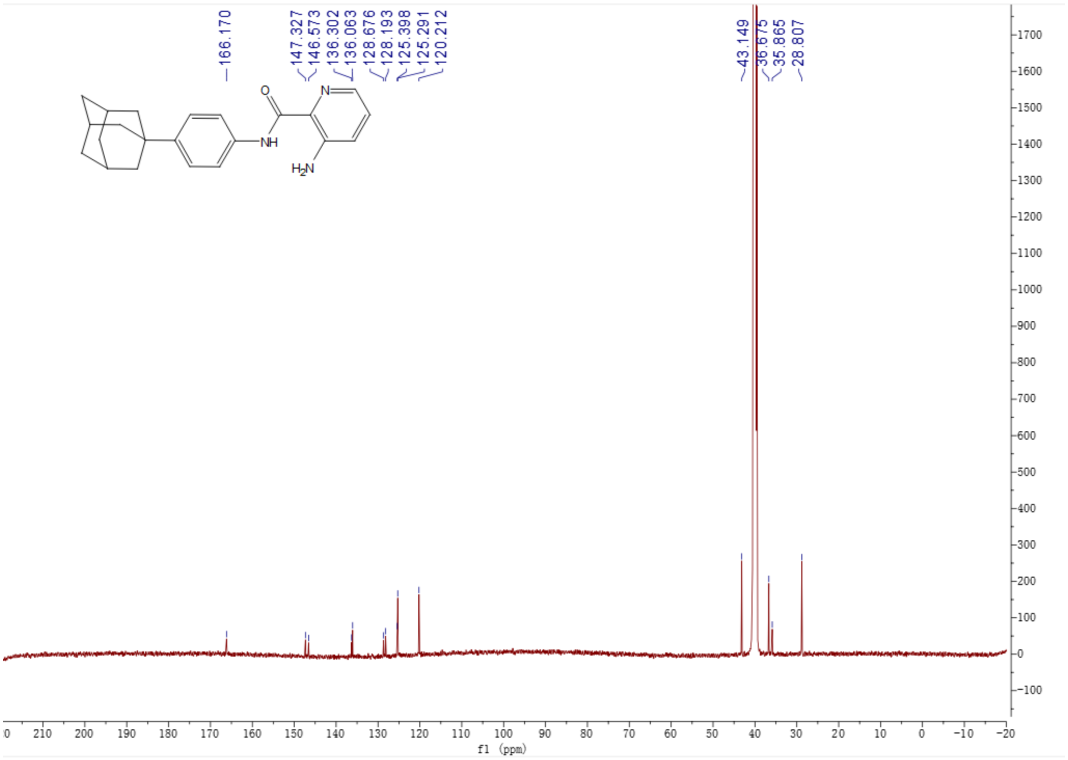

^{13}C NMR

*N-(4-(((1R,3S,5r,7r)-adamantan-2-yl)carbamoyl)phenyl)picolinamide(**2-3a**)*

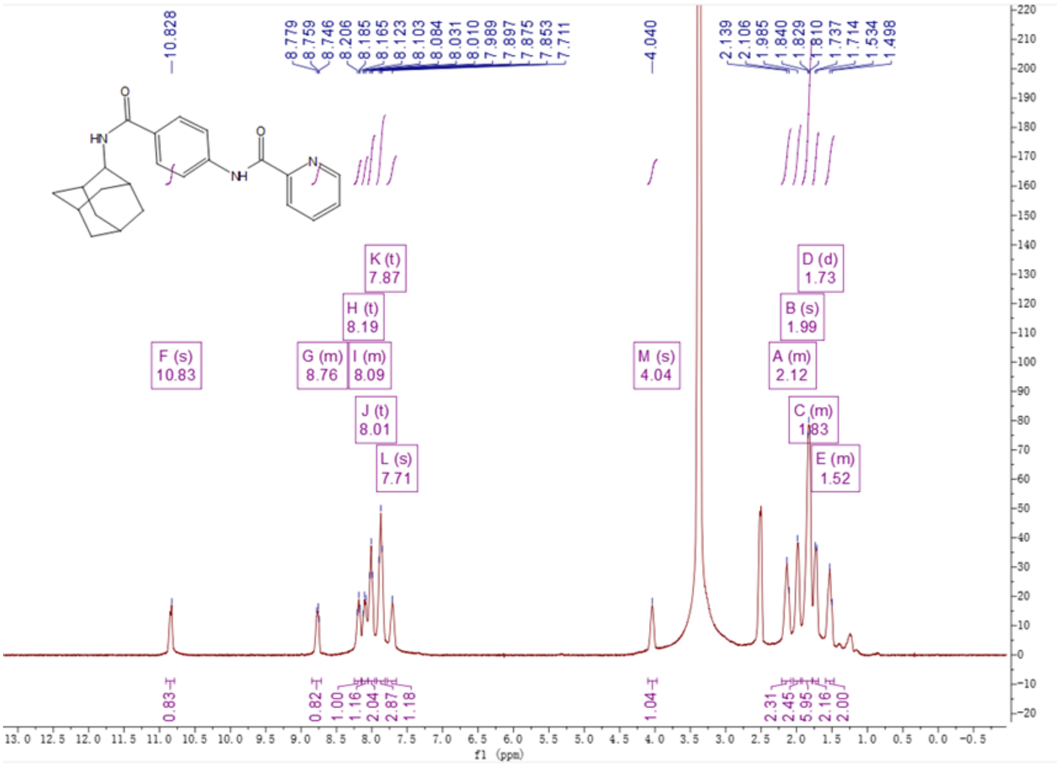

^{1}H NMR

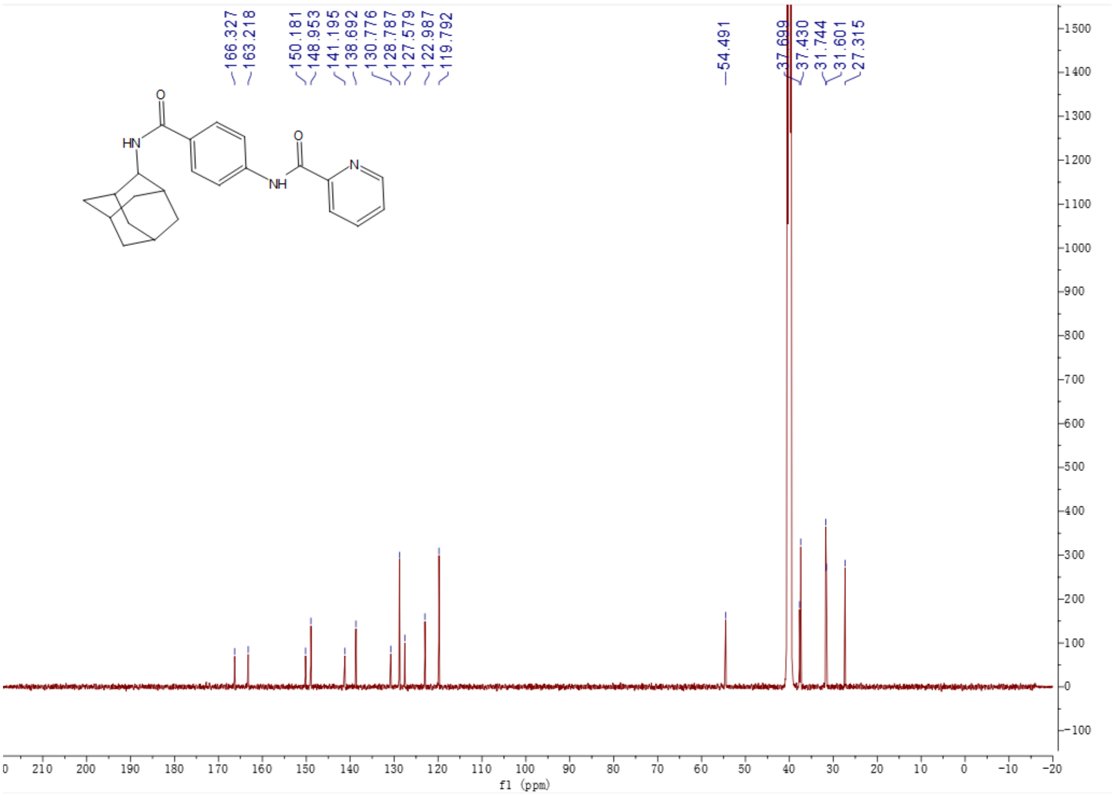

^{13}C NMR

*N-(4-(2-((3r,5r,7r)-adamantan-1-yl)acetamido)phenyl)picolinamide(**2-3b**)*

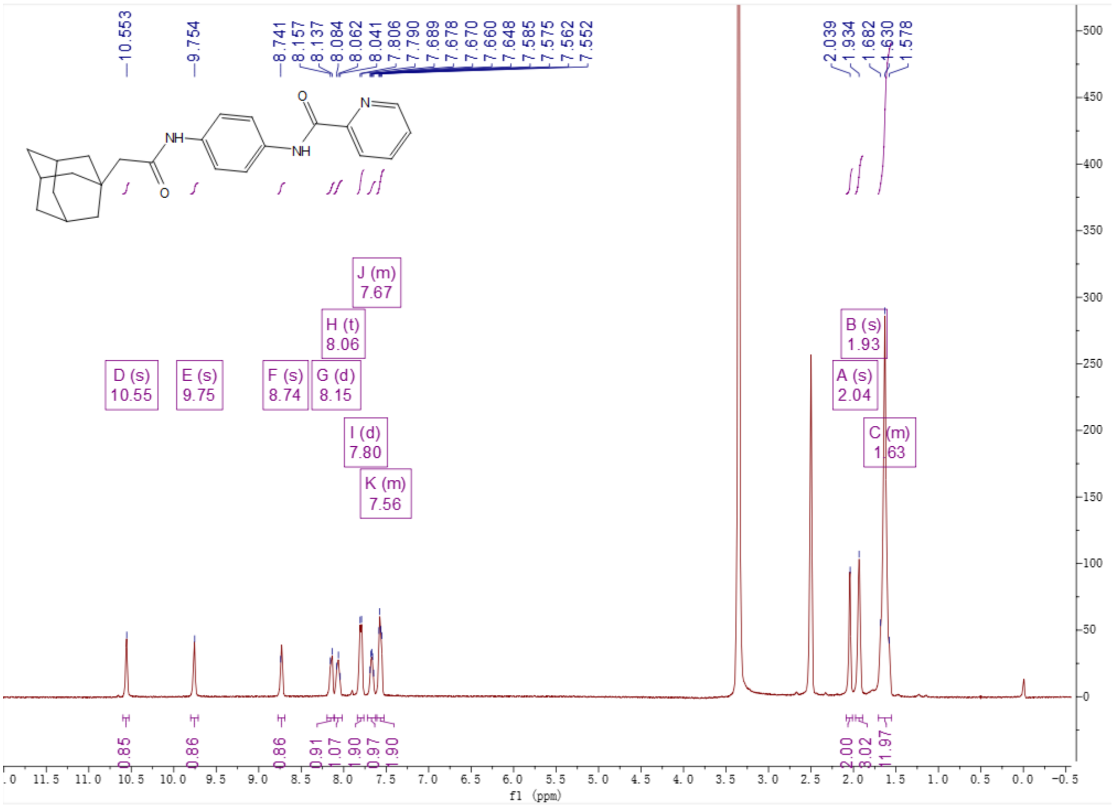

^{1}H NMR

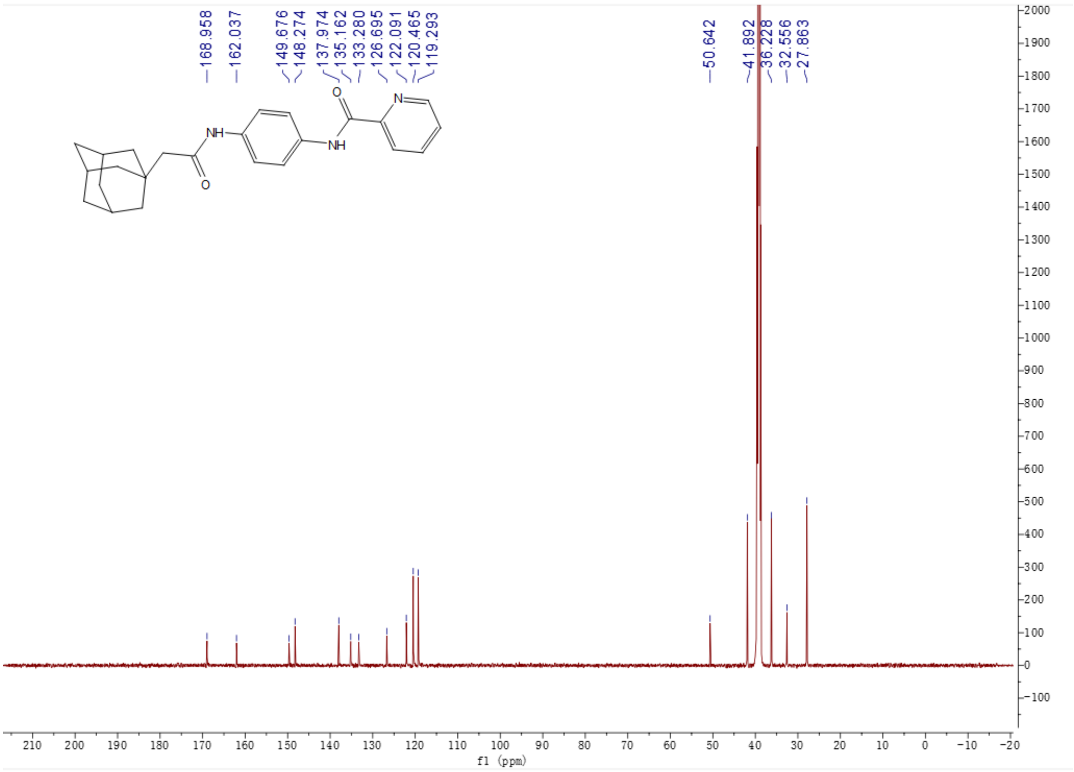

^{13}C NMR

*N-(4-((1-((3r,5r,7r)-adamantan-1-yl)ethyl)carbamoyl)phenyl)picolinamide(**2-3c**)*

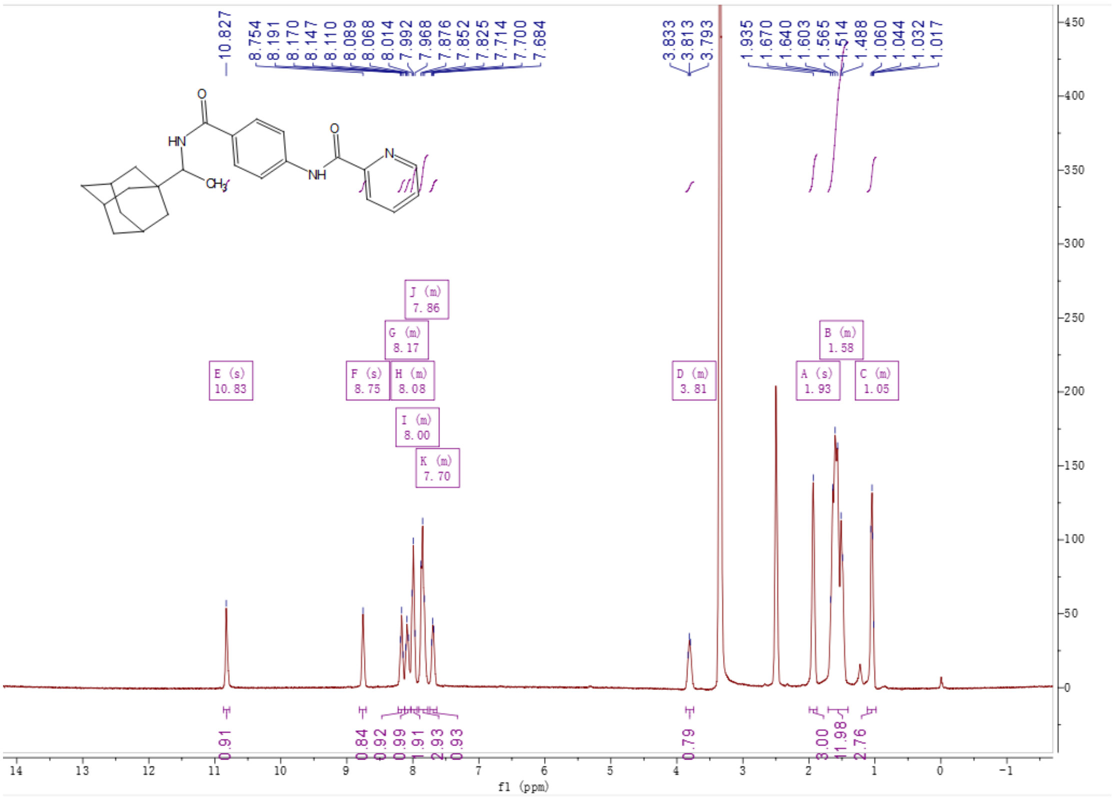

^{1}H NMR

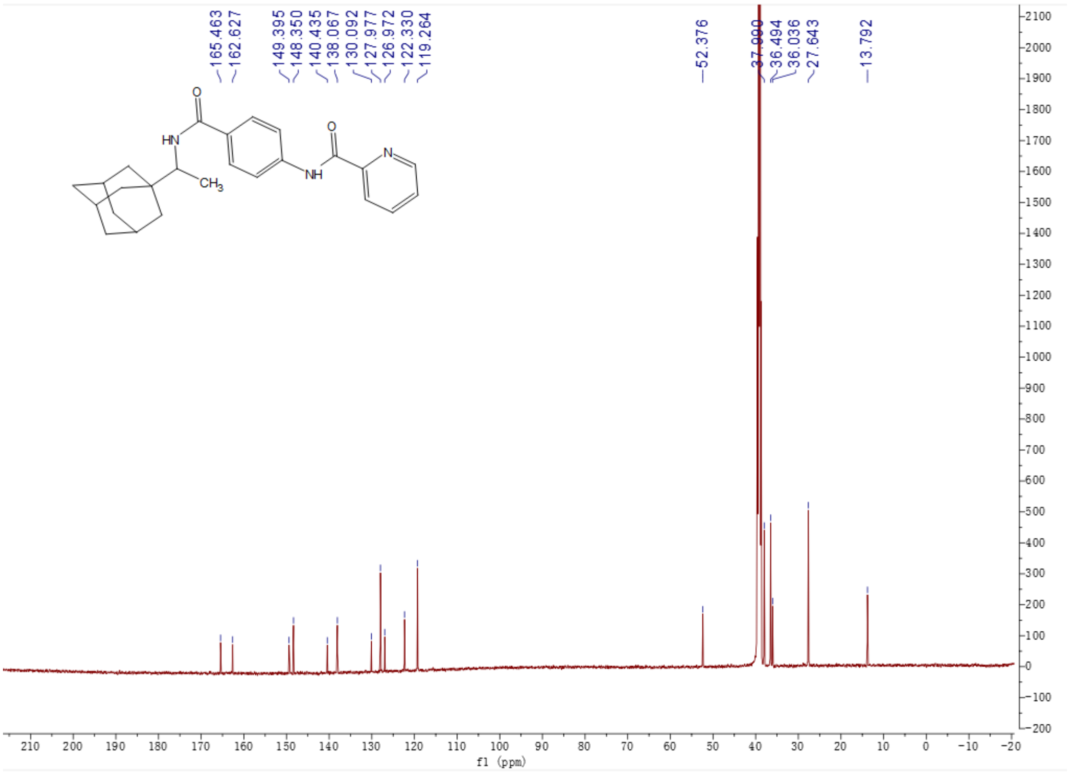

^{13}C NMR

N-(4-((1r,3s,5R,7S)-3-hydroxyadamantane-1-carboxamido)phenyl)picolinamide (**2-3d**)

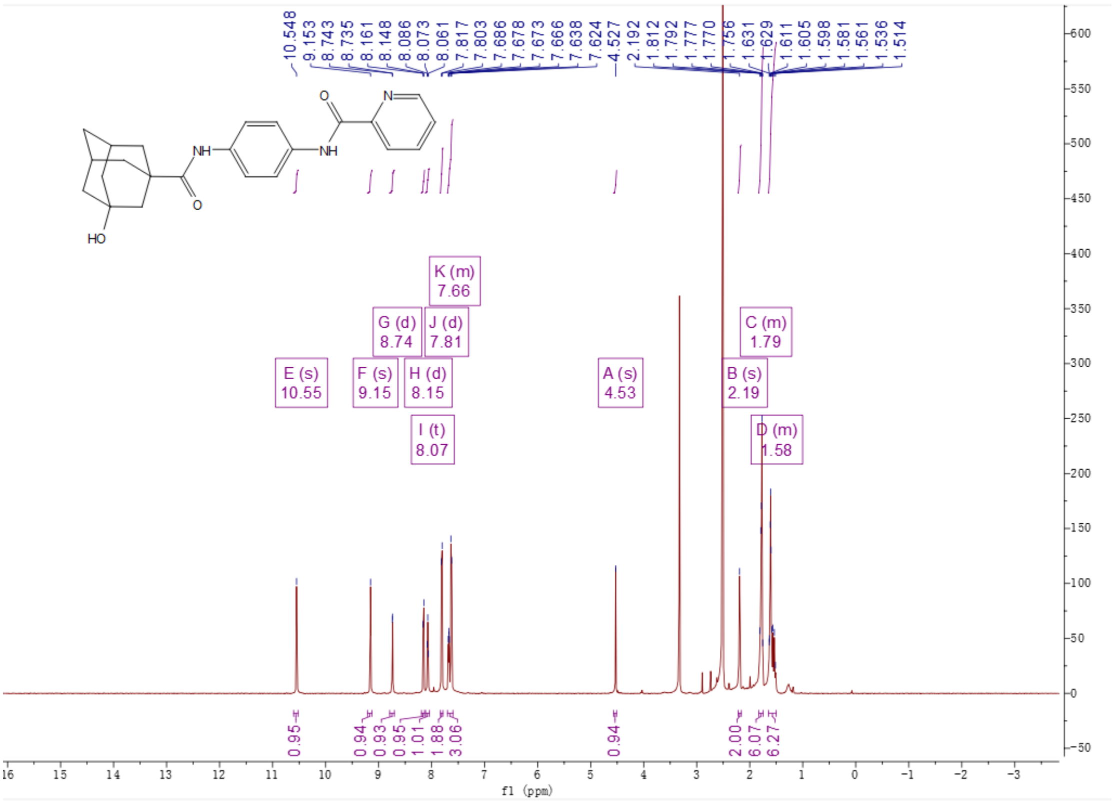

^{1}H NMR

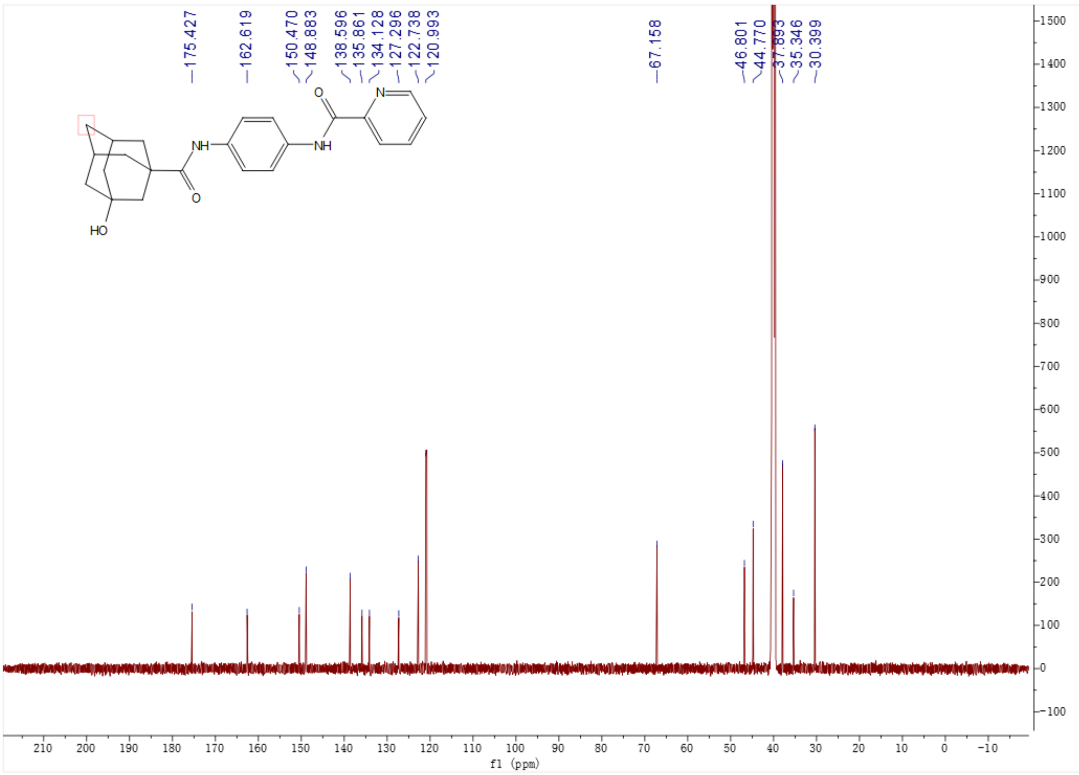

^{13}C NMR

N-(4-(((1r,3R,5S,7r)-3,5-dimethyladamantan-1-yl)carbamoyl)phenyl)picolinamide (**2-3e**)

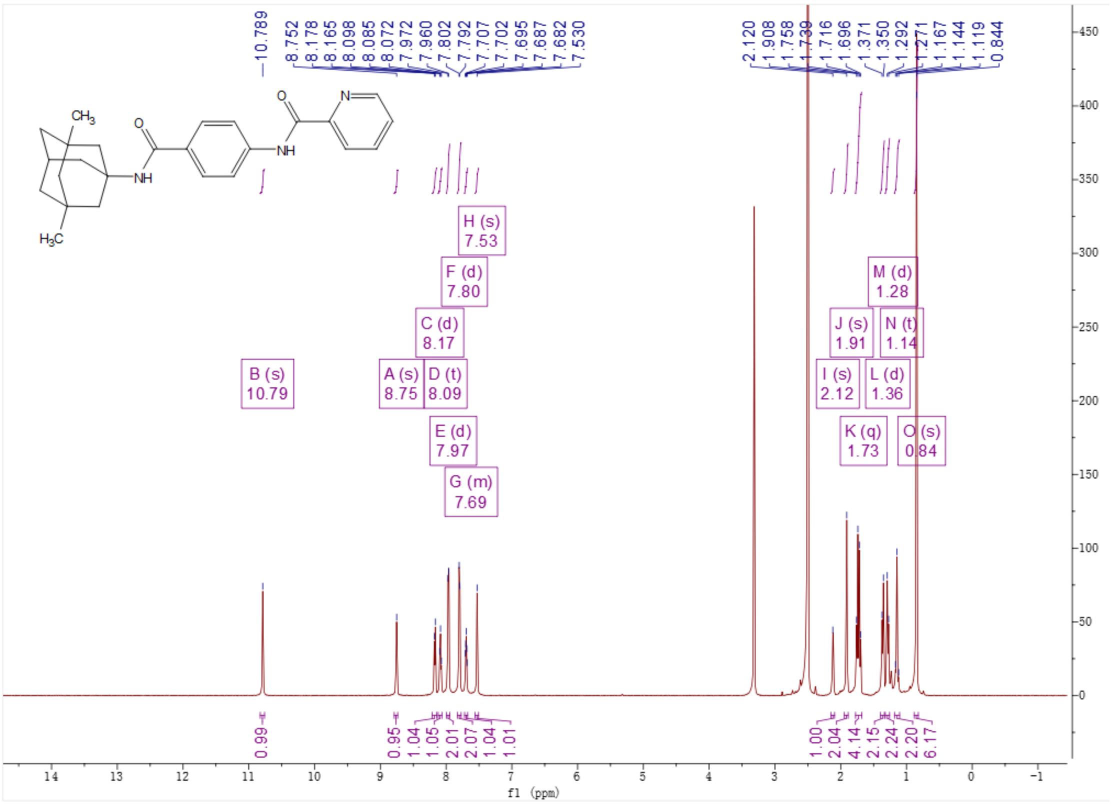

^{1}H NMR

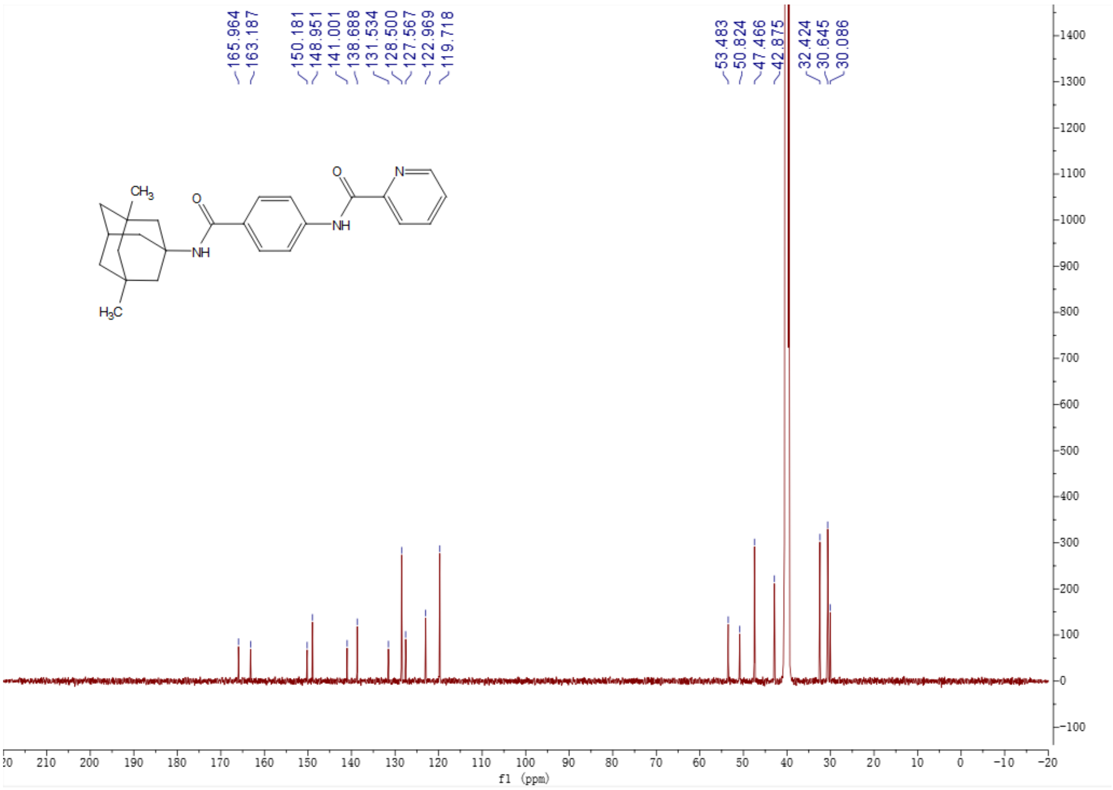

^{13}C NMR

*N-(4-((3r,5r,7r)-adamantan-1-yl)-2,6-dimethylphenyl)-3-chlorobenzamide (**3-2a**)*

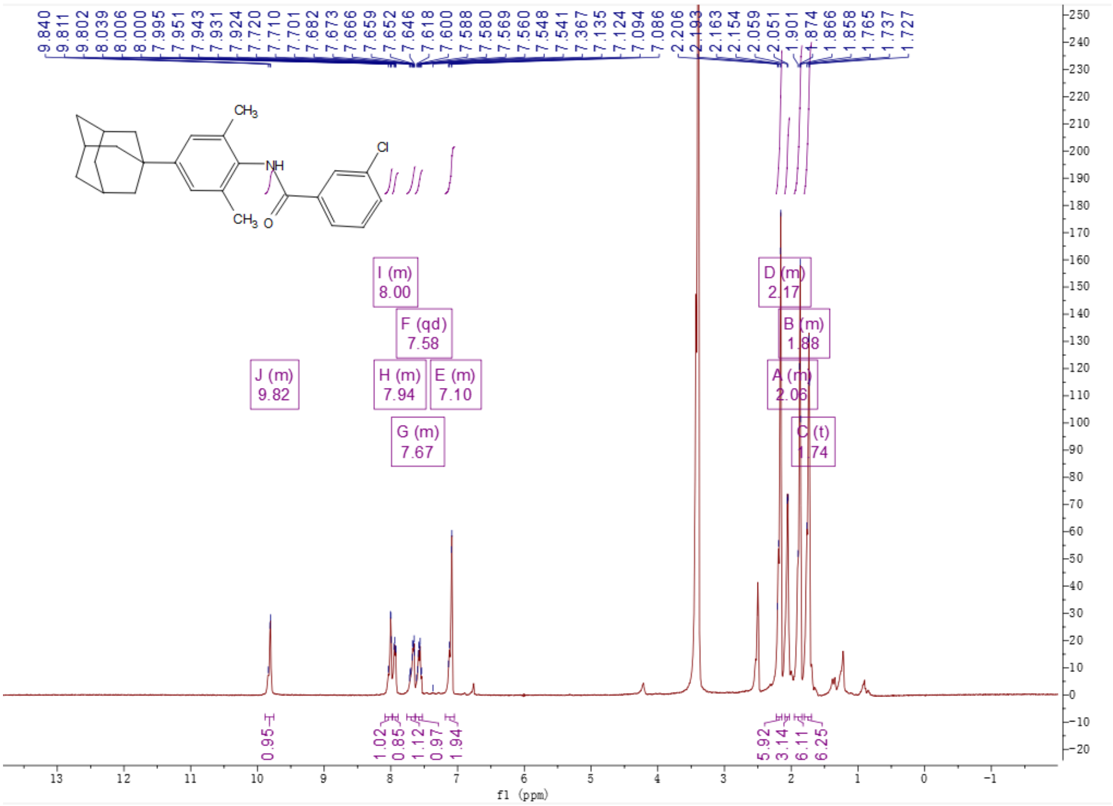

^{1}H NMR

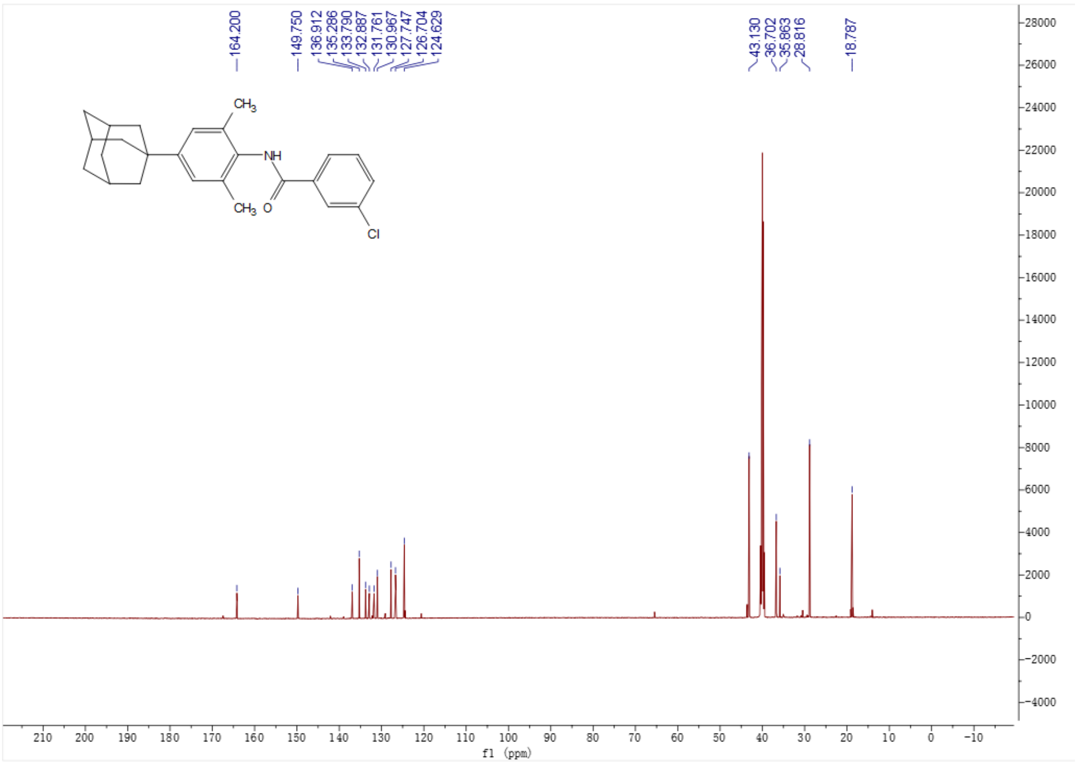

^{13}C NMR

*N-(4-((3r,5r,7r)-adamantan-1-yl)-2,6-diisopropylphenyl)-3-chlorobenzamide (**3-2b**)*

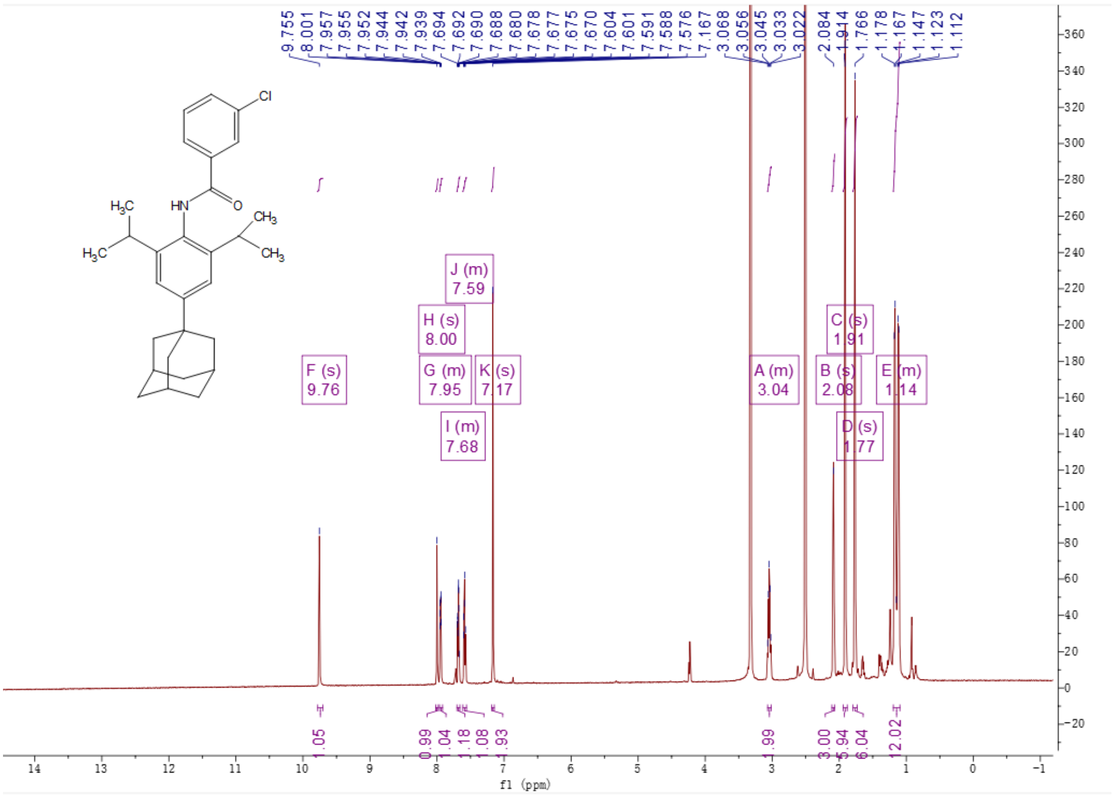

^{1}H NMR

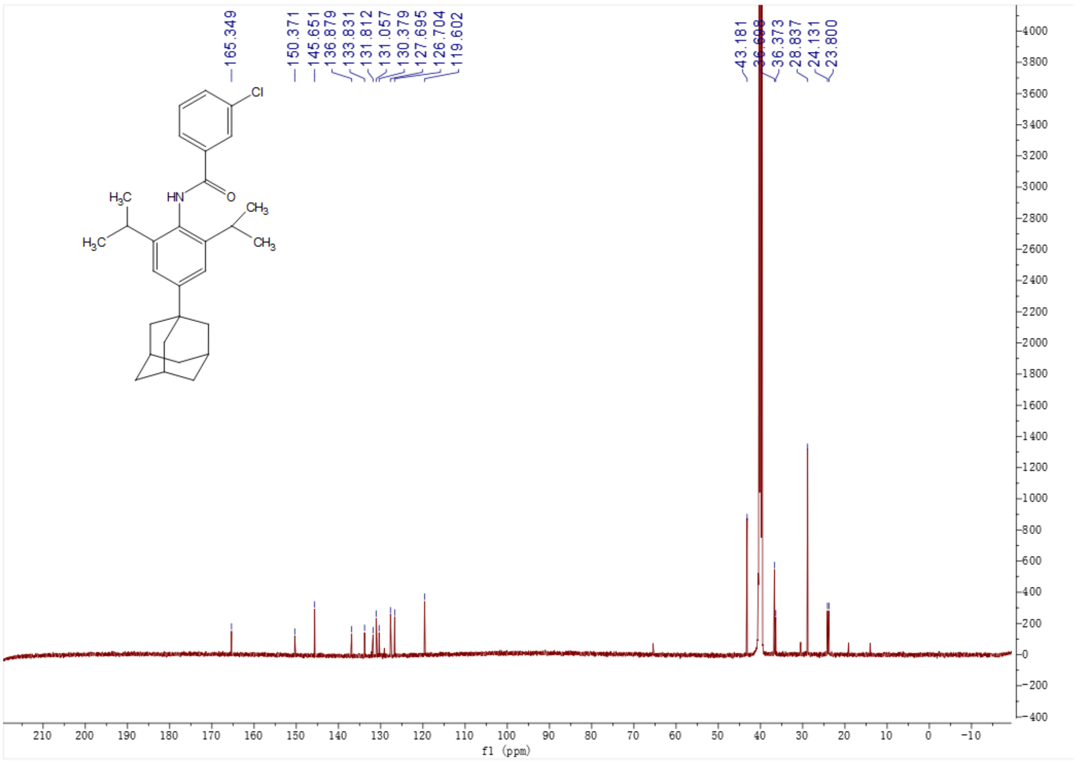

^{13}C NMR

*N-(5-((3r,5r,7r)-adamantan-1-yl)pyrimidin-2-yl)-3-chlorobenzamide(**3-2c**)*

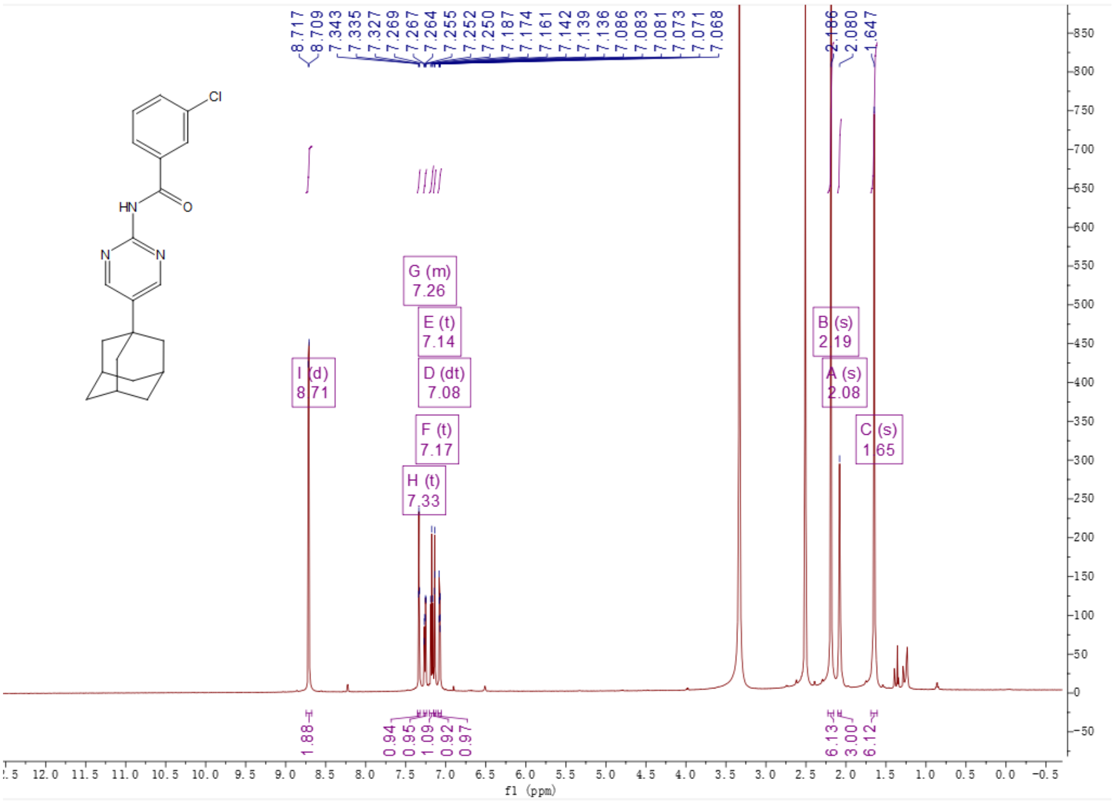

^{1}H NMR

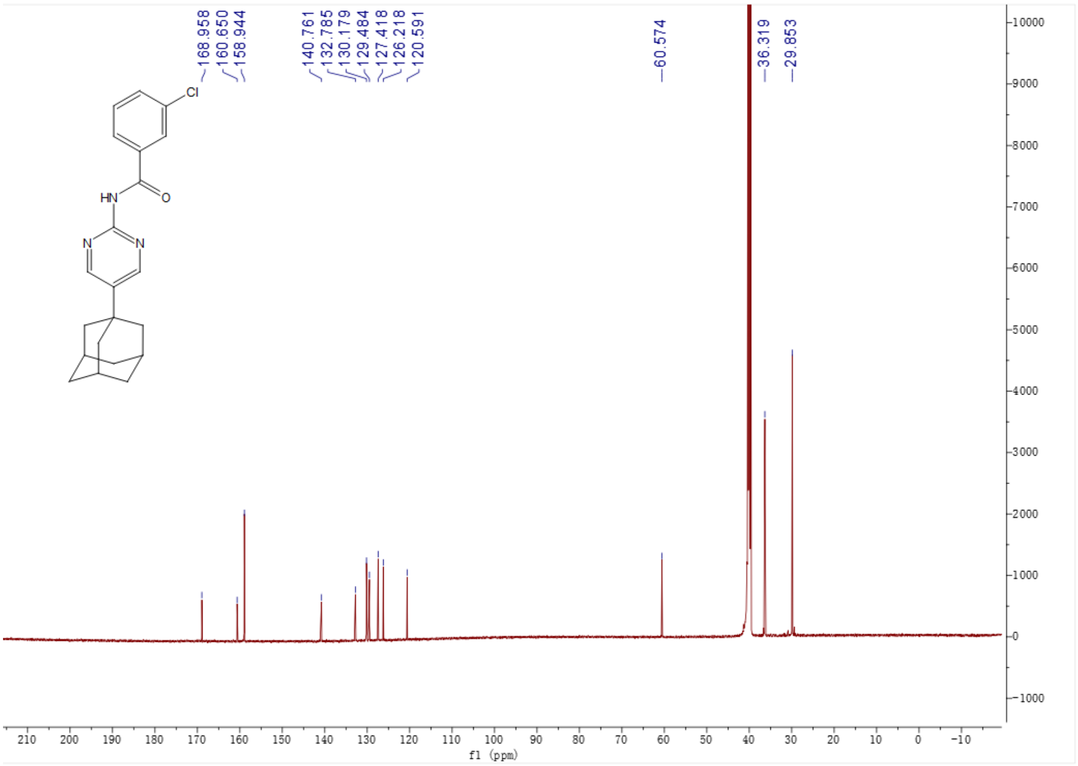

^{13}C NMR

*4-Acetamido-N-(4-((1S,3R,5S,6S,7S)-4,6-dimethyladamantan-1-yl)phenyl)benzamide(**3-2d**)*

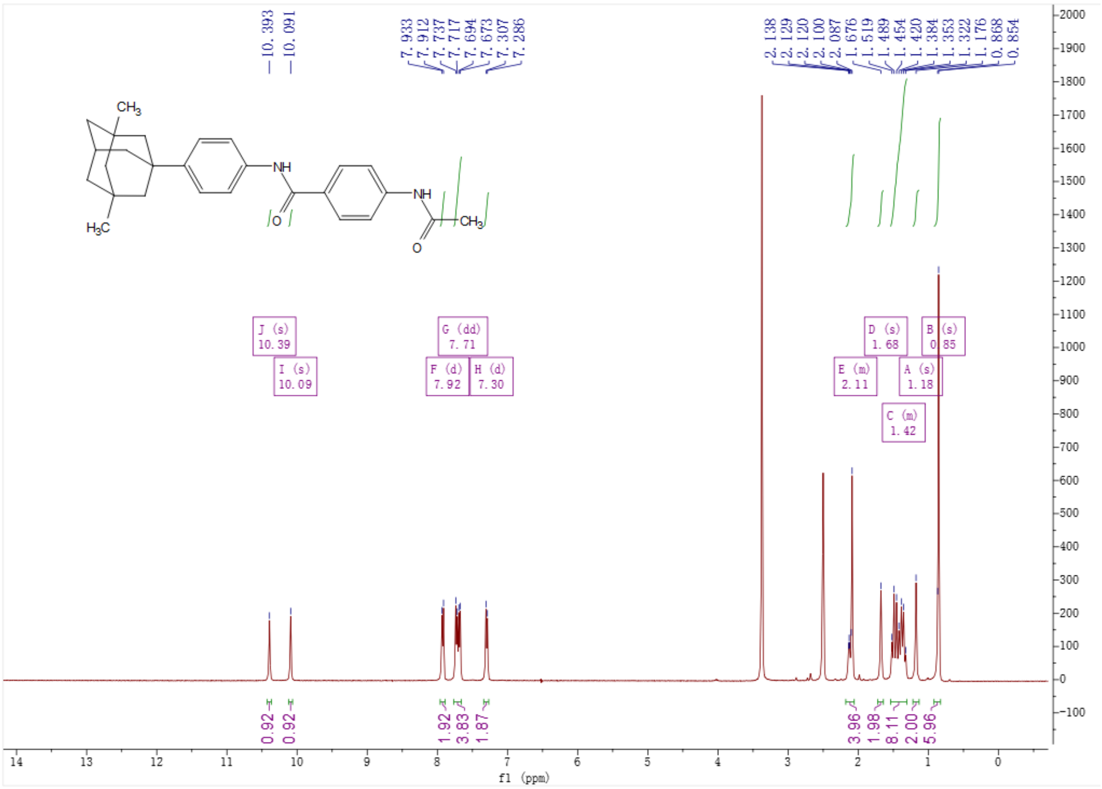

^{1}H NMR

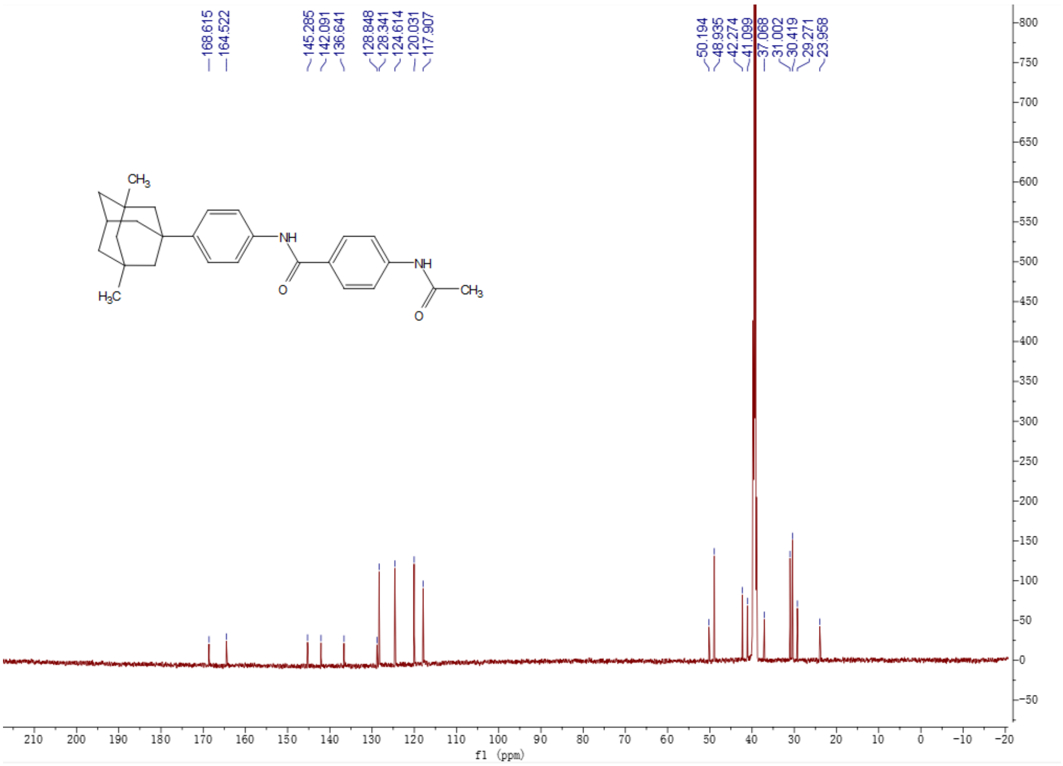

^{13}C NMR

*3-Chloro-N-(4-((1r,3R,5S,7r)-3,5-dimethyladamantan-1-yl)phenyl)benzamide(**3-2e**)*

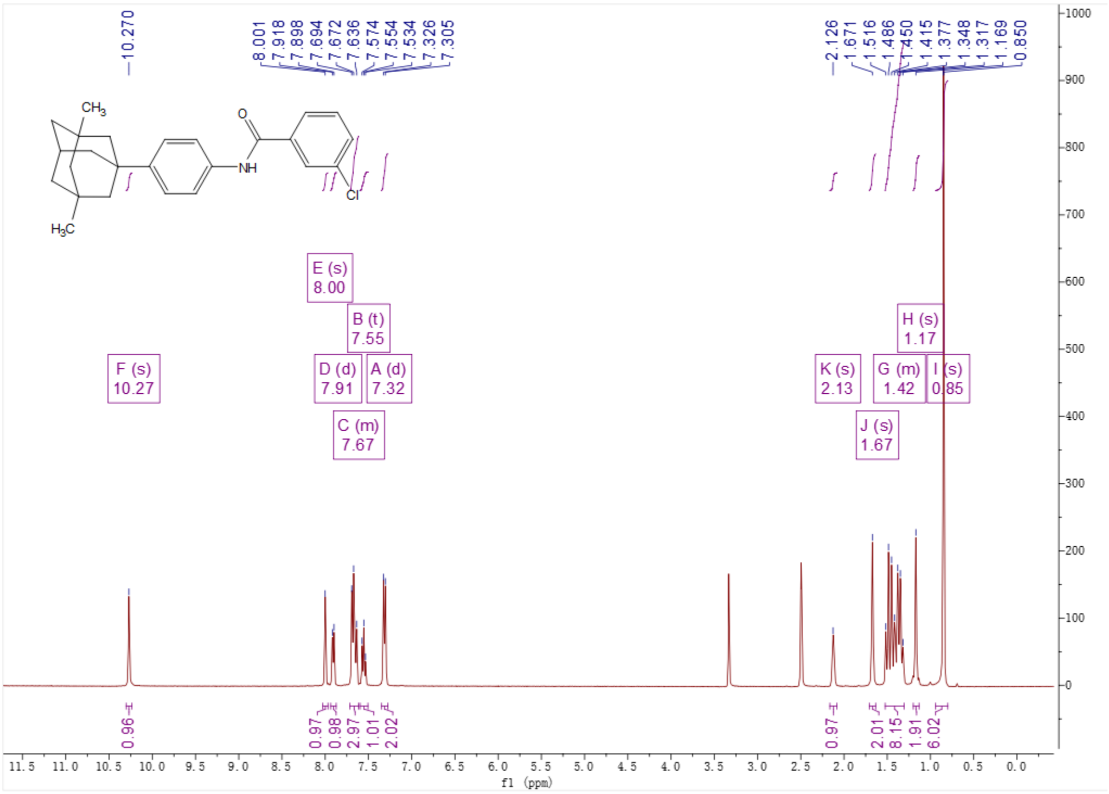

^{1}H NMR

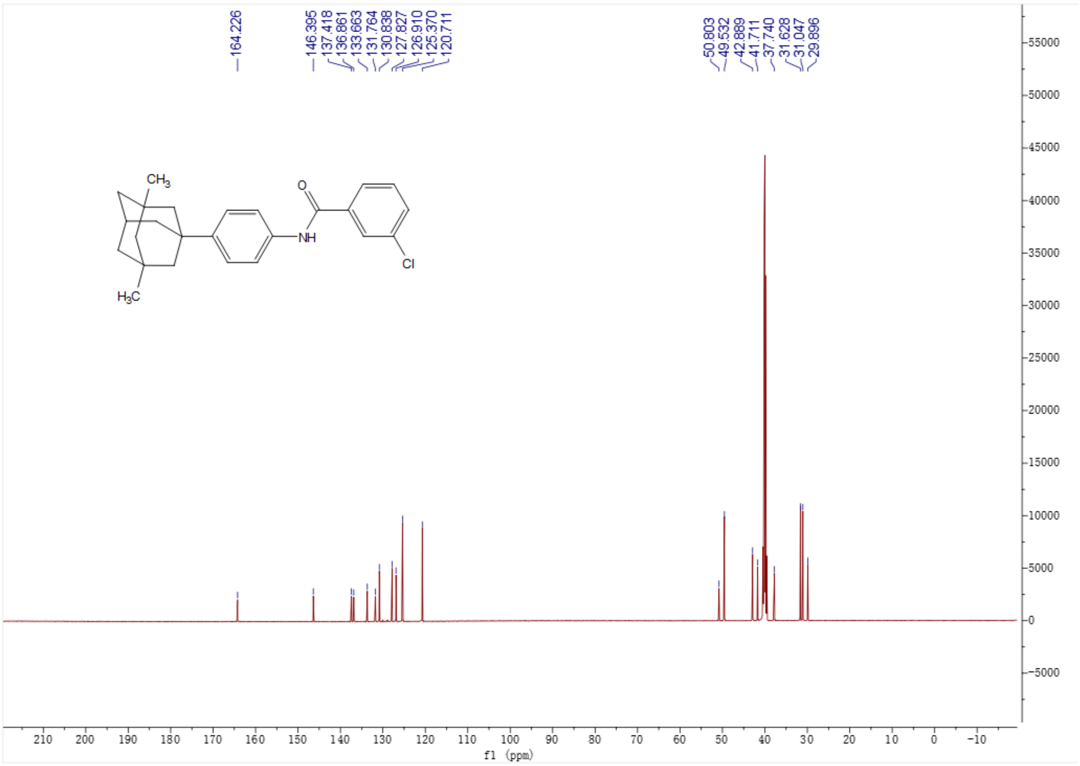

^{13}C NMR

3-Chloro-N-(4-((1r,3R,5S,7r)-3,5-dimethyladamantan-1-yl)-2,6-dimethylphenyl)benzamide ***(3-2f)***

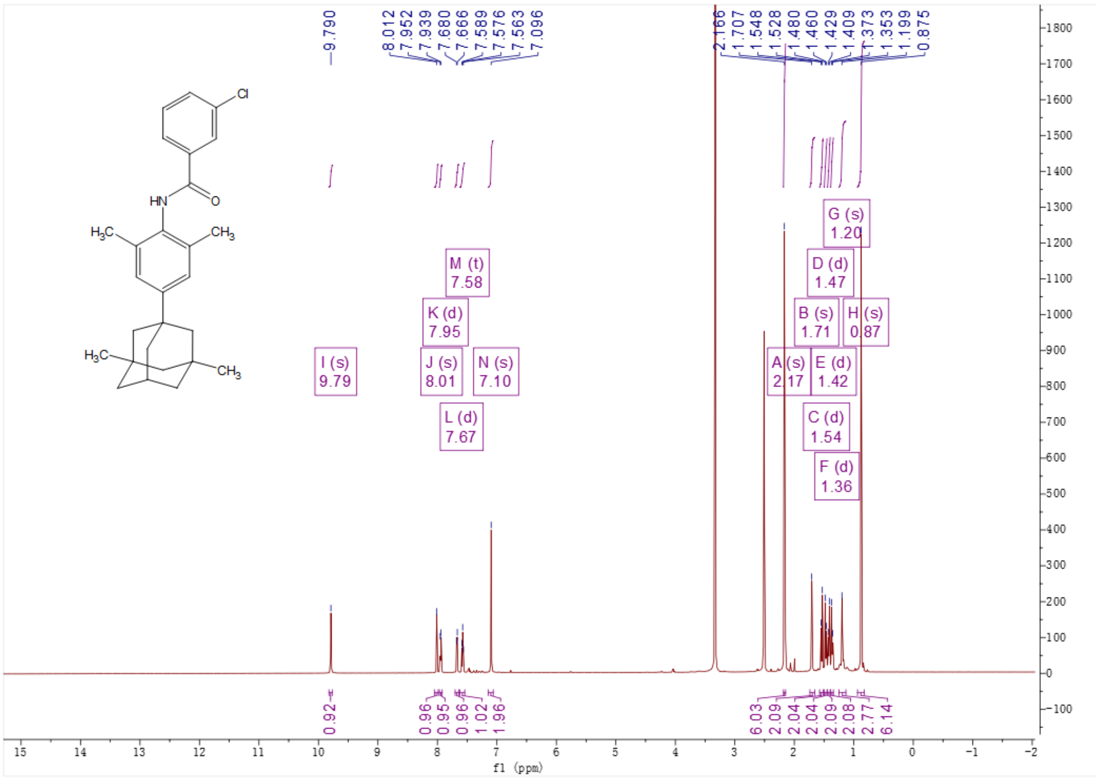

^{1}H NMR

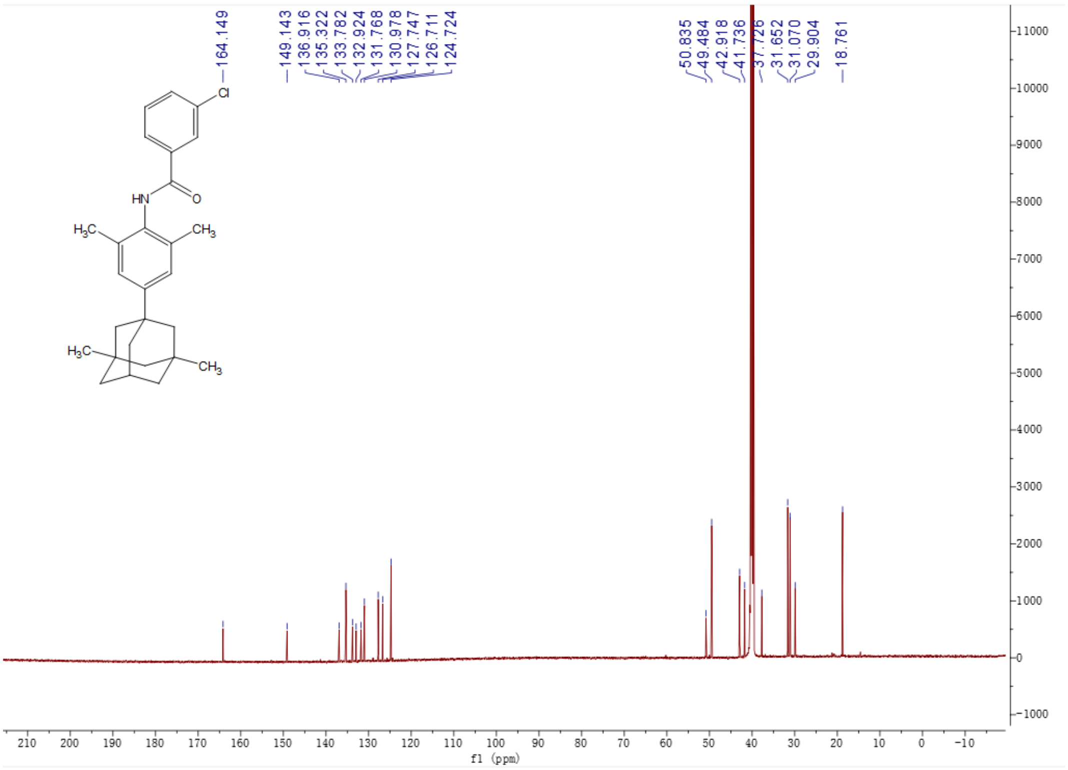

^{13}C NMR

*3-Chloro-N-(5-((1r,3R,5S,7r)-3,5-dimethyladamantan-1-yl)pyrimidin-2-yl)benzamide(**3-2g**)*

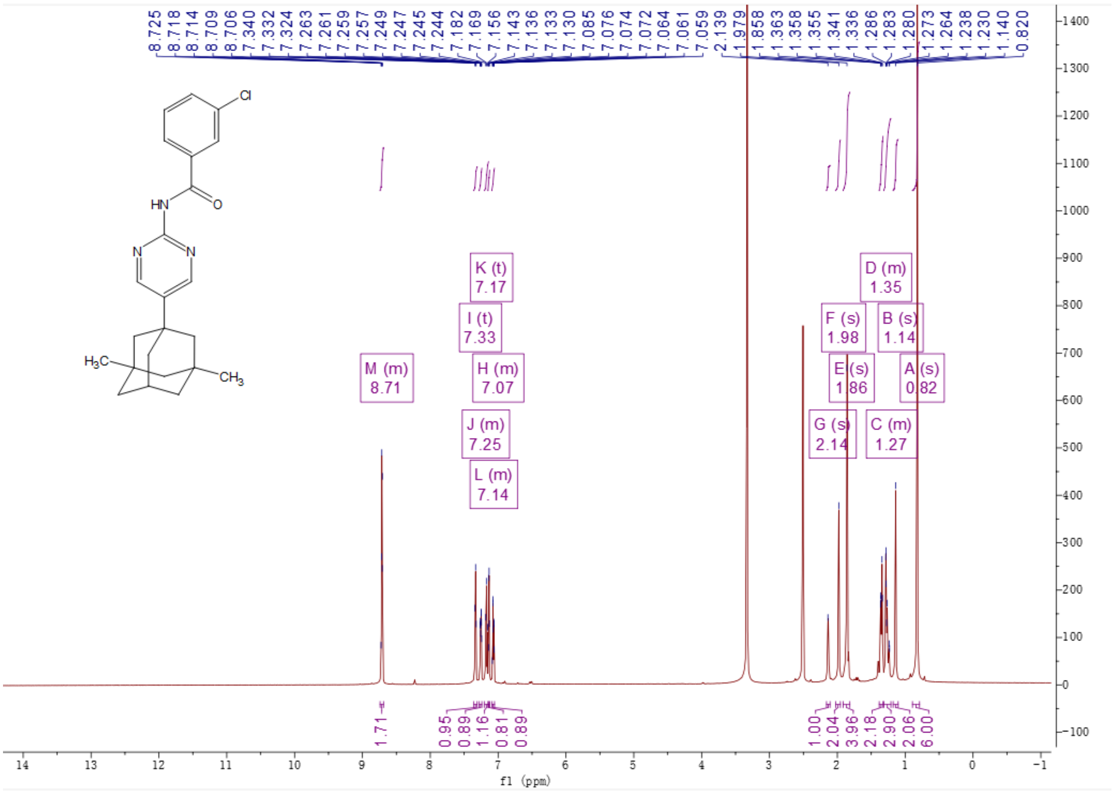

^{1}H NMR

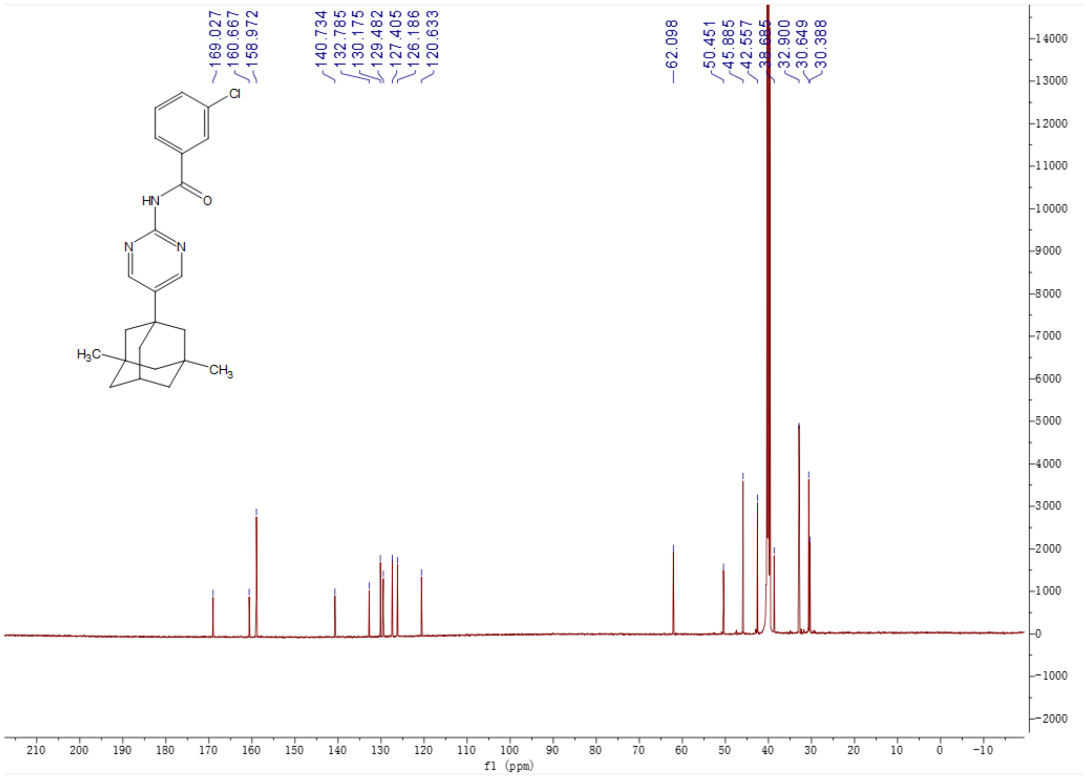

^{13}C NMR

*3-Chloro-N-(4-((1r,3R,5S,7r)-3,5-dimethyladamantan-1-yl)-2,6-diethylphenyl)benzamide (**3-2h**)*

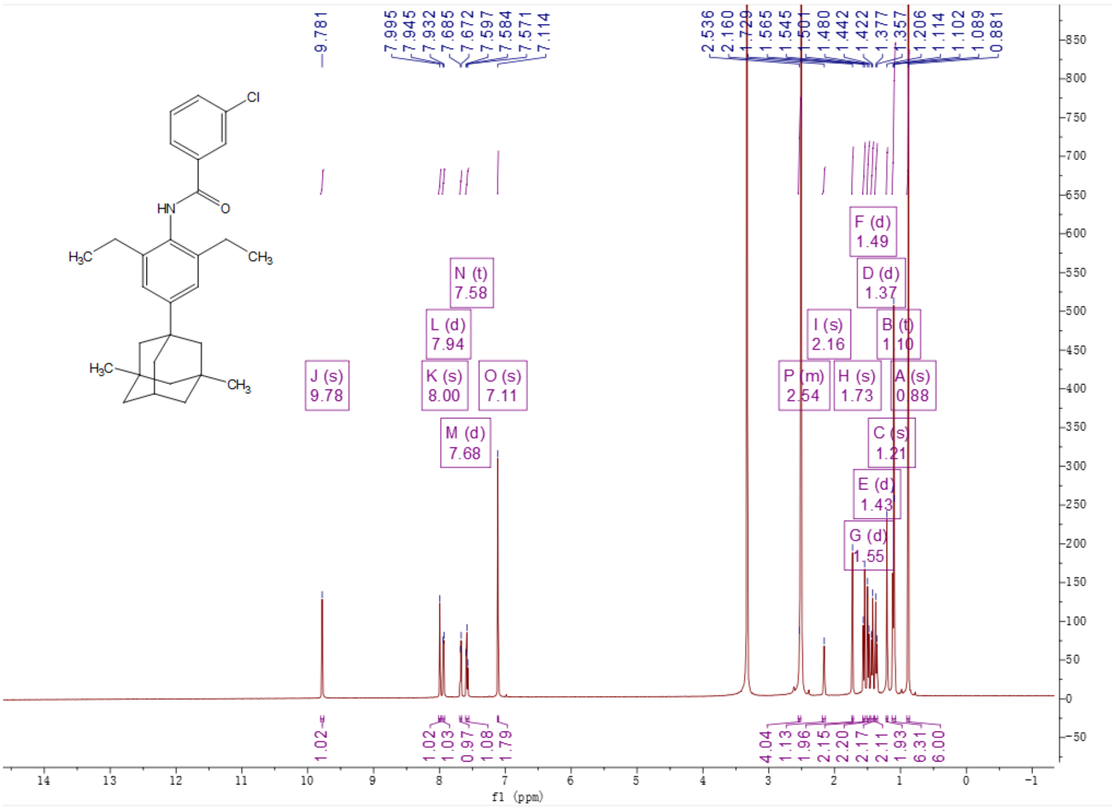

^{1}H NMR

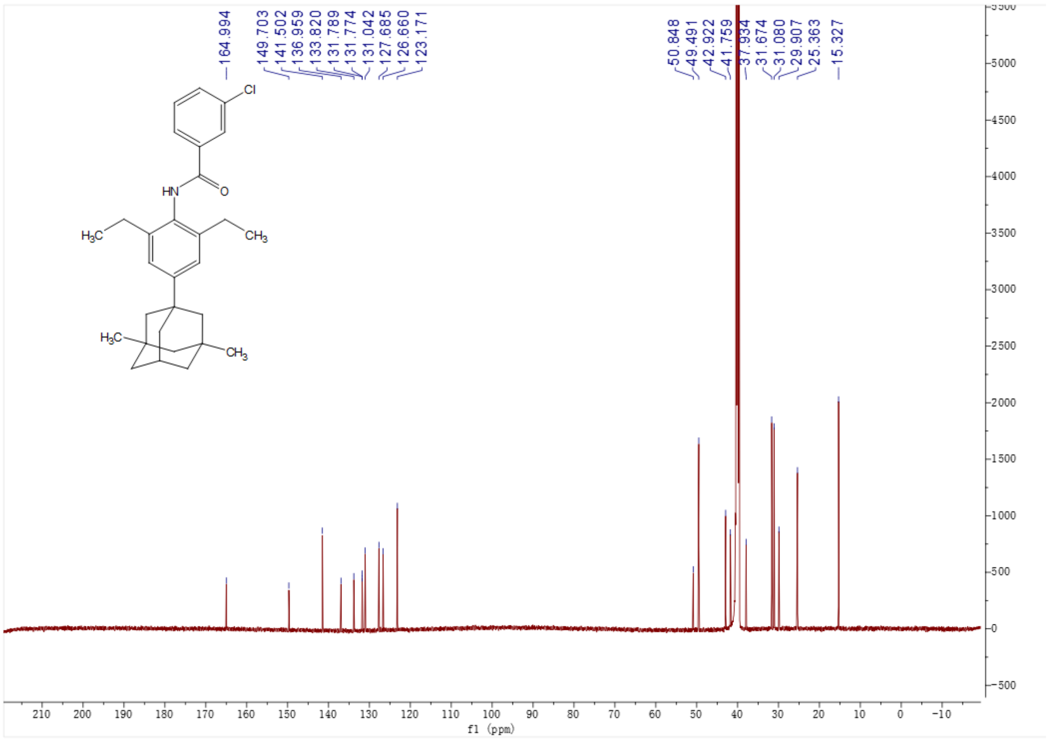

^{13}C NMR

*N-(4-((3r,5r,7r)-adamantan-1-yl)-2,6-diethylphenyl)-3-chlorobenzamide(**3-2i**)*

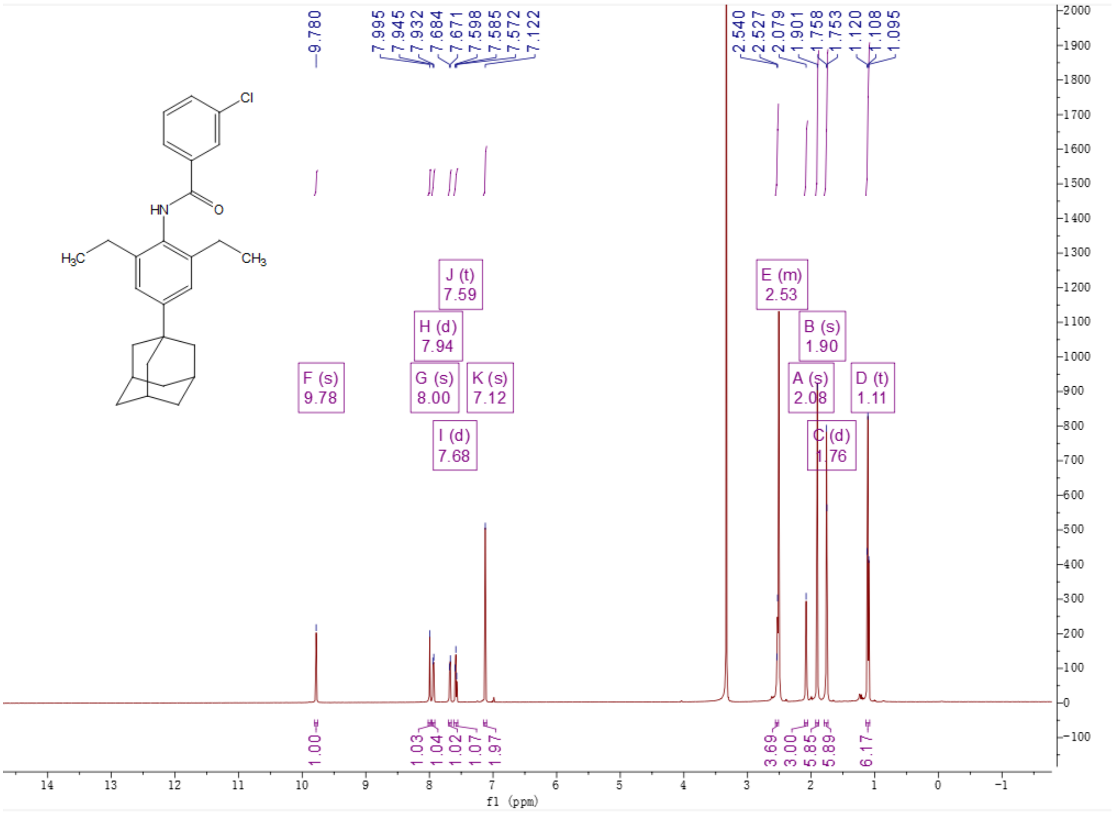

^{1}H NMR

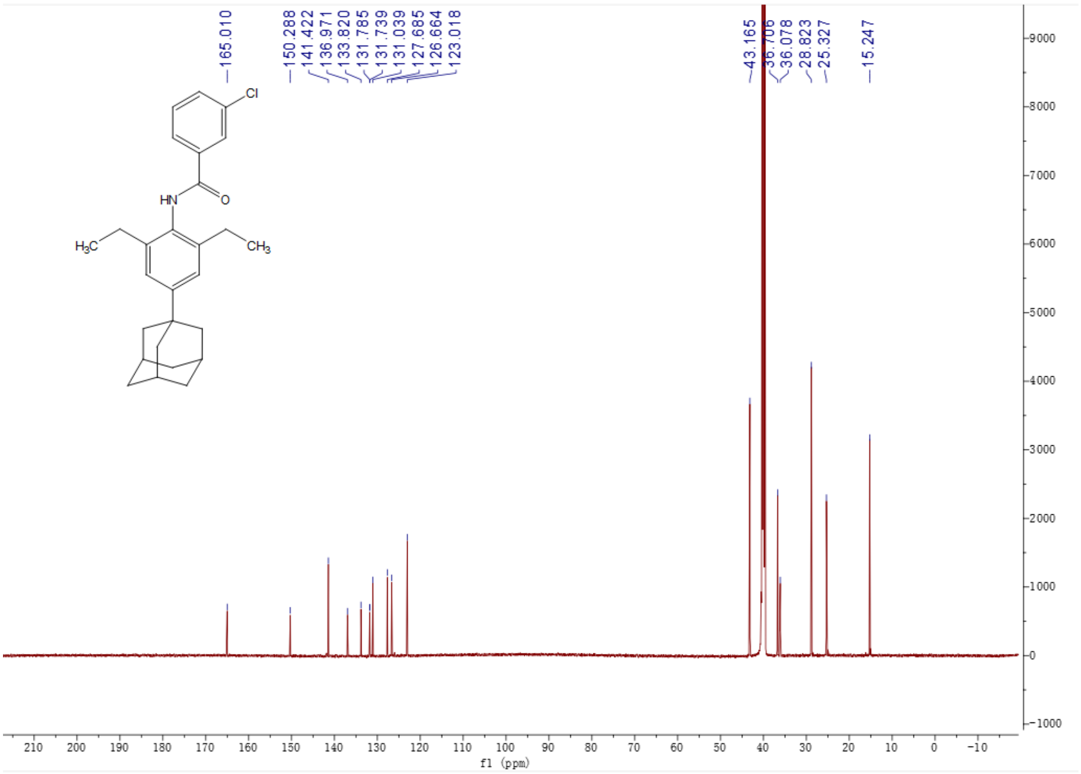

^{13}C NMR

4. Compound Purity

Compound	RT (min)	Area%
1-3a	9.634	100
1-3b	7.219	99.66
1-3c	8.625	98.21
1-3d	8.350	98.72
1-3e	9.834	99.03
1-3f	9.927	98.95
1-3g	8.641	99.27
1-3h	7.784	99.16
1-3i	7.711	99.07
1-3j	8.385	99.42
1-3k	7.519	99.00
1-3l	7.444	96.53
1-3m	8.847	97.05
1-3n	7.570	99.47
1-3o	7.601	99.83
1-3p	9.037	100
1-3q	9.020	100
1-3r	10.589	100
1-3s	7.176	98.38
1-3t	6.601	99.60
1-3u	6.978	99.53
1-3v	8.565	98.72
1-3w	9.055	99.70
1-3x	7.929	98.41
1-3y	7.528	99.65
1-3z	10.071	96.96
1-3aa	6.907	100
1-3ab	7.390	99.20
1-3ac	9.287	99.92
1-3ad	8.028	99.25
1-3ae	8.385	98.30
1-3af	9.374	100
1-3ag	5.834	96.44
1-3ah	8.231	98.65
1-3ai	4.993	98.74
1-3aj	12.970	99.14
1-3ak	7.199	99.75
1-3al	8.722	98.81
1-3am	6.415	97.75
1-3an	6.586	99.85
1-3ao	8.537	98.31
1-3ap	6.499	99.84
1-3aq	6.620	99.64

1-3ar	8.976	99.68
1-3as	6.547	99.90
1-3at	6.600	99.86
1-3au	6.607	98.67
1-4a	4.379	99.93
1-4b	8.574	98.98
1-4c	7.233	99.68
1-4d	9.331	98.71
1-4e	3.393	95.92
1-4f	9.533	99.72
1-4g	9.531	96.04
1-4h	4.142	97.62
1-4i	7.238	99.94
1-4j	7.498	99.03
1-4k	7.092	99.68
1-4l	5.825	98.02
1-4m	7.088	99.40
1-4n	7.625	99.07
1-4o	7.538	98.07
1-4p	6.457	95.76
1-4q	9.550	99.55
1-4r	7.435	97.99
2-3a	6.750	97.75
2-3b	6.858	98.81
2-3c	7.061	99.77
2-3d	5.670	96.08
2-3e	7.260	97.38
3-2a	7.869	98.04
3-2b	9.490	99.10
3-2c	6.224	99.52
3-2d	8.595	97.34
3-2e	10.945	99.86
3-2f	9.769	97.74
3-2g	6.691	98.90
3-2h	10.528	98.42
3-2i	8.564	96.38

Table S1. Purity of targeted compounds analyzed by HPLC

Figure S17. Graphic purity of targeted compounds analyzed by HPLC

1-3a

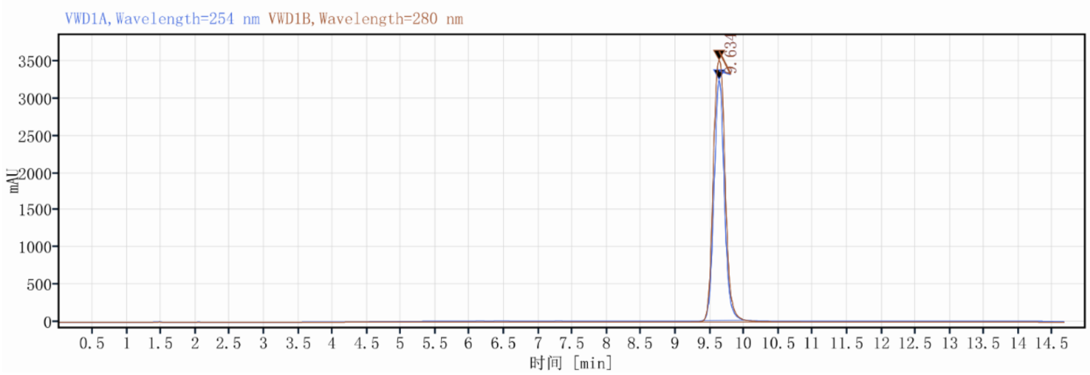

1-3b

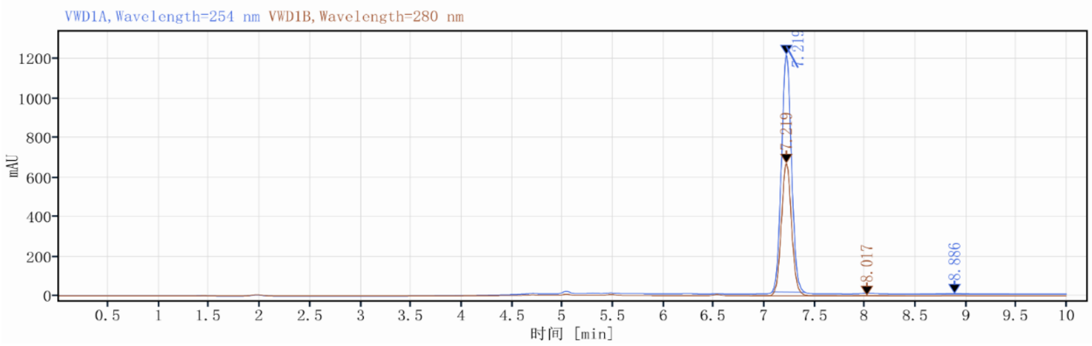

1-3c

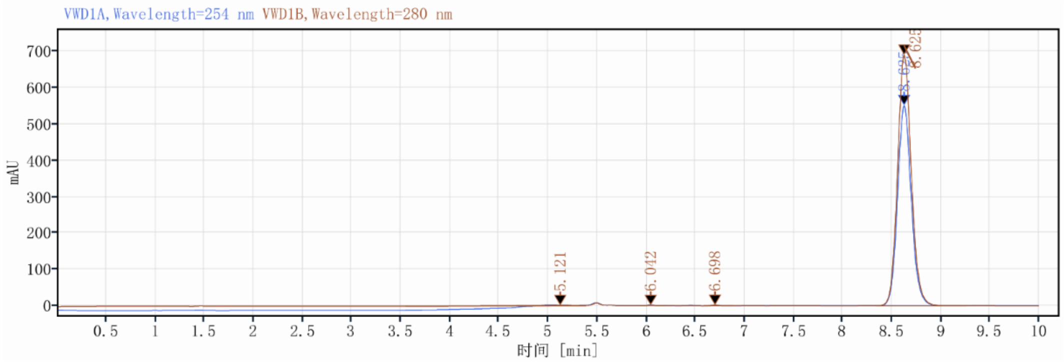

1-3d

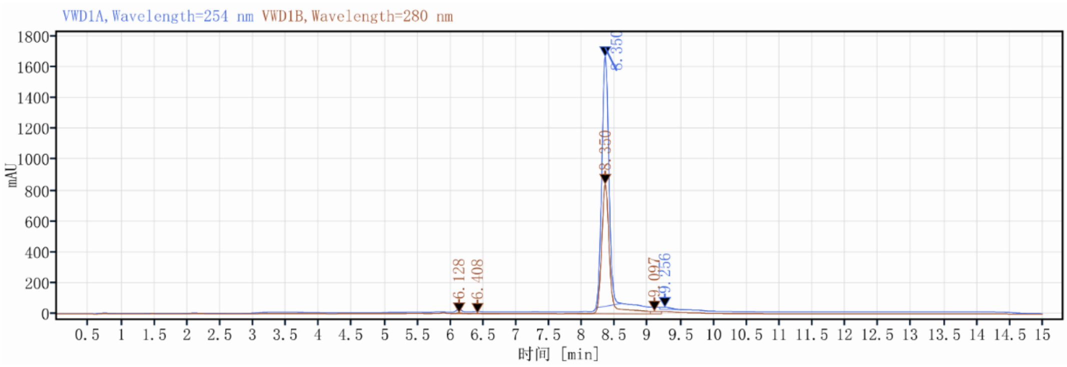

1-3e

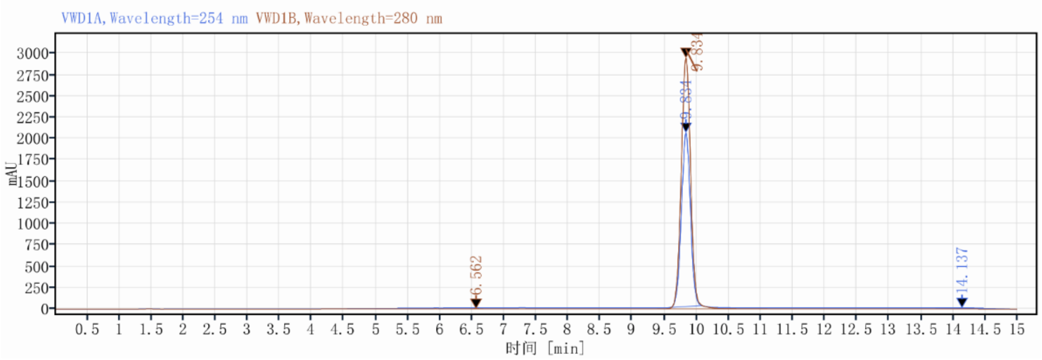

1-3f

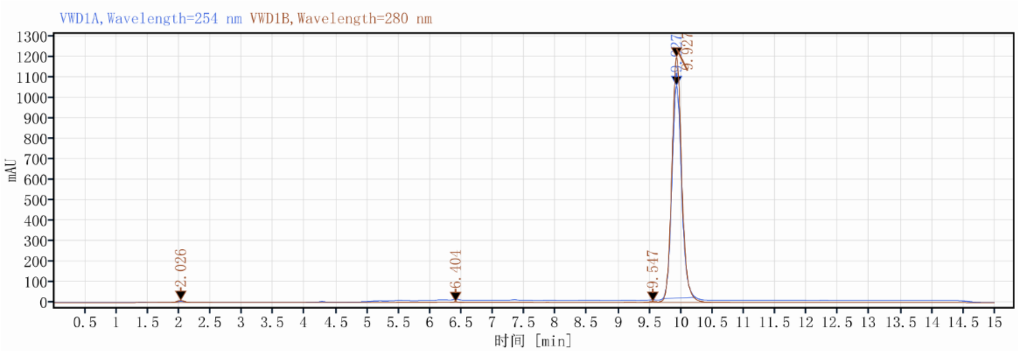

1-3g

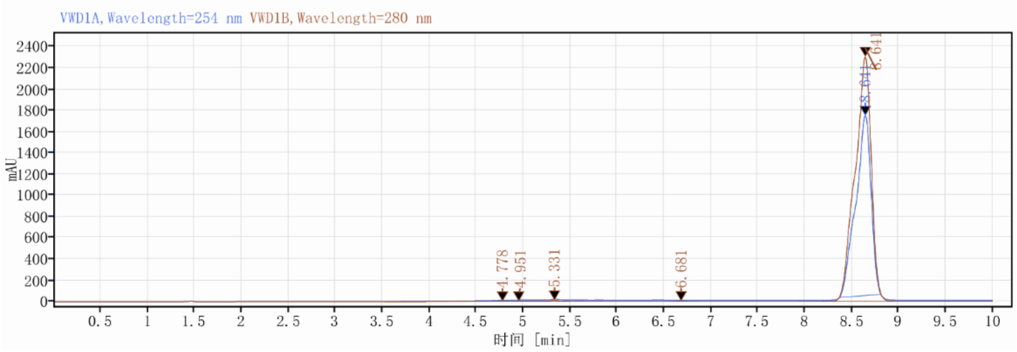

1-3h

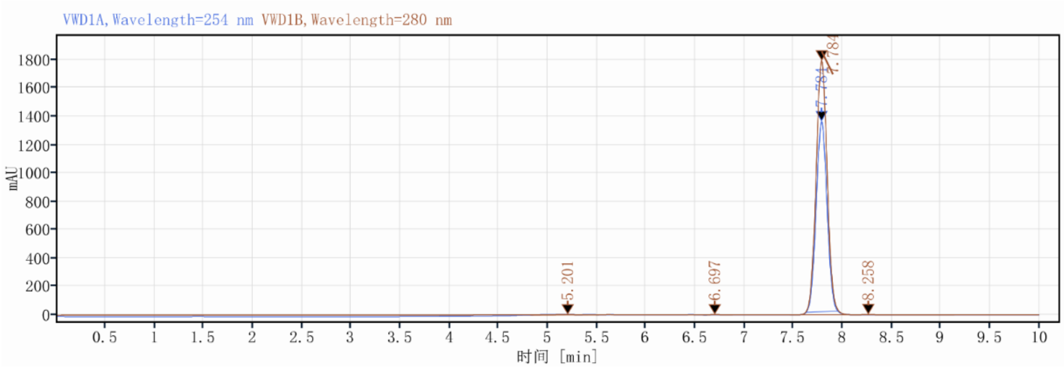

1-3i

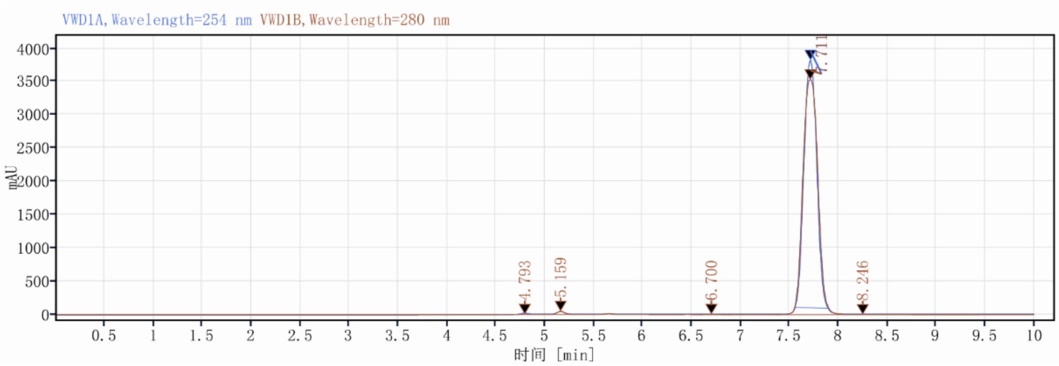

1-3j

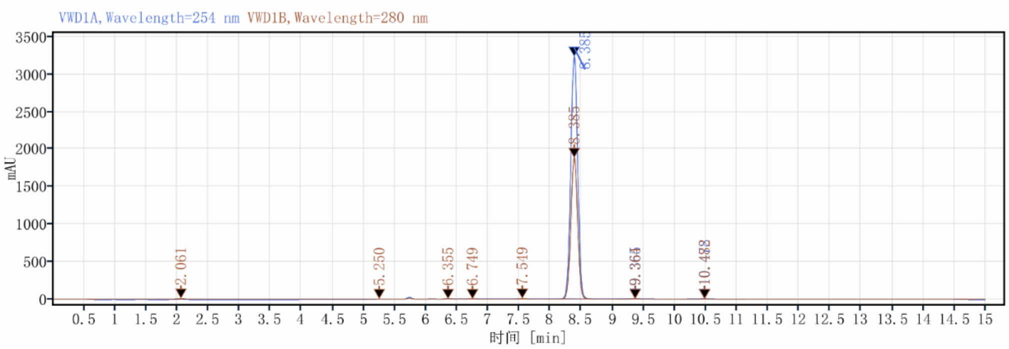

1-3k

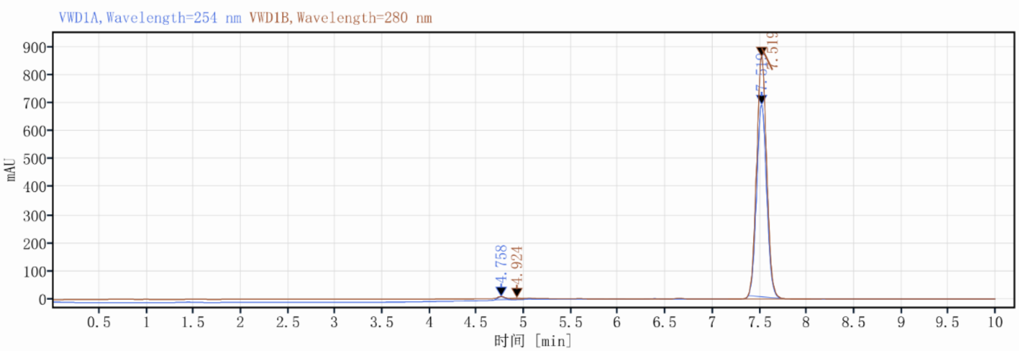

1-3l

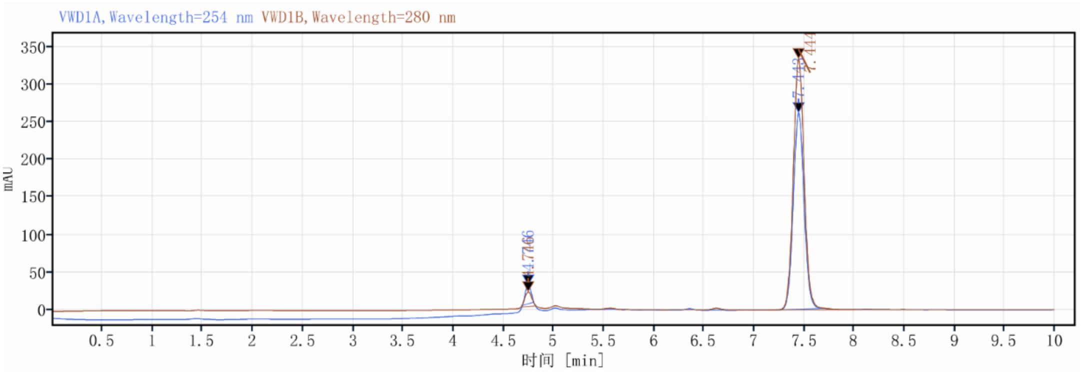

1-3m

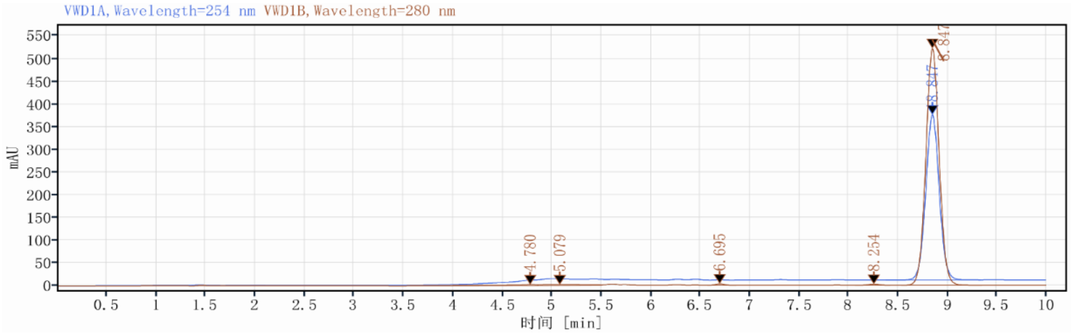

1-3n

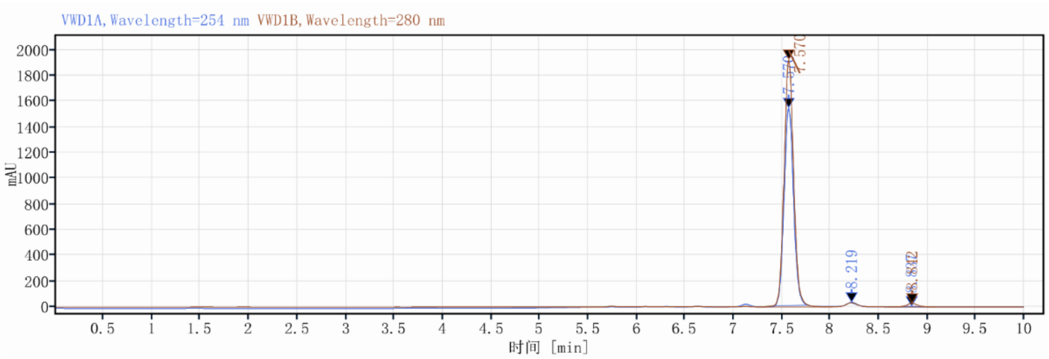

1-3o

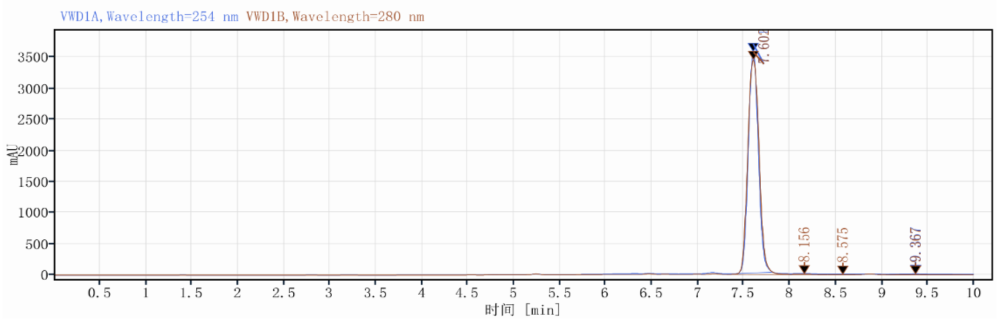

1-3p

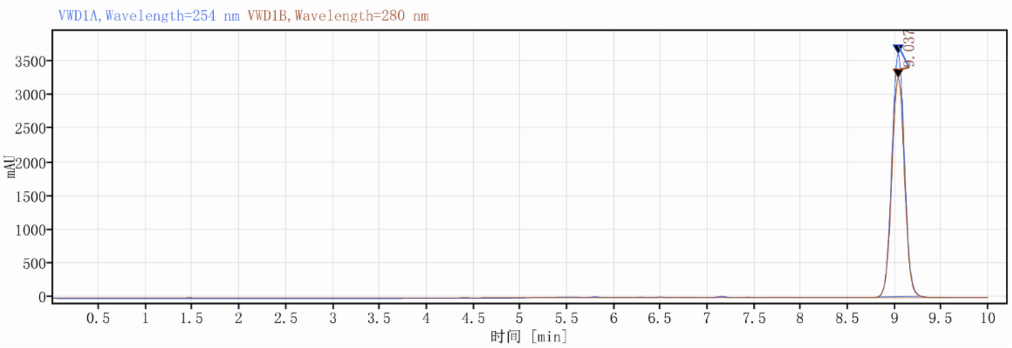

1-3q

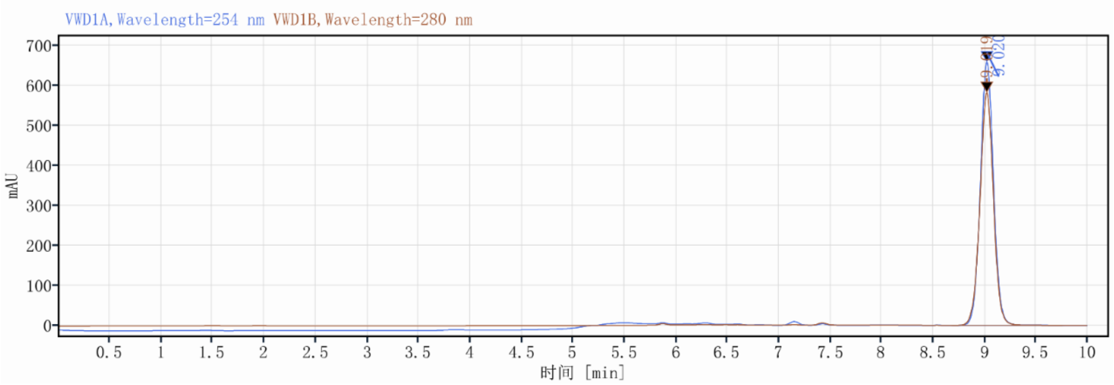

1-3r

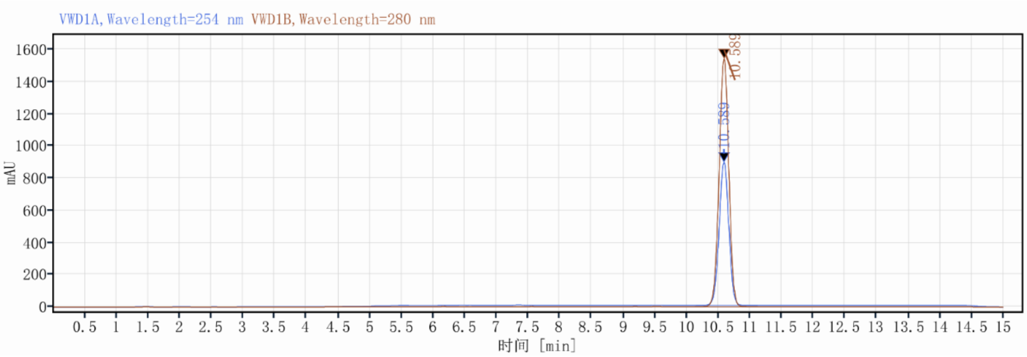

1-3s

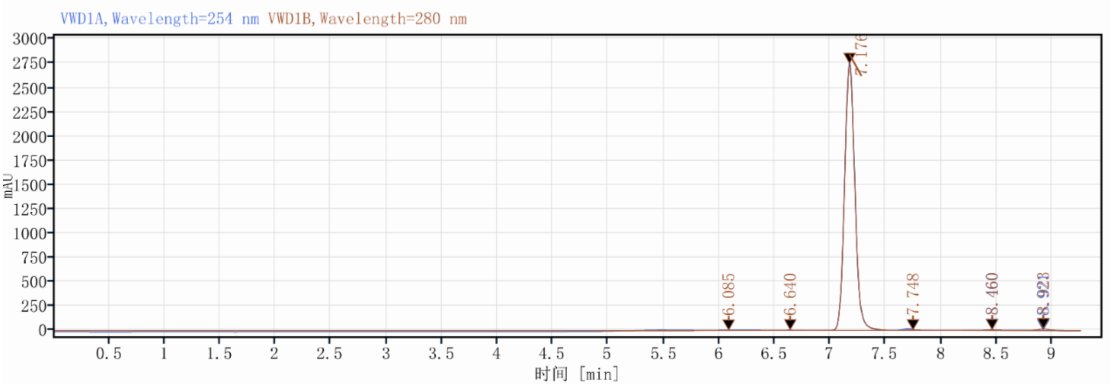

1-3t

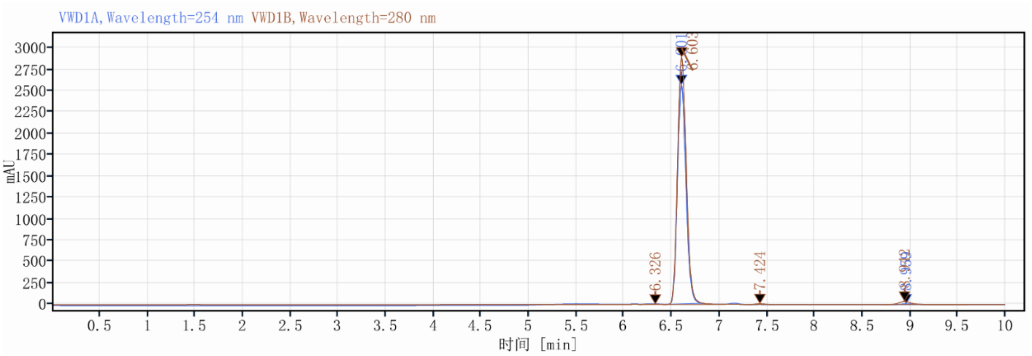

1-3u

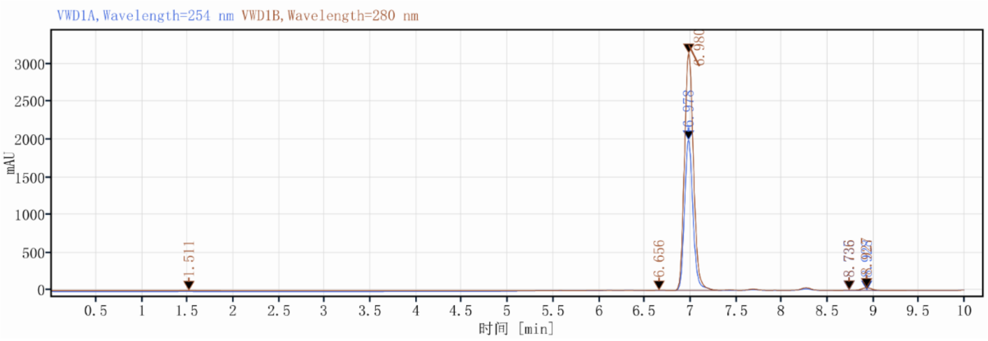

1-3v

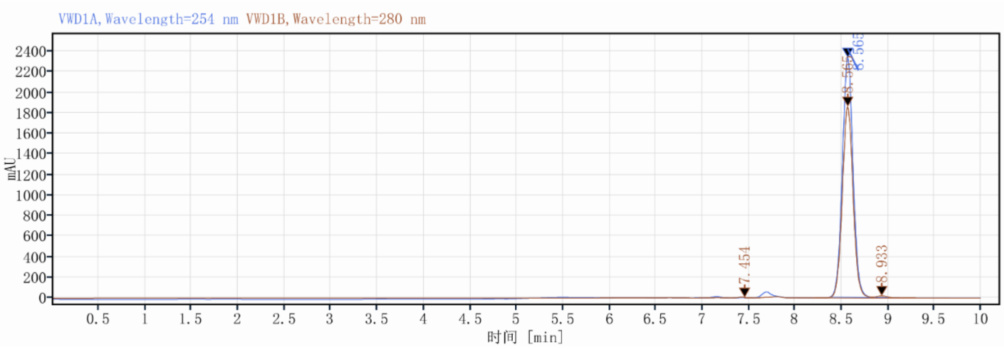

1-3w

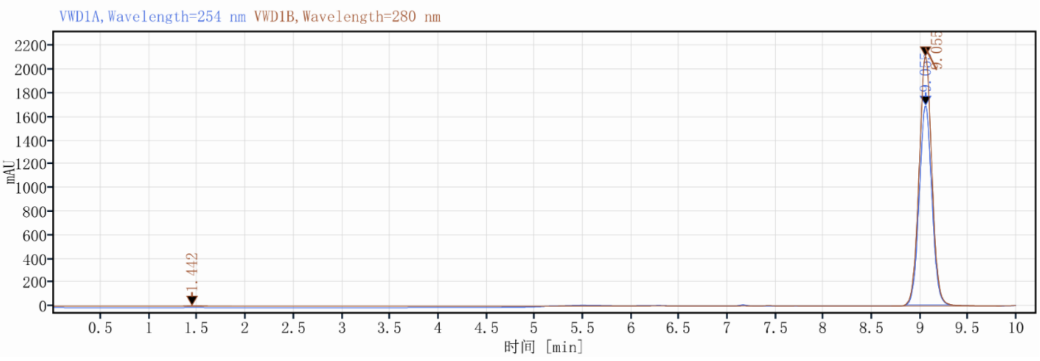

1-3x

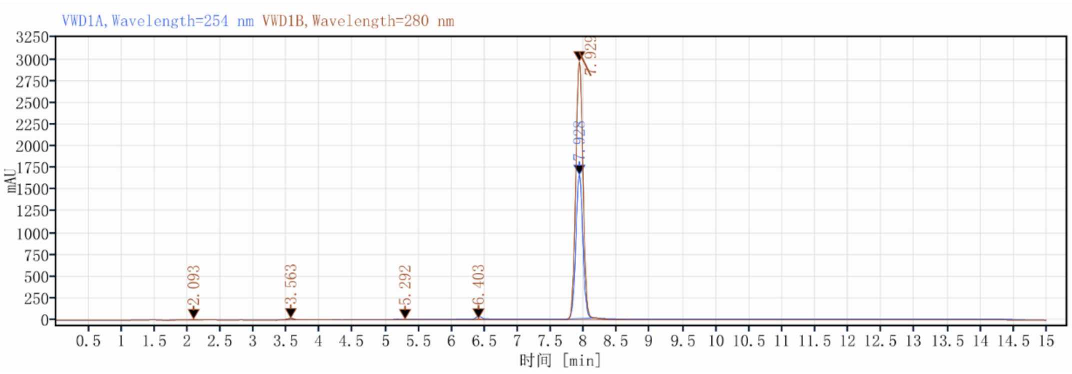

1-3y

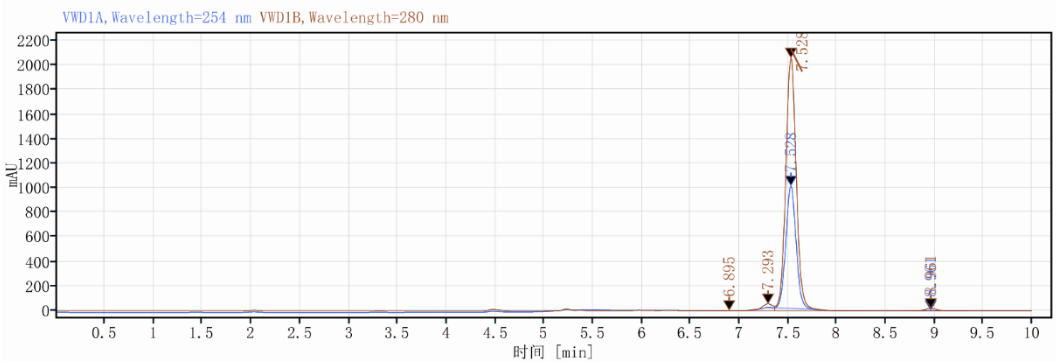

1-3z

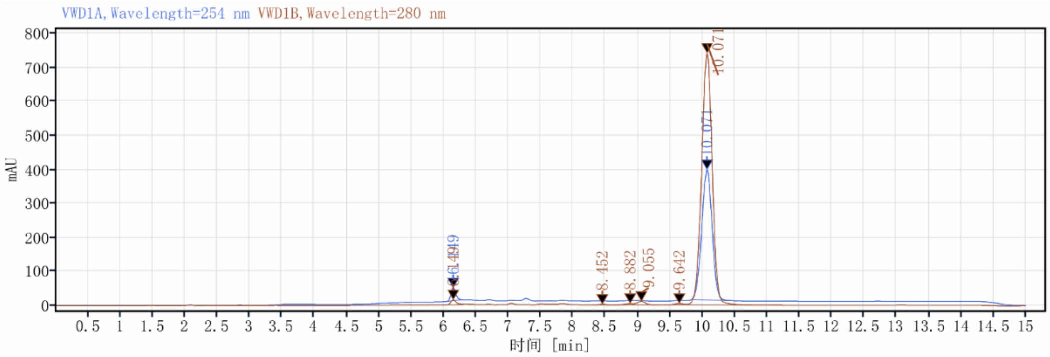

1-3aa

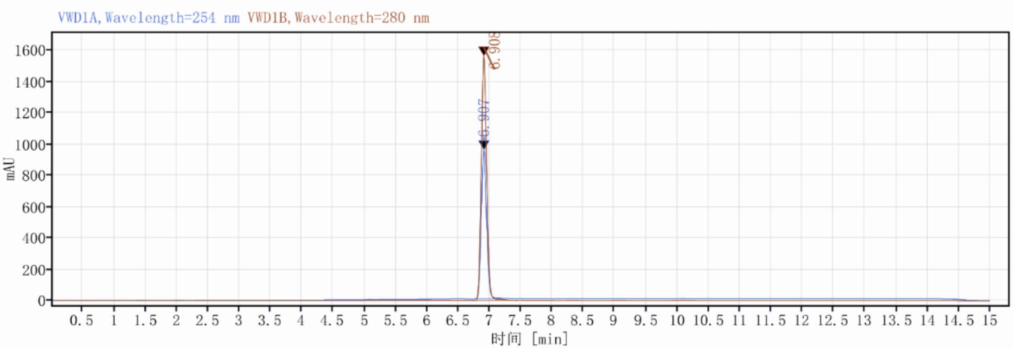

1-3ab

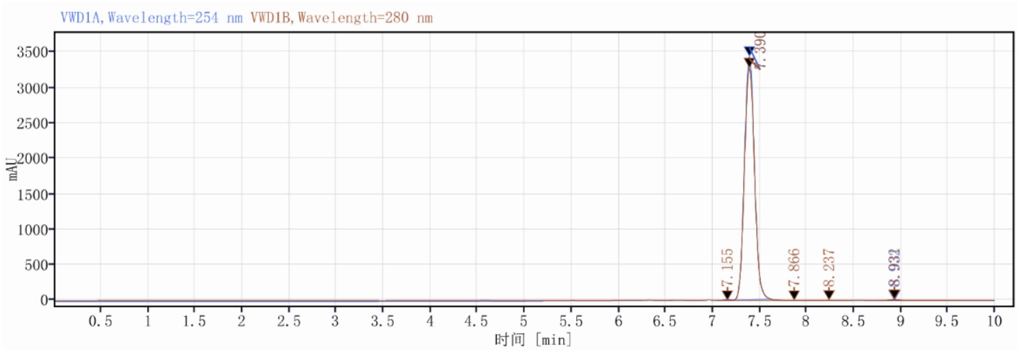

1-3ac

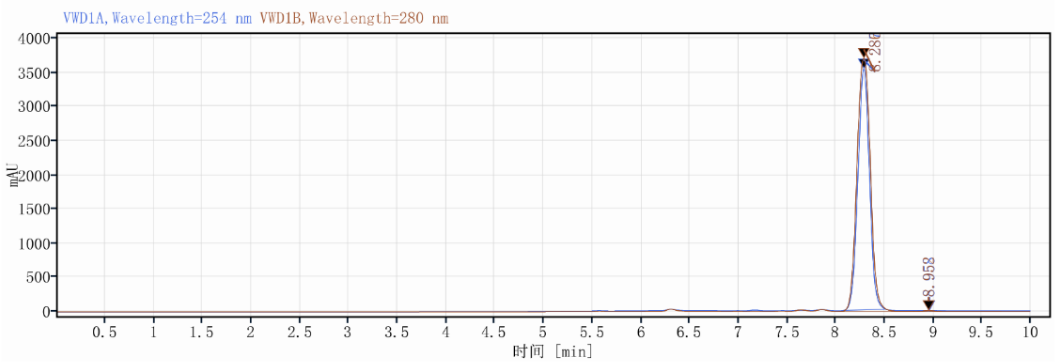

1-3ad

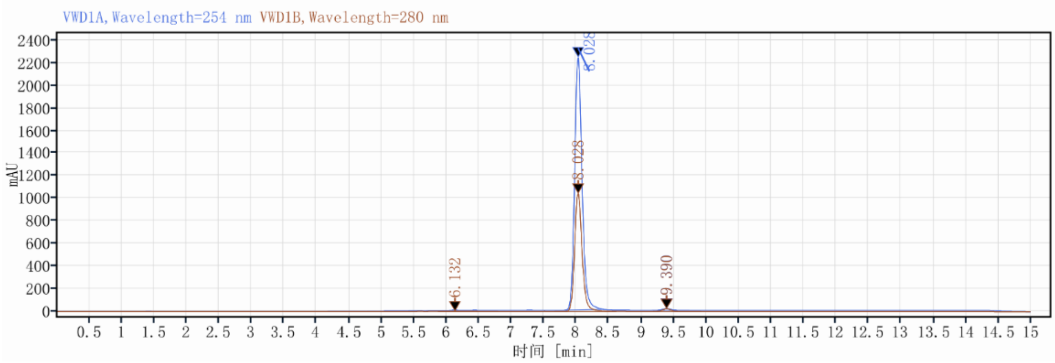

1-3ae

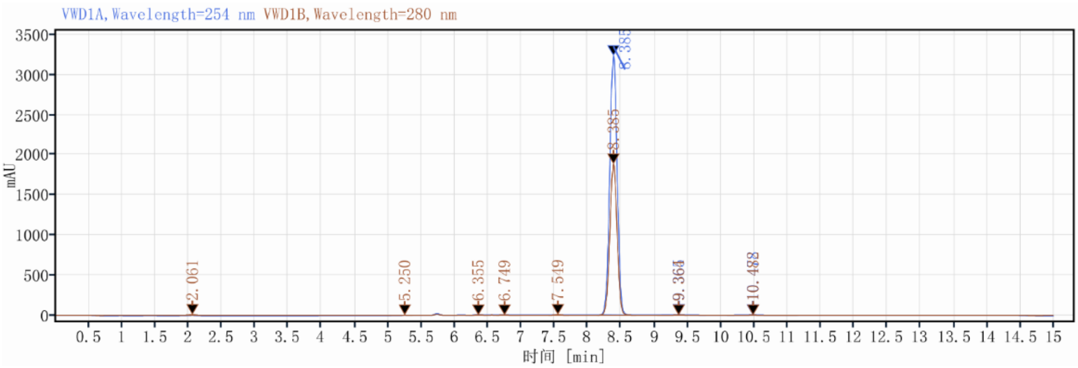

1-3af

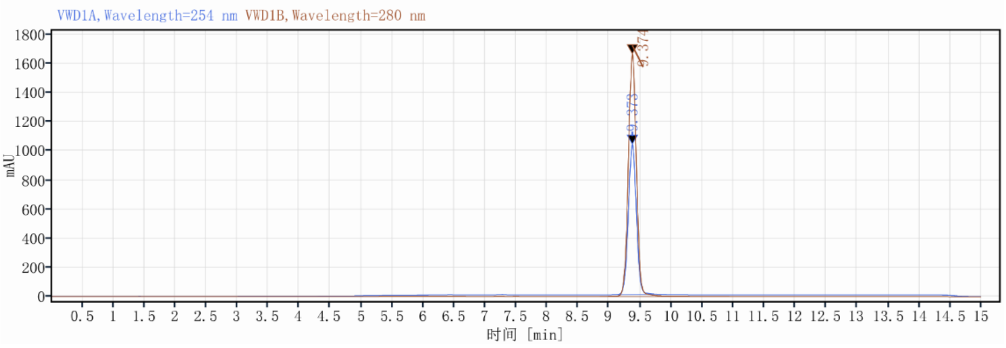

1-3ag

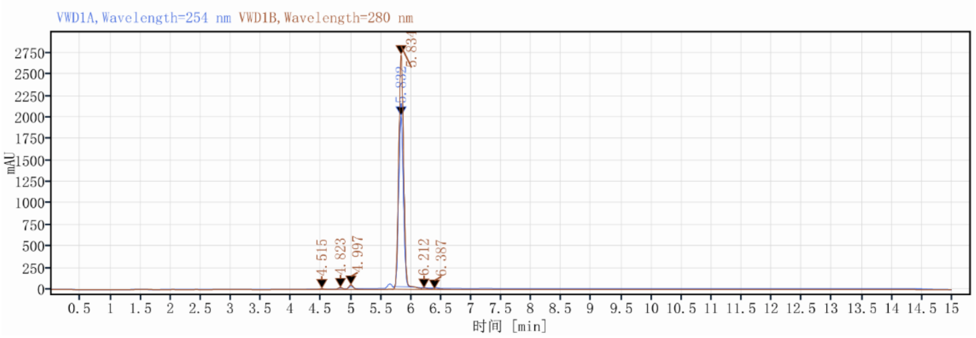

1-3ah

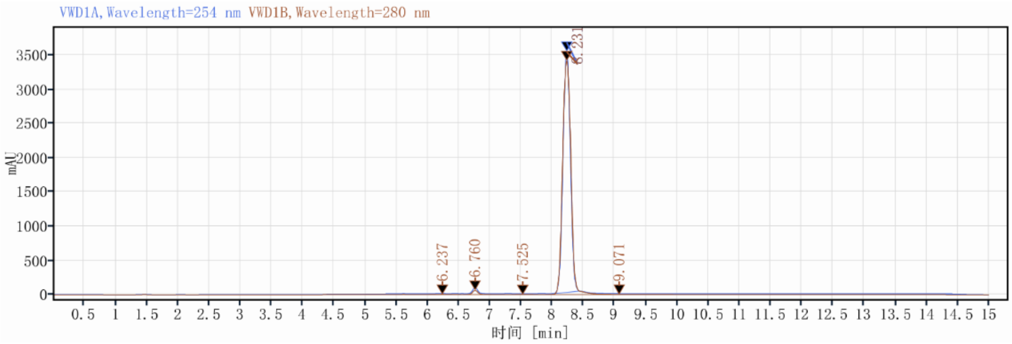

1-3ai

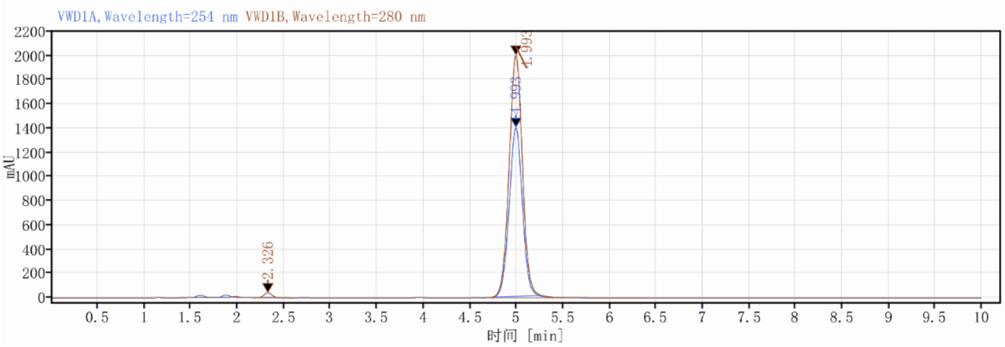

1-3aj

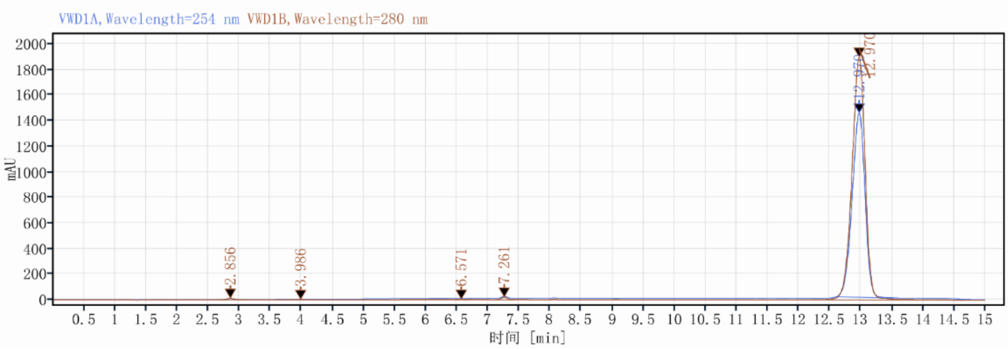

1-3ak

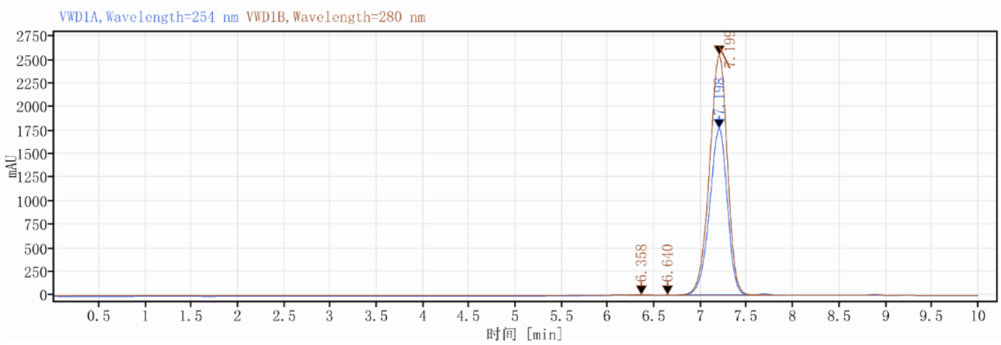

1-3al

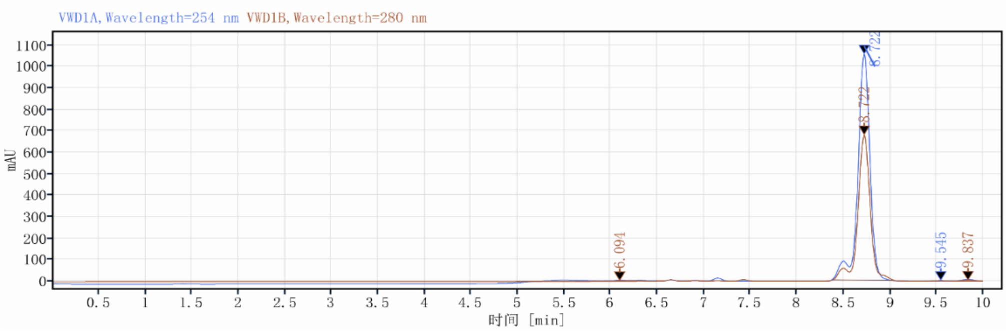

1-3am

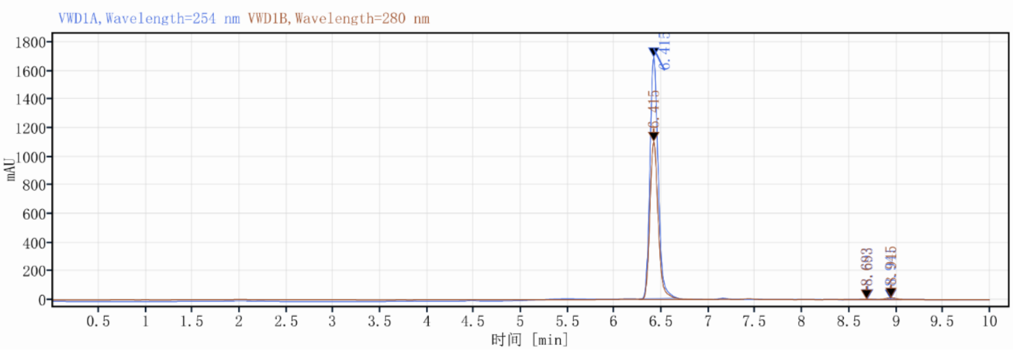

1-3an

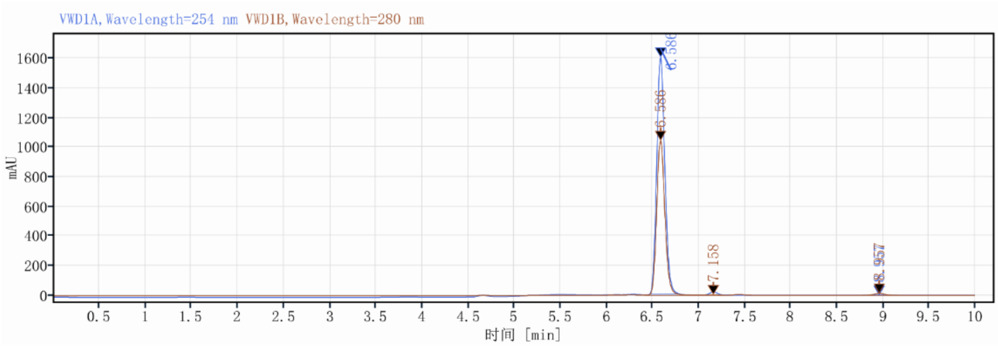

1-3ao

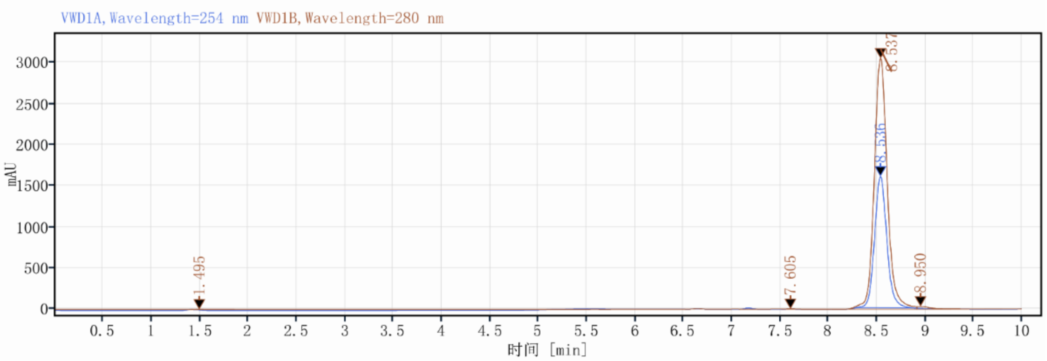

1-3ap

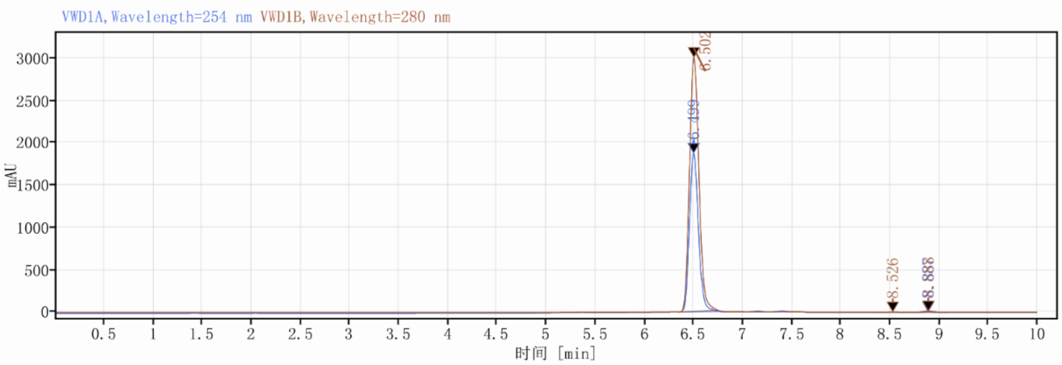

1-3aq

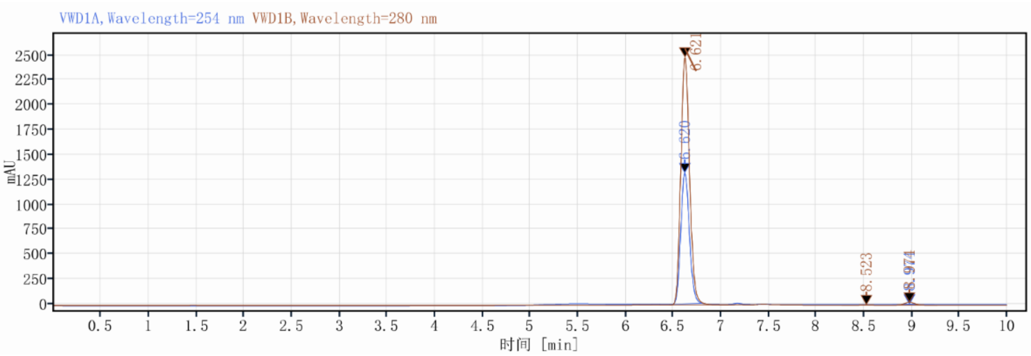

1-3ar

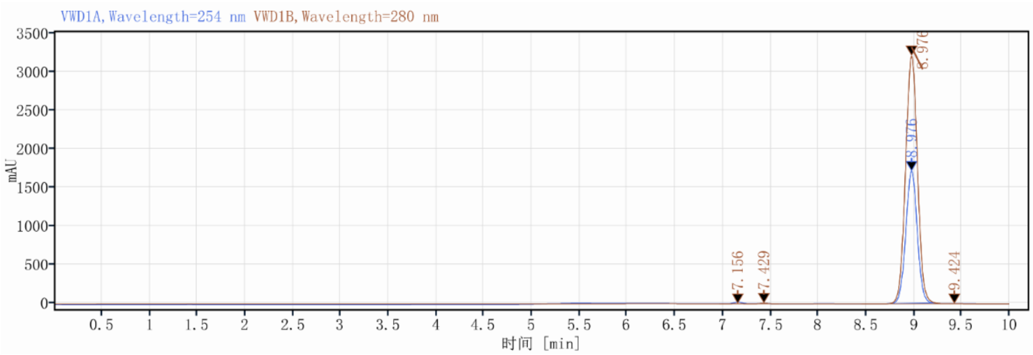

1-3as

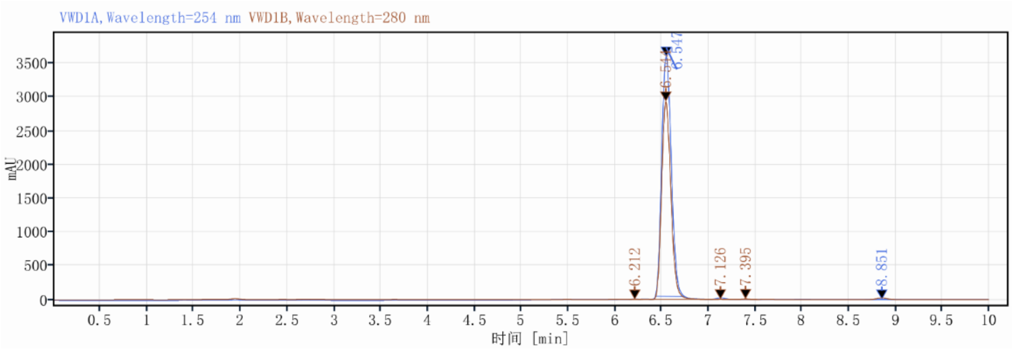

1-3at

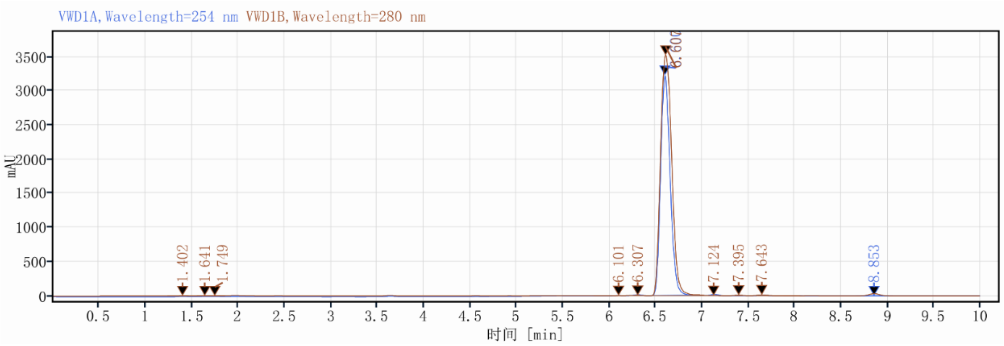

1-3au

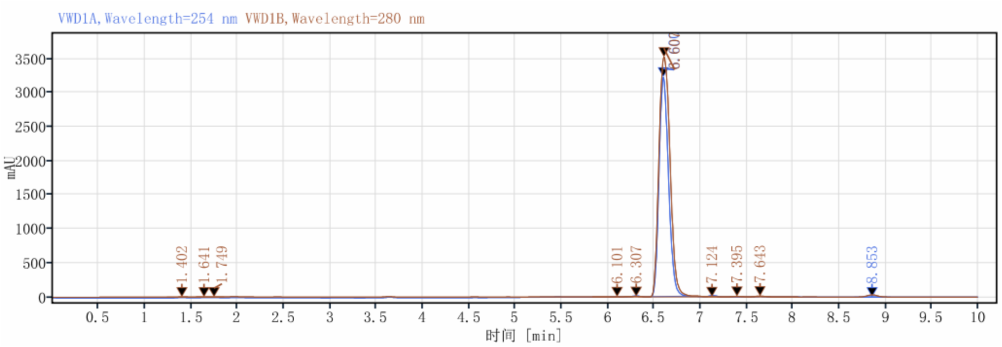

1-4a

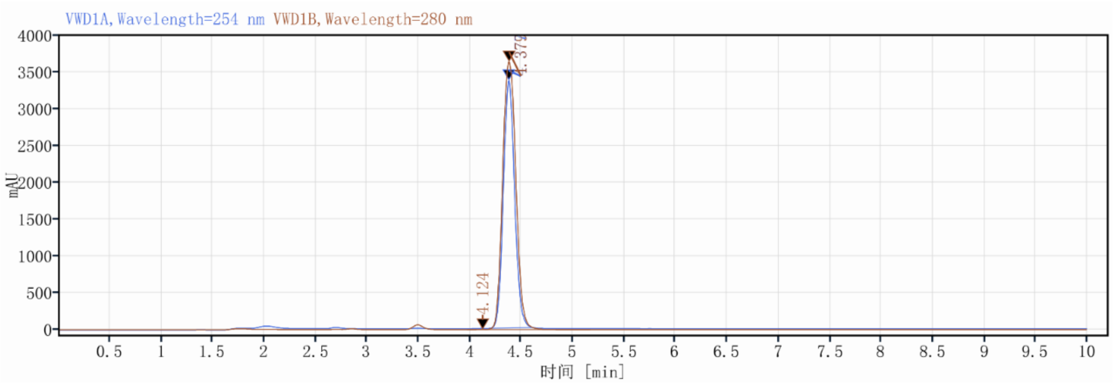

1-4b

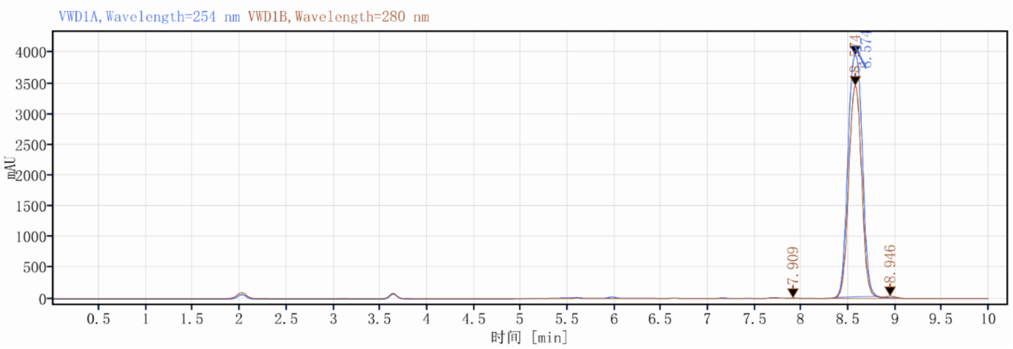

1-4c

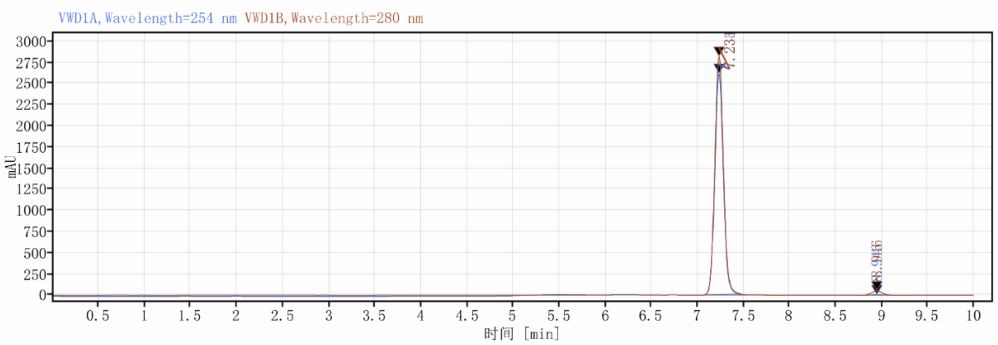

1-4d

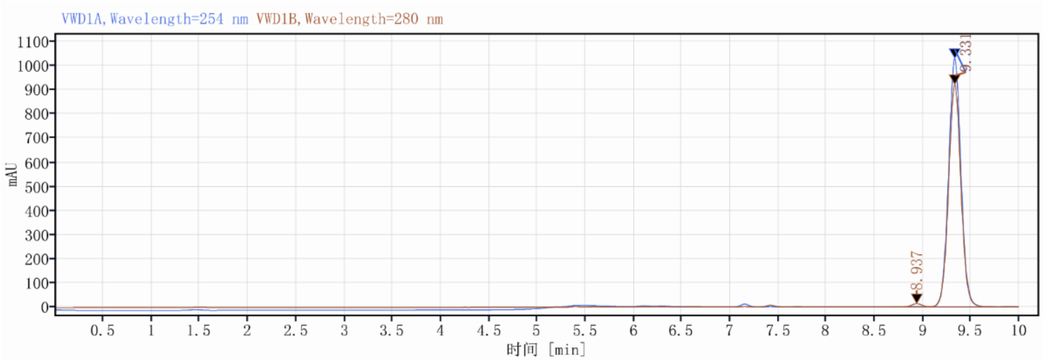

1-4e

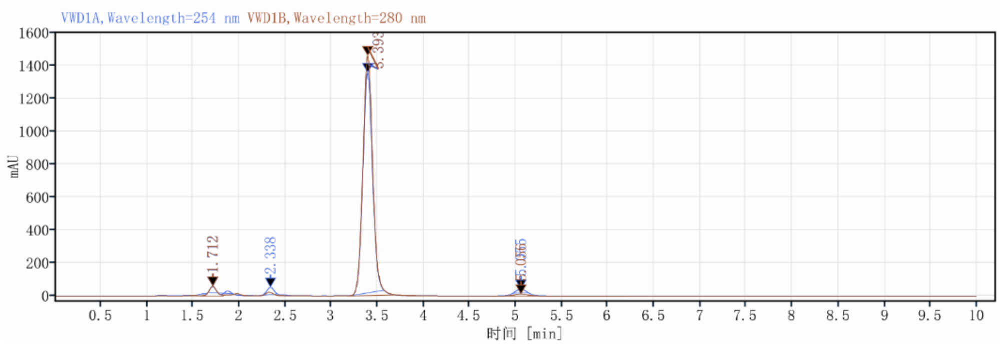

1-4f

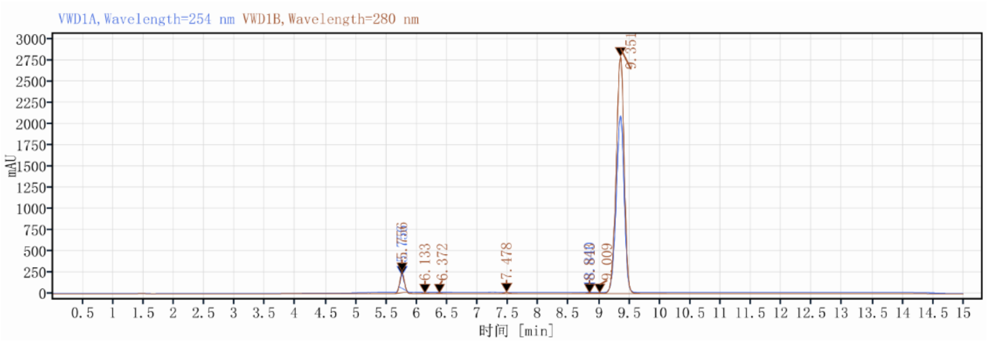

1-4g

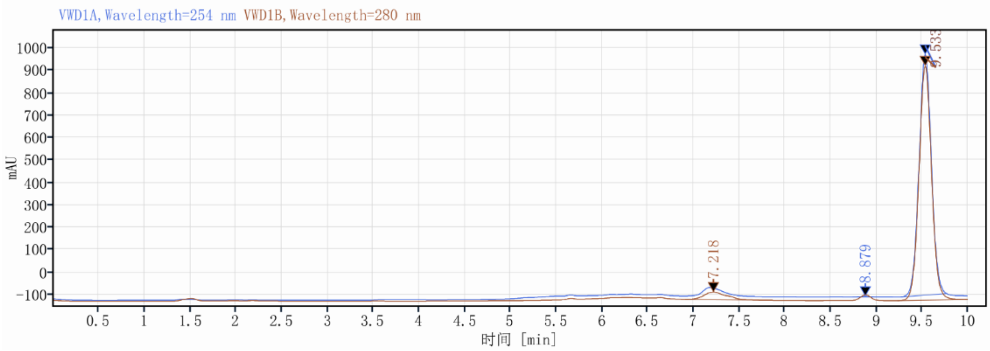

1-4h

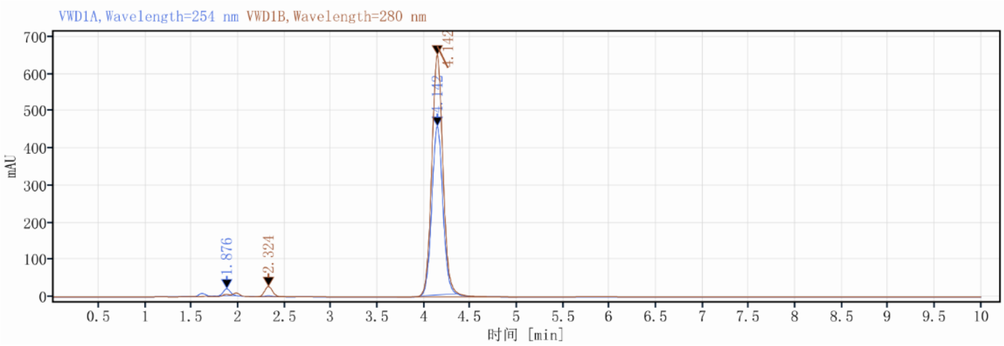

1-4i

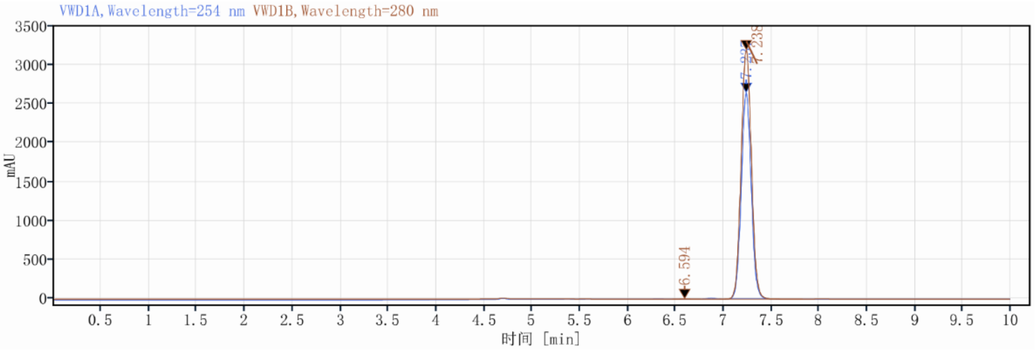

1-4j

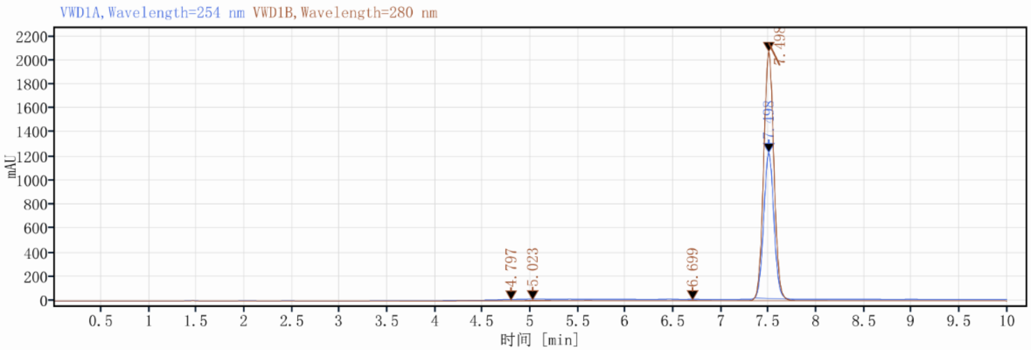

1-4k

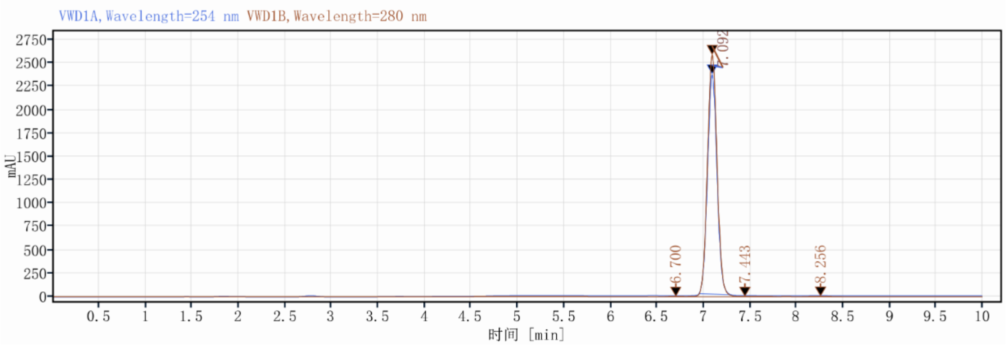

1-4l

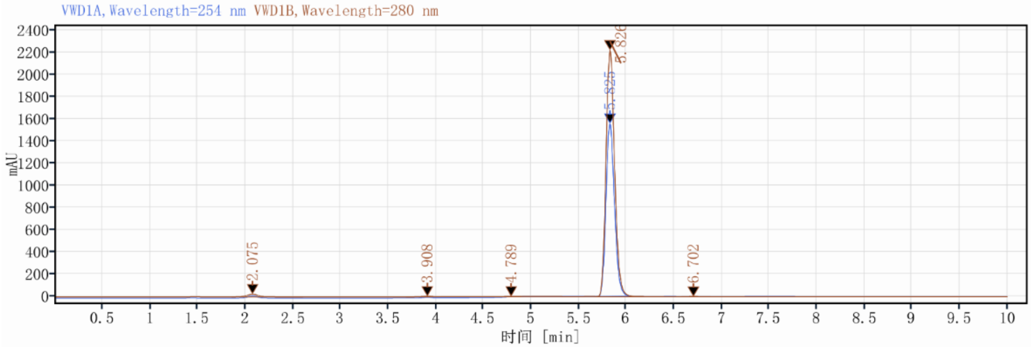

1-4m

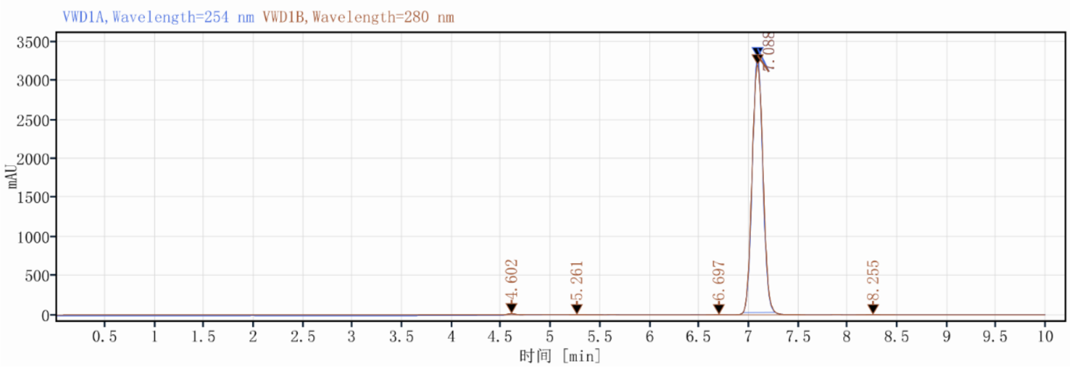

1-4n

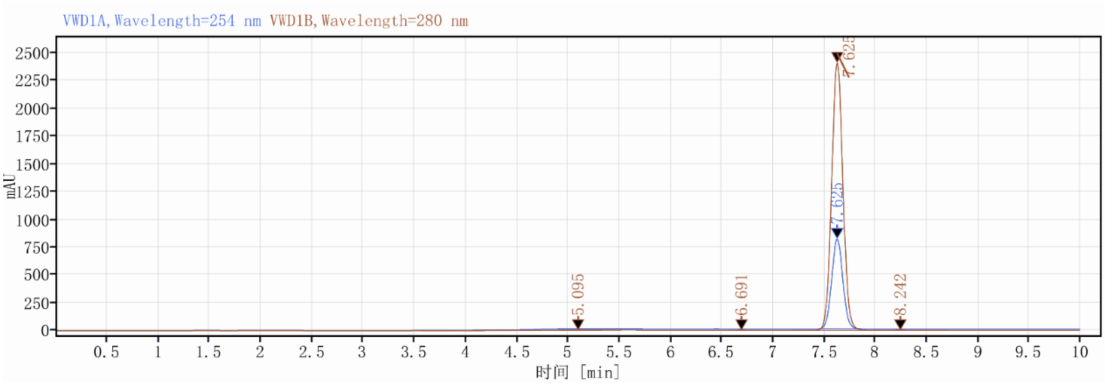

1-4o

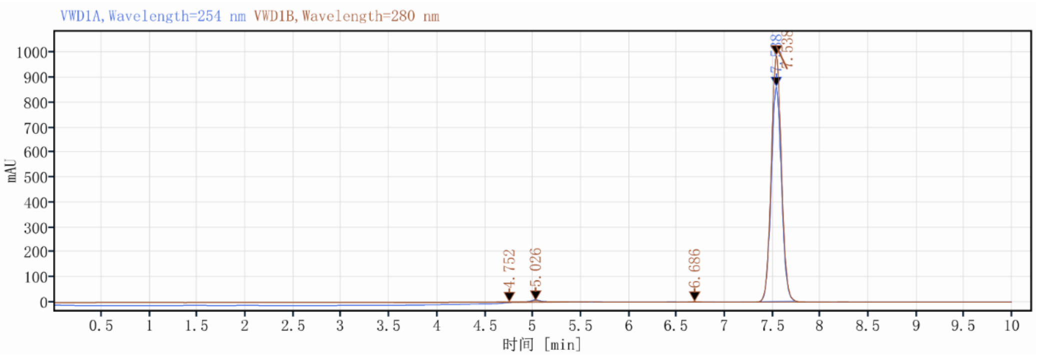

1-4p

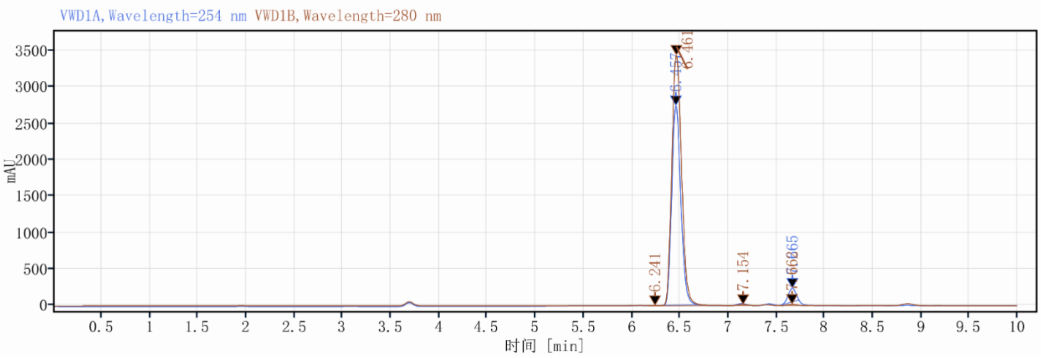

1-4q

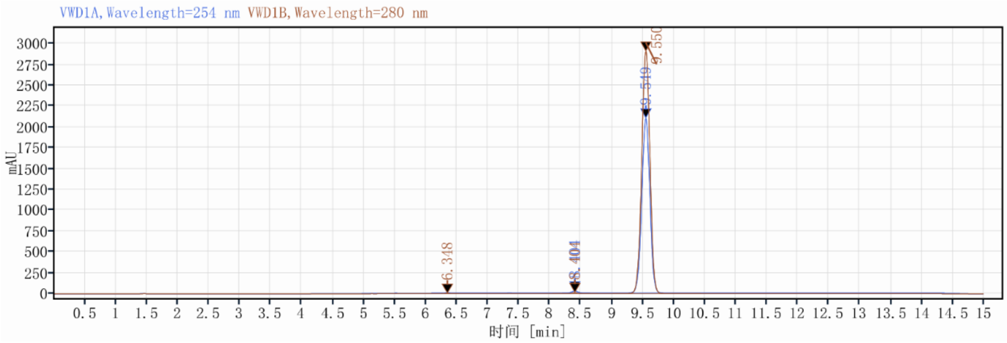

1-4r

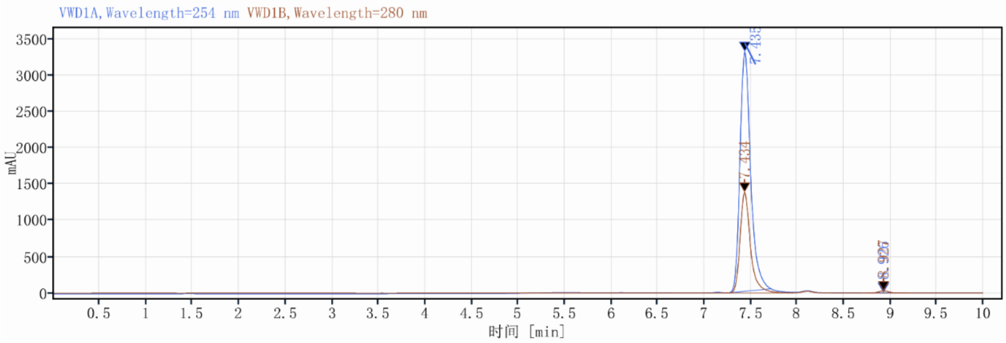

2-3a

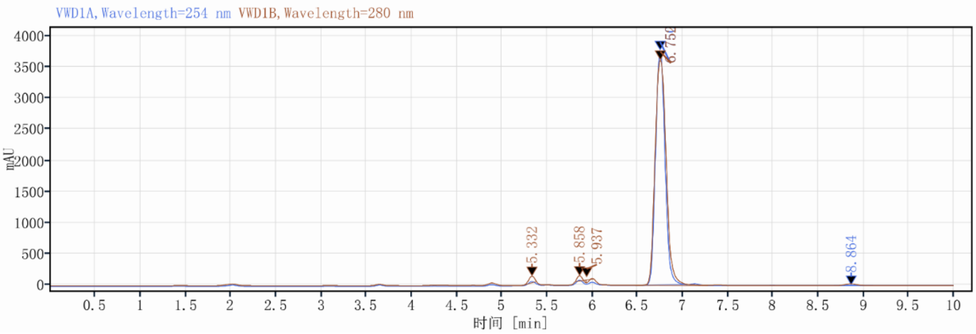

2-3b

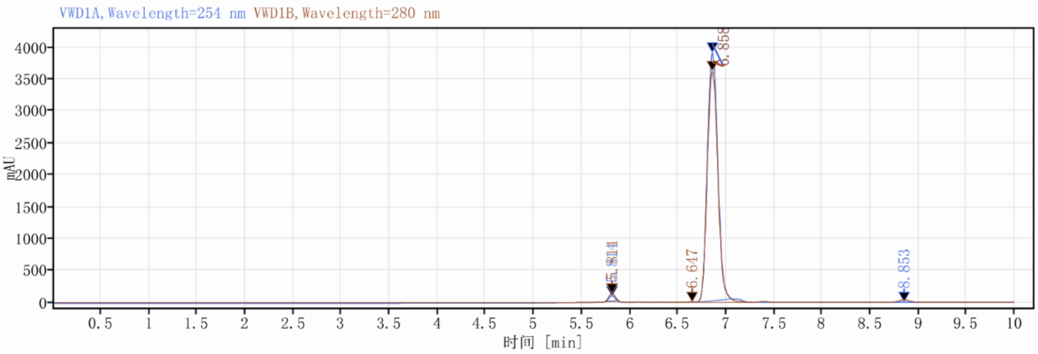

2-3c

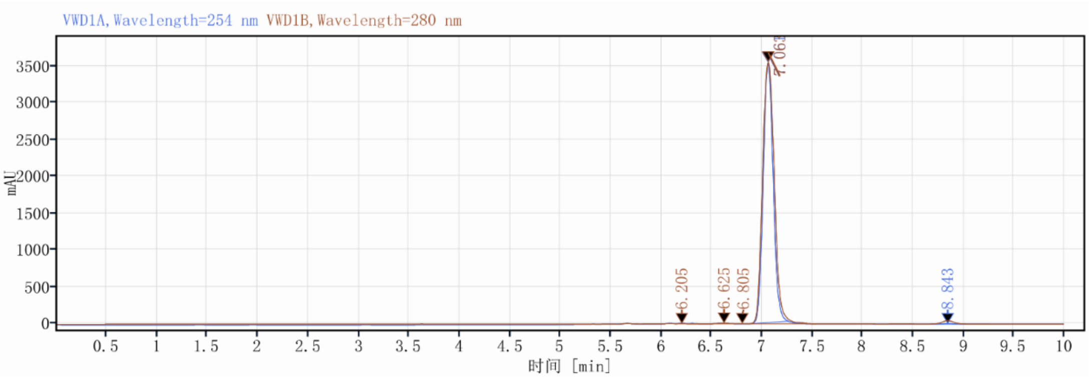

2-3d

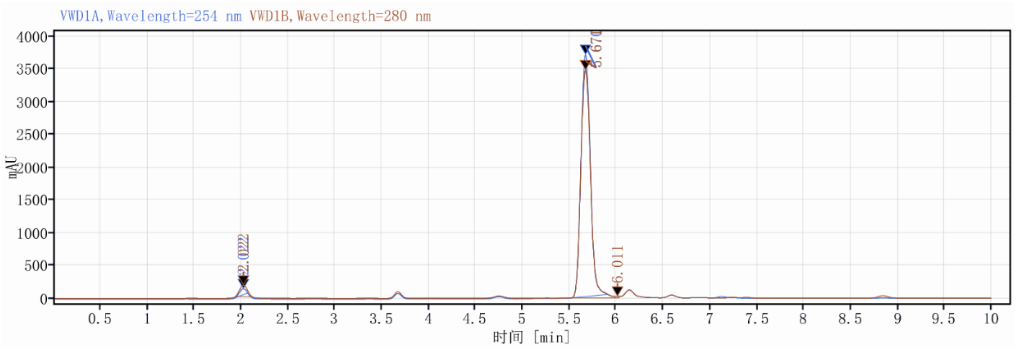

2-3e

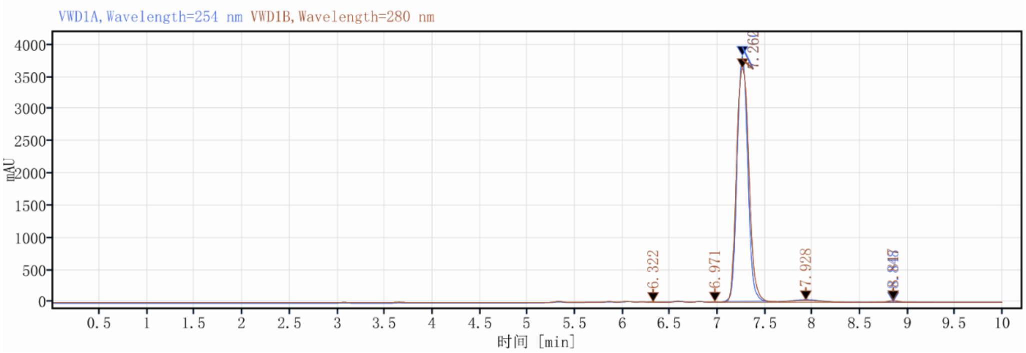

3-2a

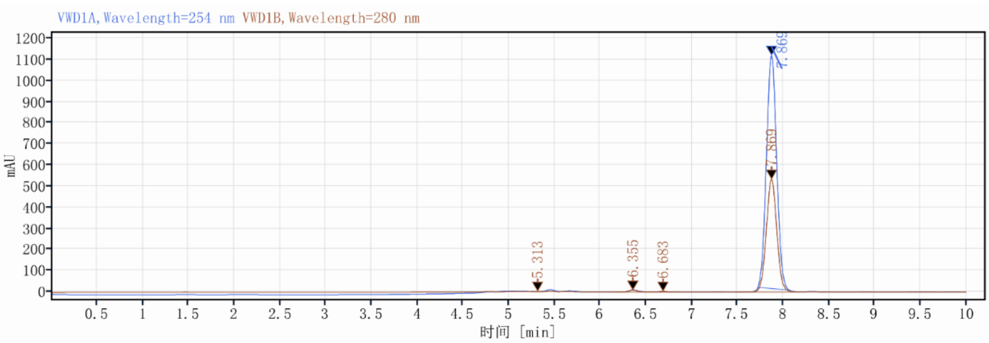

3-2b

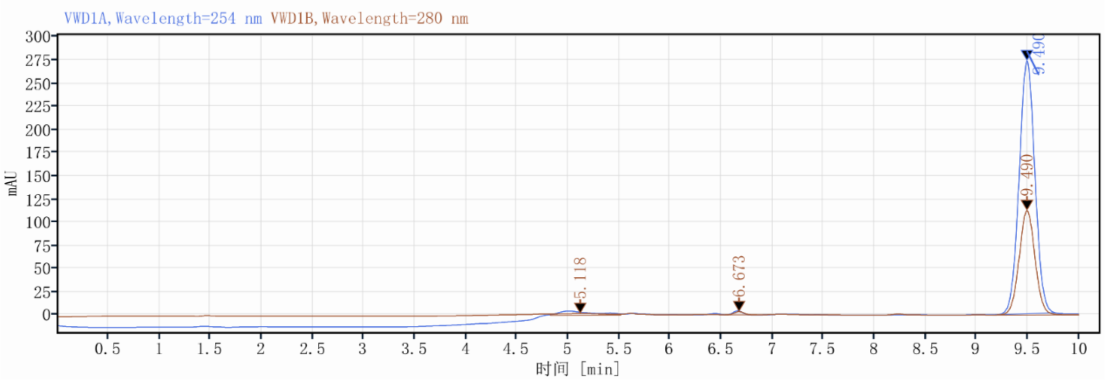

3-2c

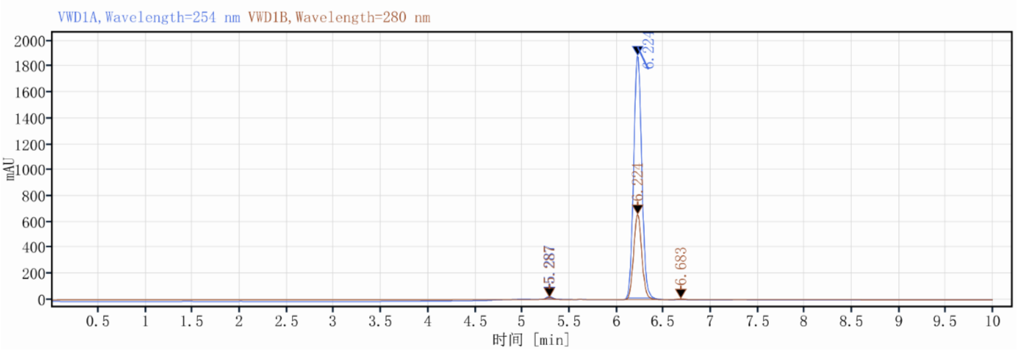

3-2d

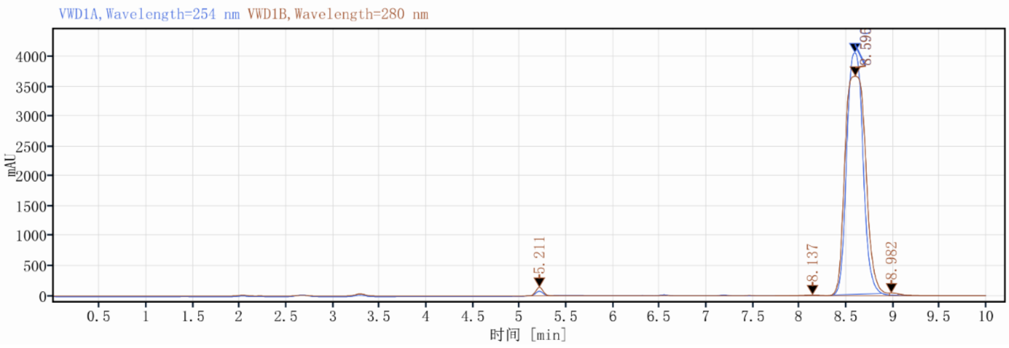

3-2e

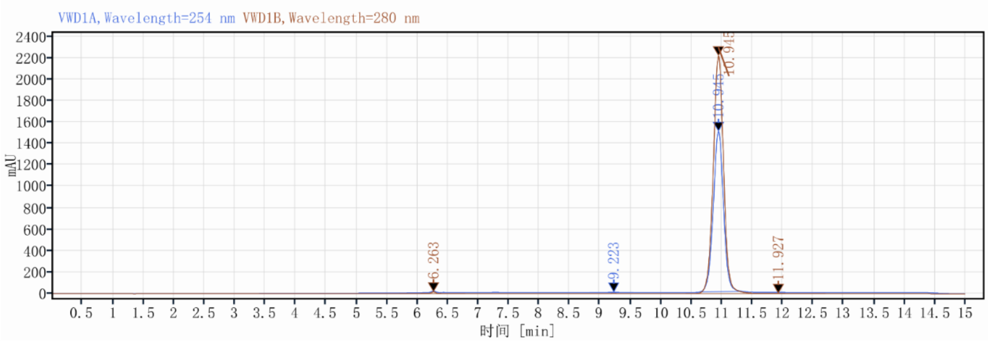

3-2f

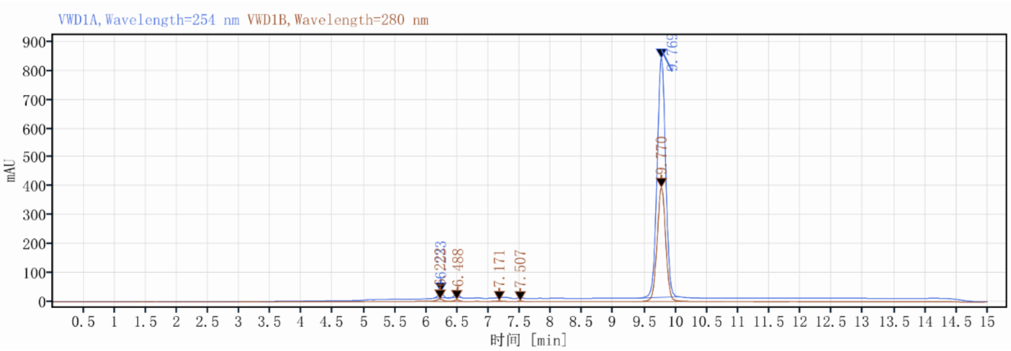

3-2g

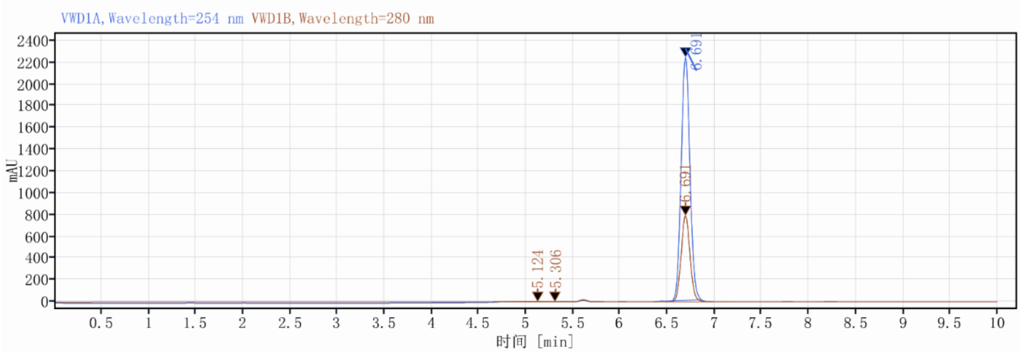

3-2h

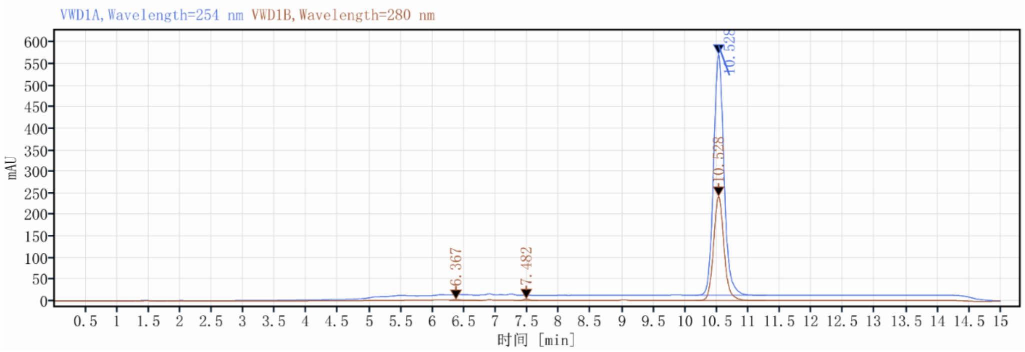

3-2i

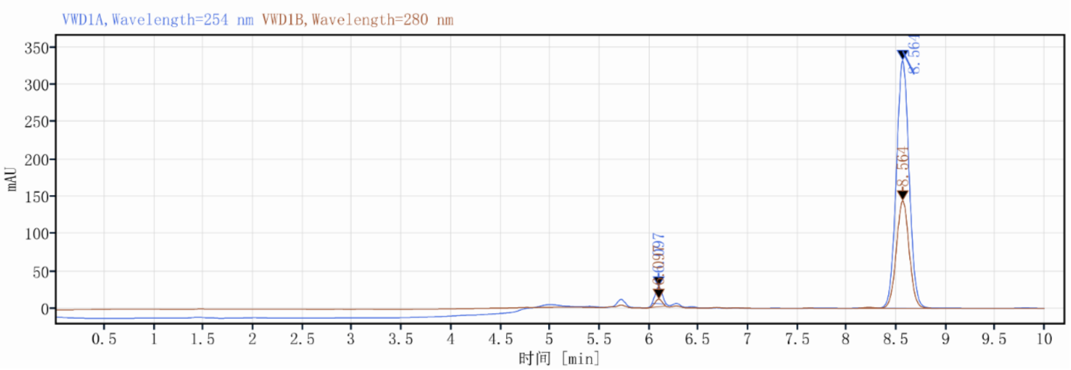

Purity of compounds was determined by Agilent 1260 Infinity II HPLC machine with Promosil C18 column from Agela Technologies (4.6 × 150 mm, 5 µm particle size). UV detection at 254 nm and 280nm. Mobile phase A was double distilled water and mobile phase B was methanol containing 0.1% trifluoroacetic acid. Flow rate was 1 ml/min using linear gradients as follow:

(1)

0min-2min: 40%B

2min-6min: 45%B-95%B

6min-7min: 95%B

7min-8min:95%B-40%B

8min-10min: 40%B

(2)

0min-2min: 40%B

2min-10min: 40%B-95%B

10min-11min: 95%B

11min-13min:95%B-40%B

13min-15min: 40%B
